# Supplementary material for: Effects of Exercise and Physical Activity Levels on Vaccination Efficacy: A Systematic Review and Meta-Analysis
Source: Vaccines (Basel). 2022 May 12;10(5):769. doi: 10.3390/vaccines10050769 (PMC9146578; doi:10.3390/vaccines10050769)
Supplement: Supplementary file 1 [file vaccines-10-00769-s001.zip › vaccines-1678135-supplementary.pdf]

Systematic Review

Effects of Exercise and Physical Activity Levels on Vaccination Efficacy: A Systematic Review and Meta-Analysis

Petros C. Dinas,[1*] Yiannis Koutedakis,[2,3] Leonidas Ioannou,[1] George Metsios,[3,4,5] George D. Kitas[5]

[1] FAME Laboratory, Department of Physical Education and Sport Science, University of Thessaly, Trikala, 42100, Greece; ioannoulg@gmail.com
[2] Department of Physical Education and Sport Science, University of Thessaly, Trikala, 42100, Greece; y.koutedakis@gmail.com
[3] Faculty of Education Health & Wellbeing, University of Wolverhampton, Walsall, WS1 3BD, UK; g.metsios@uth.gr
[4] Department of Nutrition and Dietetics, University of Thessaly, Trikala, 42100, Greece
[5] Dudley Group NHS Foundation Trust and School of Sports and Exercise Science, University of Birmingham, Birmingham, B15 2TT, UK; george.kitas@nhs.net
* Correspondence: petros.cd@gmail.com

Table of Contents

Key word algorithms 3
Supplementary Figure S1: PRISMA flow diagram 4
Supplementary Table S1: Characteristics of eligible studies 4
Risk of bias assessment 41
Supplementary Table S2: Risk of bias assessment outcomes 41
Supplementary Figure S2: Summary of risk of bias 43
Supplementary Figure S3 44
Supplementary Figure S4 45
Supplementary Figure S5 46
Supplementary Figure S6 47
Supplementary Figure S7 48
Supplementary Figure S8 49
Supplementary Figure S9 50
Supplementary Figure S10 51
Supplementary Figure S11 51
Supplementary Figure S12 52
Supplementary Figure S13 53
Supplementary Figure S14 54
Supplementary Figure S15 55
Supplementary Figure S16 56
Supplementary Figure S17 57

Supplementary Figure S18 58
Supplementary Figure S19 59
Supplementary Figure S20 60
Supplementary Figure S21 61
Supplementary Figure S22 62
Supplementary Figure S23 62
Supplementary Figure S24 63
Supplementary Figure S25 64
Supplementary Figure S26 64
Supplementary Figure S27 65
Supplementary Figure S28 65
Supplementary Figure S29 66
Supplementary Figure S30 67
Supplementary Figure S31 67
Supplementary Figure S32 68
Supplementary Figure S33 68
Supplementary Table S3: GRADE analysis 69
Supplementary Table S4: PRISMA checklist 73
List of excluded publications 77
References used in the supplement 165

Key word algorithms

PubMed

(("vaccine*"[Title/Abstract] OR "vaccination*"[Title/Abstract] OR "immune"[Title/Abstract] OR "immuniz*"[Title/Abstract] OR "immunis*"[Title/Abstract]) AND ("exercise"[Title/Abstract] OR "physical activity"[Title/Abstract] OR "physical fitness"[Title/Abstract] OR "physical exertion"[Title/Abstract] OR "sport*"[Title/Abstract] OR "dancing"[Title/Abstract] OR "walking"[Title/Abstract] OR "swimming"[Title/Abstract] OR "cycling"[Title/Abstract]) AND ("efficacy"[Title/Abstract] OR "effectiveness"[Title/Abstract] OR "antibod*"[Title/Abstract])) NOT ("animals"[MeSH Terms] NOT "humans"[MeSH Terms]).

Embase

(("vaccine*"[Title/Abstract] OR "vaccination*"[Title/Abstract] OR "immune"[Title/Abstract] OR "immuniz*"[Title/Abstract] OR "immunis*"[Title/Abstract]) AND ("exercise"[Title/Abstract] OR "physical activity"[Title/Abstract] OR "physical fitness"[Title/Abstract] OR "physical exertion"[Title/Abstract] OR "sport*"[Title/Abstract] OR "dancing"[Title/Abstract] OR "walking"[Title/Abstract] OR "swimming"[Title/Abstract] OR "cycling"[Title/Abstract]) AND ("efficacy"[Title/Abstract] OR "effectiveness"[Title/Abstract] OR "antibod*"[Title/Abstract]))

Cochrane Central Register of Controlled Trials

((("vaccine*" OR "vaccination*" OR "immune" OR "immuniz*" OR "immunis*") AND ("exercise" OR "physical activity" OR "physical fitness" OR "physical exertion" OR "sport*" OR "dancing" OR "walking" OR "swimming" OR "cycling") AND ("efficacy" OR "effectiveness" OR "antibod*"))):ti,ab,kw in Trials.

SportDiscus and CINAHL

(("vaccine*" OR "vaccination*" OR "immune" OR "immuniz*" OR "immunis*") AND ("exercise" OR "physical activity" OR "physical fitness" OR "physical exertion" OR "sport*" OR "dancing" OR "walking" OR "swimming" OR "cycling") AND ("efficacy" OR "effectiveness" OR "antibod*")).

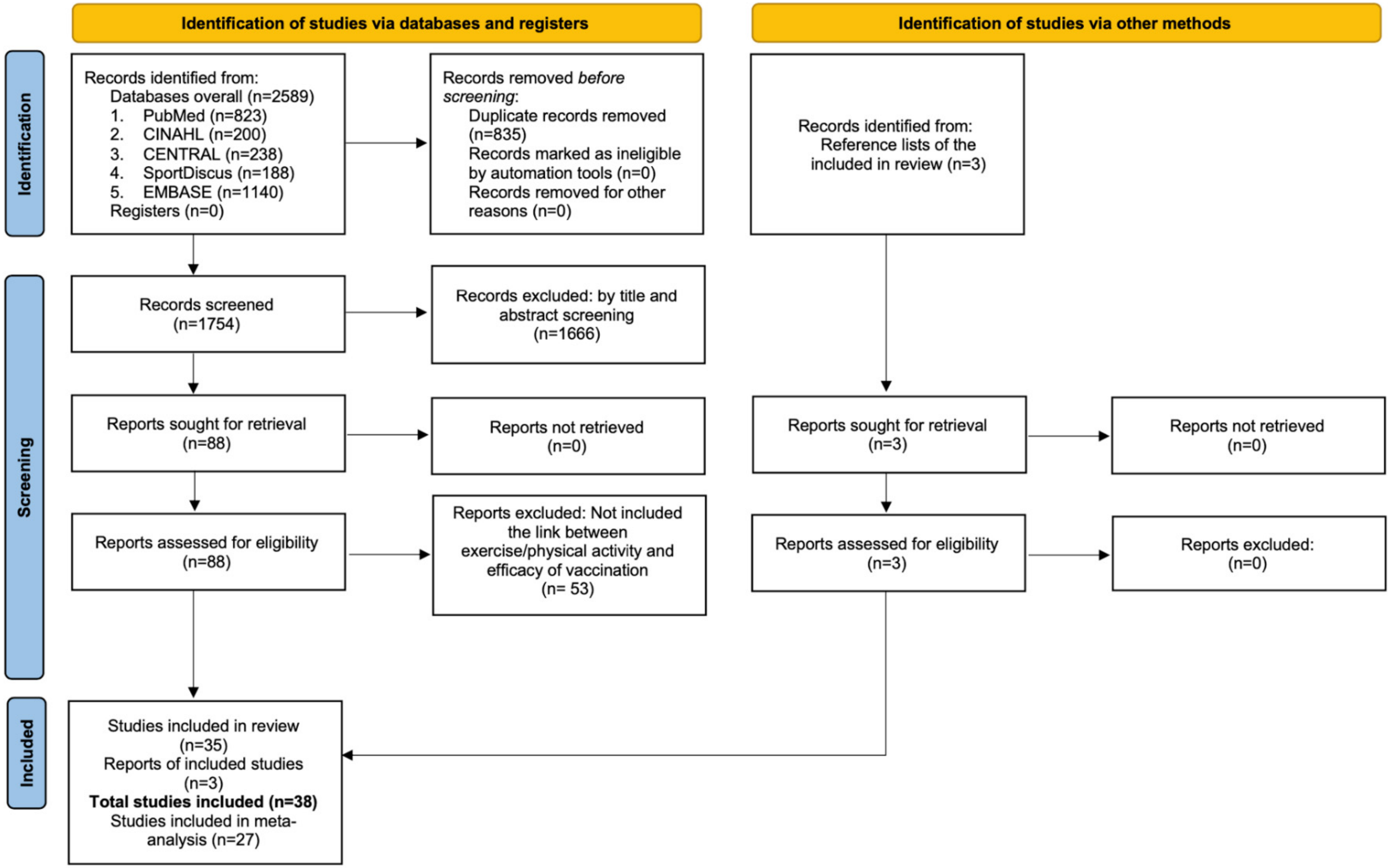

Supplementary Figure S1: PRISMA flow diagram

Supplementary Table S1: Characteristics of eligible studies

Study ID	Type of study	Design	Participants	Exercise intervention	Type & time of vaccination	Vaccination efficacy variable	Main outcome
Ayling 2018	No intervention	CSS	n=138 (61 females); Age=72.87±5.41 years;	Physical activity measurements with pedometers	Influenza vaccination: H1N1; H3N2;B	IgG titers for H1N1, H3N2 and B strains	No association between physical activity levels (steps/day) and H1N1, H3N2 and B IgG titers at 4th and 16th post vaccination.
Bachi 2013	Intervention	CT	n=110 (74 females); Age=70.29±6.89 years<br><br>1. Sedentary (n=55, females=36), age=70.7±7.1 years, BMI=29.6±4.9 kg/m^2<br><br>2. Physically active (n=55, females=38, history of 12 months of exercise), age=69.6±6.8 years, BMI=25.4±3.5 kg/m^2	Exercise for 4 weeks<br>1. Aerobic exercise 60-70% of VO2max<br>2. Resistance training for 2-3 days/week, 5-10 different exercises at 50-60% of 1RM	Influenza vaccination before the exercise intervention	IgG and IgM titers for influenza vaccine	Post-vaccination antibody levels increased significantly in physically active and the increase in IgM and IgG levels being statistically greater in the physically active than in the sedentary. Physically active women showed higher IgM and IgG post-vaccination not only in relation to values before vaccine, but also to the levels observed post-vaccination in the women of sedentary group. Physically active men showed IgM and IgG significantly higher than the values before vaccine.<br>1. IgM titers pre-vaccination:<br>Sedentary=0.061±0.076<br>Physically active=0.072±0.084<br>IgM titers post-vaccination:<br>Sedentary=0.115±0.111<br>Physically active=0.200±0.143<br>2. IgG titers pre-vaccination:<br>Sedentary=0.291±0.219<br>Physically active=0.270±0.176<br>IgG titers post-vaccination:<br>Sedentary=0.357±0.195<br>Physically active=0.491±0.214<br>3. Women IgM titers pre-vaccination:<br>Sedentary=0.075±0.075<br>Physically active=0.062±0.081<br>IgM titers post-vaccination:<br>Sedentary=0.107±0.085

							Physically active=0.203±0.121<br>4. Men IgM titers pre-vaccination:<br>Sedentary=0.055±0.050<br>Physically active=0.035±0.030<br>IgM titers post-vaccination:<br>Sedentary=0.070±0.054<br>Physically active=0.144±0.089<br>5. Women IgG titers pre-vaccination:<br>Sedentary=0.321±0.231<br>Physically active=0.283±0.164<br>IgM titers post-vaccination:<br>Sedentary=0.381±0.202<br>Physically active=0.530±0.212<br>6. Men IgG titers pre-vaccination:<br>Sedentary=0.208±0.099<br>Physically active=0.197±0.047<br>IgM titers post-vaccination:<br>Sedentary=0.223±0.147<br>Physically active=0.507±0.194
Bohn-Goldbaum 2019	Intervention	RCT	Healthy students n=45; 17 males and 28 females<br>1. Exercise group (n=22), age=12.4±0.3 years, 9 m, BMI=20.06 kg/m^2<br>2. Control group (n=23), age=12.4±0.2 years, 8 m, BMI=18.99 kg/m^2	Acute exercise session of 15 minutes, upper body resistance exercise using elastic resistance bands before receiving their vaccination	Human papillomavirus (Gardasil) vaccine at the time of exercise session for each dose	1. HPV16<br>2. HPV18	No differences between exercise and control group in antibody titers after the third dose of vaccination and at 7.5 months post vaccination.<br>1. HPV16 after third dose: Exercise=8.2±1.2, Control=8.2±1<br>2. HPV16 at 7.5 months post vaccination: Exercise=10.2±0.9, Control=10±1.3<br>3. HPV18 after third dose: Exercise=8.4±0.9, Control=8.5±1.1<br>4. HPV18 at 7.5 months post vaccination: Exercise=10.4±0.9, Control=10.3±0.9
Bohn-Goldbaum 2020	Intervention	RCT	Healthy (n=46)<br>1. Exercise group (n=23), 11 males, age=74.4±6.5 years; Body fat=32.3±9.3;	Acute resistance exercise for 45 minutes at 60% 1 RM, 5 exercises, 8 repetitions with 2-3	Infuenza vaccination Vaxigrip, Sano"	1.A/California/7/2009 (H1N1)<br>2. A/Perth/16/2009 (H3N2)	No differences between groups at any timepoint.<br>1. A/California/7/2009 (H1N1), Exercise group baseline: 0.74±0.642; Control group baseline: 0.80±0.730;

			physical activity PASE score 113.1±52.2<br>2. Control group (n=23), 11 males, age=72.3±6.7 years; Body fat=35.9±7.9; physical activity PASE score 89.5±42.7	seconds recovery/exercise, 60-90 seconds recovery/set	Pasteur 0.5 mL, batch no. H8333 after the exercise intervention	3.B/Brisbane/60/ 2008	Exercise group 1 month: 1.58±0.647; Control group 1 month: 1.81±0.0653.<br>2. A/Perth/16/2009 (H3N2)<br>Exercise group baseline: 0.94±0.815; Control group baseline: 1.21±0.717;<br>Exercise group 1 month: 1.91±0.556; Control group 1 month: 2.10±0.460.<br>3.B/Brisbane/60/ 2008<br>Exercise group baseline: 1.23±0.501; Control group baseline: 1.17±0.619;<br>Exercise group 1 month: 1.70±0.484; Control group 1 month: 1.83±0.5.
Bruunsgaard 1997	Intervention	CT	1.Healthy males triathletes (n=22), age=34±9.25 years,<br>2.Healthy males non-exercising triathletes (n=11), age=29±6.95 years<br>3.Healthy males moderately trained (n=22), age=32.75±7.80 years	Acute aerobic exercise training (triathletes competition) : 3 km swimming, 130 km bicycling and 21 km running.	1. Diphtheria and tetanus toxoid (Di-Te, SSI, Copenhagen, Denmark) after the exercise session<br><br>2.Pneumococcal polysaccharide vaccine (Pneumovax 23, Merck Sharp and Dohme, West Point, PA) after the exercise session	1.Cell mediated immunity<br><br>2.Antibody titers against diphtheria and tetanus	1. Diphtheria (14-day post vaccination)<br>Exercising triathletes (n=22): 9.97±15.24<br>Non-exercising triathletes (n=11): 4.86±5.95<br>Non exercising individuals (n=22): 14.91±24.04<br>2. Tetanus (14-day post vaccination)<br>Exercising triathletes (n=22): 35.07±13.92<br>Non-exercising triathletes (n=11): 24.16±23.24<br>Non exercising individuals (n=22): 25.47±17.33<br>3. Pneumococcal (14-day post vaccination)<br>Exercising triathletes (n=22): 141±105.93<br>Non-exercising triathletes (n=11): 108.33±65.36<br>Non exercising individuals (n=22): 122.67±84.15<br>4. Exercised healthy triathletes revealed a significantly lower response to the tetanus antigen compared with both non-exercised healthy triathletes and non-exercised healthy. Also, exercised healthy triathletes responded less to stimulation with diphtheritis antigen than non-exercised healthy triathletes and less to stimulation

							with tuberculin antigen than non-exercised healthy.
Campbell 2010	Intervention	RCT	Healthy (n=156); 76 males; age= 20.38±2.35 years<br><br>1. Immediate post-vaccination exercise group (n=38)<br>2. 6 hours post-vaccination exercise group (n=39)<br>2. 48 hours exercise post-vaccination group (n=39)<br>3. Control group (n=39)	Acute resistance exercise protocol; Eccentric portions of the bicep curl and lateral raise exercises, contracting the biceps bracchi and deltoid muscles of the non-dominant arm, at 85% concentric 1RM; 50 repetitions of each movement for 25 minutes	Influenza vaccine (Inactivated Split Virion BP, Sanofi Pasteur MSD, Batch No. B9676-1)<br>After the exercise program	1. A/Solomon Islands<br>2. A/Wisconsin<br>3. B/Malaysia<br>4. IFN-γ in cell	1. <u>A/Solomon Islands</u><br>Immediate group baseline:1.08±0.61<br>Immediate group 28th day:2.69±0.49<br>6 hours group baseline: 1.09±0.75<br>6 hours group 28th day: 1.09±0.75<br>48 hours group baseline: 1.09±0.75<br>48 hours group 28th day: 2.80±0.62<br>Control group baseline: 1.09±0.75<br>Control group 28th day:2.80±0.62<br>2. <u>A/Wisconsin</u><br>Immediate group baseline:1.21±0.68<br>Immediate group 28th day:2.53±0.43<br>6 hours group baseline:1.13±0.69<br>6 hours group 28th day:2.31±0.56<br>48 hours group baseline:0.96±0.56<br>48 hours group 28th day:2.50±0.5<br>Control group baseline:1.19±0.75<br>Control group 28th day:2.30±0.62<br>3. <u>B/Malaysia</u><br>Immediate group baseline:0.78±0.49<br>Immediate group 28th day:2.37±0.49<br>6 hours group baseline: 0.91±0.62<br>6 hours group 28th day:2.16±0.69<br>48 hours group baseline:0.90±0.56<br>48 hours group 28th day:2.32±0.56<br>Control group baseline: 0.91±0.62<br>Control group 28th day:2.55±0.62<br>4. No cell responses for IFN-γ were found in combined exercised and control group data
de Araújo 2015	No intervention	CSS	Healthy (n=61)<br><br>1. Never trained group (n=16): age=72.9±6 years, BMI=25.3±3.2	Measurements for physical activity levels via IPAQ and VO2max via treadmill test	Influenza vaccination	<u>2012 vaccination</u><br>1. A/California/7/2009 (H1N1)	The trained groups had higher titers than the non-trained group at baseline and at 6 weeks. At 6 months the moderately trained group had higher titers that the non-trained group.

			kg/m^2, METs min/week=144.7±258.8, VO2max=24.3±2.4 ml/kg/min<br><br>2. Moderately trained group (n=23): age=70.4±3.36 years, BMI=24.7±1.92 kg/m^2, METs min/week=1999±750.55, VO2max=29.3±4.79 ml/kg/min<br><br>3. Intensely trained group (n=22): age=74.8±7.03 years, BMI=23±1.88 kg/m^2, METs min/week=4587±1275.79, VO2max=35±6.57 ml/kg/min			2. A/Perth/16/2009 (H3N2)<br>3. B/Brisbane/60/2008<br><br><u>2013 vaccination</u><br>1. A/California/7/2009 (H1N1)<br>2. A/Victoria/361/2011 (H3N2)<br>3. B/Wisconsin/1/2010	<u>H1N1</u><br>Untrained 6 weeks (n=16): 99.38±193.72<br>Moderately trained 6 weeks (n=23): 171.09±274.51<br>Intensely trained 6 weeks (n=22): 195.62±150.47<br>Untrained 6 months (n=16): 52.84±125.8<br>Moderately trained 6 months (n=23): 130.20±211.16<br>Intensely trained 6 months (n=22): 164.80±330.44<br><u>H3N2</u><br>Untrained 6 weeks (n=16): 117.65±231.08<br>Moderately trained 6 weeks (n=23): 196.26±257.06<br>Intensely trained 6 weeks (n=22): 217.70±273.73<br>Untrained 6 months (n=16): 89.22±169.12<br>Moderately trained 6 months (n=23): 146.98±231.35<br>Intensely trained 6 months (n=22): 174.97±259.75<br><u>Influenza B</u><br>Untrained 6 weeks (n=16): 23.43±49.76<br>Moderately trained 6 weeks (n=23): 43.64±74.43<br>Intensely trained 6 weeks (n=22): 53.01±69<br>Untrained 6 months (n=16): 17.13±40.72<br>Moderately trained 6 months (n=23): 39.27±63.54<br>Intensely trained 6 months (n=22): 35.23±69
Edwards 2008	Intervention	RCT	Healthy (n=60), n=20 for exercise, mental stress and control; 29 males; age: 22±3.16 years; BMI: 23.6±2.78 kg/m^2	Acute incremental cycling session from 84W-231W for men and from 70W-154W for women, 4 minutes/step.	1. Meningococcal A"C (Mengivac A"C, Aventis	1. Meningococcal A (IgG)<br>2. Meningococcal C (IgG)	<u>Meningococcal A (IgG)</u><br>1. For men, exercise associated with improved responses compared with control

					Pasteur;<br>Batch#<br>W0483-4)<br><br>Vaccine after the exercise session.<br>Antibodies measurements at 4th and 20th weeks		2. For women, who had a stronger response in the control condition, exercise did not significantly alter the response.<br>Meningococcal C (IgG)<br>Antibody levels increasing from baseline to 4-week follow-up and remaining higher than baseline at the 20-week follow-up<br>Meningococcal A Men 4th week<br>1. Exercise (mean±SD)=10.31±1,33<br>2. Control=9.49±0,70<br>Meningococcal A Men 20th week<br>1. Exercise (mean±SD)=9.99±0.99<br>2. Control=9.18±0.72<br>Meningococcal A Women 4th week<br>1. Exercise (mean±SD)=9.03±1.35<br>2. Control=10.24±1.09<br>Meningococcal A Women 20th week<br>1. Exercise (mean±SD)=8.99±1.55<br>2. Control=10.42±1.29<br>Meningococcal C Men 4th week<br>1. Exercise (mean±SD)=10.99±0.94<br>2. Control=10.59±1<br>Meningococcal C Men 20th week<br>1. Exercise (mean±SD)=10.60±0.88<br>2. Control=9.89±1.56<br>Meningococcal C Women 4th week<br>1. Exercise (mean±SD)=10.20±1.03<br>2. Control=10.77±1.29<br>Meningococcal C Women 20th week<br>1. Exercise (mean±SD)=9.75±1.03<br>2. Control=10.51±1.31
Edwards 2007	Intervention	RCT	Healthy (n=60);<br>1. 29 males, age: 20.1±1.64 years; BMI: 23.9±2.67 kg/m^2	Acute eccentric resistance training at 85% of 1RM four times, at baseline and at 6th, 8th,20th week post-vaccination.	Influenza vaccination after the exercise session	1. A/Fujian/411/2002 (H3N2): A/Wyoming/3/2003(x-147)	A/Wyoming antibodies<br>1. 6 weeks women exercise (n=20):748.50±923.90<br>2. 6 weeks women control (n=11):601.80±367.41<br>3. 6 weeks men exercise (n=18): 410.18±304.87

	2. 31 females; age: 20.6±2.55 years; BMI: 23.8±3.21 kg/m²<br><br>Exercise group (n=40, 20 males and 20 females), control group (n=20, 9 males and 11 females)<br><br>Physical activity status of participants was: 17.2±8.01 hours/week for males and 15.1±5.47 hours/week for females	Eccentric portions of the bicep curl and lateral raise exercises, contracting the biceps bracchi and deltoid muscles of the non-dominant arm; 50 repetitions of each movement for 25 minutes		2. A/New Caledonia/20/99 (H1N1): A/New Caledonia/20/99 (IVR-116)<br><br>3. B/Shanghai/361/2002: B/Jiangsu/10/2003<br><br>4. Cell response IFN-γ	3. 6 weeks men control (n=8):730.54±448.81<br>4. 20 weeks women exercise (n=20):520.96±495.42<br>5. 20 weeks women control (n=11):308.38±228.38<br>6. 20 weeks men exercise (n=17):314.37±246.89<br>7. 20 weeks men control (n=9):538.92±574.86<br><u>A/New Caledonia antibodies</u><br>1. 6 weeks women exercise (n=20):1602.69±4637.78<br>2. 6 weeks women control (n=11):700.34±1027.36<br>3. 6 weeks men exercise (n=18):1966.33±2856.99<br>4. 6 weeks men control (n=8):2909.09±4799,75<br>5. 20 weeks women exercise (n=20):821.55±1144.37<br>6. 20 weeks women control (n=11):417.51±670.02<br>7. 20 weeks men exercise (n=17):1212.12±1776.98<br>8. 20 weeks men control (n=9):1333.33±2303.04<br><u>B/Shanghai antibodies</u><br>1. 6 weeks women exercise (n=20):429.55±862.13<br>2. 6 weeks women control (n=11):299.72±437.13<br>3. 6 weeks men exercise (n=18):175.78±183.62<br>4. 6 weeks men control (n=8):398.08±467.40<br>5. 20 weeks women exercise (n=20):220±316.72<br>6. 20 weeks women control (n=11):159.01±254.45<br>7. 20 weeks men exercise (n=17):96.06±105.47<br>8. 20 weeks men control (n=9):259.34±442.62

							<u>Cell response IFN-γ 8th week</u><br>1. Exercise men (n=18):5.18±3.01<br>2. Control men (n=9):2.95±1.74<br>3. Exercise women (n=17):5.05±4.91<br>4. Control women (11):4±4.28
Edwards 2006	Intervention	RCT	Healthy (n=60); 31 males, 29 females; age: 22±3.16 years; BMI: 23.6±2.78 kg/m^2	1. Acute incremental cycling session (70 rpm) group (n=20); four step session, 4 min/step at 231 W for m and 154 W for f.<br>2. No exercise control group (n=20) reading in a sitting position for 45 min	Influenza vaccination after the exercise session (measurements at 4th and 20th week after exercise session)	1. A/Panama/2007/99(RESVIR-17)<br>2. A/New Caledonia/20/99 (IVR-116)<br>3. B/Shangdong/7/97	1. Females in the exercise group display higher antibody titers than the control group for the A/Panama/2007/99(RESVIR-17), (Exercise group mean±SD 4wk=2.46±0.38; Control group 4wk=2.11±1,86); (Exercise group mean±SD 20wk=2.32±0.44; Control group 20wk=2.01±0.41).<br>2. No mean differences for female between exercise and control for the A/New Caledonia/20/99 (IVR-116). (Exercise group 4wk=3.24±0.63; Control group 4wk=3.11±0.63); (Exercise group 20wk=2.89±0.54; Control group 20wk=2.84±0.19).<br>3. No mean differences for female between exercise and control for B/Shangdong/7/97. (Exercise group 4wk=2.73±0.47; Control group 4wk=2.23±0.73); (Exercise group 20wk=2.49±0.69; Control group 20wk=1.94±0.76).<br><br>4. No mean differences for male the A/Panama/2007/99(RESVIR-17), (Exercise group 4wk=2.59±0.28; Control group 4wk=2.53±0.19); (Exercise group 20wk=2.37±0.35; Control group 20wk=2.28±0.51).<br>5. No mean differences for male between exercise and control for the A/New Caledonia/20/99 (IVR-116). (Exercise group 4wk=2.95±0.57; Control group 4wk=3.43±0.38); (Exercise group

							20wk=2.76±0.44; Control group 20wk= 3.08±0.63).<br>6. No mean differences for male between exercise and control for B/Shangdong/7/97. (Exercise group 4wk=2.66±0.47; Control group 4wk=2.71±0.41); (Exercise group 20wk=2.39±0.63; Control group 20wk=2.53±0.35).
Edwards 2010	Intervention	RCT	Exercise groups healthy (n=158), 78 males; age=20.5±1.6 years; BMI=21.6±2.6 kg/m^2<br><br>Control group healthy (n=39), 19 males; age=20.8±2 years; BMI=20.6±2.3 kg/m^2	Acute resistance exercise at 60%, 85% and 110% of the 1RM.	Influenza vaccination after the exercise session (baseline after vaccination and 28 days after vaccination)	1. A/Brisbane<br>2. A/Uruguay<br>3. B/Florida	<u>A/Brisbane</u><br>Control 1 day:16±18.51<br>Control 28 days:333±752.71<br>Exercise 60% 1 day:22±50.03<br>Exercise 60% 28 days:425±1078.75<br>Exercise 85% 1 day:19±40.65<br>Exercise 85% 28 days:323±787,95<br>Exercise 110% 1 day:15±21.59<br>Exercise 110% 28 days:440±1200.02<br><u>A/Uruguay</u><br>Control 1 day:17±30.85<br>Control 28 days:104±323.91<br>Exercise 60% 1 day:17±21.89<br>Exercise 60% 28 days:116±315.81<br>Exercise 85% 1 day:15±18.76<br>Exercise 85% 28 days:129±415.86<br>Exercise 110% 1 day:14±18.51<br>Exercise 110% 28 days:61±188.18<br><u>B/Florida</u><br>Control 1 day:388±620.06<br>Control 28 days:1220±1163<br>Exercise 60% 1 day:197±547.19<br>Exercise 60% 28 days:1433±2170<br>Exercise 85% 1 day:180±444.01<br>Exercise 85% 28 days:813±1341.40<br>Exercise 110% 1 day:270±524.43<br>Exercise 110% 28 days:1220±1894.11

Edwards 2012	Intervention	RCT	1.Healthy (n=133), 58 males, age=22±2.7 years; BMI=23.1±3.8 kg/m^2<br><br><u>Groups</u><br>1. Full dose vaccine exercise (n=30)<br>2. Full dose vaccine control (n=35)<br>3. Half dose vaccine exercise (n=32)<br>4. Half dose vaccine control (n=31)	Acute exercise session for 15 min. Elastic resistance bands in sets of 30 seconds followed by 30 seconds rest for lateral raise, upright row and chest press as many repetitions the participants could perform	Polysaccharide pneumococcal vaccine, Pneumovax 23, Lot# 12464, Merck, West Point, PA, USA<br><br>After the exercise session.	Antibody strains:<br>1<br>3<br>4<br>5<br>6B<br>7F<br>9V<br>14<br>18C<br>19A<br>23F	<u>Strain 1</u><br>Full: Baseline exercise=0.81±10.52<br>Full: exercise 28 days=20.88±346.59<br>Full: Baseline control=0.95±13.16<br>Full: control 28 days=17.52±347.67<br>Half: Baseline exercise =0.94±11.65<br>Half: exercise 28 days=18.44±387.56<br>Half: Baseline control =0.92±13.33<br>Half: control 28 days=7.87±105.37<br><u>Strain 3</u><br>Full: Baseline exercise =0.34±4.23<br>Full: exercise 28 days=2.53±73.62<br>Full: Baseline control =0.23±2.74<br>Full: control 28 days=0.9±16.71<br>Half: Baseline exercise =0.23±3.19<br>Half: exercise 28 days=1.87±41.72<br>Half: Baseline control =0.31±2.64<br>Half: control 28 days=0.91±14.31<br><u>Strain 4</u><br>Full: Baseline exercise =0.15±0.88<br>Full: exercise 28 days=0.69±6.29<br>Full: Baseline control =0.2±1.28<br>Full: control 28 days=0.81±6.08<br>Half: Baseline exercise =0.15±1.05<br>Half: exercise 28 days=0.77±5.88<br>Half: Baseline control =0.21±1.39<br>Half: control 28 days=0.61±4.42<br><u>Strain 5</u><br>Full: Baseline exercise =0.53±4.79<br>Full: exercise 28 days=9.99±125.87<br>Full: Baseline control =0.27±2.68<br>Full: control 28 days=4.91±85.76<br>Half: Baseline exercise =0.45±8.24<br>Half: exercise 28 days=10.77±263.99<br>Half: Baseline control =0.4±5.15<br>Half: control 28 days=4.15±75.49

Strain 6B
Full: Baseline exercise =0.23±4.31
Full: exercise 28 days=2.31±77.26
Full: Baseline control =0.16±2.74
Full: control 28 days=1.25±23.43
Half: Baseline exercise =0.18±2.52
Half: exercise 28 days=2.21±62.96
Half: Baseline control =0.14±3.03
Half: control 28 days=1.11±28.87
Strain 7F
Full: Baseline exercise =0.49±3.21
Full: exercise 28 days=3.08±29.08
Full: Baseline control =0.48±3.44
Full: control 28 days=2.97±31.47
Half: Baseline exercise =0.52±4.44
Half: exercise 28 days=3.28±37.61
Half: Baseline control =0.43±3.95
Half: control 28 days=2.76±20.64
Strain 9V
Full: Baseline exercise =0.11±1.07
Full: exercise 28 days=1.69±41.43
Full: Baseline control =0.13±1.48
Full: control 28 days=1.03±23.52
Half: Baseline exercise =0.09±1.53
Half: exercise 28 days=1.35±30.65
Half: Baseline control =0.11±1.77
Half: control 28 days=0.65±9.87
Strain 14
Full: Baseline exercise =0.24±3.91
Full: exercise 28 days=2.67±56.77
Full: Baseline control =0.13±3.84
Full: control 28 days=2.05±42.50
Half: Baseline exercise =0.15±3.88
Half: exercise 28 days=1.56±41.05
Half: Baseline control =0.13±1.66
Half: control 28 days=1.73±38.17
Strain 18C

							Full: Baseline exercise =0.29±5.46<br>Full: exercise 28 days=5.01±120.06<br>Full: Baseline control =0.36±7.16<br>Full: control 28 days=6.84±193.91<br>Half: Baseline exercise =0.2±3<br>Half: exercise 28 days=6.34±101.21<br>Half: Baseline control =0.62±10.58<br>Half: control 28 days=9.05±203.24<br><u>Strain 19A</u><br>Full: Baseline exercise =0.8±8.44<br>Full: exercise 28 days=6.45±47.48<br>Full: Baseline control =0.96±11.15<br>Full: control 28 days=4.85±61.02<br>Half: Baseline exercise =0.6±3.94<br>Half: exercise 28 days=3.86±33.78<br>Half: Baseline control =0.74±6.46<br>Half: control 28 days=3.66±32.55<br><u>Strain 23F</u><br>Full: Baseline exercise e =1.01±2.01<br>Full: exercise 28 days=1.54±49.36<br>Full: Baseline control =0.15±2.5<br>Full: control 28 days=1.14±28.30<br>Half: Baseline exercise =0.16±3<br>Half: exercise 28 days=1.09±28.96<br>Half: Baseline control =0.15±2.26<br>Half: control 28 days=0.99±24.95
Elzayat 2021	Intervention	RCT	Healthy non-smokers<br><u>Groups:</u><br>Control: n=10; 3 males; age=70.9±5.7 years; BMI=26.2±4.6 kg/m^2<br>Ex-S: vaccination in the same arm (eccentric exercise of the deltoid and biceps bracchi): n=10; 3 males;	Acute resistance exercise session:<br>10 sets of 5 repetitions of the eccentric component of each movement at 80% of their estimated 1RM by lowering the dumbbell in a controlled manner over the course of 4	Influenza vaccination after the exercise program: Afluria quadrivalent; Seqirus; Lot 02544611A; A/Michigan/45/2015	Antibodies for A/H1N1, A/H3N2, B/Colorado/06/2017 and B/Phuket/3073/2013	<u>A/H1N1 (Geometric means)</u><br>Control:<br>Baseline: 23.58±90.18<br>6-week: 38.17±42.66<br>25-week: 33.30±47.53<br><br>Ex-S:<br>Baseline: 28.44±85.32<br>6-week: 56.88±104.03<br>25-week: 49.03±112.26

		age=75.8±5 years; BMI=29.7±5.7 kg/m^2<br><br>Ex-Op: vaccination in the opposite arm (eccentric exercise of the deltoid and biceps bracchi): n=9; 4 males; age=75.1±5.3 years; BMI=25.8±3.7 kg/m^2	seconds. One set of lateral raise exercise alternated with one set of bicep curl exercise.	(H1N1), A/Singapore/INFIMH-16-0019/2016 (H3N2), B/Colorado/06/2017, and B/Phuket/3073/2013)	Ex-Op:<br>Baseline: 14.22±99.92<br>6-week: 14.59±28.07<br>25-week: 19.83±8.99<br><br><u>A/H3N2 (Geometric means)</u><br>Control:<br>Baseline: 15.97±13.24<br>6-week: 29.13±51.04<br>25-week: 29.62±50.26<br><br>Ex-S:<br>Baseline: 57.27±56.1<br>6-week: 57.18±56.49<br>25-week: 34.69±45.19<br><br>Ex-Op:<br>Baseline: 40.51±39.74<br>6-week: 40.04±56.49<br>25-week: 40.14±72.86<br><br><u>B/Colorado/06/2017 (Geometric means)</u><br>Control:<br>Baseline: 8.28±11.72<br>6-week: 8.41±5.72<br>25-week: 6.27±8<br><br>Ex-S:<br>Baseline: 4.91±2.09<br>6-week: 6.95±3<br>25-week: 4.99±5.09<br><br>Ex-Op:<br>Baseline: 4.91±2.09<br>6-week: 4.95±5<br>25-week: 4.99±5.09

							B/Phuket/3073/2013 (Geometric means)<br>Control:<br>Baseline: 28.18±11.82<br>6-week: 28.29±11.68<br>25-week: 20±36.38<br><br>Ex-S:<br>Baseline: 4.96±15.17<br>6-week: 6.95±2.95<br>25-week: 4.97±9.13<br><br>Ex-Op:<br>Baseline: 5.23±4.7<br>6-week: 13.93±6.17<br>25-week: 9.94±4.03
Felismino 2021	Intervention	CT	Healthy (seropositive to Cytomegalovirus) adults age=60-85 years, women (n=62), men (n=18)<br><br>Groups:<br>Exercise: Practitioners of combined exercise training (CET, n = 49); age=71.7±5.8 years; BMI=25.4±3.7 kg/m^2<br><br>Control: Non-practitioners (NP, n = 31); age=74.1±6.4 years; BMI=28.1±3.9 kg/m^2	Combination of resistance and aerobic exercises, 60–75 min of exercise training per session, three times per week, on alternate days, for 30 days	Before the exercise program. Trivalent vaccine of two influenza A virus types [A/Michigan/45/2015 (H1N1) and A/Switzerland/8060/2017(H3N2)] and one influenza B virus type (B/Colorado/06/2017)	IgG, IgA and IgM titers for influenza vaccine	IgG Exercise Baseline: 0.96±0.44<br>IgG Control Baseline: 0.88±0.41<br>IgG Exercise 30-day: 0.89±0.47<br>IgG Control 30-day: 0.98±0.4<br><br>IgA Exercise Baseline: 0.26±0.14<br>IgA Control Baseline: 0.33±0.25<br>IgA Exercise 30-day: 0.55±0.55<br>IgA Control 30-day: 0.36±0.27<br><br>IgM Exercise Baseline: 0.21±0.12<br>IgM Control Baseline: 0.23±0.14<br>IgM Exercise 30-day: 0.41±0.27<br>IgM Control 30-day: 0.35±0.21
Gualano 2021	No intervention	CSS	Patients with autoimmune rheumatic diseases (ARD-n=898);	Physical activity levels	CoronaVac (Sinovac Life Sciences,	Geometric mean titers of anti-S1/S2 IgG (GMT)	ARD Inactive: 40.07±38.42<br>ARD Active: 52.43±43.28<br>Non-ARD Inactive: 70.14±37.4

			age=52 (41-62) years; BMI=27.5 (24.2-31.2) kg/m² Non-autoimmune rheumatic disease individuals (Non-ARD- n=197); age=47 (35-58.5) years; BMI=26.6 (23.5-30.5) kg/m²		Beijing, China, batch #20200412)		Non-ARD Active: 78.45±36.15
Hayney 2014	Intervention	RCT	Healthy comorbidities not reported<br><br>Groups:<br>Control: n=51; 10 males; age=58.8±6.8 years; BMI=29.8±6.8 kg/m²<br><br>Meditation: n=51, 9 males; age=60.0±6.5 years; BMI=29.0±6.0 kg/m²<br><br>Exercise: n=47; 8 males; age=59.0±6.6 years; BMI=29.0±6.9 kg/m²<br><br>Physical activity<br>Only the SF12 reported which is functional ability not Physical activity	1.Chronic, 8 weeks<br>2. Moderately intensive aerobic exercise using stationary bicycles, treadmills, and other equipment). For most participants, home exercise consisted of brisk walking or jogging.<br>3. weekly 2½ hour group sessions), with home practice (45 minutes per day)<br>4. Frequency not reported for the 2½ hour group sessions. For the home practice please see above (45 min / day)<br>5. Intensity target was 12 to 16 points on the 6 to 20 point scale	1.Trivalent, inactivated influenza vaccine (Sanofi-Pasteur, # U3197AB).<br><br>2. All participants received trivalent inactivated influenza vaccination during week 6 of the interventions. Antibody concentra-tions and cytokine production were determined again at 3 and 12 weeks after immunization	1. A/Brisbane H1N1<br>2. A/Brisbane H3N2<br>3. B/Brisbane	1. A/Brisbane H1N1 (3-week after vaccination_1-week after the end of the 8-week exercise program)<br>Exercise group= 84±33.4<br>Control group= 68.7±26.2<br>2. A/Brisbane H1N1 (12-week after vaccination_10-week after the end of the 8-week exercise program)<br>Exercise group= 62.3±27.9<br>Control group= 61.1±27.4<br>3. A/Brisbane H3N2 (3-week after vaccination_1-week after the end of the 8-week exercise program)<br>Exercise group= 215.6±41.1<br>Control group= 211.1±40<br>4. A/Brisbane H3N2 (12-week after vaccination_10-week after the end of the 8-week exercise program)<br>Exercise group=152.4±38.6<br>Control group=149.3±39.5<br>5. B/Brisbane (3-week after vaccination_1-week after the end of the 8-week exercise program)<br>Exercise group=55.8±28.3<br>Control group=53.5±26.4<br>6. B/Brisbane (12-week after vaccination_10-week after the end of the 8-week exercise program)

							Exercise group=46.6±26.9<br>Control group=47.2±28.5
Kenzaka 2021	No intervention	CSS	Healthy hospital employees (n=2731); age=42.7±12.71 years; males=647	Exercise as routine activity on the day of vaccination	Influenza vaccines	Not reported	Exercise on the day of vaccination do not affect efficacy of influenza vaccine. Exercising increased the risk of developing systemic adverse reactions due to influenza vaccination (self-reported)
Keshtkar-Jahromi 2010	No intervention	CSS	Groups:<br>Coronary artery disease: n=137; age=54.5±9.2 years; males=92; BMI=27.6±4.5 kg/m^2<br>Healthy: n=67; age=52.3±7.3 years; males=39; BMI=25.9±3.6 kg/m^2	Measurements of exercise are not reported	Influenza: Solomon Islands/3/2006 (H1N1), Wisconsin/67/ 2005 (H3N2) Malaysia/2506/ 2004 (B)	1. Solomon Islands/3/2006 (H1N1),<br>2. Wisconsin/67/2005 (H3N2)<br>3. Malaysia/2506/2004 (B)	Malaysia/2506/2004 (B) antibodies were positively associated with regular exercise (minimum of 15 min daily, 4 days a week)
Keylock 2007	No intervention	CSS	1. N=26 (13 low fitness, 5 males; 13 high fitness, 8 males)<br><br>2. Age<br>Low Fitness= 67.9±1.2<br>High Fitness= 64.8±1.2<br><br>3. Sex<br>Low Fitness=5 males<br>High Fitness=8 males<br><br>4. BMI<br>Low Fitness=not reported<br>High Fitness= not reported	VO2max test was assessed in the study. High fitness >50th percentile of VO2max; Low fitness <20th percentile of VO2max	1. Dominant arm influenza vaccine (Aventis Pasteur, Swiftwater, PA)<br>2. In the opposite arm, Tetanus Toxoid (Aventis Pasteur) booster vaccination containing 5 floccuation units of	Influenza vaccine strains:<br>1. New Caledonia/20/99 (H1N1),<br>2. Panama/2007/99 (H3N2),<br>3. B Hong Kong/1434/2002 (H3N2)<br>Tetanus toxoid Anti-TT IgG1 and IgG2 concentrations<br><br>INF-γ pg/ml	New Caledonia/20/99 (H1N1)<br>High fitness 6-week post vaccination: 4.68±0.57<br>High fitness 6-month post vaccination: 3.87±0.49<br>Low fitness 6-week post vaccination: 4.38±0.31<br>Low fitness 6-month post vaccination: 4.07±0.20<br>Panama/2007/99 (H3N2)<br>Low fitness 6-week post vaccination: 6.94±0.34<br>Low fitness 6-month post vaccination: 6.41±0.32<br>High fitness 6-week post vaccination: 7.59±0.49<br>High fitness 6-month post vaccination:7.20±0.50<br>B Hong Kong/1434/2002 (H3N2)

			5. Low Fitness VO2peak= 21.1±1.1 mL/kg/min<br>High Fitness VO2peak= 46.8±3.4 mL/kg/min<br><br>6. Generally healthy, independently living elderly (60–76 years) formed the two groups. Although the paper says that, 11 were on statin, 5 on aspirin and 5 on beta-blockers, which means that there were comorbidities.		Tetanus Toxoid Vaccination was done 1 week after VO2max testing.		Low fitness 6-week post vaccination: 5.789±0.39<br>Low fitness 6-month post vaccination: 5.42±0.33<br>High fitness 6-week post vaccination: 6.93±0.50<br>High fitness 6-month post vaccination: 6.23±0.46<br>Tetanus toxoid IgG1 (optical density)<br>Low fitness 6-week post vaccination: 2.67±0.90<br>Low fitness 6-month post vaccination: 1.66±1.08<br>High fitness 6-week post vaccination: 1.96±1.19<br>High fitness 6-month post vaccination: 1.82±1.23<br>Tetanus toxoid IgG2 (optical density)<br>Low fitness 6-week post vaccination: 1.11±0.86<br>Low fitness 6-month post vaccination: 0.95±0.86<br>High fitness 6-week post vaccination: 1.48±0.79<br>High fitness 6-month post vaccination: 1.39±0.79<br>INFγ Low Fitness (means+SD)<br>6 weeks post-vaccination:21±7.21<br>6 months post-vaccination:25±10.82<br>INFγ High Fitness<br>6 weeks post-vaccination: 22±7.21<br>6 months post-vaccination: 21±10.82
Kohut 2004	Intervention	RCT	1. n=28, 14 in each group (1 stopped from the control group due to cancer diagnosis)	1. Chronic: 10 months<br>2. Type of exercise: Aerobic<br>3. Duration: started with 20 minutes and	1. 2000/2001 trivalent influenza Fluzone®	1. A/New Caledonia/20/99<br>2. A/Panama/2007/99	A/New Caledonia/20/99<br>Exercise group 4-week post vaccination:7.3±3.74<br>Control 4-week post vaccination: 6.08±1.23

	2. Age<br>Intervention 73.07±5.6 years<br>Controls 70.25±5.6 years<br><br>3. Sex<br>Not reported<br><br>4. BMI<br>Intervention 28.4±4.7 kg/m^2<br>Controls 27.3±5.7 kg/m^2<br><br>5. Physical activity levels not reported<br><br>6. Health status not reported; participants did not suffer from autoimmune dysfunction; All subjects had previously been vaccinated with the recommended influenza vaccine for at least the previous 3 years	progressed to 25-30min<br>4. Frequency: 3 times/week<br>5. Intensity: started with 40-60% of HR reserve and progressed to 65-75% HR reserve	vaccine manufactured by Aventis Pasteur, Swiftwater, PA. Study done in USA<br>2. Time of vaccination in relation to intervention: First vaccination in fall 2000. Subjects then started the exercise intervention 4 weeks after receiving the first vaccine. They received the vaccination again in Fall 2001, so the exercise was between the two vaccines, but the author states that they continued to exercise after the 2nd vaccination (i.e. after fall 2001)	3. B/Yamanashi/166/98	Exercise group 3-month post vaccination: 6.95±2.02<br>Control 3-month post vaccination:5.7±1.19<br>A/Panama/2007/99<br>Exercise group 4-week post vaccination:6.73±0.82<br>Control 4-week post vaccination:6.54±1.23<br>Exercise group 3-month post vaccination:6.45±0,94<br>Control 3-month post vaccination:6.54±1.08<br>B/Yamanashi/166/98<br>Exercise group 4-week post vaccination:5.74±1.20<br>Control 4-week post vaccination:5.93±0.87<br>Exercise group 3-month post vaccination:5.32±1.01<br>Control 3-month post vaccination:5.73±1

Kohut 2002	No intervention	CSS	1. n=46<br>Sedentary=15<br>Moderately active=25<br>Active=16<br>2. Age<br>Sedentary=71.5±7.1 years<br>Moderately active=70.7±6.3 years<br>Active=71.9±5.2 years<br>3. Sex<br>Sedentary=6 males<br>Moderately active=8 males<br>Active =7 males<br>4. BMI: Not reported<br>5. Sedentary= did not exercise or participated in aerobic exercise less than once per week;<br>Moderately active= participated in aerobic exercise at a "moderate" intensity one or more times per week over the previous year, but did not meet criteria for inclusion in the active group.<br>Active= participated in aerobic exercise at a "vigorous" intensity 20 minutes or longer three or more times per week for the previous year.	Physical activity levels	1. Trivalent Influenza Type A and B vac- cine (Flushield, Wyeth-Ayerst, Marietta, PA) was adminis- tered to all subjects and contained 15 μg HA of A/Beijing/ 262/95 (H1N1), 15 g HA of A/Sydney/5/97 (H3N2), and 15 μg HA of B/Yamanashi/ 166/98 (B/Beijing 184/93-like). The study was conducted in USA.<br>2. All participants were immunized during the first 2 weeks of October, 1999. For PBMCs blood was collected on day 14	1. Antibody response via IgG, IgM<br>2. INF-γ	<u>IgG Antibodies</u><br>Sedentary 1:25 dilution: 3.13±0.12<br>Moderately active 1:25 dilution:3.05±0.25<br>Active 1:25 dilution:3.31±0.2<br>Sedentary 1:125 dilution: 2.70±0.35<br>Moderately active 1:125 dilution: 2.65±0.3<br>Active 1:125 dilution: 2.87±0.36<br><u>IgM Antibodies</u><br>Sedentary 1:25 dilution: 1.24±0.74<br>Moderately active 1:25 dilution: 1.16±0.7<br>Active 1:25 dilution: 1.71±0.6<br>Sedentary 1:125 dilution: 0.62±0.54<br>Moderately active 1:125 dilution:0.40±0.35<br>Active 1:125 dilution:0.78±0.44<br><u>INF-γ</u><br>Sedentary: 1026.28±364.02<br>Moderately active: 865.57±338.8<br>Active: 849.12±341

			6. Healthy, Individuals suffering from untreated chronic disease, autoimmune disease, cancer, or any other disease known to alter immunity were excluded.		postimmunization.		
Kohut 2005	Intervention	RCT	Generally healthy (n=27); Exercise group: age (years)= 73.07±5.59, 7 males, 7 females, BMI: 28.5±4.8 kg/m^2; Control group: age (years)=70.25±5.57, 6 males, 7 females, BMI: 27.5±6 kg/m^2	1. Chronic supervised cycling 3 times/week, 20 min/session, 40-60% of heart rate reserve gradually progressed at 65-75%. 10 months before vaccination to 12 weeks post vaccination<br><br>2. Control group current level of physical activity (i.e. walking, no structured exercise)	1. Influenza H1N1 after a 10-month exercise program<br><br>2. Influenza H3N2 after a 10-month exercise program	1. A/New Caledonia/20/99, H1N1<br>2. A/Panama/2007/99, H3N2	1. No mean differences for A/New Caledonia/20/99, H1N1,<br>10-month after exercise (vaccination time point): Exercise group (mean±SD)=6.4±1.76, Control group=5.9±1.01,<br>4-wk post vaccination: Exercise group (mean±SD)=7.3±1.91, Control group=6±1.15,<br>12-wk post vaccination: Exercise group (mean±SD)=7±2.02, Control group=5.8±1.19.<br>2. No mean differences for A/Panama/2007/99, H3N2, 10-month after exercise (vaccination time point): Exercise group (mean±SD)=5.7±1.12, Control group= 6.2±1.15,<br>4-wk post vaccination: Exercise group (mean±SD)=6.7±1.91, Control group=6.5±0.82,<br>12-wk post vaccination: Exercise group (mean±SD)=6.5±0.94, Control group= 6.5±1.15.<br>3. INF-γ for H1N1: Exercise group: 0.59±3,78 Control group:-1.01±2.02<br>4. INF-γ for H3N2: Exercise group: 0.63±3.33 Control group:-0.60±2.67
Ledo 2020	No Intervention	CSS	Athletes (n=45); females (n=9); age=23.2±7.7 years	1. Athletes performed training at least 5 days/week, between	Influenza vaccine (Influsplit Tetra 2016/17)	Antibodies for the following strains:	H1N1<br>Athletes: 5.50±1.60<br>Non-athletes: 4.63±1.19<br>H3N2

			Controls (n=25); females (n=8); age=22.8±4.1 years	1.5-4 hours per session<br><br>2. Controls: No professional sport and leisure sport in excess of 2 training/week for the last year and during the study	After the last training	1. A/California/7/2009 (H1N1)<br>2. A/Hong Kong/4801/2014 (H3N2)<br>3. B/Brisbane/60/2008<br>4. B/Phuket/3073/2013	Athletes: 5.30±1.62<br>Non-athletes: 4.31±1.55<br>Brisbane<br>Athletes: 4.82±1.50<br>Non-athletes: 4.47±1.30<br>Phuket<br>Athletes: 4.92±1.57<br>Non-athletes: 4.50±1.93
Long 2013	Intervention	RCT	1. Controls n=45<br>Exercise n=44<br>NOTE: There was a 6 month follow up in this study and there was a significant withdrawal of participants 6 months post vaccination (13 from the intervention group and 25 from the control group.<br><br>2. Age<br>Controls=46.87±7.38 years<br>Exercise= 46.84±6.56 years<br><br>3. Sex<br>female only<br><br>4. BMI<br>Controls=27.30±4.32 kg/m^2	1. Chronic. The intervention was 16 weeks but there was a 6 month follow up.<br>2. Type of exercise: physical activity consultation by a trained practitioner, were given a pedometer to use for the study duration, and received weekly prompts by telephone, e-mail, or text throughout the 16-week intervention<br><br>3. Duration: 16 weeks<br>4. Frequency: not reported<br>5. Intensity: not reported	1. Pn vaccination (Pneumovax II, Batch no. NK527801616X; Sanofi Pasteur MSD, UK). The study was done in the UK<br>2. the intervention was 16 weeks; the vaccination was done 12 weeks after the start of the intervention.	Antibody response for pneumococcal IgG; 11 strains antibody titers	<u>IgG titers overall</u><br>Exercise 4-week post vaccination: -0.04±0.62<br>Control 4-week post vaccination: 0.19±0.63<br>Exercise 6-month post vaccination: -0.14±0.76<br>Control 6-month post vaccination: 0.14±0.79<br><u>Pn1</u><br>Exercise 4-week post vaccination (n=38): 4±8.18<br>Control 4-week post vaccination (n=33): 7.07±16.31<br>Exercise 6-month post vaccination (n=26): 3.55±6.20<br>Control 6-month post vaccination (n=8): 1.49±2.62<br><u>Pn3</u><br>Exercise 4-week post vaccination (n=38): 21.86±100.89<br>Control 4-week post vaccination (n=33): 279.26±1338.19<br>Exercise 6-month post vaccination (n=26): 36.01±177.46<br>Control 6-month post vaccination (n=8): 37±61.99<br><u>Pn5</u>

	Exercise=27.70±5.29 kg/m^2<br><br>5.<br>Controls=insufficiently active<br>Exercise= insufficiently active<br><br>6. Health status<br>Controls n=6 on medication<br>Exercise n=10 on medication<br>Eligibility criteria excluded diagnosed non-communicable diseases		Exercise 4-week post vaccination (n=38): 7.88±15.31<br>Control 4-week post vaccination (n=33): 7.44±14.87<br>Exercise 6-month post vaccination (n=26): 8.96±29.45<br>Control 6-month post vaccination (n=8): 1.55±2.46<br>Pn6B<br>Exercise 4-week post vaccination (n=38): 4.33±12.82<br>Control 4-week post vaccination (n=33): 107.09±508.62<br>Exercise 6-month post vaccination (n=26): 3.04±6.66<br>Control 6-month post vaccination (n=8): 3.77±9.52<br>Pn7F<br>Exercise 4-week post vaccination (n=38): 5.19±9.05<br>Control 4-week post vaccination (n=33): 10.52±19.19<br>Exercise 6-month post vaccination (n=26): 4.11±6.34<br>Control 6-month post vaccination (n=8): 6.99±11.22<br>Pn9V<br>Exercise 4-week post vaccination (n=38): 1.50±2.29<br>Control 4-week post vaccination (n=33): 6.85±11.94<br>Exercise 6-month post vaccination (n=26): 1.09±1.88<br>Control 6-month post vaccination (n=8): 1.07±1.27<br>Pn14

Exercise 4-week post vaccination (n=38):
3.16±4.06
Control 4-week post vaccination (n=33):
6.18±8.81
Exercise 6-month post vaccination (n=26):
14.78±56.43
Control 6-month post vaccination (n=8):
5.46±6.26
Pn18C
Exercise 4-week post vaccination (n=38):
7.73±11.61
Control 4-week post vaccination (n=33):
16.39±24.26
Exercise 6-month post vaccination (n=26):
7.39±10.57
Control 6-month post vaccination (n=8):
11.79±14.07
Pn19A
Exercise 4-week post vaccination (n=38):
4.81±13.41
Control 4-week post vaccination (n=33):
4±4.80
Exercise 6-month post vaccination (n=26):
6.25±18.91
Control 6-month post vaccination (n=8):
4.36±4.72
Pn19F
Exercise 4-week post vaccination (n=38):
1.33±2.59
Control 4-week post vaccination (n=33):
29.82±153.82
Exercise 6-month post vaccination (n=26):
1.27±2.28
Control 6-month post vaccination (n=8):
1.47±2.54
Pn23F

							Exercise 4-week post vaccination (n=38): 63.82±374.82<br>Control 4-week post vaccination (n=33): 8.73±16.30<br>Exercise 6-month post vaccination (n=26): 3.38±9.72<br>Control 6-month post vaccination (n=8): 3.84±5.68
Long 2012	Intervention	RCT	1. n=122<br>-2 age groups randomized: 18-30 and 50-64 years old<br>Exercise n=61<br>Controls n=61<br>2. Age<br>18-30 year group:<br>Exercise=21.37±2.82 years<br>Controls=21.13±2.19 years<br>50-64 year group:<br>Exercise=59.94±4.4 years<br>Controls=58.55±4.38 years<br>3. Sex:<br>An equal number of males/females was randomized into groups, but there is no information of the exact numbers<br>4. BMI:<br>18-30 year group:<br>Exercise=25.12±5.11 kg/m^2	1. Acute<br>2. Type of exercise: brisk walking<br>3. Duration: 45 min<br>4. Frequency: NA<br>5. Intensity: 55% of HRmax	1. A full dose pneumonia vaccine (Pneumovax II, Sanofi Pasteur, batch number NL30250)<br>2. A half dose influenza vaccination (Fluarix, GSK, batch number AFLUA538AA)<br>The study was conducted in UK.<br><br>3. Time of vaccination in relation to intervention: vaccination received after the 45 min Brisk walking	1. Antibody response for pneumococcal IgG, IgM<br><br>2. Anti-influenza strains:<br>A/California/7/ 2009<br>B/Brisbane/60/2008<br>A/Perth/16/2009	Pneumococcal<br>IgG exercise young 4-week post vaccination: 0.56±0.22<br>IgG control young 4-week post vaccination: 0.46±0.33<br>IgG exercise old 4-week post vaccination: 0.33±0.27<br>IgG control old 4-week post vaccination: 0.22±0.38<br>IgM exercise young 4-week post vaccination: 0.31±0.38<br>IgM control young 4-week post vaccination: 0.41±0.27<br>IgM exercise old 4-week post vaccination: -0.23±0.38<br>IgM control old 4-week post vaccination: -0.19±0.3<br>Influenza A/California/7/ 2009<br>Exercise young 4-week post vaccination: 2.67±0.38<br>Control young 4-week post vaccination: 2.74±0.55<br>Exercise old 4-week post vaccination: 2.05±0.88<br>Control old 4-week post vaccination: 2.10±0.77<br>Influenza B/Brisbane/60/2008<br>Exercise young 4-week post vaccination: 1.99±0.49

			Controls=23.82±3.35 kg/m^2<br>50-64 year group:<br>Exercise=25.74±3.03<br>Controls=26.21±5.21<br>5. Participants with high endurance training<br>6. Health status:<br>No history of cancer, inflammatory disease or cardiovascular disease; no chronic obstructive pulmonary disorder, diabetes mellitus, asthma, congestive heart failure, Guillain Barre syndrome or psychiatric disorder				Control young 4-week post vaccination: 2.23±0.71<br>Exercise old 4-week post vaccination: 1.41±0.88<br>Control old 4-week post vaccination: 1.41±0.93<br><u>Influenza A/Perth/16/2009</u><br>Exercise young 4-week post vaccination: 2.36±0.33<br>Control young 4-week post vaccination: 2.60±0.38<br>Exercise old 4-week post vaccination: 2.22±0.49<br>Control old 4-week post vaccination: 2.22±0.60
Mitsunaga 2021	No intervention	CSS	Healthy hospital workers (n=374; male=110)<br>Age=median 36 (16 interquartile range) years	Exercise hours/day	BNT162b2 vaccine (COMIRNATY Tozinameran)	Anti-SARS-CoV-2 antibody titer	Exercisers with <30 minutes of exercise/week were more susceptible to low-antibody titer than exercisers with >30 minutes of exercise/week (Odds ratio=0.86, confidence interval 0.51-1.44). Lack of outdoor exercise was a suppressor of antibody responses
Monteiro 2020	Intervention	RCT	Healthy elderly (n=84; males=19)<br><br><u>Combined exercise group (CET; n=53)</u>;<br>Placebo sub-group (n=27): age=72.2±5.9 years; BMI=25.4±3.6 kg/m^2	Aerobic and resistance 50–75% of maximal cardiac frequency or strength repetition, 60-75 minutes per session, 3 times per week, on alternate days, for 30 days	Before the exercise program: Influenza trivalent vaccine was composed of two types of influenza A virus	IgM, IgG, IgA	<u>IgM</u><br>CET placebo baseline: 0.2±0.11<br>CET placebo 30 days: 0.32±0.18<br>CET l-Glutamine baseline: 0.18±0.1<br>CET l-Glutamine 30 days: 0.39±0.25<br>NP-control placebo baseline: 0.2±0.06<br>NP-control placebo 30 days: 0.3±0.19<br>NP-control l-Glutamine baseline: 0.22±0.11<br>NP-control l-Glutamine 30 days: 0.39±0.25<br><u>IgG</u>

			l-Glutamine sub-group (n=26): age=71.2±5.9 years; BMI=25.4±3.9 kg/m²<br><br><u>Control non-practitioners group (NP; n=31)</u>; Placebo sub-group (n=17): age=75.1±7.1 years; BMI=27.7±4 kg/m²<br>l-Glutamine sub-group (n=14): age=72.9±5.4 years; BMI=28.5±3.7 kg/m²		(A/Michigan/45/2015 (H1N1), and A/Switzerland/8060/2017(H3N2)) and one type of influenza B virus (B/Colorado/06/2017)		CET placebo baseline: 1.02±0.34<br>CET placebo 30 days: 0.96±0.39<br>CET l-Glutamine baseline: 0.85±0.49<br>CET l-Glutamine 30 days: 0.88±0.47<br>NP-control placebo baseline: 0.91±0.44<br>NP-control placebo 30 days: 1.07±0.33<br>NP-control l-Glutamine baseline: 0.96±0.3<br>NP-control l-Glutamine 30 days: 0.85±0.33<br><u>IgA</u><br>CET placebo baseline: 0.29±0.17<br>CET placebo 30 days: 0.32±0.14<br>CET l-Glutamine baseline: 0.28±0.16<br>CET l-Glutamine 30 days: 0.57±0.39<br>NP-control placebo baseline: 0.32±0.24<br>NP-control placebo 30 days: 0.39±0.26<br>NP-control l-Glutamine baseline: 0.38±0.27<br>NP-control l-Glutamine 30 days: 0.39±0.27
Ranadive 2014	Intervention	RCT	Exercise group (n=28): Age=66±4.92 years; BMI=28.07±4.39 kg/m²; VO2peak=25.90±6.35 mL/kg/min<br><br>Control group (n=27): Age=67±4 years; BMI=25.94±5.09 kg/m²; VO2peak=25.14±6.70 mL/kg/min<br><br>Healthy: Free from cardiovascular and respiratory disease, non-smokers	Acute<br>Moderate intensity aerobic session at 55-65% of maximum heart rate	Influenza vaccination 2010-2011 after the exercise session	Antibodies for<br>H1N1<br>H3N2<br>B-Brisbane<br><br>Seroprotection percentage	<u>H1N1</u><br>Exercise group baseline 24-48 hours post vaccination: 1.16±2.06<br>Control group baseline 24-48 hours post vaccination: 2.42±2.03<br>Exercise group 4-week post vaccination: 3.16±2.17<br>Control group 4-week post vaccination: 3.97±1.92<br><u>H3N2</u><br>Exercise group baseline 24-48 hours post vaccination: 3.36±2.06<br>Control group baseline 24-48 hours post vaccination: 3.55±1.97<br>Exercise group 4-week post vaccination: 5.40±2.17<br>Control group 4-week post vaccination: 5.55±2.03<br><u>B-Brisbane</u>

							Exercise group baseline 24-48 hours post vaccination: 0.97±2.06<br>Control group baseline 24-48 hours post vaccination: 1.09±0.31<br>Exercise group 4-week post vaccination: 2.54±2.17<br>Control group 4-week post vaccination: 2.92±2.03<br>Sero-protection<br>H1N1 exercise=44 %<br>H1N1 control=56 %<br>H3N2 exercise=47.37 %<br>H3N2 control=52.63 %<br>B-Brisbane exercise=25 %<br>B-Brisbane control=75 %
Schuler 2003	No intervention	CSS	n=30<br>Age: 81±5 years<br>Sex: 10 males, 20 females<br>BMI: 24 ± 4 kg/m^2<br>Healthy with some medication for this age; mean number of medications: 2.7±1.9	Chronic (assessed via the Physical Activity Scale for the Elderly – "PASE"-Questionnaire, which generates a score for physical activity).	1998-99 influenza virus vaccine<br><br>One scheduled influenza vaccination for all participants	Reference antigens:<br>H1N1 (A/Beijing/262/95) &<br>H3N2 (A/Sindey/05/97)	Significant association between H3N2 and levels of physical activity ONLY at week 1 post-vaccination (r=0.593, p=0.007, n=19).<br>No significant associations between H3N2-specific antibody & exercise on weeks 2, 4 & 6.<br>No significant associations between H1N1-specific antibody & exercise on weeks 1, 2, 4 & 6.
Schuler 1999	No intervention	CSS	N: 67 (males=24; females=43)<br>Age (males)=22±2.2 years<br>Age (females)=21±2.2 years<br>Weight (males)=84.5±16.1 kg<br>Height (males)= 180.1±6.1 cm<br>Weight (females)= 61.6±8.6 kg	Physical activity levels assessed via the Stanford 7-day Physical Activity Recall Questionnaire<br><br>Group 1 (lower active): <8823 kJ.60kg^{-1}.day^{-1}	1996 influenza virus vaccine	Reference antigens:<br>H1 (A/Texas/36/91) &<br>H3 (A/Johannesburg/ 33/94)	No significant associations between various levels of physical activity and physical fitness and the immune response in college males and females.<br>H1 fitness levels<br>Lower fit 1-week post vaccination: 46.1±44.8 (n=31)<br>Lower fit 2-week post vaccination: 36.9±28.1 (n=31)<br>Lower fit 4-week post vaccination: 58.4±51.9 (n=31)

	Height (females)= 163.6±8.6 cm<br><br>Apparently healthy university students, USA	Group 2 (moderate active): >8823 & <10924 kJ.60kg^{-1}.day^{-1}<br><br>Group 3 (higher active): >10924 kJ.60kg^{-1}.day^{-1}<br><br><u>Fitness levels</u> assessed via estimation of maximal oxygen consumption<br><br>Group 1 (lower fit): <36 ml.kg-1.min-1<br><br>Group 2 (moderate fit): >36 & <44 ml.kg-1.min-1<br><br>Group 3 (higher fit): >44 ml.kg-1.min-1		Lower fit 6-week post vaccination: 26.9±27.8 (n=31)<br>Moderate fit 1-week post vaccination: 28.9±29.6 (n=23)<br>Moderate fit 2-week post vaccination: 32.8±30.8 (n=23)<br>Moderate fit 4-week post vaccination: 38.5±32.6 (n=23)<br>Moderate fit 6-week post vaccination: 21±24.3 (n=23)<br>Higher fit 1-week post vaccination: 29.6±32.8 (n=13)<br>Higher fit 2-week post vaccination: 25.6±20.4 (n=13)<br>Higher fit 4-week post vaccination: 22.4±18.5 (n=13)<br>Higher fit 6-week post vaccination: 16±13.8 (n=13)<br><u>H3 fitness levels</u><br>Lower fit 1-week post vaccination: 21.5±44.3 (n=31)<br>Lower fit 2-week post vaccination: 44.6±41.7 (n=31)<br>Lower fit 4-week post vaccination: 35.8±39.7 (n=31)<br>Lower fit 6-week post vaccination: 43±42.1 (n=31)<br>Moderate fit 1-week post vaccination: 31±38.3 (n=23)<br>Moderate fit 2-week post vaccination: 69.5±93.2 (n=23)<br>Moderate fit 4-week post vaccination: 40.3±63.3 (n=23)<br>Moderate fit 6-week post vaccination: 88.6±99.7 (n=23)<br>Higher fit 1-week post vaccination: 29.2±70 (n=13)

	Higher fit 2-week post vaccination: 41.6±52.8 (n=13)<br>Higher fit 4-week post vaccination: 52.8±71.9 (n=13)<br>Higher fit 6-week post vaccination: 52.8±61.8 (n=13)<br><u>H1 physical activity levels</u><br>Lower fit 1-week post vaccination: 27.3±24.8 (n=13)<br>Lower fit 2-week post vaccination: 40±41.4 (n=13)<br>Lower fit 4-week post vaccination: 53.3±43.3 (n=13)<br>Lower fit 6-week post vaccination: 24±29.6 (n=13)<br>Moderate fit 1-week post vaccination: 32.8±39.6 (n=31)<br>Moderate fit 2-week post vaccination: 27.1±18.4 (n=31)<br>Moderate fit 4-week post vaccination: 32.3±30.1 (n=31)<br>Moderate fit 6-week post vaccination: 20±21.9 (n=31)<br>Higher fit 1-week post vaccination: 34±44.7 (n=23)<br>Higher fit 2-week post vaccination: 30.7±25.5 (n=23)<br>Higher fit 4-week post vaccination: 31±36.3 (n=23)<br>Higher fit 6-week post vaccination: 16.9±19.1 (n=23)<br><u>H3 physical activity levels</u><br>Lower fit 1-week post vaccination: 16.9±15.4 (n=13)<br>Lower fit 2-week post vaccination: 56.9±53.4 (n=13)

							Lower fit 4-week post vaccination: 66±87.2 (n=13)<br>Lower fit 6-week post vaccination: 63.8±59.8 (n=13)<br>Moderate fit 1-week post vaccination: 14.7±39.9 (n=31)<br>Moderate fit 2-week post vaccination: 40±49.9 (n=31)<br>Moderate fit 4-week post vaccination: 38.8±64.6 (n=31)<br>Moderate fit 6-week post vaccination: 67±92.9 (n=31)<br>Higher fit 1-week post vaccination: 53.9±94.2 (n=23)<br>Higher fit 2-week post vaccination: 73.6±58.9 (n=23)<br>Higher fit 4-week post vaccination: 39.6±35.3 (n=23)<br>Higher fit 6-week post vaccination: 66±70.6 (n=23)
Segerstrom 2012	No intervention	CSS	n=134 (78 females; 56 males)<br>Age: 74 (range=60-91)<br>BMI not reported<br>"Generally healthy" – analyses were corrected for beta-blockers and inflammatory and activation markers	Physical activity levels measures via the Leisure Time Exercise Questionnaire	Influenza	H1N1, H3N2 and B<br><br>- Longitudinal over up to 8 years (2000-2008)	Mean on state physical activity (low physical activity ≈500 METs-minutes lower than average; high physical activity ≈500 METs-minutes higher than average). Significant simple main effect of distress (antibody response) physical activity was higher than average (γ = -3.56, standard error = 1.48, $t(172) = -2.41$, $p < 0.02$)
Stewart 2018	Non-intervention	CSS	1. Baseline measurements (n=76); Follow-up measurements (n=45)<br>2. Age: 24.75±4.91 years	1. Physical activity levels assessed via the Godin-Shepard Leisure Time Physical Activity Questionnaire.	Influenza<br>H1N1<br>H3N2<br><br>Antibodies examined pre-vaccination	The vaccine contained:<br><br>1. A/California/7/2009 (H1N1) pdm09-like virus,	No relationship between physical activity levels and antibody detection.

			3. Sex: male<br>4. BMI: 25.55 ± 4.22 kg/m²<br>5. Seemingly healthy participants	2. Physical activity score: 57.5±34.07	and 4 weeks post vaccination	2. A/Texas/50/2012 (H3N2)-like virus<br>3. B/Massachusetts/2/2012-like virus	
Turner 2021	No intervention	CSS	Elite athletes (n=120; females=54; age=24±4 years)<br>Susceptible acute respiratory tract infection athletes (RTI-HS, n=22)<br>Non-susceptible acute respiratory tract infection athletes (RTI-NS, n=23)<br>Asthmatic athletes (n=33),<br>Healthy non-athletes (n=10; females=7; age=27±3 years)	Comparison between athletes and non-athletes	Pneumococcus	Anti-pneumococcus IgG titetrs (mg/L)	Non-parametric data<br>Healthy non-athletes controls: 82.62±58.02 median=47.73 (17.06-217.07)<br>RTI-HS: 73.82±56.17 median=40 (10.4-204.89)<br>RTI-NS: 92.60±68.47 median=53.6 (13.06-250.17)<br>Asthmatic athletes: 50.4±54.31 median=50.4 (12.53-229.77)
Whitham & Blannin 2003	Intervention	CT	1. n=21<br>2. Age=22.5±3.5 years<br>3. Sex=male<br>4. Healthy<br>5. VO2max heavy training group=53.1±3.7 mL/kg/min; VO2max light training group=41.1±1.5 mL/kg/min	Two groups:<br>1. Heavy training group encouraged to increase their exercise training for 3 weeks with no specific training regimens<br><br>2. Light training group maintained their habitual physical activity and acted as controls	The 2001 season Influvac Influenza vaccine (Solvay Pharmaceuticals BV) was used.<br><br>Participants were vaccinated at the start of	IgG for:<br>1. A/Panama/2007/99 (H3N2),<br>2. A/New Caledonia/20/99 (H1N1),<br>3. B/Yamanashi/166/98	1. Three weeks of intensified training had no effect on the kinetics of the IgG response to influenza vaccination, when compared with very light exercise.<br>2. Inverse correlations between exercise training intensity and fold antibody increases were found<br>3. Training loads had no effect on overall IgG 14 days post vaccination<br>Heavy training (n=7):<br>IgG overall 14-day post vaccination_1-week post exercise: 4.12±0.08

				3. Physical activity assessed by activity duration and heart rate (over 60% HRmax) monitoring	week 3 of exercise training		IgG overall 12-month post vaccination_11-month post exercise: 4.06±0.03<br>Light training (n=3):<br>IgG overall 14-day post vaccination_1-week post exercise: 3.96±0.1<br>IgG overall 12-month post vaccination_11-month post exercise: 3.59±0.02
Wong 2019	No intervention	CSS	Healthy females (n=56)<br>Subgroups<br>1. Sedentary (n=28)<br>Age=75.4±5.1 years<br>BMI=23.1±2.3 kg/m^2<br>2. Active (n=28)<br>Age=70.2±3.9 years<br>BMI=22.9±3.5 kg/m^2	Subgroups<br>1. Sedentary (n=28)<br><10927 steps/day<br>2. Active (n=28)<br>>18509 steps/day<br><br>Activity levels were monitored via a wrist-worn for a 14-day period that commenced immediately after vaccination.	Two 2013-14 seasonal influenza vaccines were used.<br><br>The strains of the 1st vaccine were: A/California/07/2009 (H1N1), A/Texas/50/2012 (H3N2) and B/Massachusetts/02/2012.<br><br>The 2nd vaccination at the 20-month follow-up, included -- A/California/07/2009 (H1N1), A/Switzerland/97/2013 (H3N2) B/Phuket/3073/2013.	1st vaccine: A/California/07/2009 (H1N1), A/Texas/50/2012 (H3N2) and B/Massachusetts/02/2012.<br><br>2nd vaccine at the 20-month follow-up, included -- A/California/07/2009 (H1N1), A/Switzerland/97/2013 (H3N2) B/Phuket/3073/2013.	1. In physically active elderly females, improvements in both arms of the immune response. In addition to a greater post vaccination expansion of monocytes, it was also observed an upregulation of genes related to phagocytic function of monocytes and macrophages.<br>2. A stronger post-vaccination plasmablast and vaccine-specific B-cell response in active elderly females as compared to sedentary.<br>3.In active elderly females, the magnitude of the post-vaccination monocyte (D2) and CD4 T-cell (D28) was found to correlate positively to H1N1 titres.<br>A/California/07/2009 (H1N1) vaccine 1<br>Active 28-day post vaccination (n=28): 820.55±265.40<br>Sedentary 28-day post vaccination (n=28): 802.33±247.65<br>A/California/07/2009 (H1N1) vaccine 2<br>Active 28-day post vaccination (n=14): 971.72±195.46<br>Sedentary 28-day post vaccination (n=11): 876.81±145.46<br>A/Texas/50/2012 (H3N2) vaccine 1<br>Active 28-day post vaccination (n=26): 7246.25±1513.07<br>Sedentary 28-day post vaccination (n=28): 7316.29±1933.84<br>A/Switzerland/97/2013 (H3N2) vaccine 2

							Active 28-day post vaccination (n=14): 8347.79±1049.23<br>Sedentary 28-day post vaccination (n=11): 8280.57±1095.88<br>B/Massachusetts/02/2012 vaccine 1<br>Active 28-day post vaccination (n=28): 7369.29±1346.69<br>Sedentary 28-day post vaccination (n=28): 6972.62±1665.12<br>B/Phuket/3073/2013 vaccine 2<br>Active 28-day post vaccination (n=14): 7988.08±1180.39<br>Sedentary 28-day post vaccination (n=11): 6826.82±1048.36
Woods 2009	Intervention	RCT (treated as CSS due to the 4 month exercise period preceding the vaccination)	Healthy (n=144)<br><br>Flexibility/balance exercise Group (n=70, 43 females)<br>Age=70.1±5.7 years<br>BMI=28.2±0.5 kg/m^2<br><br>Cardiovascular exercise Group (n=74, 47 females)<br>Age=69.6±4.9 years<br>BMI=27.4±0.6 kg/m^2<br><br>Inclusion criteria ability to participate in an exercise program, medical clearance from personal physician, nonsmoker (>10 years)	1. Flexibility/balance exercise Group (supervised sessions) 2 days/week for 75 minutes stretching and balance exercises using low-level resistance exercises; Intensity: <20% VO2peak; Mode: Thera-bands, foam rollers, balance balls<br>2. Cardiovascular exercise Group (supervised sessions) 45-55% of VO2max progressed to 60-70% VO2max within 3 months, 3 days/week, for 10-15 minutes increased to 56-60 minutes in 4th month); Mode: Walking,	In October of each year (2003–2005), 4 months after the start of the intervention, participants vaccinated with the trivalent Fluzone (Aventis Pasteur, PA) influenza vaccine.<br><br>Blood samples before vaccination and at 3, 6, and 24 weeks after	1. H1N1 (New Caledonia/20/99)<br><br>2. H3N2 Variants (Panama, Fujian, New York)<br><br>3. B Variants (Hong Kong, Shanghai)	Cardiovascular exercise training resulted in a longer-lasting (24 weeks) seroprotective response (HI>40) to influenza vaccination (H1N1 & H3N2 but not B variants), than flexibility/balance exercise.<br>Parametric data<br>Flexibility group<br>H1N1 baseline: 3.75±0.26<br>H1N1 3 weeks: 5.69±0.19<br>H1N1 6 weeks: 5.61±0.19<br>H1N1 24 weeks: 4.70±0.18<br>H3N2 baseline: 4.47±0.31<br>H3N2 3 weeks: 6.24±0.2<br>H3N2 6 weeks: 6.22±0.27<br>H3N2 24 weeks: 5.57±0.31<br>B baseline: 4.97±0.28<br>B 3 weeks: 6.69±0.23<br>B 6 weeks: 6.57±0.22<br>B 24 weeks: 5.78±0.23<br>Exercise group<br>H1N1 baseline: 3.90±0.25<br>H1N1 3 weeks: 5.72±0.19<br>H1N1 6 weeks: 5.64±0.20

				cycling, elliptical, stair climbing<br>3. Duration: 10 months for both groups	vaccination.		H1N1 24 weeks: 5.02±0.21<br>H3N2 baseline: 4.58±0.25<br>H3N2 3 weeks: 6.55±0.19<br>H3N2 6 weeks: 6.40±0.19<br>H3N2 24 weeks: 6.05±0.19<br>B baseline: 5.06±0.25<br>B 3 weeks: 6.59±0.24<br>B 6 weeks: 6.53±0.23<br>B 24 weeks: 5.97±0.31
Gleeson 1996	No intervention	CSS	1. Swimming elite athletes (n=20); 10 males, 10 females) elite swimmers aged 17-23 years<br><br>2. Untrained university students (n=19); 8 males, 11 females aged years	Mean 2-day training volumes (km) for elite swimmers 20 days before and 14 days after vaccination	Pneumovax 23 (pneumococcal vaccine)<br><br>Blood samples just before vaccination (day 0) and 14 days after vaccination	Strains:<br>4, 6B, 8, 14, 18C and 23F (Danish Nomenclature System)<br><br>IgA, IgG, IgM and IgG subclasses were measured	<u>Strain 4:</u><br>Swimmers 14-day_post vaccination: 3788±4342<br>Controls 14-day_post vaccination: 5114±7985.5<br><u>Strain 6B:</u><br>Swimmers 14-day_post vaccination: 2837±3108<br>Controls 14-day_post vaccination: 2600±3363.5<br><u>Strain 8:</u><br>Swimmers 14-day_post vaccination: 9202±4292.5<br>Controls 14-day_post vaccination: 8676±4518<br><u>Strain 14:</u><br>Swimmers 14-day_post vaccination: 5757±6531<br>Controls 14-day_post vaccination: 6827±20745<br><u>Strain 18C:</u><br>Swimmers 14-day_post vaccination: 33412±45304.5<br>Controls 14-day_post vaccination: 30306±61761<br><u>Strain 23F:</u><br>Swimmers 14-day_post vaccination: 7759±12805.5

Controls 14-day_post vaccination: 5816±16048

Strain 4:

Post vaccination responses swimmers males (n=10): 100%

Post vaccination responses swimmers females (n=10): 100%

Post vaccination responses controls males (n=8): 100%

Post vaccination responses controls females (n=11): 91%

Strain 6B:

Post vaccination responses swimmers males (n=10): 80%

Post vaccination responses swimmers females (n=10): 70%

Post vaccination responses controls males (n=8): 75%

Post vaccination responses controls females (n=11): 64%

Strain 8:

Post vaccination responses swimmers males (n=10): 60%

Post vaccination responses swimmers females (n=10): 40%

Post vaccination responses controls males (n=8): 38%

Post vaccination responses controls females (n=11): 45%

Strain 14:

Post vaccination responses swimmers males (n=10): 70%

Post vaccination responses swimmers females (n=10): 100%

Post vaccination responses controls males (n=8): 75%

							Post vaccination responses controls females (n=11): 73<br>Strain 18C:<br>Post vaccination responses swimmers males (n=10): 70%<br>Post vaccination responses swimmers females (n=10): 80%<br>Post vaccination responses controls males (n=8): 63%<br>Post vaccination responses controls females (n=11): 82%<br>Intensive training schedules had not compromised the swimmers' ability to mount an effective antibody response against the 6 antigens.
Yang 2007	Intervention	CT	Exercise Group (n=27; 6 males, 21 females)<br>Age=79.5±1.9 years<br>BMI=24.1±1.0 kg/m²<br><br>Control Group (n=23; 7 males, 16 females)<br>Age=74.5±1.6 years<br>BMI=26.2±0.57 kg/m²	Chronic<br>Three of 1-hour classes/week for 20 weeks.<br>Each 1-hour class consisted of equal parts of Chinese Qigong and Taiji form practice.<br>By week 13 participants were performing one 10-minute sitting and two 10-minute standing meditations in each hour class.	2003-2004 influenza vaccine<br><br>Blood samples 3,6 and 20 weeks after vaccination during the exercise program– the last time point corresponds to the end of intervention	Strains<br>H1N1 (New Caledonia/20/99)<br><br>H3N2 (Panama/2007/99)<br><br>B (Hong Kong/1434/2002)	Antibodies<br>Exercise 3-week_post vaccination: 2.77±4.81<br>Control 3-week_post vaccination: 1.57±1.42<br>Exercise 6-week_post vaccination: 2.32±3.23<br>Control 6-week_pOveost vaccination: 1.53±2.03<br>Exercise 20-week_post vaccination: 2.10±4.17<br>Control 20-week_post vaccination: 1.10±0.79<br>Percentage of responses<br>H1N1 exercise: 37%<br>H1N1 control: 20%<br>H3N2 exercise: 56%<br>H3N2 control: 45%<br>B exercise: 41%<br>B control: 40%

Key: Values reported as means and standard deviations; CSS=cross-sectional study; CT=control trial; BMI=body mass index; VO2max= maximum oxygen uptake; 1RM= 1 repetition maximum; RCT=randomized controlled trial; PASE=physical activity scale for elderly; MET=metabolic equivalent; IPAQ=international physical activity questionnaire; W=watt; IFN-γ=interferon gamma; rpm=revolutions per minute; HR=heart rate; VO2peak= peak oxygen uptake.

Risk of bias assessment

Regarding the eligible randomized controlled trials (RCT), three [1-3] showed low, five [4-8] showed high and nine [9-17] showed some concerns risk of bias in randomization process. In intervention assignment, three RCT [3,5,17] displayed some concerns, five [4,9-11,14] high and the remaining nine low risk of bias. In intervention adherence, two RCT [6,16] showed low risk, four [9-11,14] high risk and 11 [1-5,7,8,12,13,15,17] some concerns. In missing data, four RCT [9-11,14] displayed high risk and the remaining 13 low risk of bias. Finally, in outcome and reported results, one RCT [8] showed high risk and the remaining 16 low risk of bias.

Regarding the eligible controlled trials (CT), all five showed low risk of bias in confounding and some concerns in intervention assignment, intervention adherence, missing data as well as outcome. Also, all five CT showed not applicable risk of bias in reported results, while in selection bias one CT [18] displayed some concerns and four [19-22] low risk of bias. Ten of the cross-sectional studies (CSS)[23-32] showed low risk and five [33-37] not applicable risk, in selection bias. In performance bias five CSS [23,24,30,32,38] displayed low risk, while the remaining 11 showed not applicable risk of bias. In detection bias, two CSS [25,27] displayed high risk, five [23,30,32,33,38] some concerns and the remaining nine showed low risk of bias. In attrition bias, six CSS [23,25-28,35] showed low risk and 10 not applicable risk of bias. In selective outcome, two CSS [25,29] displayed some concerns and the remaining 14 low risk of bias, while in confounding all the CSS showed low risk apart from three studies [29,30,38] that displayed some concerns.

Supplementary Table S2: Risk of bias assessment outcomes Key: +: low, ?: some concerns, -: high, N: not applicable.

Randomized controlled trials						
First author	Randomization process	Intervention assignment	Intervention adherence	Missing data	Outcome	Reported results
Bohn-GoldBaum, 2019	?	-	-	-	+	+
Bohn-GoldBaum, 2020	+	+	?	+	+	+
Campbell, 2010	-	-	?	+	+	+
Edwards, 2008	?	-	-	-	+	+
Edwards, 2007	-	?	?	+	+	+
Edwards, 2006	?	-	-	-	+	+
Edwards, 2010	-	+	+	+	+	+
Edwards, 2012	?	+	?	+	+	+
Elzayat 2021	+	?	?	+	+	+
Hayney, 2014	?	+	?	+	+	+
Kohut, 2004	-	+	?	+	+	+
Kohut, 2005	-	+	?	+	-	-
Long, 2013	?	-	-	-	+	+
Long, 2012	?	+	?	+	+	+
Monteiro 2020	?	?	?	+	+	+
Ranadive, 2014	?	+	+	+	+	+
Woods, 2009	+	+	?	+	+	+
**Controlled Trials**						

First author	Confounding	Selection	Intervention assignment	Intervention adherence	Missing data	Outcome	Reported results
Bachi, 2013	+	+	?	?	?	?	N
Bruunsgaard, 1997	+	+	?	?	?	?	N
Felismino 2021	+	+	?	?	?	?	N
Whitham & Blannin, 2003	+	?	?	?	?	?	N
Yang, 2007	+	+	?	?	?	?	N
**Cross-sectional studies**							
First author	Selection	Performance	Detection	Attrition	Selective Outcome	Confounding	
Ayling, 2018	N	N	?	N	+	+	
de Araújo, 2015	+	+	?	+	+	+	
Gleeson, 1996	+	+	+	N	+	+	
Gualano 2021	?	+	?	N	+	?	
Kenzaka 2021	+	+	?	N	+	?	
Keshtkar-Jahromi, 2010	+	N	-	+	?	+	
Keylock, 2007	+	N	+	+	+	+	
Kohut, 2002	+	N	-	+	+	+	
Ledo, 2020	+	N	+	+	+	+	
Mitsunaga 2021	+	N	+	N	+	+	
Schuler, 2003	N	N	+	N	+	+	
Schuler, 1999	+	N	+	N	?	?	
Segerstrom, 2012	N	N	+	+	+	+	
Stewart, 2018	N	N	+	N	+	+	
Turner 2021	+	+	?	N	+	+	
Wong, 2019	N	N	+	N	+	+	

A. *Randomized controlled trials*

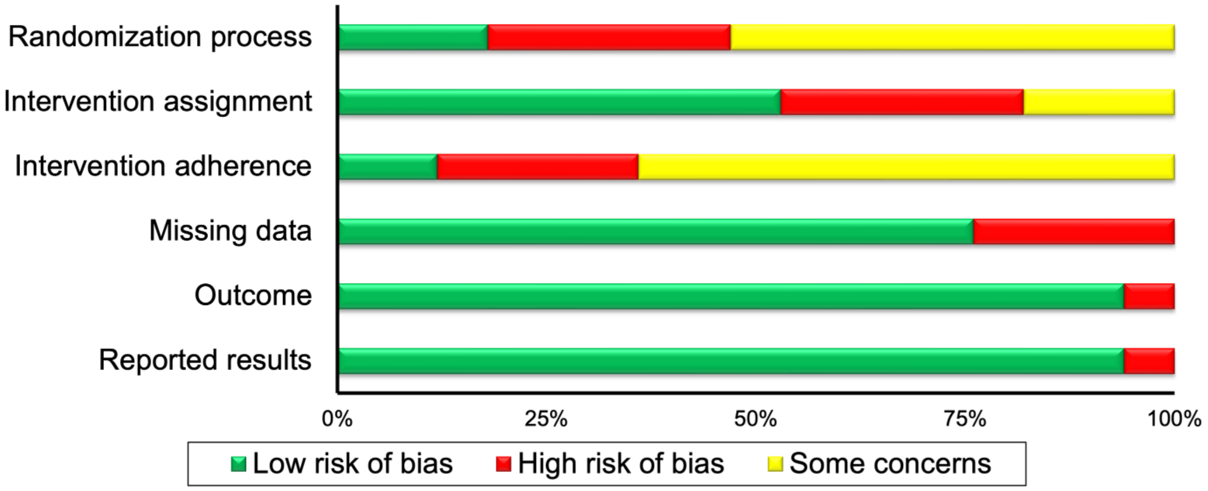

B. *Controlled trials*

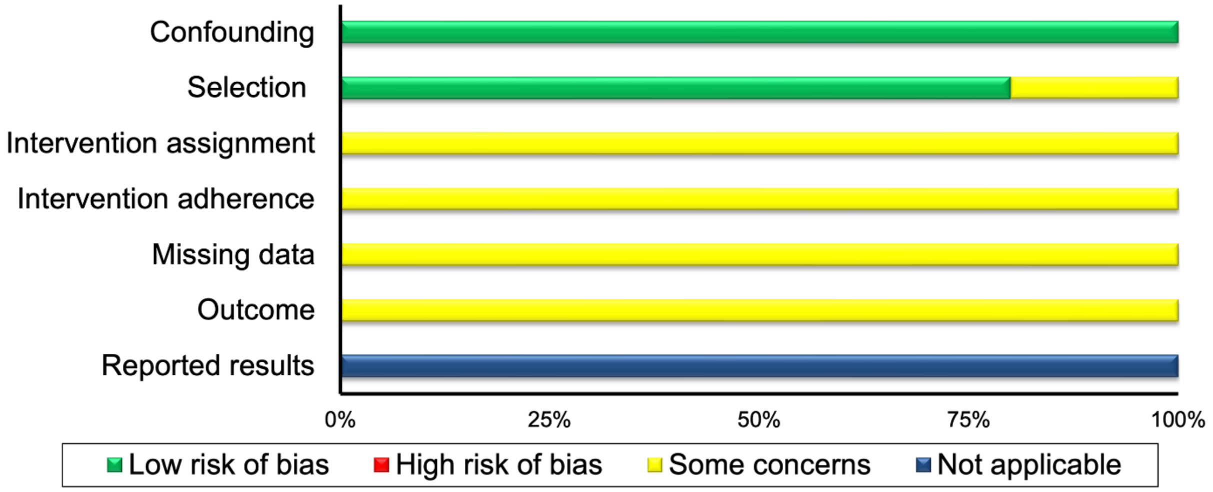

C. *Cross-sectional studies*

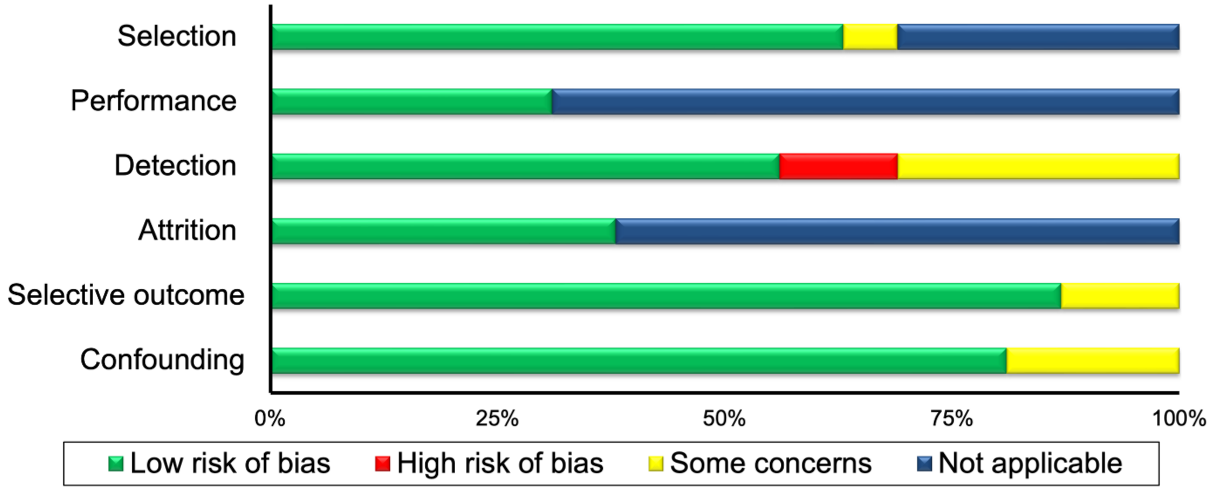

Supplementary Figure S2: Summary of risk of bias

Study or Subgroup	Exercise Δ scores Mean	SD	Total	Control Δ scores Mean	SD	Total	Weight	Std. Mean Difference IV, Random, 95% CI
**1.2.1 Aerobic**								
Long 2012 (A/Perth/16/2009-young)	−0.82	0.5	30	−0.47	0.29	30	1.9%	−0.85 [−1.37, −0.32]
Edwards 2006 (B/Shangdong/7/97-20 week-male)	−0.06	0.32	10	0.16	0.16	10	1.2%	−0.83 [−1.76, 0.09]
Edwards 2006 (A/Panama/2007/99-20th week-male)	−1.23	0.88	10	−0.66	0.44	10	1.2%	−0.78 [−1.70, 0.13]
Long 2012 (A/California/7/2009-old)	−0.61	0.62	30	−0.21	0.37	30	2.0%	−0.77 [−1.30, −0.25]
Long 2012 (A/California/7/2009-young)	−0.14	0.5	30	0.1	0.27	30	2.0%	−0.59 [−1.11, −0.07]
Edwards 2006 (A/Panama/2007/99-4 week-male)	−0.67	0.69	10	−0.32	0.5	10	1.2%	−0.56 [−1.45, 0.34]
Edwards 2006 (B/Shangdong/7/97-4 week-male)	0.2	0.24	10	0.31	0.14	10	1.2%	−0.54 [−1.43, 0.36]
Edwards 2006 (A/New Caledonia/20/99-4 week-male)	0.58	0.18	10	0.64	0.1	10	1.2%	−0.39 [−1.28, 0.49]
Ranadive 2014 (B/Brisbane-4 week)	0.24	0.64	28	0.28	0.81	27	1.9%	−0.05 [−0.58, 0.47]
Ranadive 2014 (H3N2-4 week)	0.58	0.51	28	0.6	0.43	27	1.9%	−0.04 [−0.57, 0.49]
Edwards 2006 (B/Shangdong/7/97-4 week-female)	0.18	0.25	10	0.13	0.37	10	1.2%	0.15 [−0.73, 1.03]
Edwards 2006 (A/New Caledonia/20/99-20 week-male)	0.47	0.14	10	0.43	0.19	10	1.2%	0.23 [−0.65, 1.11]
Long 2012 (B/Brisbane/60/2008-young)	0.11	0.22	30	0.01	0.43	30	2.0%	0.29 [−0.22, 0.80]
Edwards 2006 (B/Shangdong/7/97-20 week-female)	−0.06	0.34	10	−0.2	0.5	10	1.2%	0.31 [−0.57, 1.20]
Long 2012 (A/Perth/16/2009-old)	−0.47	0.47	30	−0.66	0.45	30	2.0%	0.41 [−0.10, 0.92]
Ranadive 2014 (H1N1-4 week)	0.56	0.52	28	0.3	0.53	27	1.9%	0.49 [−0.05, 1.03]
Edwards 2006 (A/New Caledonia/20/99-20 week-femal)	0.37	0.19	10	0.19	0.43	10	1.2%	0.52 [−0.38, 1.41]
Edwards 2006 (A/New Caledonia/20/99-4 week-female)	0.59	0.18	10	0.45	0.21	10	1.2%	0.69 [−0.22, 1.59]
Long 2012 (B/Brisbane/60/2008-old)	−0.2	0.49	30	−0.69	0.63	30	1.9%	0.86 [0.33, 1.39]
Edwards 2006 (A/Panama/2007/99-20th week-female)	−1.53	0.71	10	−3.7	1.54	10	1.0%	1.73 [0.67, 2.80]
Edwards 2006 (A/Panama/2007/99-4 week-female)	−0.98	0.47	10	−3.77	2.03	10	1.0%	1.81 [0.74, 2.89]
**Subtotal (95% CI)**			**384**			**381**	**31.6%**	**0.06 [−0.23, 0.36]**
Heterogeneity: $Tau^2 = 0.32$; $Chi^2 = 74.97$, df = 20 ($P < 0.00001$); $I^2 = 73\%$								
Test for overall effect: Z = 0.42 (P = 0.67)								
**1.2.2 Resistance**								
Edwards 2007 (H3N2-6th week-male)	349.48	200.6	18	653.86	332.12	8	1.2%	−1.20 [−2.10, −0.29]
Edwards 2007 (B/Shanghai-6th week-male)	158.43	114.84	18	390.54	442.59	8	1.2%	−0.87 [−1.74, −0.00]
Campbell 2010 (A/Solomon)	−2.465	4.4486	116	0.52	0.19	39	2.3%	−0.77 [−1.14, −0.40]
Bohn-Goldbaum 2020 (H1N1-1 month)	0.38	0.39	23	0.59	0.04	23	1.8%	−0.74 [−1.34, −0.15]
Edwards 2007 (H3N2-20th week-male)	253.67	149.56	17	462.24	449.86	9	1.3%	−0.70 [−1.54, 0.13]
Campbell 2010 (B/Malaysia)	0.3189	0.2469	116	0.48	0.17	39	2.3%	−0.70 [−1.07, −0.33]
Edwards 2007 (B/Shanghai-20th week-male)	78.71	53.85	17	251.8	416.33	9	1.3%	−0.69 [−1.52, 0.15]
Bohn-Goldbaum 2020 (H3N2)	−0.34	0.75	23	0.04	0.55	23	1.8%	−0.57 [−1.16, 0.02]
Bohn-Goldbaum 2020 (H3N2-1 month)	0.61	0.29	23	0.72	0.22	23	1.8%	−0.42 [−1.00, 0.16]
Bohn-Goldbaum 2020 (B/Brisbane/60/2008-1 month)	0.49	0.28	23	0.57	0.27	23	1.8%	−0.29 [−0.87, 0.30]
Edwards 2007 (H1N1-6th week-male)	1,912.89	2,559.28	18	2,855.65	4,588.75	8	1.3%	−0.28 [−1.11, 0.56]
Edwards 2010 (B/Florida-28th day-exercise 85%)	633	985.94	40	832	662.58	39	2.2%	−0.23 [−0.68, 0.21]
Elzayat 2021-H3N2-25 wk post vaccination Ex-S	−11.28	120	10	6.85	72.87	10	1.2%	−0.17 [−1.05, 0.70]
Edwards 2010 (A/Uruguay-28th day-exercise 110%)	47	172.52	39	87	296.45	39	2.1%	−0.16 [−0.61, 0.28]
Elzayat 2021-H3N2-6 wk post vaccination Ex-Op	−0.24	2.42	9	6.61	66.55	10	1.2%	−0.14 [−1.04, 0.77]
Elzayat 2021-H3N2-25 wk post vaccination Ex-Op	−0.19	1.91	9	6.85	68.97	10	1.2%	−0.13 [−1.04, 0.77]
Elzayat 2021-H3N2-6 wk post vaccination Ex-S	−0.04	0.43	10	6.61	70.32	10	1.2%	−0.13 [−1.01, 0.75]
Elzayat 2021-H1N1-6 wk post vaccination Ex-Op	0.01	0.11	9	7.21	81.93	10	1.2%	−0.12 [−1.02, 0.79]
Edwards 2007 (H1N1-20th week-male)	1,158.68	1,481.81	17	1,279.89	2,080.88	9	1.4%	−0.07 [−0.88, 0.74]
Bohn-Goldbaum 2020 (H1N1)	−0.58	0.75	23	−0.53	0.78	23	1.8%	−0.06 [−0.64, 0.51]
Elzayat 2021-H1N1-25 wk post vaccination Ex-Op	2.61	29.66	9	4.86	55.23	10	1.2%	−0.05 [−0.95, 0.85]
Elzayat 2021-B-6 wk post vaccination Ex-Op	0.02	0.5	9	0.07	1.74	10	1.2%	−0.04 [−0.94, 0.86]
Edwards 2010 (A/Brisbane-28th day-exercise 85%)	304	753.28	40	317	736.1	39	2.2%	−0.02 [−0.46, 0.42]
Edwards 2010 (A/Uruguay-28th day-exercise 60%)	99	297.19	40	87	296.45	39	2.2%	0.04 [−0.40, 0.48]
Elzayat 2021-B-6 wk post vaccination Ex-S	1.02	26.76	10	0.07	1.84	10	1.2%	0.05 [−0.83, 0.92]
Elzayat 2021-H1N1-6 wk post vaccination Ex-S	14.22	170.73	10	7.3	87.64	10	1.2%	0.05 [−0.83, 0.93]
Elzayat 2021-B-25 wk post vaccination Ex-S	0.04	1.05	10	−1	26.24	10	1.2%	0.05 [−0.82, 0.93]
Elzayat 2021-H1N1-25 wk post vaccination Ex-S	10.28	123.42	10	4.86	58.35	10	1.2%	0.05 [−0.82, 0.93]
Elzayat 2021-B-25 wk post vaccination Ex-Op	0.04	0.99	9	−1	24.84	10	1.2%	0.05 [−0.85, 0.96]
Edwards 2010 (A/Uruguay-28th day-exercise 85%)	114	399.84	40	87	296.45	39	2.2%	0.08 [−0.37, 0.52]
Edwards 2010 (A/Brisbane-28th day-exercise 60%)	403	1,036.04	40	317	736.1	39	2.2%	0.09 [−0.35, 0.54]
Edwards 2010 (B/Florida-28th day-exercise 110%)	950	1,467.7	39	832	662.58	39	2.1%	0.10 [−0.34, 0.55]
Edwards 2010 (A/Brisbane-28th day-exercise 110%)	425	1,181.5	39	317	736.1	39	2.1%	0.11 [−0.34, 0.55]
Edwards 2007 (H3N2-6th week-female)	678.21	725.69	20	573.05	311.42	11	1.5%	0.17 [−0.57, 0.90]
Edwards 2007 (B/Shanghai-6th week-female)	412.17	773.5	20	274.53	396.68	11	1.5%	0.20 [−0.54, 0.94]
Bohn-Goldbaum 2020 (B/Brisbane/60/2008)	0.13	0.39	23	0.03	0.5	23	1.8%	0.22 [−0.36, 0.80]
Edwards 2007 (H1N1-6th week-female)	1,549.25	4,321.44	20	646.9	787.86	11	1.5%	0.25 [−0.49, 0.99]
Edwards 2010 (B/Florida-28th day-exercise 60%)	1,236	1,722.2	40	832	662.58	39	2.2%	0.31 [−0.14, 0.75]
Edwards 2007 (B/Shanghai-20th week-female)	202	232.51	20	133.82	21.42	11	1.5%	0.35 [−0.39, 1.09]
Edwards 2007 (H1N1-20th week-female)	768.11	845.51	20	364.07	438.14	11	1.5%	0.54 [−0.21, 1.29]
Edwards 2007 (H3N2-20th week-female)	450.67	312.2	20	279.63	173.39	11	1.5%	0.61 [−0.14, 1.36]
Campbell 2010 (A/Wisconsin)	0.2568	0.2795	116	0.06	0.29	39	2.3%	0.69 [0.32, 1.07]
**Subtotal (95% CI)**			**1182**			**843**	**68.4%**	**−0.10 [−0.23, 0.04]**
Heterogeneity: $Tau^2 = 0.09$; $Chi^2 = 79.64$, df = 41 ($P = 0.0003$); $I^2 = 49\%$								
Test for overall effect: Z = 1.41 (P = 0.16)								
**Total (95% CI)**			**1566**			**1224**	**100.0%**	**−0.05 [−0.18, 0.08]**
Heterogeneity: $Tau^2 = 0.15$; $Chi^2 = 156.18$, df = 62 ($P < 0.00001$); $I^2 = 60\%$								
Test for overall effect: Z = 0.79 (P = 0.43)								
Test for subgroup differences: $Chi^2 = 0.95$, df = 1 (P = 0.33), $I^2 = 0\%$								

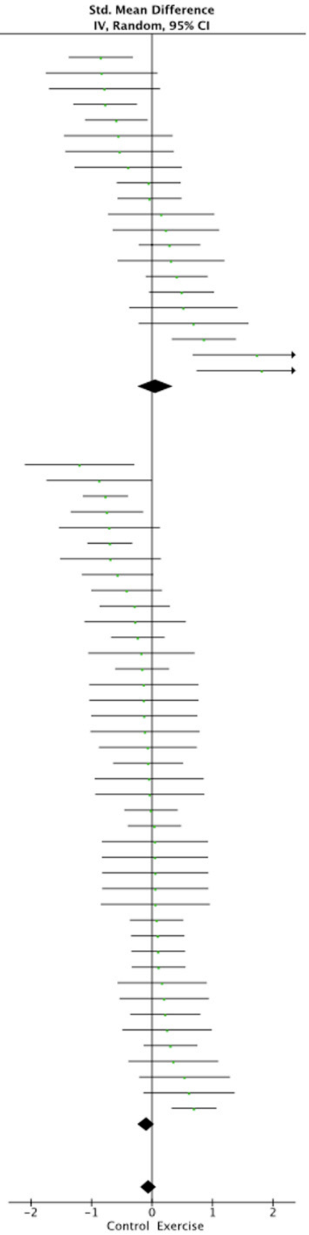

Supplementary Figure S3: Forest plot of the effect of acute exercise on influenza vaccine antibodies (subgroup analysis for exercise type). Δ scores: post intervention – baseline; SD: standard deviation; 95% CI: 95% confidence interval

Study or Subgroup	Exercise Δ scores Mean	SD	Total	Control Δ scores Mean	SD	Total	Weight	Std. Mean Difference IV, Random, 95% CI
**1.3.1 H1N1**								
Long 2012 (A/California/7/2009-old)	-0.61	0.62	30	-0.21	0.37	30	2.0%	-0.77 [-1.30, -0.25]
Campbell 2010 (A/Solomon)	-2.465	4.4486	116	0.52	0.19	39	2.3%	-0.77 [-1.14, -0.40]
Bohn-Goldbaum 2020 (H1N1-1 month)	0.38	0.39	23	0.59	0.04	23	1.8%	-0.74 [-1.34, -0.15]
Long 2012 (A/California/7/2009-young)	-0.14	0.5	30	0.1	0.27	30	2.0%	-0.59 [-1.11, -0.07]
Edwards 2006 (A/New Caledonia/20/99-4 week-male)	0.58	0.18	10	0.64	0.1	10	1.2%	-0.39 [-1.28, 0.49]
Edwards 2007 (H1N1-6th week-male)	1,912.89	2,559.28	18	2,855.65	4,588.75	8	1.3%	-0.28 [-1.11, 0.56]
Elzayat 2021-H1N1-6 wk post vaccination Ex-Op	0.01	0.11	9	7.21	81.93	10	1.2%	-0.12 [-1.02, 0.79]
Edwards 2007 (H1N1-20th week-male)	1,158.68	1,481.81	17	1,279.89	2,080.88	9	1.4%	-0.07 [-0.88, 0.74]
Bohn-Goldbaum 2020 (H1N1)	-0.58	0.75	23	-0.53	0.78	23	1.8%	-0.06 [-0.64, 0.51]
Elzayat 2021-H1N1-25 wk post vaccination Ex-Op	2.61	29.66	9	4.86	55.23	10	1.2%	-0.05 [-0.95, 0.85]
Elzayat 2021-H1N1-6 wk post vaccination Ex-S	14.22	170.73	10	7.3	87.64	10	1.2%	0.05 [-0.83, 0.93]
Elzayat 2021-H1N1-25 wk post vaccination Ex-S	10.28	123.42	10	4.86	58.35	10	1.2%	0.05 [-0.82, 0.93]
Edwards 2006 (A/New Caledonia/20/99-20 week-male)	0.47	0.14	10	0.43	0.19	10	1.2%	0.23 [-0.65, 1.11]
Edwards 2007 (H1N1-6th week-female)	1,549.25	4,321.44	20	646.9	787.86	11	1.5%	0.25 [-0.49, 0.99]
Ranadive 2014 (H1N1-4 week)	0.56	0.52	28	0.3	0.53	27	1.9%	0.49 [-0.05, 1.03]
Edwards 2006 (A/New Caledonia/20/99-20 week-femal)	0.37	0.19	10	0.19	0.43	10	1.2%	0.52 [-0.38, 1.41]
Edwards 2007 (H1N1-20th week-female)	768.11	845.51	20	364.07	438.14	11	1.5%	0.54 [-0.21, 1.29]
Edwards 2006 (A/New Caledonia/20/99-4 week-female)	0.59	0.18	10	0.45	0.21	10	1.2%	0.69 [-0.22, 1.59]
**Subtotal (95% CI)**			**403**			**291**	**27.1%**	**-0.11 [-0.36, 0.13]**
Heterogeneity: Tau² = 0.15; Chi² = 38.61, df = 17 (P = 0.002); I² = 56%								
Test for overall effect: Z = 0.90 (P = 0.37)								
**1.3.2 H3N2**								
Edwards 2007 (H3N2-6th week-male)	349.48	200.6	18	653.86	332.12	8	1.2%	-1.20 [-2.10, -0.29]
Long 2012 (A/Perth/16/2009-young)	-0.82	0.5	30	-0.47	0.29	30	1.9%	-0.85 [-1.37, -0.32]
Edwards 2006 (A/Panama/2007/99-20th week-male)	-1.23	0.88	10	-0.66	0.44	10	1.2%	-0.78 [-1.70, 0.13]
Edwards 2007 (H3N2-20th week-male)	253.67	149.56	17	462.24	449.86	9	1.3%	-0.70 [-1.54, 0.13]
Bohn-Goldbaum 2020 (H3N2)	-0.34	0.75	23	0.04	0.55	23	1.8%	-0.57 [-1.16, 0.02]
Edwards 2006 (A/Panama/2007/99-4 week-male)	-0.67	0.69	10	-0.32	0.5	10	1.2%	-0.56 [-1.45, 0.34]
Bohn-Goldbaum 2020 (H3N2-1 month)	0.61	0.29	23	0.72	0.22	23	1.8%	-0.42 [-1.00, 0.16]
Elzayat 2021-H3N2-25 wk post vaccination Ex-S	-11.28	120	10	6.85	72.87	10	1.2%	-0.17 [-1.05, 0.70]
Edwards 2010 (A/Uruguay-28th day-exercise 110%)	47	172.52	39	87	296.45	39	2.1%	-0.16 [-0.61, 0.28]
Elzayat 2021-H3N2-6 wk post vaccination Ex-Op	-0.24	2.42	9	6.61	66.55	10	1.2%	-0.14 [-1.04, 0.77]
Elzayat 2021-H3N2-25 wk post vaccination Ex-Op	-0.19	1.91	9	6.85	68.97	10	1.2%	-0.13 [-1.04, 0.77]
Elzayat 2021-H3N2-6 wk post vaccination Ex-S	-0.04	0.43	10	6.61	70.32	10	1.2%	-0.13 [-1.01, 0.75]
Ranadive 2014 (H3N2-4 week)	0.58	0.51	28	0.6	0.43	27	1.9%	-0.04 [-0.57, 0.49]
Edwards 2010 (A/Brisbane-28th day-exercise 85%)	304	753.28	40	317	736.1	39	2.2%	-0.02 [-0.46, 0.42]
Edwards 2010 (A/Uruguay-28th day-exercise 60%)	99	297.19	40	87	296.45	39	2.2%	0.04 [-0.40, 0.48]
Edwards 2010 (A/Uruguay-28th day-exercise 85%)	114	399.84	40	87	296.45	39	2.2%	0.08 [-0.37, 0.52]
Edwards 2010 (A/Brisbane-28th day-exercise 60%)	403	1,036.04	40	317	736.1	39	2.2%	0.09 [-0.35, 0.54]
Edwards 2010 (A/Brisbane-28th day-exercise 110%)	425	1,181.5	39	317	736.1	39	2.1%	0.11 [-0.34, 0.55]
Edwards 2007 (H3N2-6th week-female)	678.21	725.69	20	573.05	311.42	11	1.5%	0.17 [-0.57, 0.90]
Long 2012 (A/Perth/16/2009-old)	-0.47	0.47	30	-0.66	0.45	30	2.0%	0.41 [-0.10, 0.92]
Edwards 2007 (H3N2-20th week-female)	450.67	312.2	20	279.63	173.39	11	1.5%	0.61 [-0.14, 1.36]
Campbell 2010 (A/Wisconsin)	0.2568	0.2795	116	0.06	0.29	39	2.3%	0.69 [0.32, 1.07]
Edwards 2006 (A/Panama/2007/99-20th week-female)	-1.53	0.71	10	-3.7	1.54	10	1.0%	1.73 [0.67, 2.80]
Edwards 2006 (A/Panama/2007/99-4 week-female)	-0.98	0.47	10	-3.77	2.03	10	1.0%	1.81 [0.74, 2.89]
**Subtotal (95% CI)**			**641**			**525**	**39.4%**	**-0.02 [-0.24, 0.20]**
Heterogeneity: Tau² = 0.19; Chi² = 70.22, df = 23 (P < 0.00001); I² = 67%								
Test for overall effect: Z = 0.19 (P = 0.85)								
**1.3.3 B**								
Edwards 2007 (B/Shanghai-6th week-male)	158.43	114.84	18	390.54	442.59	8	1.2%	-0.87 [-1.74, -0.00]
Edwards 2006 (B/Shangdong/7/97-20 week-male)	-0.06	0.32	10	0.16	0.16	10	1.2%	-0.83 [-1.76, 0.09]
Campbell 2010 (B/Malaysia)	0.3189	0.2469	116	0.48	0.17	39	2.3%	-0.70 [-1.07, -0.33]
Edwards 2007 (B/Shanghai-20th week-male)	78.71	53.85	17	251.8	416.33	9	1.3%	-0.69 [-1.52, 0.15]
Edwards 2006 (B/Shangdong/7/97-4 week-male)	0.2	0.24	10	0.31	0.14	10	1.2%	-0.54 [-1.43, 0.36]
Bohn-Goldbaum 2020 (B/Brisbane/60/2008-1 month)	0.49	0.28	23	0.57	0.27	23	1.8%	-0.29 [-0.87, 0.30]
Edwards 2010 (B/Florida-28th day-exercise 85%)	633	985.94	40	832	662.58	39	2.2%	-0.23 [-0.68, 0.21]
Ranadive 2014 (B/Brisbane-4 week)	0.24	0.64	28	0.28	0.81	27	1.9%	-0.05 [-0.58, 0.47]
Elzayat 2021-B-6 wk post vaccination Ex-Op	0.02	0.5	9	0.07	1.74	10	1.2%	-0.04 [-0.94, 0.86]
Elzayat 2021-B-6 wk post vaccination Ex-S	1.02	26.76	10	0.07	1.84	10	1.2%	0.05 [-0.83, 0.92]
Elzayat 2021-B-25 wk post vaccination Ex-S	0.04	1.05	10	-1	26.24	10	1.2%	0.05 [-0.82, 0.93]
Elzayat 2021-B-25 wk post vaccination Ex-Op	0.04	0.99	9	-1	24.84	10	1.2%	0.05 [-0.85, 0.96]
Edwards 2010 (B/Florida-28th day-exercise 110%)	950	1,467.7	39	832	662.58	39	2.1%	0.10 [-0.34, 0.55]
Edwards 2006 (B/Shangdong/7/97-4 week-female)	0.18	0.25	10	0.13	0.37	10	1.2%	0.15 [-0.73, 1.03]
Edwards 2007 (B/Shanghai-6th week-female)	412.17	773.5	20	274.53	396.68	11	1.5%	0.20 [-0.54, 0.94]
Bohn-Goldbaum 2020 (B/Brisbane/60/2008)	0.13	0.39	23	0.03	0.5	23	1.8%	0.22 [-0.36, 0.80]
Long 2012 (B/Brisbane/60/2008-young)	0.11	0.22	30	0.01	0.43	30	2.0%	0.29 [-0.22, 0.80]
Edwards 2010 (B/Florida-28th day-exercise 60%)	1,236	1,722.2	40	832	662.58	39	2.2%	0.31 [-0.14, 0.75]
Edwards 2006 (B/Shangdong/7/97-20 week-female)	-0.06	0.34	10	-0.2	0.5	10	1.2%	0.31 [-0.57, 1.20]
Edwards 2007 (B/Shanghai-20th week-female)	202	232.51	20	133.82	21.42	11	1.5%	0.35 [-0.39, 1.09]
Long 2012 (B/Brisbane/60/2008-old)	-0.2	0.49	30	-0.69	0.63	30	1.9%	0.86 [0.33, 1.39]
**Subtotal (95% CI)**			**522**			**408**	**33.5%**	**-0.04 [-0.24, 0.17]**
Heterogeneity: Tau² = 0.11; Chi² = 41.75, df = 20 (P = 0.003); I² = 52%								
Test for overall effect: Z = 0.35 (P = 0.73)								
**Total (95% CI)**			**1566**			**1224**	**100.0%**	**-0.05 [-0.18, 0.08]**
Heterogeneity: Tau² = 0.15; Chi² = 156.18, df = 62 (P < 0.00001); I² = 60%								
Test for overall effect: Z = 0.79 (P = 0.43)								
Test for subgroup differences: Chi² = 0.33, df = 2 (P = 0.85), I² = 0%								

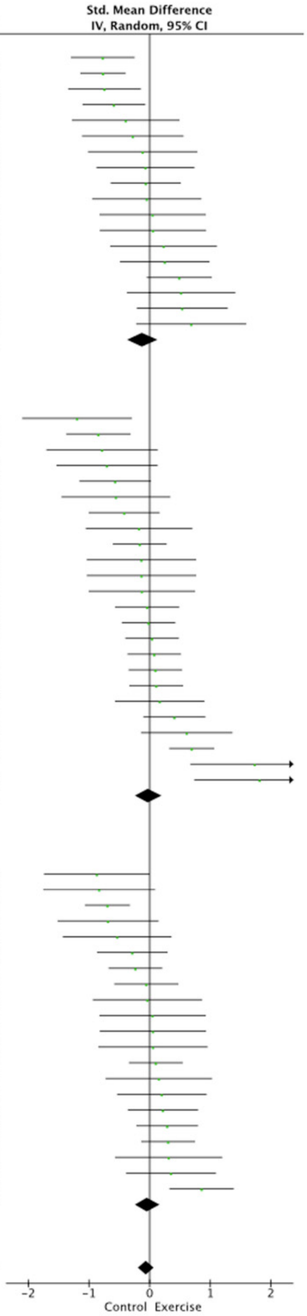

Supplementary Figure S4: Forest plot of the effect of acute exercise on influenza vaccine antibodies (subgroup analysis for antibodies type). Δ scores: post intervention – baseline; SD: standard deviation; 95% CI: 95% confidence interval

Study or Subgroup	Exercise Δ scores Mean	SD	Total	Control Δ scores Mean	SD	Total	Weight	Std. Mean Difference IV, Random, 95% CI
**1.4.1 Low < 65% HR 1 RM**								
Long 2012 (A/Perth/16/2009-young)	-0.82	0.5	30	-0.47	0.29	30	1.9%	-0.85 [-1.37, -0.32]
Long 2012 (A/California/7/2009-old)	-0.61	0.62	30	-0.21	0.37	30	2.0%	-0.77 [-1.30, -0.25]
Bohn-Goldbaum 2020 (H1N1-1 month)	0.38	0.39	23	0.59	0.04	23	1.8%	-0.74 [-1.34, -0.15]
Long 2012 (A/California/7/2009-young)	-0.14	0.5	30	0.1	0.27	30	2.0%	-0.59 [-1.11, -0.07]
Bohn-Goldbaum 2020 (H3N2)	-0.34	0.75	23	0.04	0.55	23	1.8%	-0.57 [-1.16, 0.02]
Bohn-Goldbaum 2020 (H3N2-1 month)	0.61	0.29	23	0.72	0.22	23	1.8%	-0.42 [-1.00, 0.16]
Bohn-Goldbaum 2020 (B/Brisbane/60/2008-1 month)	0.49	0.28	23	0.57	0.27	23	1.8%	-0.29 [-0.87, 0.30]
Bohn-Goldbaum 2020 (H1N1)	-0.58	0.75	23	-0.53	0.78	23	1.8%	-0.06 [-0.64, 0.51]
Ranadive 2014 (B/Brisbane-4 week)	0.24	0.64	28	0.28	0.81	27	1.9%	-0.05 [-0.58, 0.47]
Ranadive 2014 (H3N2-4 week)	0.58	0.51	28	0.6	0.43	27	1.9%	-0.04 [-0.57, 0.49]
Edwards 2010 (A/Uruguay-28th day-exercise 60%)	99	297.19	40	87	296.45	39	2.2%	0.04 [-0.40, 0.48]
Edwards 2010 (A/Brisbane-28th day-exercise 60%)	403	1,036.04	40	317	736.1	39	2.2%	0.09 [-0.35, 0.54]
Bohn-Goldbaum 2020 (B/Brisbane/60/2008)	0.13	0.39	23	0.03	0.5	23	1.8%	0.22 [-0.36, 0.80]
Long 2012 (B/Brisbane/60/2008-young)	0.11	0.22	30	0.01	0.43	30	2.0%	0.29 [-0.22, 0.80]
Edwards 2010 (B/Florida-28th day-exercise 60%)	1,236	1,722.2	40	832	662.58	39	2.2%	0.31 [-0.14, 0.75]
Long 2012 (A/Perth/16/2009-old)	-0.47	0.47	30	-0.66	0.45	30	2.0%	0.41 [-0.10, 0.92]
Ranadive 2014 (H1N1-4 week)	0.56	0.52	28	0.3	0.53	27	1.9%	0.49 [-0.05, 1.03]
Long 2012 (B/Brisbane/60/2008-old)	-0.2	0.49	30	-0.69	0.63	30	1.9%	0.86 [0.33, 1.39]
**Subtotal (95% CI)**			**522**			**516**	**34.9%**	**-0.08 [-0.30, 0.14]**
Heterogeneity: $Tau^2 = 0.16$; $Chi^2 = 54.08$, df = 17 ($P < 0.00001$); $I^2 = 69\%$								
Test for overall effect: Z = 0.74 (P = 0.46)								
**1.4.3 High >75% HR 1 RM**								
Edwards 2007 (H3N2-6th week-male)	349.48	200.6	18	653.86	332.12	8	1.2%	-1.20 [-2.10, -0.29]
Edwards 2007 (B/Shanghai-6th week-male)	158.43	114.84	18	390.54	442.59	8	1.2%	-0.87 [-1.74, -0.00]
Edwards 2006 (B/Shangdong/7/97-20 week-male)	-0.06	0.32	10	0.16	0.16	10	1.2%	-0.83 [-1.76, 0.09]
Edwards 2006 (A/Panama/2007/99-20th week-male)	-1.23	0.88	10	-0.66	0.44	10	1.2%	-0.78 [-1.70, 0.13]
Campbell 2010 (A/Solomon)	-2.465	4.4486	116	0.52	0.19	39	2.3%	-0.77 [-1.14, -0.40]
Edwards 2007 (H3N2-20th week-male)	253.67	149.56	17	462.24	449.86	9	1.3%	-0.70 [-1.54, 0.13]
Campbell 2010 (B/Malaysia)	0.3189	0.2469	116	0.48	0.17	39	2.3%	-0.70 [-1.07, -0.33]
Edwards 2007 (B/Shanghai-20th week-male)	78.71	53.85	17	251.8	416.33	9	1.3%	-0.69 [-1.52, 0.15]
Edwards 2006 (A/Panama/2007/99-4 week-male)	-0.67	0.69	10	-0.32	0.5	10	1.2%	-0.56 [-1.45, 0.34]
Edwards 2006 (B/Shangdong/7/97-4 week-male)	0.2	0.24	10	0.31	0.14	10	1.2%	-0.54 [-1.43, 0.36]
Edwards 2006 (A/New Caledonia/20/99-4 week-male)	0.58	0.18	10	0.64	0.1	10	1.2%	-0.39 [-1.28, 0.49]
Edwards 2007 (H1N1-6th week-male)	1,912.89	2,559.28	18	2,855.65	4,588.75	8	1.3%	-0.28 [-1.11, 0.56]
Edwards 2010 (B/Florida-28th day-exercise 85%)	633	985.94	40	832	662.58	39	2.2%	-0.23 [-0.68, 0.21]
Elzayat 2021-H3N2-25 wk post vaccination Ex-S	-11.28	120	10	6.85	72.87	10	1.2%	-0.17 [-1.05, 0.70]
Edwards 2010 (A/Uruguay-28th day-exercise 110%)	47	172.52	39	87	296.45	39	2.1%	-0.16 [-0.61, 0.28]
Elzayat 2021-H3N2-6 wk post vaccination Ex-Op	-0.24	2.42	9	6.61	66.55	10	1.2%	-0.14 [-1.04, 0.77]
Elzayat 2021-H3N2-25 wk post vaccination Ex-Op	-0.19	1.91	9	6.85	68.97	10	1.2%	-0.13 [-1.04, 0.77]
Elzayat 2021-H3N2-6 wk post vaccination Ex-S	-0.04	0.43	10	6.61	70.32	10	1.2%	-0.13 [-1.01, 0.75]
Elzayat 2021-H1N1-6 wk post vaccination Ex-Op	0.01	0.11	9	7.21	81.93	10	1.2%	-0.12 [-1.02, 0.79]
Edwards 2007 (H1N1-20th week-male)	1,158.68	1,481.81	17	1,279.89	2,080.88	9	1.4%	-0.07 [-0.88, 0.74]
Elzayat 2021-H1N1-25 wk post vaccination Ex-Op	2.61	29.66	9	4.86	55.23	10	1.2%	-0.05 [-0.95, 0.85]
Elzayat 2021-B-6 wk post vaccination Ex-Op	0.02	0.5	9	0.07	1.74	10	1.2%	-0.04 [-0.94, 0.86]
Edwards 2010 (A/Brisbane-28th day-exercise 85%)	304	753.28	40	317	736.1	39	2.2%	-0.02 [-0.46, 0.42]
Elzayat 2021-B-6 wk post vaccination Ex-S	1.02	26.76	10	0.07	1.84	10	1.2%	0.05 [-0.83, 0.92]
Elzayat 2021-H1N1-6 wk post vaccination Ex-S	14.22	170.73	10	7.3	87.64	10	1.2%	0.05 [-0.83, 0.93]
Elzayat 2021-B-25 wk post vaccination Ex-S	0.04	1.05	10	-1	26.24	10	1.2%	0.05 [-0.82, 0.93]
Elzayat 2021-H1N1-25 wk post vaccination Ex-S	10.28	123.42	10	4.86	58.35	10	1.2%	0.05 [-0.82, 0.93]
Elzayat 2021-B-25 wk post vaccination Ex-Op	0.04	0.99	9	-1	24.84	10	1.2%	0.05 [-0.85, 0.96]
Edwards 2010 (A/Uruguay-28th day-exercise 85%)	114	399.84	40	87	296.45	39	2.2%	0.08 [-0.37, 0.52]
Edwards 2010 (B/Florida-28th day-exercise 110%)	950	1,467.7	39	832	662.58	39	2.1%	0.10 [-0.34, 0.55]
Edwards 2010 (A/Brisbane-28th day-exercise 110%)	425	1,181.5	39	317	736.1	39	2.1%	0.11 [-0.34, 0.55]
Edwards 2006 (B/Shangdong/7/97-4 week-female)	0.18	0.25	10	0.13	0.37	10	1.2%	0.15 [-0.73, 1.03]
Edwards 2007 (H3N2-6th week-female)	678.21	725.69	20	573.05	311.42	11	1.5%	0.17 [-0.57, 0.90]
Edwards 2007 (B/Shanghai-6th week-female)	412.17	773.5	20	274.53	396.68	11	1.5%	0.20 [-0.54, 0.94]
Edwards 2006 (A/New Caledonia/20/99-20 week-male)	0.47	0.14	10	0.43	0.19	10	1.2%	0.23 [-0.65, 1.11]
Edwards 2007 (H1N1-6th week-female)	1,549.25	4,321.44	20	646.9	787.86	11	1.5%	0.25 [-0.49, 0.99]
Edwards 2006 (B/Shangdong/7/97-20 week-female)	-0.06	0.34	10	-0.2	0.5	10	1.2%	0.31 [-0.57, 1.20]
Edwards 2007 (B/Shanghai-20th week-female)	202	232.51	20	133.82	21.42	11	1.5%	0.35 [-0.39, 1.09]
Edwards 2006 (A/New Caledonia/20/99-20 week-femal)	0.37	0.19	10	0.19	0.43	10	1.2%	0.52 [-0.38, 1.41]
Edwards 2007 (H1N1-20th week-female)	768.11	845.51	20	364.07	438.14	11	1.5%	0.54 [-0.21, 1.29]
Edwards 2007 (H3N2-20th week-female)	450.67	312.2	20	279.63	173.39	11	1.5%	0.61 [-0.14, 1.36]
Edwards 2006 (A/New Caledonia/20/99-4 week-female)	0.59	0.18	10	0.45	0.21	10	1.2%	0.69 [-0.22, 1.59]
Campbell 2010 (A/Wisconsin)	0.2568	0.2795	116	0.06	0.29	39	2.3%	0.69 [0.32, 1.07]
Edwards 2006 (A/Panama/2007/99-20th week-female)	-1.53	0.71	10	-3.7	1.54	10	1.0%	1.73 [0.67, 2.80]
Edwards 2006 (A/Panama/2007/99-4 week-female)	-0.98	0.47	10	-3.77	2.03	10	1.0%	1.81 [0.74, 2.89]
**Subtotal (95% CI)**			**1044**			**708**	**65.1%**	**-0.04 [-0.20, 0.13]**
Heterogeneity: $Tau^2 = 0.16$; $Chi^2 = 102.09$, df = 44 ($P < 0.00001$); $I^2 = 57\%$								
Test for overall effect: Z = 0.43 (P = 0.67)								
**Total (95% CI)**			**1566**			**1224**	**100.0%**	**-0.05 [-0.18, 0.08]**
Heterogeneity: $Tau^2 = 0.15$; $Chi^2 = 156.18$, df = 62 ($P < 0.00001$); $I^2 = 60\%$								
Test for overall effect: Z = 0.79 (P = 0.43)								
Test for subgroup differences: $Chi^2 = 0.12$, df = 1 ($P = 0.73$), $I^2 = 0\%$								

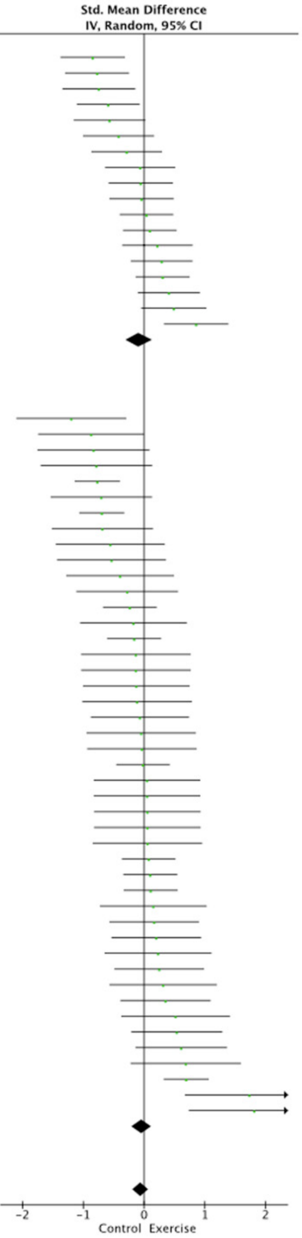

Supplementary Figure S5: Forest plot of the effect of acute exercise on influenza vaccine antibodies (subgroup analysis for exercise intensity). Δ scores: post intervention – baseline; SD: standard deviation; 95% CI: 95% confidence interval

Study or Subgroup	Exercise Δ scores Mean	SD	Total	Control Δ scores Mean	SD	Total	Weight	Std. Mean Difference IV, Random, 95% CI
**1.5.1 < 50 years**								
Edwards 2007 (H3N2-6th week-male)	349.48	200.6	18	653.86	332.12	8	1.2%	-1.20 [-2.10, -0.29]
Edwards 2007 (B/Shanghai-6th week-male)	158.43	114.84	18	390.54	442.59	8	1.2%	-0.87 [-1.74, -0.00]
Long 2012 (A/Perth/16/2009-young)	-0.82	0.5	30	-0.47	0.29	30	1.9%	-0.85 [-1.37, -0.32]
Edwards 2006 (B/Shangdong/7/97-20 week-male)	-0.06	0.32	10	0.16	0.16	10	1.2%	-0.83 [-1.76, 0.09]
Edwards 2006 (A/Panama/2007/99-20th week-male)	-1.23	0.88	10	-0.66	0.44	10	1.2%	-0.78 [-1.70, 0.13]
Campbell 2010 (A/Solomon)	-2.465	4.4486	116	0.52	0.19	39	2.3%	-0.77 [-1.14, -0.40]
Edwards 2007 (H3N2-20th week-male)	253.67	149.56	17	462.24	449.86	9	1.3%	-0.70 [-1.54, 0.13]
Campbell 2010 (B/Malaysia)	0.3189	0.2469	116	0.48	0.17	39	2.3%	-0.70 [-1.07, -0.33]
Edwards 2007 (B/Shanghai-20th week-male)	78.71	53.85	17	251.8	416.33	9	1.3%	-0.69 [-1.52, 0.15]
Long 2012 (A/California/7/2009-young)	-0.14	0.5	30	0.1	0.27	30	2.0%	-0.59 [-1.11, -0.07]
Edwards 2006 (A/Panama/2007/99-4 week-male)	-0.67	0.69	10	-0.32	0.5	10	1.2%	-0.56 [-1.45, 0.34]
Edwards 2006 (B/Shangdong/7/97-4 week-male)	0.2	0.24	10	0.31	0.14	10	1.2%	-0.54 [-1.43, 0.36]
Edwards 2006 (A/New Caledonia/20/99-4 week-male)	0.58	0.18	10	0.64	0.1	10	1.2%	-0.39 [-1.28, 0.49]
Edwards 2007 (H1N1-6th week-male)	1,912.89	2,559.28	18	2,855.65	4,588.75	8	1.3%	-0.28 [-1.11, 0.56]
Edwards 2010 (B/Florida-28th day-exercise 85%)	633	985.94	40	832	662.58	39	2.2%	-0.23 [-0.68, 0.21]
Edwards 2010 (A/Uruguay-28th day-exercise 110%)	47	172.52	39	87	296.45	39	2.1%	-0.16 [-0.61, 0.28]
Edwards 2007 (H1N1-20th week-male)	1,158.68	1,481.81	17	1,279.89	2,080.88	9	1.4%	-0.07 [-0.88, 0.74]
Edwards 2010 (A/Brisbane-28th day-exercise 85%)	304	753.28	40	317	736.1	39	2.2%	-0.02 [-0.46, 0.42]
Edwards 2010 (A/Uruguay-28th day-exercise 60%)	99	297.19	40	87	296.45	39	2.2%	0.04 [-0.40, 0.48]
Edwards 2010 (A/Uruguay-28th day-exercise 85%)	114	399.84	40	87	296.45	39	2.2%	0.08 [-0.37, 0.52]
Edwards 2010 (A/Brisbane-28th day-exercise 60%)	403	1,036.04	40	317	736.1	39	2.2%	0.09 [-0.35, 0.54]
Edwards 2010 (B/Florida-28th day-exercise 110%)	950	1,467.7	39	832	662.58	39	2.1%	0.10 [-0.34, 0.55]
Edwards 2010 (A/Brisbane-28th day-exercise 110%)	425	1,181.5	39	317	736.1	39	2.1%	0.11 [-0.34, 0.55]
Edwards 2006 (B/Shangdong/7/97-4 week-female)	0.18	0.25	10	0.13	0.37	10	1.2%	0.15 [-0.73, 1.03]
Edwards 2007 (H3N2-6th week-female)	678.21	725.69	20	573.05	311.42	11	1.5%	0.17 [-0.57, 0.90]
Edwards 2007 (B/Shanghai-6th week-female)	412.17	773.5	20	274.53	396.68	11	1.5%	0.20 [-0.54, 0.94]
Edwards 2006 (A/New Caledonia/20/99-20 week-male)	0.47	0.14	10	0.43	0.19	10	1.2%	0.23 [-0.65, 1.11]
Edwards 2007 (H1N1-6th week-female)	1,549.25	4,321.44	20	646.9	787.86	11	1.5%	0.25 [-0.49, 0.99]
Long 2012 (B/Brisbane/60/2008-young)	0.11	0.22	30	0.01	0.43	30	2.0%	0.29 [-0.22, 0.80]
Edwards 2010 (B/Florida-28th day-exercise 60%)	1,236	1,722.2	40	832	662.58	39	2.2%	0.31 [-0.14, 0.75]
Edwards 2006 (B/Shangdong/7/97-20 week-female)	-0.06	0.34	10	-0.2	0.5	10	1.2%	0.31 [-0.57, 1.20]
Edwards 2007 (B/Shanghai-20th week-female)	202	232.51	20	133.82	21.42	11	1.5%	0.35 [-0.39, 1.09]
Edwards 2006 (A/New Caledonia/20/99-20 week-femal)	0.37	0.19	10	0.19	0.43	10	1.2%	0.52 [-0.38, 1.41]
Edwards 2007 (H1N1-20th week-female)	768.11	845.51	20	364.07	438.14	11	1.5%	0.54 [-0.21, 1.29]
Edwards 2007 (H3N2-20th week-female)	450.67	312.2	20	279.63	173.39	11	1.5%	0.61 [-0.14, 1.36]
Edwards 2006 (A/New Caledonia/20/99-4 week-female)	0.59	0.18	10	0.45	0.21	10	1.2%	0.69 [-0.22, 1.59]
Campbell 2010 (A/Wisconsin)	0.2568	0.2795	116	0.06	0.29	39	2.3%	0.69 [0.32, 1.07]
Edwards 2006 (A/Panama/2007/99-20th week-female)	-1.53	0.71	10	-3.7	1.54	10	1.0%	1.73 [0.67, 2.80]
Edwards 2006 (A/Panama/2007/99-4 week-female)	-0.98	0.47	10	-3.77	2.03	10	1.0%	1.81 [0.74, 2.89]
**Subtotal (95% CI)**			**1140**			**795**	**62.8%**	**-0.05 [-0.22, 0.13]**
Heterogeneity: Tau² = 0.20; Chi² = 119.14, df = 38 (P < 0.00001); I² = 68%								
Test for overall effect: Z = 0.50 (P = 0.62)								
**1.5.3 > 50 years**								
Long 2012 (A/California/7/2009-old)	-0.61	0.62	30	-0.21	0.37	30	2.0%	-0.77 [-1.30, -0.25]
Bohn-Goldbaum 2020 (H1N1-1 month)	0.38	0.39	23	0.59	0.04	23	1.8%	-0.74 [-1.34, -0.15]
Bohn-Goldbaum 2020 (H3N2)	-0.34	0.75	23	0.04	0.55	23	1.8%	-0.57 [-1.16, 0.02]
Bohn-Goldbaum 2020 (H3N2-1 month)	0.61	0.29	23	0.72	0.22	23	1.8%	-0.42 [-1.00, 0.16]
Bohn-Goldbaum 2020 (B/Brisbane/60/2008-1 month)	0.49	0.28	23	0.57	0.27	23	1.8%	-0.29 [-0.87, 0.30]
Elzayat 2021-H3N2-25 wk post vaccination Ex-S	-11.28	120	10	6.85	72.87	10	1.2%	-0.17 [-1.05, 0.70]
Elzayat 2021-H3N2-6 wk post vaccination Ex-Op	-0.24	2.42	9	6.61	66.55	10	1.2%	-0.14 [-1.04, 0.77]
Elzayat 2021-H3N2-25 wk post vaccination Ex-Op	-0.19	1.91	9	6.85	68.97	10	1.2%	-0.13 [-1.04, 0.77]
Elzayat 2021-H3N2-6 wk post vaccination Ex-S	-0.04	0.43	10	6.61	70.32	10	1.2%	-0.13 [-1.01, 0.75]
Elzayat 2021-H1N1-6 wk post vaccination Ex-Op	0.01	0.11	9	7.21	81.93	10	1.2%	-0.12 [-1.02, 0.79]
Bohn-Goldbaum 2020 (H1N1)	-0.58	0.75	23	-0.53	0.78	23	1.8%	-0.06 [-0.64, 0.51]
Ranadive 2014 (B/Brisbane-4 week)	0.24	0.64	28	0.28	0.81	27	1.9%	-0.05 [-0.58, 0.47]
Elzayat 2021-H1N1-25 wk post vaccination Ex-Op	2.61	29.66	9	4.86	55.23	10	1.2%	-0.05 [-0.95, 0.85]
Ranadive 2014 (H3N2-4 week)	0.58	0.51	28	0.6	0.43	27	1.9%	-0.04 [-0.57, 0.49]
Elzayat 2021-B-6 wk post vaccination Ex-Op	0.02	0.5	9	0.07	1.74	10	1.2%	-0.04 [-0.94, 0.86]
Elzayat 2021-B-6 wk post vaccination Ex-S	1.02	26.76	10	0.07	1.84	10	1.2%	0.05 [-0.83, 0.92]
Elzayat 2021-H1N1-6 wk post vaccination Ex-S	14.22	170.73	10	7.3	87.64	10	1.2%	0.05 [-0.83, 0.93]
Elzayat 2021-B-25 wk post vaccination Ex-S	0.04	1.05	10	-1	26.24	10	1.2%	0.05 [-0.82, 0.93]
Elzayat 2021-H1N1-25 wk post vaccination Ex-S	10.28	123.42	10	4.86	58.35	10	1.2%	0.05 [-0.82, 0.93]
Elzayat 2021-B-25 wk post vaccination Ex-Op	0.04	0.99	9	-1	24.84	10	1.2%	0.05 [-0.85, 0.96]
Bohn-Goldbaum 2020 (B/Brisbane/60/2008)	0.13	0.39	23	0.03	0.5	23	1.8%	0.22 [-0.36, 0.80]
Long 2012 (A/Perth/16/2009-old)	-0.47	0.47	30	-0.66	0.45	30	2.0%	0.41 [-0.10, 0.92]
Ranadive 2014 (H1N1-4 week)	0.56	0.52	28	0.3	0.53	27	1.9%	0.49 [-0.05, 1.03]
Long 2012 (B/Brisbane/60/2008-old)	-0.2	0.49	30	-0.69	0.63	30	1.9%	0.86 [0.33, 1.39]
**Subtotal (95% CI)**			**426**			**429**	**37.2%**	**-0.06 [-0.24, 0.12]**
Heterogeneity: Tau² = 0.07; Chi² = 37.00, df = 23 (P = 0.03); I² = 38%								
Test for overall effect: Z = 0.65 (P = 0.52)								
**Total (95% CI)**			**1566**			**1224**	**100.0%**	**-0.05 [-0.18, 0.08]**
Heterogeneity: Tau² = 0.15; Chi² = 156.18, df = 62 (P < 0.00001); I² = 60%								
Test for overall effect: Z = 0.79 (P = 0.43)								
Test for subgroup differences: Chi² = 0.01, df = 1 (P = 0.92), I² = 0%								

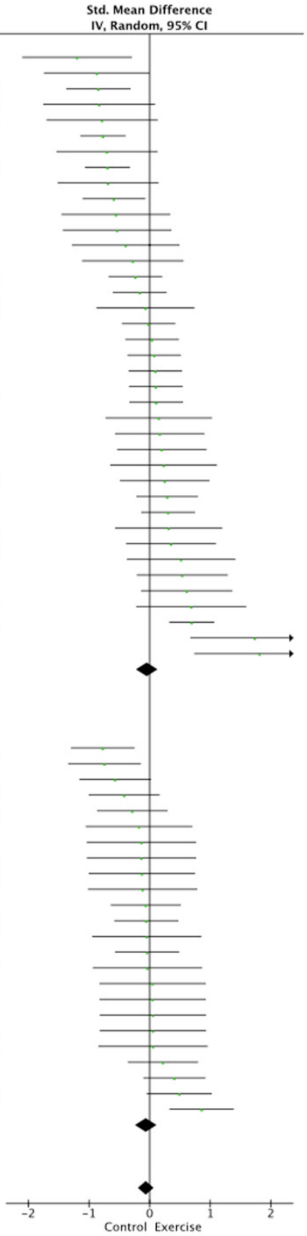

Supplementary Figure S6: Forest plot of the effect of acute exercise on influenza vaccine antibodies (subgroup analysis for age). Δ scores: post intervention – baseline; SD: standard deviation; 95% CI: 95% confidence interval

Study or Subgroup	Exercise Δ scores Mean	SD	Total	Control Δ scores Mean	SD	Total	Weight	Std. Mean Difference IV, Random, 95% CI
**1.6.1 <4 weeks**								
Long 2012 (A/Perth/16/2009-young)	-0.82	0.5	30	-0.47	0.29	30	1.9%	-0.85 [-1.37, -0.32]
Long 2012 (A/California/7/2009-old)	-0.61	0.62	30	-0.21	0.37	30	2.0%	-0.77 [-1.30, -0.25]
Campbell 2010 (A/Solomon)	-2.465	4.4486	116	0.52	0.19	39	2.3%	-0.77 [-1.14, -0.40]
Bohn-Goldbaum 2020 (H1N1-1 month)	0.38	0.39	23	0.59	0.04	23	1.8%	-0.74 [-1.34, -0.15]
Campbell 2010 (B/Malaysia)	0.3189	0.2469	116	0.48	0.17	39	2.3%	-0.70 [-1.07, -0.33]
Long 2012 (A/California/7/2009-young)	-0.14	0.5	30	0.1	0.27	30	2.0%	-0.59 [-1.11, -0.07]
Bohn-Goldbaum 2020 (H3N2)	-0.34	0.75	23	0.04	0.55	23	1.8%	-0.57 [-1.16, 0.02]
Edwards 2006 (A/Panama/2007/99-4 week-male)	-0.67	0.69	10	-0.32	0.5	10	1.2%	-0.56 [-1.45, 0.34]
Edwards 2006 (B/Shangdong/7/97-4 week-male)	0.2	0.24	10	0.31	0.14	10	1.2%	-0.54 [-1.43, 0.36]
Bohn-Goldbaum 2020 (H3N2-1 month)	0.61	0.29	23	0.72	0.22	23	1.8%	-0.42 [-1.00, 0.16]
Edwards 2006 (A/New Caledonia/20/99-4 week-male)	0.58	0.18	10	0.64	0.1	10	1.2%	-0.39 [-1.28, 0.49]
Bohn-Goldbaum 2020 (B/Brisbane/60/2008-1 month)	0.49	0.28	23	0.57	0.27	23	1.8%	-0.29 [-0.87, 0.30]
Edwards 2010 (B/Florida-28th day-exercise 85%)	633	985.94	40	832	662.58	39	2.2%	-0.23 [-0.68, 0.21]
Edwards 2010 (A/Uruguay-28th day-exercise 110%)	47	172.52	39	87	296.45	39	2.1%	-0.16 [-0.61, 0.28]
Bohn-Goldbaum 2020 (H1N1)	-0.58	0.75	23	-0.53	0.78	23	1.8%	-0.06 [-0.64, 0.51]
Ranadive 2014 (B/Brisbane-4 week)	0.24	0.64	28	0.28	0.81	27	1.9%	-0.05 [-0.58, 0.47]
Ranadive 2014 (H3N2-4 week)	0.58	0.51	28	0.6	0.43	27	1.9%	-0.04 [-0.57, 0.49]
Edwards 2010 (A/Brisbane-28th day-exercise 85%)	304	753.28	40	317	736.1	39	2.2%	-0.02 [-0.46, 0.42]
Edwards 2010 (A/Uruguay-28th day-exercise 60%)	99	297.19	40	87	296.45	39	2.2%	0.04 [-0.40, 0.48]
Edwards 2010 (A/Uruguay-28th day-exercise 85%)	114	399.84	40	87	296.45	39	2.2%	0.08 [-0.37, 0.52]
Edwards 2010 (A/Brisbane-28th day-exercise 60%)	403	1,036.04	40	317	736.1	39	2.2%	0.09 [-0.35, 0.54]
Edwards 2010 (B/Florida-28th day-exercise 110%)	950	1,467.7	39	832	662.58	39	2.1%	0.10 [-0.34, 0.55]
Edwards 2010 (A/Brisbane-28th day-exercise 110%)	425	1,181.5	39	317	736.1	39	2.1%	0.11 [-0.34, 0.55]
Edwards 2006 (B/Shangdong/7/97-4 week-female)	0.18	0.25	10	0.13	0.37	10	1.2%	0.15 [-0.73, 1.03]
Bohn-Goldbaum 2020 (B/Brisbane/60/2008)	0.13	0.39	23	0.03	0.5	23	1.8%	0.22 [-0.36, 0.80]
Long 2012 (B/Brisbane/60/2008-young)	0.11	0.22	30	0.01	0.43	30	2.0%	0.29 [-0.22, 0.80]
Edwards 2010 (B/Florida-28th day-exercise 60%)	1,236	1,722.2	40	832	662.58	39	2.2%	0.31 [-0.14, 0.75]
Long 2012 (A/Perth/16/2009-old)	-0.47	0.47	30	-0.66	0.45	30	2.0%	0.41 [-0.10, 0.92]
Ranadive 2014 (H1N1-4 week)	0.56	0.52	28	0.3	0.53	27	1.9%	0.49 [-0.05, 1.03]
Edwards 2006 (A/New Caledonia/20/99-4 week-female)	0.59	0.18	10	0.45	0.21	10	1.2%	0.69 [-0.22, 1.59]
Campbell 2010 (A/Wisconsin)	0.2568	0.2795	116	0.06	0.29	39	2.3%	0.69 [0.32, 1.07]
Long 2012 (B/Brisbane/60/2008-old)	-0.2	0.49	30	-0.69	0.63	30	1.9%	0.86 [0.33, 1.39]
Edwards 2006 (A/Panama/2007/99-4 week-female)	-0.98	0.47	10	-3.77	2.03	10	1.0%	1.81 [0.74, 2.89]
**Subtotal (95% CI)**			**1167**			**927**	**61.8%**	**-0.06 [-0.24, 0.11]**
Heterogeneity: $Tau^2 = 0.18$; $Chi^2 = 114.52$, df = 32 (P < 0.00001); $I^2 = 72\%$								
Test for overall effect: Z = 0.72 (P = 0.47)								
**1.6.2 >4 weeks**								
Edwards 2007 (H3N2-6th week-male)	349.48	200.6	18	653.86	332.12	8	1.2%	-1.20 [-2.10, -0.29]
Edwards 2007 (B/Shanghai-6th week-male)	158.43	114.84	18	390.54	442.59	8	1.2%	-0.87 [-1.74, -0.00]
Edwards 2006 (B/Shangdong/7/97-20 week-male)	-0.06	0.32	10	0.16	0.16	10	1.2%	-0.83 [-1.76, 0.09]
Edwards 2006 (A/Panama/2007/99-20th week-male)	-1.23	0.88	10	-0.66	0.44	10	1.2%	-0.78 [-1.70, 0.13]
Edwards 2007 (H3N2-20th week-male)	253.67	149.56	17	462.24	449.86	9	1.3%	-0.70 [-1.54, 0.13]
Edwards 2007 (B/Shanghai-20th week-male)	78.71	53.85	17	251.8	416.33	9	1.3%	-0.69 [-1.52, 0.15]
Edwards 2007 (H1N1-6th week-male)	1,912.89	2,559.28	18	2,855.65	4,588.75	8	1.3%	-0.28 [-1.11, 0.56]
Elzayat 2021-H3N2-25 wk post vaccination Ex-S	-11.28	120	10	6.85	72.87	10	1.2%	-0.17 [-1.05, 0.70]
Elzayat 2021-H3N2-6 wk post vaccination Ex-Op	-0.24	2.42	9	6.61	66.55	10	1.2%	-0.14 [-1.04, 0.77]
Elzayat 2021-H3N2-25 wk post vaccination Ex-Op	-0.19	1.91	9	6.85	68.97	10	1.2%	-0.13 [-1.04, 0.77]
Elzayat 2021-H3N2-6 wk post vaccination Ex-S	-0.04	0.43	10	6.61	70.32	10	1.2%	-0.13 [-1.01, 0.75]
Elzayat 2021-H1N1-6 wk post vaccination Ex-Op	0.01	0.11	9	7.21	81.93	10	1.2%	-0.12 [-1.02, 0.79]
Edwards 2007 (H1N1-20th week-male)	1,158.68	1,481.81	17	1,279.89	2,080.88	9	1.4%	-0.07 [-0.88, 0.74]
Elzayat 2021-H1N1-25 wk post vaccination Ex-Op	2.61	29.66	9	4.86	55.23	10	1.2%	-0.05 [-0.95, 0.85]
Elzayat 2021-B-6 wk post vaccination Ex-Op	0.02	0.5	9	0.07	1.74	10	1.2%	-0.04 [-0.94, 0.86]
Elzayat 2021-B-6 wk post vaccination Ex-S	1.02	26.76	10	0.07	1.84	10	1.2%	0.05 [-0.83, 0.92]
Elzayat 2021-H1N1-6 wk post vaccination Ex-S	14.22	170.73	10	7.3	87.64	10	1.2%	0.05 [-0.83, 0.93]
Elzayat 2021-B-25 wk post vaccination Ex-S	0.04	1.05	10	-1	26.24	10	1.2%	0.05 [-0.82, 0.93]
Elzayat 2021-H1N1-25 wk post vaccination Ex-S	10.28	123.42	10	4.86	58.35	10	1.2%	0.05 [-0.82, 0.93]
Elzayat 2021-B-25 wk post vaccination Ex-Op	0.04	0.99	9	-1	24.84	10	1.2%	0.05 [-0.85, 0.96]
Edwards 2007 (H3N2-6th week-female)	678.21	725.69	20	573.05	311.42	11	1.5%	0.17 [-0.57, 0.90]
Edwards 2007 (B/Shanghai-6th week-female)	412.17	773.5	20	274.53	396.68	11	1.5%	0.20 [-0.54, 0.94]
Edwards 2006 (A/New Caledonia/20/99-20 week-male)	0.47	0.14	10	0.43	0.19	10	1.2%	0.23 [-0.65, 1.11]
Edwards 2007 (H1N1-6th week-female)	1,549.25	4,321.44	20	646.9	787.86	11	1.5%	0.25 [-0.49, 0.99]
Edwards 2006 (B/Shangdong/7/97-20 week-female)	-0.06	0.34	10	-0.2	0.5	10	1.2%	0.31 [-0.57, 1.20]
Edwards 2007 (B/Shanghai-20th week-female)	202	232.51	20	133.82	21.42	11	1.5%	0.35 [-0.39, 1.09]
Edwards 2006 (A/New Caledonia/20/99-20 week-femal)	0.37	0.19	10	0.19	0.43	10	1.2%	0.52 [-0.38, 1.41]
Edwards 2007 (H1N1-20th week-female)	768.11	845.51	20	364.07	438.14	11	1.5%	0.54 [-0.21, 1.29]
Edwards 2007 (H3N2-20th week-female)	450.67	312.2	20	279.63	173.39	11	1.5%	0.61 [-0.14, 1.36]
Edwards 2006 (A/Panama/2007/99-20th week-female)	-1.53	0.71	10	-3.7	1.54	10	1.0%	1.73 [0.67, 2.80]
**Subtotal (95% CI)**			**399**			**297**	**38.2%**	**-0.03 [-0.22, 0.16]**
Heterogeneity: $Tau^2 = 0.08$; $Chi^2 = 41.32$, df = 29 (P = 0.06); $I^2 = 30\%$								
Test for overall effect: Z = 0.31 (P = 0.76)								
**Total (95% CI)**			**1566**			**1224**	**100.0%**	**-0.05 [-0.18, 0.08]**
Heterogeneity: $Tau^2 = 0.15$; $Chi^2 = 156.18$, df = 62 (P < 0.00001); $I^2 = 60\%$								
Test for overall effect: Z = 0.79 (P = 0.43)								
Test for subgroup differences: $Chi^2 = 0.07$, df = 1 (P = 0.79), $I^2 = 0\%$								

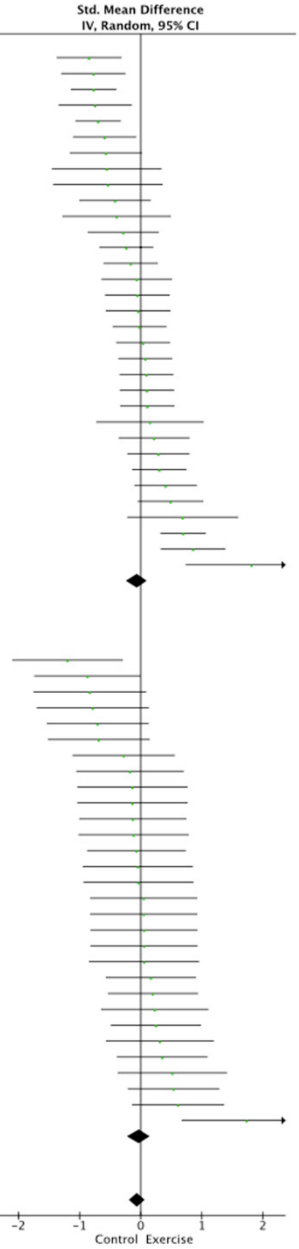

Supplementary Figure S7: Forest plot of the effect of acute exercise on influenza vaccine antibodies (subgroup analysis for measurements of <4 weeks vs. >4 weeks from vaccination). Δ scores: post intervention – baseline; SD: standard deviation; 95% CI: 95% confidence interval.

Study or Subgroup	Exercise Δ scores Mean	SD	Total	Control Δ scores Mean	SD	Total	Weight	Std. Mean Difference IV, Random, 95% CI
**1.8.1 Aerobic**								
Long 2012 (Pneumococcal-IgM young)	−4.5	2.33	30	−0.68	0.55	30	2.7%	−2.23 [−2.88, −1.57]
Long 2012 (Pneumococcal-IgM old)	−0.77	0.43	30	−0.7	0.69	30	3.6%	−0.12 [−0.63, 0.39]
Bruunsgaard 1997 (Pneumococcal 2)	90	87.26	22	76.34	54.4	22	3.0%	0.18 [−0.41, 0.78]
Long 2012 (Pneumococcal-IgG young)	−0.4	0.42	30	−0.55	0.41	30	3.6%	0.36 [−0.15, 0.87]
Long 2012 (Pneumococcal-IgG old)	−0.65	0.31	30	−0.99	0.84	30	3.5%	0.53 [0.01, 1.05]
Bruunsgaard 1997 (Pneumococcal 1)	90	87.26	22	35.33	36.18	11	2.3%	0.71 [−0.03, 1.46]
**Subtotal (95% CI)**			**164**			**153**	**18.6%**	**−0.09 [−0.87, 0.69]**
Heterogeneity: $Tau^2 = 0.85$; $Chi^2 = 54.71$, df = 5 (P < 0.00001); $I^2 = 91\%$								
Test for overall effect: Z = 0.23 (P = 0.82)								
**1.8.2 Resistance**								
Edwards 2012 (Pneumococcal-18C-28 day-half dose)	6.14	98.67	32	8.43	194.33	31	3.7%	−0.01 [−0.51, 0.48]
Edwards 2012 (Pneumococcal-4-28 day-full dose)	0.54	5.56	30	0.61	5.04	35	3.7%	−0.01 [−0.50, 0.47]
Edwards 2012 (Pneumococcal-23F-28 day-full dose)	0.53	47.66	30	0.99	26.21	35	3.7%	−0.01 [−0.50, 0.48]
Edwards 2012 (Pneumococcal-18C-28 day-full dose)	4.72	115.45	30	6.48	187.86	35	3.7%	−0.01 [−0.50, 0.48]
Edwards 2012 (Pneumococcal-14-28 day-half dose)	1.41	37.81	32	1.6	36.77	31	3.7%	−0.01 [−0.50, 0.49]
Edwards 2012 (Pneumococcal-23F-28 day-half dose)	0.93	26.46	32	0.84	23.06	31	3.7%	0.00 [−0.49, 0.50]
Edwards 2012 (Pneumococcal-7F-28 day-full dose)	2.59	26.41	30	2.49	28.6	35	3.7%	0.00 [−0.48, 0.49]
Edwards 2012 (Pneumococcal-1-28 day-full dose)	20.07	337.69	30	16.57	336.56	35	3.7%	0.01 [−0.48, 0.50]
Edwards 2012 (Pneumococcal-19A-28 day-half dose)	3.26	30.5	32	2.92	27.27	31	3.7%	0.01 [−0.48, 0.51]
Edwards 2012 (Pneumococcal-14-28 day-full dose)	2.43	53.49	30	1.92	29.29	35	3.7%	0.01 [−0.48, 0.50]
Edwards 2012 (Pneumococcal-7F-28 day-half dose)	2.76	33.92	32	2.33	17.41	31	3.7%	0.02 [−0.48, 0.51]
Edwards 2012 (Pneumococcal-6B-28 day-full dose)	2.08	73.63	30	1.09	21.15	35	3.7%	0.02 [−0.47, 0.51]
Edwards 2012 (Pneumococcal-9V-28 day-full dose)	1.58	40.52	30	0.9	22.28	35	3.7%	0.02 [−0.47, 0.51]
Edwards 2012 (Pneumococcal-6B-28 day-half dose)	2.03	60.83	32	0.97	26.34	31	3.7%	0.02 [−0.47, 0.52]
Edwards 2012 (Pneumococcal-3-28 day-full dose)	2.19	70.06	30	0.67	14.45	35	3.7%	0.03 [−0.46, 0.52]
Edwards 2012 (Pneumococcal-9V-28 day-half dose)	1.26	29.36	32	0.54	8.42	31	3.7%	0.03 [−0.46, 0.53]
Edwards 2012 (Pneumococcal-5-28 day-half dose)	10.32	257.02	32	3.75	71.16	31	3.7%	0.03 [−0.46, 0.53]
Edwards 2012 (Pneumococcal-3-28 day-half dose)	1.64	39.04	32	0.6	12.15	31	3.7%	0.04 [−0.46, 0.53]
Edwards 2012 (Pneumococcal-19A-28 day-full dose)	5.65	40.55	30	3.89	51.88	35	3.7%	0.04 [−0.45, 0.52]
Edwards 2012 (Pneumococcal-1-28 day-half dose)	17.5	377.71	32	6.95	94.3	31	3.7%	0.04 [−0.46, 0.53]
Edwards 2012 (Pneumococcal-5-28 day-full dose)	9.46	121.82	35	4.64	83.49	35	3.9%	0.05 [−0.42, 0.51]
Edwards 2012 (Pneumococcal-4-28 day-half dose)	0.62	5.02	32	0.4	3.32	31	3.7%	0.05 [−0.44, 0.54]
**Subtotal (95% CI)**			**687**			**726**	**81.4%**	**0.02 [−0.09, 0.12]**
Heterogeneity: $Tau^2 = 0.00$; $Chi^2 = 0.13$, df = 21 (P = 1.00); $I^2 = 0\%$								
Test for overall effect: Z = 0.31 (P = 0.75)								
**Total (95% CI)**			**851**			**879**	**100.0%**	**0.00 [−0.13, 0.14]**
Heterogeneity: $Tau^2 = 0.07$; $Chi^2 = 54.96$, df = 27 (P = 0.001); $I^2 = 51\%$								
Test for overall effect: Z = 0.03 (P = 0.97)								
Test for subgroup differences: $Chi^2 = 0.07$, df = 1 (P = 0.79), $I^2 = 0\%$								

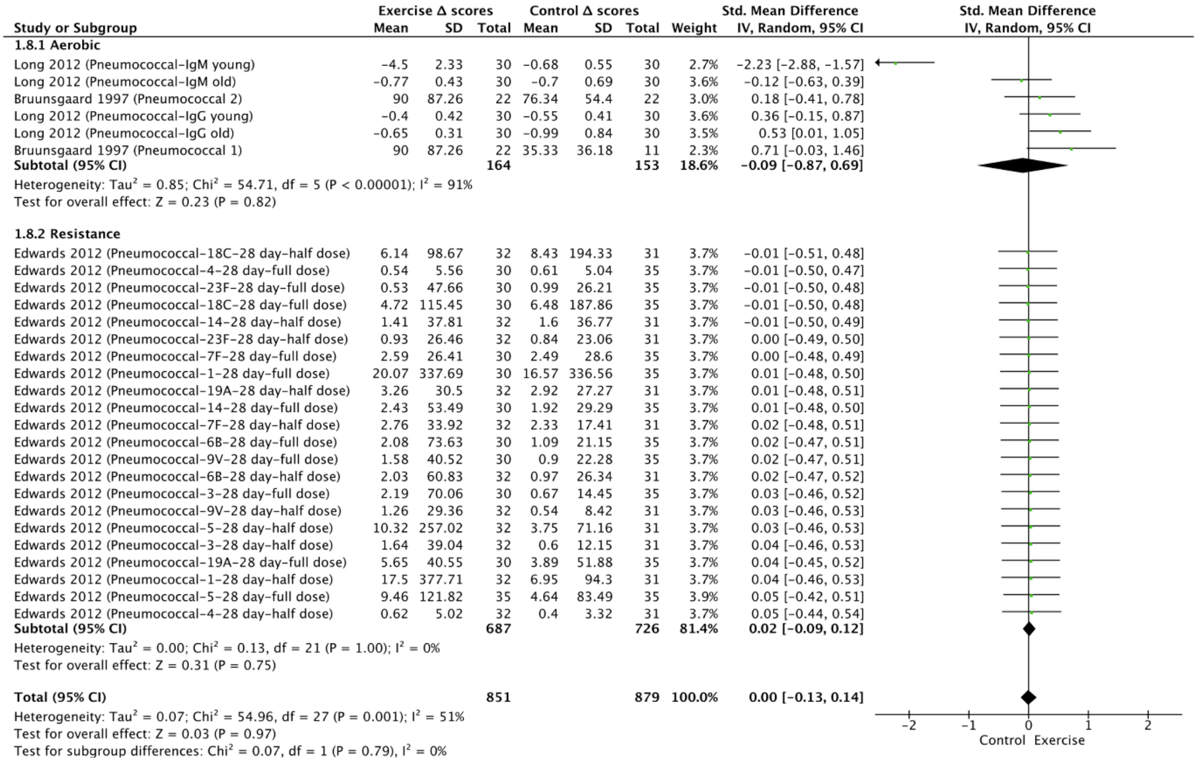

Supplementary Figure S8: Forest plot of the effect of acute exercise on pneumococcal vaccine antibodies (subgroup analysis for exercise type). Δ scores: post intervention – baseline; SD: standard deviation; 95% CI: 95% confidence interval.

Study or Subgroup	Exercise Δ scores Mean	SD	Total	Control Δ scores Mean	SD	Total	Weight	Std. Mean Difference IV, Random, 95% CI
**1.9.1 Low <65% HR 1 RM**								
Long 2012 (Pneumococcal-IgM young)	−4.5	2.33	30	−0.68	0.55	30	2.7%	−2.23 [−2.88, −1.57]
Long 2012 (Pneumococcal-IgM old)	−0.77	0.43	30	−0.7	0.69	30	3.6%	−0.12 [−0.63, 0.39]
Long 2012 (Pneumococcal-IgG young)	−0.4	0.42	30	−0.55	0.41	30	3.6%	0.36 [−0.15, 0.87]
Long 2012 (Pneumococcal-IgG old)	−0.65	0.31	30	−0.99	0.84	30	3.5%	0.53 [0.01, 1.05]
**Subtotal (95% CI)**			**120**			**120**	**13.4%**	**−0.35 [−1.45, 0.75]**
Heterogeneity: $Tau^2 = 1.18$; $Chi^2 = 49.37$, df = 3 ($P < 0.00001$); $I^2 = 94\%$								
Test for overall effect: Z = 0.62 (P = 0.53)								
**1.9.2 Moderate 65–75% HR 1 RM**								
Edwards 2012 (Pneumococcal-18C-28 day-half dose)	6.14	98.67	32	8.43	194.33	31	3.7%	−0.01 [−0.51, 0.48]
Edwards 2012 (Pneumococcal-4-28 day-full dose)	0.54	5.56	30	0.61	5.04	35	3.7%	−0.01 [−0.50, 0.47]
Edwards 2012 (Pneumococcal-23F-28 day-full dose)	0.53	47.66	30	0.99	26.21	35	3.7%	−0.01 [−0.50, 0.48]
Edwards 2012 (Pneumococcal-18C-28 day-full dose)	4.72	115.45	30	6.48	187.86	35	3.7%	−0.01 [−0.50, 0.48]
Edwards 2012 (Pneumococcal-14-28 day-half dose)	1.41	37.81	32	1.6	36.77	31	3.7%	−0.01 [−0.50, 0.49]
Edwards 2012 (Pneumococcal-23F-28 day-half dose)	0.93	26.46	32	0.84	23.06	31	3.7%	0.00 [−0.49, 0.50]
Edwards 2012 (Pneumococcal-7F-28 day-full dose)	2.59	26.41	30	2.49	28.6	35	3.7%	0.00 [−0.48, 0.49]
Edwards 2012 (Pneumococcal-1-28 day-full dose)	20.07	337.69	30	16.57	336.56	35	3.7%	0.01 [−0.48, 0.50]
Edwards 2012 (Pneumococcal-19A-28 day-half dose)	3.26	30.5	32	2.92	27.27	31	3.7%	0.01 [−0.48, 0.51]
Edwards 2012 (Pneumococcal-14-28 day-full dose)	2.43	53.49	30	1.92	29.29	35	3.7%	0.01 [−0.48, 0.50]
Edwards 2012 (Pneumococcal-7F-28 day-half dose)	2.76	33.92	32	2.33	17.41	31	3.7%	0.02 [−0.48, 0.51]
Edwards 2012 (Pneumococcal-6B-28 day-full dose)	2.08	73.63	30	1.09	21.15	35	3.7%	0.02 [−0.47, 0.51]
Edwards 2012 (Pneumococcal-9V-28 day-full dose)	1.58	40.52	30	0.9	22.28	35	3.7%	0.02 [−0.47, 0.51]
Edwards 2012 (Pneumococcal-6B-28 day-half dose)	2.03	60.83	32	0.97	26.34	31	3.7%	0.02 [−0.47, 0.52]
Edwards 2012 (Pneumococcal-3-28 day-full dose)	2.19	70.06	30	0.67	14.45	35	3.7%	0.03 [−0.46, 0.52]
Edwards 2012 (Pneumococcal-9V-28 day-half dose)	1.26	29.36	32	0.54	8.42	31	3.7%	0.03 [−0.46, 0.53]
Edwards 2012 (Pneumococcal-5-28 day-half dose)	10.32	257.02	32	3.75	71.16	31	3.7%	0.03 [−0.46, 0.53]
Edwards 2012 (Pneumococcal-3-28 day-half dose)	1.64	39.04	32	0.6	12.15	31	3.7%	0.04 [−0.46, 0.53]
Edwards 2012 (Pneumococcal-19A-28 day-full dose)	5.65	40.55	30	3.89	51.88	35	3.7%	0.04 [−0.45, 0.52]
Edwards 2012 (Pneumococcal-1-28 day-half dose)	17.5	377.71	32	6.95	94.3	31	3.7%	0.04 [−0.46, 0.53]
Edwards 2012 (Pneumococcal-5-28 day-full dose)	9.46	121.82	35	4.64	83.49	35	3.9%	0.05 [−0.42, 0.51]
Edwards 2012 (Pneumococcal-4-28 day-half dose)	0.62	5.02	32	0.4	3.32	31	3.7%	0.05 [−0.44, 0.54]
**Subtotal (95% CI)**			**687**			**726**	**81.4%**	**0.02 [−0.09, 0.12]**
Heterogeneity: $Tau^2 = 0.00$; $Chi^2 = 0.13$, df = 21 ($P = 1.00$); $I^2 = 0\%$								
Test for overall effect: Z = 0.31 (P = 0.75)								
**1.9.3 High >75% HR 1 RM**								
Bruunsgaard 1997 (Pneumococcal 2)	90	87.26	22	76.34	54.4	22	3.0%	0.18 [−0.41, 0.78]
Bruunsgaard 1997 (Pneumococcal 1)	90	87.26	22	35.33	36.18	11	2.3%	0.71 [−0.03, 1.46]
**Subtotal (95% CI)**			**44**			**33**	**5.3%**	**0.40 [−0.11, 0.91]**
Heterogeneity: $Tau^2 = 0.02$; $Chi^2 = 1.19$, df = 1 ($P = 0.28$); $I^2 = 16\%$								
Test for overall effect: Z = 1.53 (P = 0.13)								
**Total (95% CI)**			**851**			**879**	**100.0%**	**0.00 [−0.13, 0.14]**
Heterogeneity: $Tau^2 = 0.07$; $Chi^2 = 54.96$, df = 27 ($P = 0.001$); $I^2 = 51\%$								
Test for overall effect: Z = 0.03 (P = 0.97)								
Test for subgroup differences: $Chi^2 = 2.53$, df = 2 ($P = 0.28$), $I^2 = 21.0\%$								

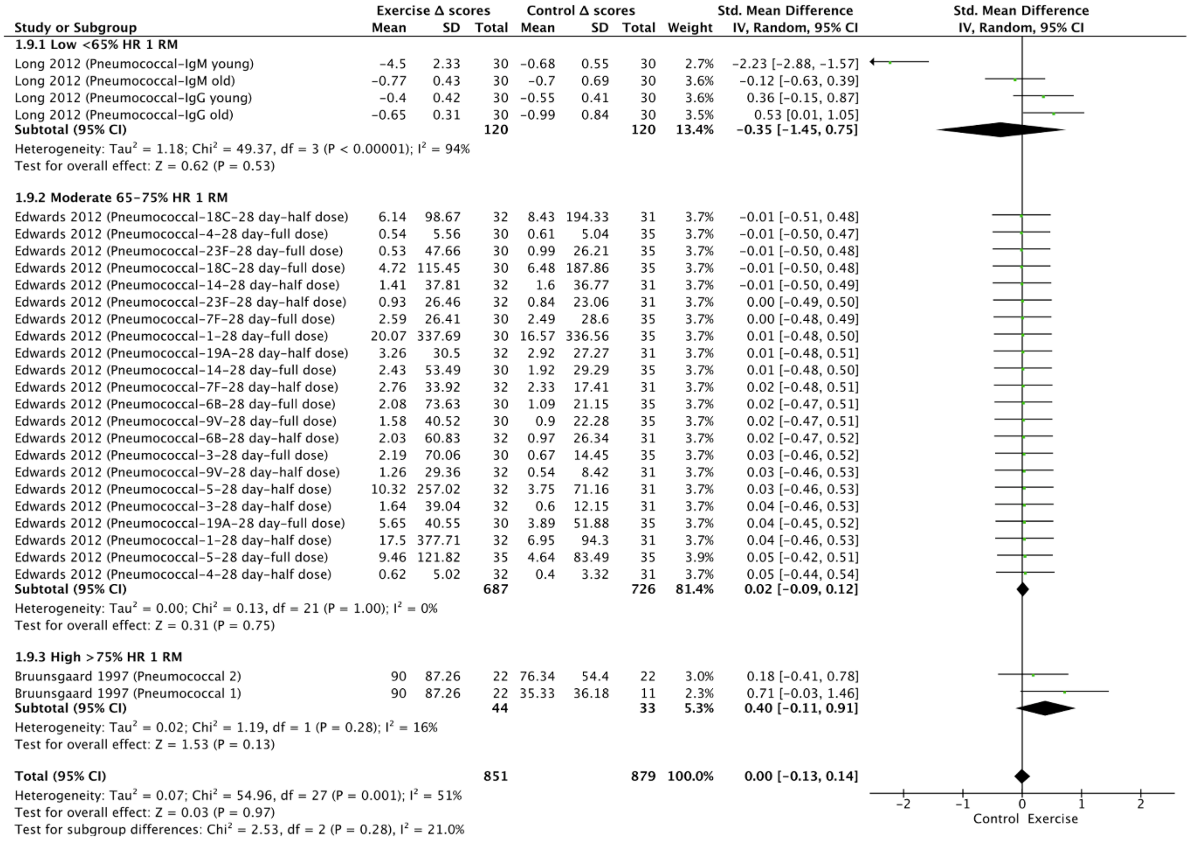

Supplementary Figure S9: Forest plot of the effect of acute exercise on pneumococcal vaccine antibodies (subgroup analysis for exercise intensity). Δ scores: post intervention – baseline; SD: standard deviation; 95% CI: 95% confidence interval.

Study or Subgroup	Exercise Δ scores Mean	SD	Total	Control Δ scores Mean	SD	Total	Weight	Std. Mean Difference IV, Random, 95% CI
**1.10.1 < 31 years**								
Long 2012 (Pneumococcal-IgM young)	-4.5	2.33	30	-0.68	0.55	30	2.7%	-2.23 [-2.88, -1.57]
Edwards 2012 (Pneumococcal-18C-28 day-half dose)	6.14	98.67	32	8.43	194.33	31	3.7%	-0.01 [-0.51, 0.48]
Edwards 2012 (Pneumococcal-4-28 day-full dose)	0.54	5.56	30	0.61	5.04	35	3.7%	-0.01 [-0.50, 0.47]
Edwards 2012 (Pneumococcal-23F-28 day-full dose)	0.53	47.66	30	0.99	26.21	35	3.7%	-0.01 [-0.50, 0.48]
Edwards 2012 (Pneumococcal-18C-28 day-full dose)	4.72	115.45	30	6.48	187.86	35	3.7%	-0.01 [-0.50, 0.48]
Edwards 2012 (Pneumococcal-14-28 day-half dose)	1.41	37.81	32	1.6	36.77	31	3.7%	-0.01 [-0.50, 0.49]
Edwards 2012 (Pneumococcal-23F-28 day-half dose)	0.93	26.46	32	0.84	23.06	31	3.7%	0.00 [-0.49, 0.50]
Edwards 2012 (Pneumococcal-7F-28 day-full dose)	2.59	26.41	30	2.49	28.6	35	3.7%	0.00 [-0.48, 0.49]
Edwards 2012 (Pneumococcal-1-28 day-full dose)	20.07	337.69	30	16.57	336.56	35	3.7%	0.01 [-0.48, 0.50]
Edwards 2012 (Pneumococcal-19A-28 day-half dose)	3.26	30.5	32	2.92	27.27	31	3.7%	0.01 [-0.48, 0.51]
Edwards 2012 (Pneumococcal-14-28 day-full dose)	2.43	53.49	30	1.92	29.29	35	3.7%	0.01 [-0.48, 0.50]
Edwards 2012 (Pneumococcal-7F-28 day-half dose)	2.76	33.92	32	2.33	17.41	31	3.7%	0.02 [-0.48, 0.51]
Edwards 2012 (Pneumococcal-6B-28 day-full dose)	2.08	73.63	30	1.09	21.15	35	3.7%	0.02 [-0.47, 0.51]
Edwards 2012 (Pneumococcal-9V-28 day-full dose)	1.58	40.52	30	0.9	22.28	35	3.7%	0.02 [-0.47, 0.51]
Edwards 2012 (Pneumococcal-6B-28 day-half dose)	2.03	60.83	32	0.97	26.34	31	3.7%	0.02 [-0.47, 0.52]
Edwards 2012 (Pneumococcal-3-28 day-full dose)	2.19	70.06	30	0.67	14.45	35	3.7%	0.03 [-0.46, 0.52]
Edwards 2012 (Pneumococcal-9V-28 day-half dose)	1.26	29.36	32	0.54	8.42	31	3.7%	0.03 [-0.46, 0.53]
Edwards 2012 (Pneumococcal-5-28 day-half dose)	10.32	257.02	32	3.75	71.16	31	3.7%	0.03 [-0.46, 0.53]
Edwards 2012 (Pneumococcal-3-28 day-half dose)	1.64	39.04	32	0.6	12.15	31	3.7%	0.04 [-0.46, 0.53]
Edwards 2012 (Pneumococcal-19A-28 day-full dose)	5.65	40.55	30	3.89	51.88	35	3.7%	0.04 [-0.45, 0.52]
Edwards 2012 (Pneumococcal-1-28 day-half dose)	17.5	377.71	32	6.95	94.3	31	3.7%	0.04 [-0.46, 0.53]
Edwards 2012 (Pneumococcal-5-28 day-full dose)	9.46	121.82	35	4.64	83.49	35	3.9%	0.05 [-0.42, 0.51]
Edwards 2012 (Pneumococcal-4-28 day-half dose)	0.62	5.02	32	0.4	3.32	31	3.7%	0.05 [-0.44, 0.54]
Bruunsgaard 1997 (Pneumococcal 2)	90	87.26	22	76.34	54.4	22	3.0%	0.18 [-0.41, 0.78]
Long 2012 (Pneumococcal-IgG young)	-0.4	0.42	30	-0.55	0.41	30	3.6%	0.36 [-0.15, 0.87]
Bruunsgaard 1997 (Pneumococcal 1)	90	87.26	22	35.33	36.18	11	2.3%	0.71 [-0.03, 1.46]
**Subtotal (95% CI)**			**791**			**819**	**92.9%**	**-0.01 [-0.15, 0.13]**
Heterogeneity: $Tau^2 = 0.07$; $Chi^2 = 50.71$, df = 25 (P = 0.002); $I^2 = 51\%$								
Test for overall effect: Z = 0.18 (P = 0.86)								
**1.10.2 = 57 years**								
Long 2012 (Pneumococcal-IgM old)	-0.77	0.43	30	-0.7	0.69	30	3.6%	-0.12 [-0.63, 0.39]
Long 2012 (Pneumococcal-IgG old)	-0.65	0.31	30	-0.99	0.84	30	3.5%	0.53 [0.01, 1.05]
**Subtotal (95% CI)**			**60**			**60**	**7.1%**	**0.20 [-0.43, 0.84]**
Heterogeneity: $Tau^2 = 0.14$; $Chi^2 = 3.11$, df = 1 (P = 0.08); $I^2 = 68\%$								
Test for overall effect: Z = 0.62 (P = 0.53)								
**Total (95% CI)**			**851**			**879**	**100.0%**	**0.00 [-0.13, 0.14]**
Heterogeneity: $Tau^2 = 0.07$; $Chi^2 = 54.96$, df = 27 (P = 0.001); $I^2 = 51\%$								
Test for overall effect: Z = 0.03 (P = 0.97)								
Test for subgroup differences: $Chi^2 = 0.42$, df = 1 (P = 0.52), $I^2 = 0\%$								

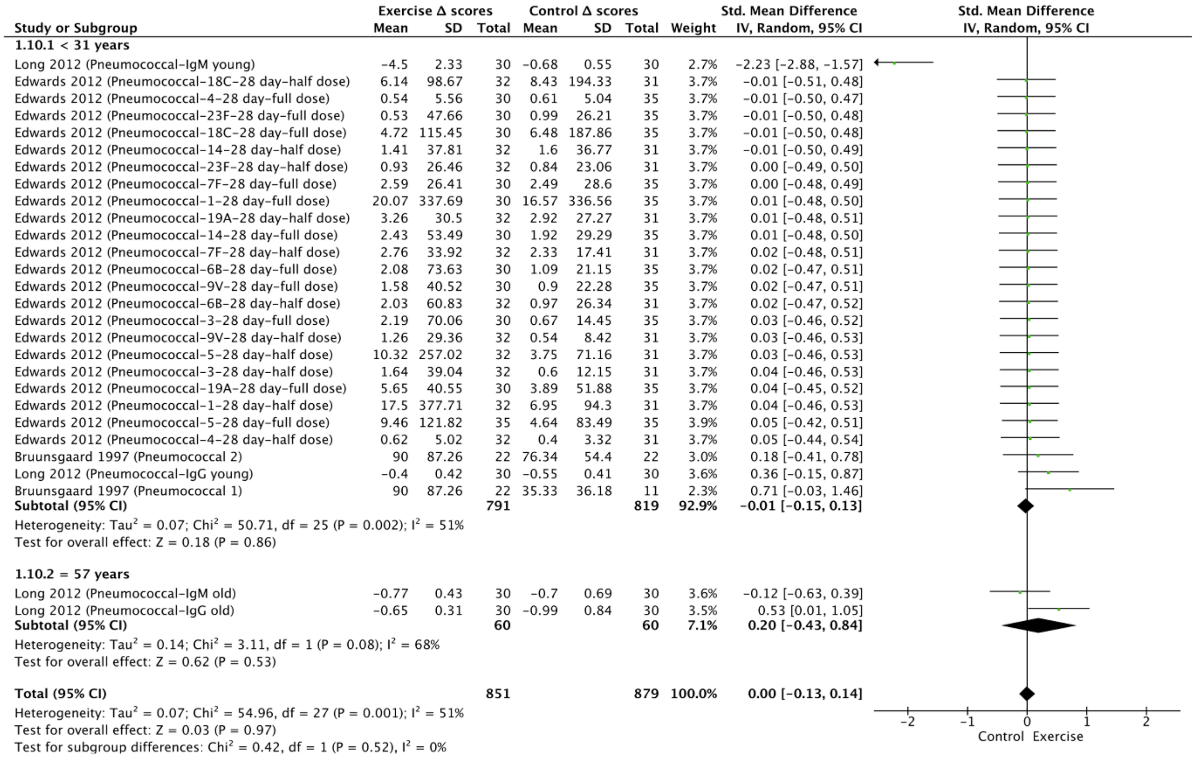

Supplementary Figure S10: Forest plot of the effect of acute exercise on pneumococcal vaccine antibodies (subgroup analysis for age). Δ scores: post intervention – baseline; SD: standard deviation; 95% CI: 95% confidence interval.

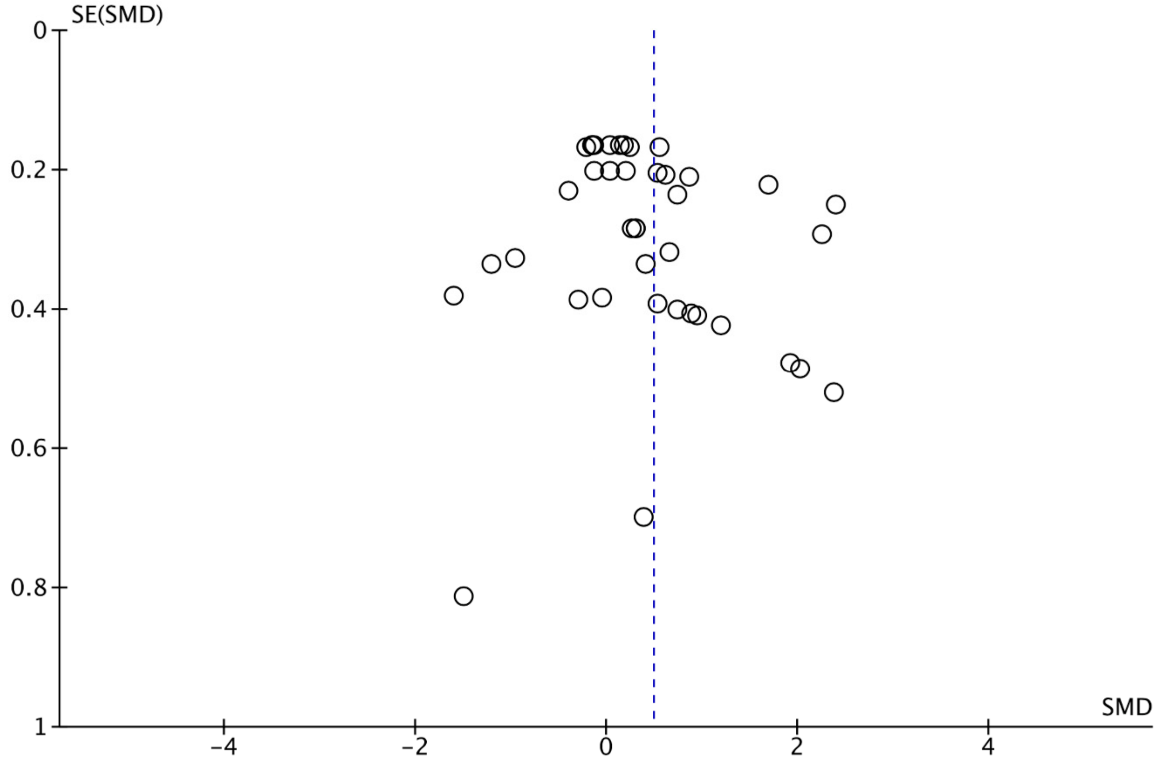

Supplementary Figure S11: Funnel plot of the effect of chronic exercise on influenza vaccine antibodies. SMD: standardized mean difference; SE: standard error

Study or Subgroup	Exercise Δ scores Mean	SD	Total	Control Δ scores Mean	SD	Total	Weight	Std. Mean Difference IV, Random, 95% CI
**2.2.1 Aerobic**								
Kohut 2005 (Panama/2007/99-H3N2-12 week)	-0.29	0.37	14	-0.18	0.39	13	2.3%	-0.28 [-1.04, 0.48]
Woods 2009 B 3 weeks	-0.3	1.2	74	-0.07	1.11	70	2.8%	-0.20 [-0.53, 0.13]
Woods 2009 B 6 weeks	-0.36	1.22	74	-0.18	1.14	70	2.8%	-0.15 [-0.48, 0.18]
Woods 2009 H1N1 6 weeks	-0.02	1.07	74	0.13	0.99	70	2.8%	-0.14 [-0.47, 0.18]
Woods 2009 H1N1 3 weeks	0.07	1.03	74	0.2	0.96	70	2.8%	-0.13 [-0.46, 0.20]
Hayney 2014 (A/Brisbane H1N1-12 week)	36.6	13.82	47	38.4	16.38	51	2.7%	-0.12 [-0.51, 0.28]
Kohut 2005 (Panama/2007/99-H3N2-4 week)	-0.25	0.71	14	-0.22	0.47	13	2.3%	-0.05 [-0.80, 0.71]
Woods 2009 B 24 weeks	-1.39	1.61	74	-1.45	1.57	70	2.8%	0.04 [-0.29, 0.36]
Hayney 2014 (B/Brisbane -12 week)	31.8	17.22	47	31	19.25	51	2.7%	0.04 [-0.35, 0.44]
Woods 2009 H1N1 24 weeks	-0.82	1.36	74	-1.03	1.4	70	2.8%	0.15 [-0.18, 0.48]
Woods 2009 H3N2 6 weeks	0.07	1.03	74	-0.13	1.18	70	2.8%	0.18 [-0.15, 0.51]
Hayney 2014 (B/Brisbane-3 week)	41	18.61	47	37.3	17.19	51	2.7%	0.21 [-0.19, 0.60]
Woods 2009 H3N2 3 weeks	0.2	0.98	74	-0.05	1.11	70	2.8%	0.24 [-0.09, 0.57]
Hayney 2014 (A/Brisbane H3N2-12 week)	121.4	7.98	47	112.2	22.72	51	2.7%	0.53 [0.12, 0.93]
Kohut 2004 (B/Yamanash/166/98-3 month)	-4.13	2.29	14	-5.48	2.56	13	2.3%	0.54 [-0.23, 1.31]
Woods 2009 H3N2 24 weeks	-0.3	1.17	74	-1.04	1.5	70	2.8%	0.55 [0.22, 0.88]
Hayney 2014 (A/Brisbane H3N2-3 week)	184.6	8.18	47	174	22.4	51	2.7%	0.61 [0.21, 1.02]
Kohut 2004 (B/Yamanashi/166/98-4 week)	-1.95	1.73	14	-3.45	2.13	13	2.2%	0.75 [-0.03, 1.54]
Hayney 2014 (A/Brisbane H1N1-3 week)	58.3	9.85	47	46	17.18	51	2.7%	0.86 [0.45, 1.28]
Kohut 2004 (H3N2-3 month)	-1.67	1.68	14	-3.42	2.12	13	2.2%	0.89 [0.09, 1.69]
Kohut 2004 (H1N1-3 month)	-2.11	1.72	14	-4.06	2.25	13	2.2%	0.95 [0.15, 1.75]
Kohut 2004 (Panama/2007/99-H3N2-4 week)	-1.11	1.53	14	-3.37	2.1	13	2.2%	1.20 [0.37, 2.03]
Kohut 2005 (Caledonia/20/99-H1N1-4 week)	-0.03	0.65	14	-1.82	1.11	13	2.0%	1.93 [0.99, 2.86]
Kohut 2004 (Caledonia/20/99-H1N1-4 week)	-0.5	0.86	14	-4.18	2.39	13	2.0%	2.02 [1.06, 2.97]
Kohut 2005 (Caledonia/20/99-H1N1-12 week)	-0.49	0.88	14	-3.94	1.81	13	1.9%	2.38 [1.36, 3.40]
**Subtotal (95% CI)**			**1088**			**1066**	**63.1%**	**0.39 [0.19, 0.58]**
Heterogeneity: Tau^2 = 0.16; Chi^2 = 103.56, df = 24 (P < 0.00001); I^2 = 77%								
Test for overall effect: Z = 3.96 (P < 0.0001)								
**2.2.2 Combined**								
Monteiro 2020 IgG L-glutamine	-4.74	1.57	26	-2.53	0.8	14	2.3%	-1.60 [-2.34, -0.85]
Monteiro 2020 IgG placebo	-3.39	1.07	27	-2.19	0.84	17	2.4%	-1.19 [-1.85, -0.53]
Monteiro 2020 IgA placebo	-4.07	1.06	27	-3.09	0.93	17	2.4%	-0.95 [-1.59, -0.31]
Felismino 2021 IgG	-3.19	1.03	49	-2.79	0.99	31	2.7%	-0.39 [-0.84, 0.06]
Monteiro 2020 IgM L-glutamine	-1.77	0.65	26	-2.06	0.76	14	2.4%	0.41 [-0.24, 1.07]
Monteiro 2020 IgM placebo	-2.29	0.58	27	-2.82	1.02	17	2.5%	0.67 [0.05, 1.29]
Felismino 2021 IgM	-1.84	0.68	49	-2.34	0.66	31	2.6%	0.74 [0.27, 1.20]
Bachi 2013 (Influenza-IgM-30 day)	-2.16	0.45	55	-3.19	0.73	55	2.7%	1.69 [1.25, 2.12]
Felismino 2021 IgA	-1.79	1.05	49	-4.6	1.48	31	2.5%	2.26 [1.68, 2.83]
Bachi 2013 (Influenza-IgG-30 day)	-1.54	0.23	55	-3.01	0.83	55	2.6%	2.40 [1.90, 2.89]
Monteiro 2020 IgA L-glutamine	1.49	0.71	26	-6.76	2.07	14	1.4%	6.03 [4.50, 7.57]
**Subtotal (95% CI)**			**416**			**296**	**26.5%**	**0.83 [-0.05, 1.72]**
Heterogeneity: Tau^2 = 2.13; Chi^2 = 255.45, df = 10 (P < 0.00001); I^2 = 96%								
Test for overall effect: Z = 1.84 (P = 0.07)								
**2.2.3 Other**								
Whitham 2003 (B/Yamanashi/166/88-14 day)	0.51	0.16	7	0.76	0.12	3	1.3%	-1.50 [-3.09, 0.10]
Yang 2007 (Influenza-6 week)	2.32	3.33	27	1.53	2.09	23	2.5%	0.27 [-0.28, 0.83]
Yang 2007 (Influenza-20 week)	2.1	4.3	27	1.1	0.82	23	2.5%	0.31 [-0.25, 0.87]
Yang 2007 (Influenza-3 week)	2.77	4.94	27	1.57	1.46	23	2.5%	0.31 [-0.25, 0.87]
Whitham 2003 (Influenza IgG-12 month)	0.45	0.15	7	0.39	0.1	3	1.5%	0.39 [-0.98, 1.76]
**Subtotal (95% CI)**			**95**			**75**	**10.4%**	**0.22 [-0.13, 0.57]**
Heterogeneity: Tau^2 = 0.03; Chi^2 = 4.74, df = 4 (P = 0.32); I^2 = 16%								
Test for overall effect: Z = 1.24 (P = 0.22)								
**Total (95% CI)**			**1599**			**1437**	**100.0%**	**0.49 [0.25, 0.73]**
Heterogeneity: Tau^2 = 0.53; Chi^2 = 389.67, df = 40 (P < 0.00001); I^2 = 90%								
Test for overall effect: Z = 3.95 (P < 0.0001)								
Test for subgroup differences: Chi^2 = 1.77, df = 2 (P = 0.41), I^2 = 0%								

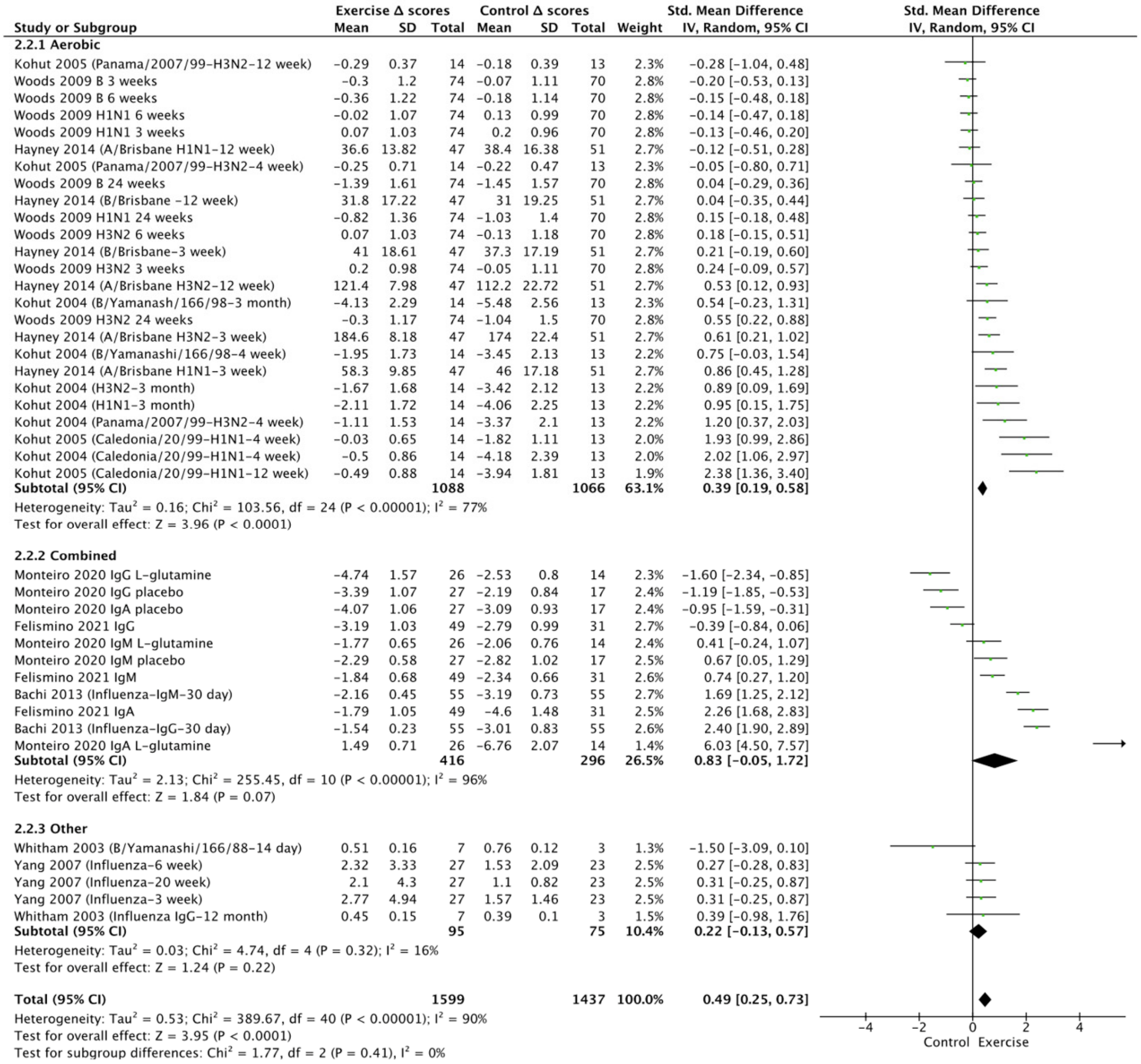

Supplementary Figure S12: Forest plot of the effects of chronic exercise on influenza vaccine antibodies (subgroup analysis for exercise type). Δ scores: post intervention – baseline; SD: standard deviation; 95% CI: 95% confidence interval

Study or Subgroup	Exercise Δ scores Mean	SD	Total	Control Δ scores Mean	SD	Total	Weight	Std. Mean Difference IV, Random, 95% CI
**2.3.1 Low <65% HR 1 RM**								
Felismino 2021 IgG	−3.19	1.03	49	−2.79	0.99	31	2.7%	−0.39 [−0.84, 0.06]
Woods 2009 B 3 weeks	−0.3	1.2	74	−0.07	1.11	70	2.8%	−0.20 [−0.53, 0.13]
Woods 2009 B 6 weeks	−0.36	1.22	74	−0.18	1.14	70	2.8%	−0.15 [−0.48, 0.18]
Woods 2009 H1N1 6 weeks	−0.02	1.07	74	0.13	0.99	70	2.8%	−0.14 [−0.47, 0.18]
Woods 2009 H1N1 3 weeks	0.07	1.03	74	0.2	0.96	70	2.8%	−0.13 [−0.46, 0.20]
Woods 2009 B 24 weeks	−1.39	1.61	74	−1.45	1.57	70	2.8%	0.04 [−0.29, 0.36]
Woods 2009 H1N1 24 weeks	−0.82	1.36	74	−1.03	1.4	70	2.8%	0.15 [−0.18, 0.48]
Woods 2009 H3N2 6 weeks	0.07	1.03	74	−0.13	1.18	70	2.8%	0.18 [−0.15, 0.51]
Woods 2009 H3N2 3 weeks	0.2	0.98	74	−0.05	1.11	70	2.8%	0.24 [−0.09, 0.57]
Woods 2009 H3N2 24 weeks	−0.3	1.17	74	−1.04	1.5	70	2.8%	0.55 [0.22, 0.88]
Felismino 2021 IgM	−1.84	0.68	49	−2.34	0.66	31	2.6%	0.74 [0.27, 1.20]
Bachi 2013 (Influenza-IgM-30 day)	−2.16	0.45	55	−3.19	0.73	55	2.7%	1.69 [1.25, 2.12]
Felismino 2021 IgA	−1.79	1.05	49	−4.6	1.48	31	2.5%	2.26 [1.68, 2.83]
Bachi 2013 (Influenza-IgG-30 day)	−1.54	0.23	55	−3.01	0.83	55	2.6%	2.40 [1.90, 2.89]
**Subtotal (95% CI)**			**923**			**833**	**38.2%**	**0.49 [0.11, 0.88]**
Heterogeneity: Tau² = 0.50; Chi² = 200.62, df = 13 (P < 0.00001); I² = 94%								
Test for overall effect: Z = 2.51 (P = 0.01)								
**2.3.2 Moderate 65–75% HR 1 RM**								
Monteiro 2020 IgG L-glutamine	−4.74	1.57	26	−2.53	0.8	14	2.3%	−1.60 [−2.34, −0.85]
Monteiro 2020 IgG placebo	−3.39	1.07	27	−2.19	0.84	17	2.4%	−1.19 [−1.85, −0.53]
Monteiro 2020 IgA placebo	−4.07	1.06	27	−3.09	0.93	17	2.4%	−0.95 [−1.59, −0.31]
Kohut 2005 (Panama/2007/99-H3N2-12 week)	−0.29	0.37	14	−0.18	0.39	13	2.3%	−0.28 [−1.04, 0.48]
Hayney 2014 (A/Brisbane H1N1-12 week)	36.6	13.82	47	38.4	16.38	51	2.7%	−0.12 [−0.51, 0.28]
Kohut 2005 (Panama/2007/99-H3N2-4 week)	−0.25	0.71	14	−0.22	0.47	13	2.3%	−0.05 [−0.80, 0.71]
Hayney 2014 (B/Brisbane -12 week)	31.8	17.22	47	31	19.25	51	2.7%	0.04 [−0.35, 0.44]
Hayney 2014 (B/Brisbane-3 week)	41	18.61	47	37.3	17.19	51	2.7%	0.21 [−0.19, 0.60]
Monteiro 2020 IgM L-glutamine	−1.77	0.65	26	−2.06	0.76	14	2.4%	0.41 [−0.24, 1.07]
Hayney 2014 (A/Brisbane H3N2-12 week)	121.4	7.98	47	112.2	22.72	51	2.7%	0.53 [0.12, 0.93]
Kohut 2004 (B/Yamanash/166/98-3 month)	−4.13	2.29	14	−5.48	2.56	13	2.3%	0.54 [−0.23, 1.31]
Hayney 2014 (A/Brisbane H3N2-3 week)	184.6	8.18	47	174	22.4	51	2.7%	0.61 [0.21, 1.02]
Monteiro 2020 IgM placebo	−2.29	0.58	27	−2.82	1.02	17	2.5%	0.67 [0.05, 1.29]
Kohut 2004 (B/Yamanashi/166/98-4 week)	−1.95	1.73	14	−3.45	2.13	13	2.2%	0.75 [−0.03, 1.54]
Hayney 2014 (A/Brisbane H1N1-3 week)	58.3	9.85	47	46	17.18	51	2.7%	0.86 [0.45, 1.28]
Kohut 2004 (H3N2-3 month)	−1.67	1.68	14	−3.42	2.12	13	2.2%	0.89 [0.09, 1.69]
Kohut 2004 (H1N1-3 month)	−2.11	1.72	14	−4.06	2.25	13	2.2%	0.95 [0.15, 1.75]
Kohut 2004 (Panama/2007/99-H3N2-4 week)	−1.11	1.53	14	−3.37	2.1	13	2.2%	1.20 [0.37, 2.03]
Kohut 2005 (Caledonia/20/99-H1N1-4 week)	−0.03	0.65	14	−1.82	1.11	13	2.0%	1.93 [0.99, 2.86]
Kohut 2004 (Caledonia/20/99-H1N1-4 week)	−0.5	0.86	14	−4.18	2.39	13	2.0%	2.02 [1.06, 2.97]
Kohut 2005 (Caledonia/20/99-H1N1-12 week)	−0.49	0.88	14	−3.94	1.81	13	1.9%	2.38 [1.36, 3.40]
Monteiro 2020 IgA L-glutamine	1.49	0.71	26	−6.76	2.07	14	1.4%	6.03 [4.50, 7.57]
**Subtotal (95% CI)**			**581**			**529**	**51.4%**	**0.59 [0.21, 0.98]**
Heterogeneity: Tau² = 0.72; Chi² = 183.45, df = 21 (P < 0.00001); I² = 89%								
Test for overall effect: Z = 3.02 (P = 0.003)								
**2.3.4 Not applicable**								
Whitham 2003 (B/Yamanashi/166/88-14 day)	0.51	0.16	7	0.76	0.12	3	1.3%	−1.50 [−3.09, 0.10]
Yang 2007 (Influenza-6 week)	2.32	3.33	27	1.53	2.09	23	2.5%	0.27 [−0.28, 0.83]
Yang 2007 (Influenza-20 week)	2.1	4.3	27	1.1	0.82	23	2.5%	0.31 [−0.25, 0.87]
Yang 2007 (Influenza-3 week)	2.77	4.94	27	1.57	1.46	23	2.5%	0.31 [−0.25, 0.87]
Whitham 2003 (Influenza IgG-12 month)	0.45	0.15	7	0.39	0.1	3	1.5%	0.39 [−0.98, 1.76]
**Subtotal (95% CI)**			**95**			**75**	**10.4%**	**0.22 [−0.13, 0.57]**
Heterogeneity: Tau² = 0.03; Chi² = 4.74, df = 4 (P = 0.32); I² = 16%								
Test for overall effect: Z = 1.24 (P = 0.22)								
**Total (95% CI)**			**1599**			**1437**	**100.0%**	**0.49 [0.25, 0.73]**
Heterogeneity: Tau² = 0.53; Chi² = 389.67, df = 40 (P < 0.00001); I² = 90%								
Test for overall effect: Z = 3.95 (P < 0.0001)								
Test for subgroup differences: Chi² = 2.17, df = 2 (P = 0.34), I² = 7.7%								

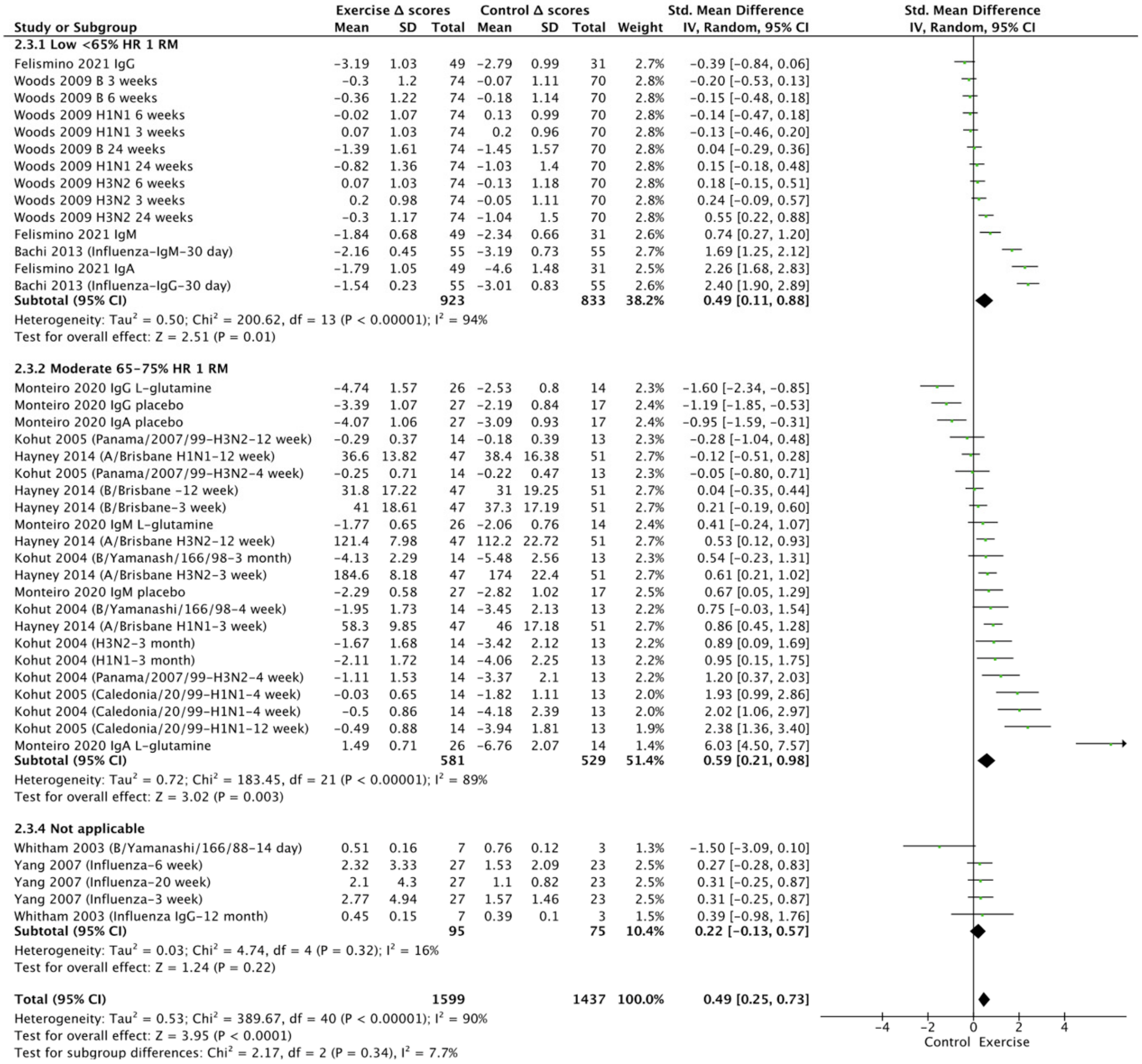

Supplementary Figure S13: Forest plot of the effects of chronic exercise on influenza vaccine antibodies (subgroup analysis for exercise intensity). Δ scores: post intervention – baseline; SD: standard deviation; 95% CI: 95% confidence interval.

Study or Subgroup	Exercise Δ scores Mean	SD	Total	Control Δ scores Mean	SD	Total	Weight	Std. Mean Difference IV, Random, 95% CI
**2.4.1 <59 years**								
Whitham 2003 (B/Yamanashi/166/88-14 day)	0.51	0.16	7	0.76	0.12	3	1.3%	-1.50 [-3.09, 0.10]
Woods 2009 B 3 weeks	-0.3	1.2	74	-0.07	1.11	70	2.8%	-0.20 [-0.53, 0.13]
Woods 2009 B 6 weeks	-0.36	1.22	74	-0.18	1.14	70	2.8%	-0.15 [-0.48, 0.18]
Woods 2009 H1N1 6 weeks	-0.02	1.07	74	0.13	0.99	70	2.8%	-0.14 [-0.47, 0.18]
Woods 2009 H1N1 3 weeks	0.07	1.03	74	0.2	0.96	70	2.8%	-0.13 [-0.46, 0.20]
Hayney 2014 (A/Brisbane H1N1-12 week)	36.6	13.82	47	38.4	16.38	51	2.7%	-0.12 [-0.51, 0.28]
Woods 2009 B 24 weeks	-1.39	1.61	74	-1.45	1.57	70	2.8%	0.04 [-0.29, 0.36]
Hayney 2014 (B/Brisbane -12 week)	31.8	17.22	47	31	19.25	51	2.7%	0.04 [-0.35, 0.44]
Woods 2009 H1N1 24 weeks	-0.82	1.36	74	-1.03	1.4	70	2.8%	0.15 [-0.18, 0.48]
Woods 2009 H3N2 6 weeks	0.07	1.03	74	-0.13	1.18	70	2.8%	0.18 [-0.15, 0.51]
Hayney 2014 (B/Brisbane-3 week)	41	18.61	47	37.3	17.19	51	2.7%	0.21 [-0.19, 0.60]
Woods 2009 H3N2 3 weeks	0.2	0.98	74	-0.05	1.11	70	2.8%	0.24 [-0.09, 0.57]
Whitham 2003 (Influenza IgG-12 month)	0.45	0.15	7	0.39	0.1	3	1.5%	0.39 [-0.98, 1.76]
Hayney 2014 (A/Brisbane H3N2-12 week)	121.4	7.98	47	112.2	22.72	51	2.7%	0.53 [0.12, 0.93]
Woods 2009 H3N2 24 weeks	-0.3	1.17	74	-1.04	1.5	70	2.8%	0.55 [0.22, 0.88]
Hayney 2014 (A/Brisbane H3N2-3 week)	184.6	8.18	47	174	22.4	51	2.7%	0.61 [0.21, 1.02]
Hayney 2014 (A/Brisbane H1N1-3 week)	58.3	9.85	47	46	17.18	51	2.7%	0.86 [0.45, 1.28]
Bachi 2013 (Influenza-IgM-30 day)	-2.16	0.45	55	-3.19	0.73	55	2.7%	1.69 [1.25, 2.12]
Bachi 2013 (Influenza-IgG-30 day)	-1.54	0.23	55	-3.01	0.83	55	2.6%	2.40 [1.90, 2.89]
**Subtotal (95% CI)**			**1072**			**1052**	**49.5%**	**0.34 [0.07, 0.62]**
Heterogeneity: Tau² = 0.31; Chi² = 165.12, df = 18 (P < 0.00001); I² = 89%								
Test for overall effect: Z = 2.46 (P = 0.01)								
**2.4.2 >59 years**								
Monteiro 2020 IgG L-glutamine	-4.74	1.57	26	-2.53	0.8	14	2.3%	-1.60 [-2.34, -0.85]
Monteiro 2020 IgG placebo	-3.39	1.07	27	-2.19	0.84	17	2.4%	-1.19 [-1.85, -0.53]
Monteiro 2020 IgA placebo	-4.07	1.06	27	-3.09	0.93	17	2.4%	-0.95 [-1.59, -0.31]
Felismino 2021 IgG	-3.19	1.03	49	-2.79	0.99	31	2.7%	-0.39 [-0.84, 0.06]
Kohut 2005 (Panama/2007/99-H3N2-12 week)	-0.29	0.37	14	-0.18	0.39	13	2.3%	-0.28 [-1.04, 0.48]
Kohut 2005 (Panama/2007/99-H3N2-4 week)	-0.25	0.71	14	-0.22	0.47	13	2.3%	-0.05 [-0.80, 0.71]
Yang 2007 (Influenza-6 week)	2.32	3.33	27	1.53	2.09	23	2.5%	0.27 [-0.28, 0.83]
Yang 2007 (Influenza-20 week)	2.1	4.3	27	1.1	0.82	23	2.5%	0.31 [-0.25, 0.87]
Yang 2007 (Influenza-3 week)	2.77	4.94	27	1.57	1.46	23	2.5%	0.31 [-0.25, 0.87]
Monteiro 2020 IgM L-glutamine	-1.77	0.65	26	-2.06	0.76	14	2.4%	0.41 [-0.24, 1.07]
Kohut 2004 (B/Yamanash/166/98-3 month)	-4.13	2.29	14	-5.48	2.56	13	2.3%	0.54 [-0.23, 1.31]
Monteiro 2020 IgM placebo	-2.29	0.58	27	-2.82	1.02	17	2.5%	0.67 [0.05, 1.29]
Felismino 2021 IgM	-1.84	0.68	49	-2.34	0.66	31	2.6%	0.74 [0.27, 1.20]
Kohut 2004 (B/Yamanashi/166/98-4 week)	-1.95	1.73	14	-3.45	2.13	13	2.2%	0.75 [-0.03, 1.54]
Kohut 2004 (H3N2-3 month)	-1.67	1.68	14	-3.42	2.12	13	2.2%	0.89 [0.09, 1.69]
Kohut 2004 (H1N1-3 month)	-2.11	1.72	14	-4.06	2.25	13	2.2%	0.95 [0.15, 1.75]
Kohut 2004 (Panama/2007/99-H3N2-4 week)	-1.11	1.53	14	-3.37	2.1	13	2.2%	1.20 [0.37, 2.03]
Kohut 2005 (Caledonia/20/99-H1N1-4 week)	-0.03	0.65	14	-1.82	1.11	13	2.0%	1.93 [0.99, 2.86]
Kohut 2004 (Caledonia/20/99-H1N1-4 week)	-0.5	0.86	14	-4.18	2.39	13	2.0%	2.02 [1.06, 2.97]
Felismino 2021 IgA	-1.79	1.05	49	-4.6	1.48	31	2.5%	2.26 [1.68, 2.83]
Kohut 2005 (Caledonia/20/99-H1N1-12 week)	-0.49	0.88	14	-3.94	1.81	13	1.9%	2.38 [1.36, 3.40]
Monteiro 2020 IgA L-glutamine	1.49	0.71	26	-6.76	2.07	14	1.4%	6.03 [4.50, 7.57]
**Subtotal (95% CI)**			**527**			**385**	**50.5%**	**0.69 [0.22, 1.17]**
Heterogeneity: Tau² = 1.14; Chi² = 220.74, df = 21 (P < 0.00001); I² = 90%								
Test for overall effect: Z = 2.87 (P = 0.004)								
**Total (95% CI)**			**1599**			**1437**	**100.0%**	**0.49 [0.25, 0.73]**
Heterogeneity: Tau² = 0.53; Chi² = 389.67, df = 40 (P < 0.00001); I² = 90%								
Test for overall effect: Z = 3.95 (P < 0.0001)								
Test for subgroup differences: Chi² = 1.57, df = 1 (P = 0.21), I² = 36.4%								

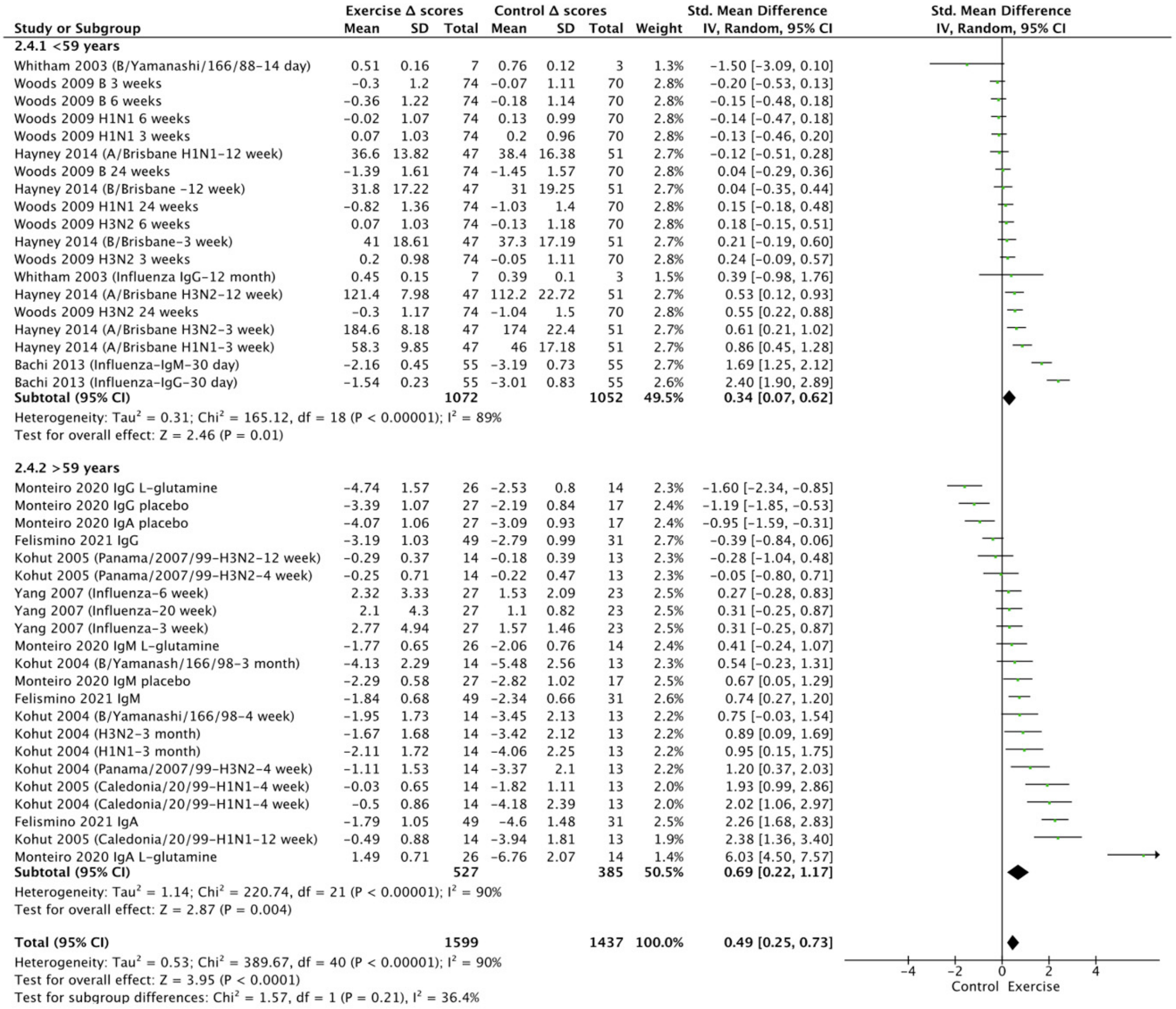

Supplementary Figure S14: Forest plot of the effects of chronic exercise on influenza vaccine antibodies (subgroup analysis for age). Δ scores: post intervention – baseline; SD: standard deviation; 95% CI: 95% confidence interval**.**

Study or Subgroup	Exercise Δ scores Mean	SD	Total	Control Δ scores Mean	SD	Total	Weight	Std. Mean Difference IV, Random, 95% CI
**2.6.1 <4 weeks**								
Monteiro 2020 IgG L-glutamine	−4.74	1.57	26	−2.53	0.8	14	2.3%	−1.60 [−2.34, −0.85]
Whitham 2003 (B/Yamanashi/166/88-14 day)	0.51	0.16	7	0.76	0.12	3	1.3%	−1.50 [−3.09, 0.10]
Monteiro 2020 IgG placebo	−3.39	1.07	27	−2.19	0.84	17	2.4%	−1.19 [−1.85, −0.53]
Monteiro 2020 IgA placebo	−4.07	1.06	27	−3.09	0.93	17	2.4%	−0.95 [−1.59, −0.31]
Felismino 2021 IgG	−3.19	1.03	49	−2.79	0.99	31	2.7%	−0.39 [−0.84, 0.06]
Woods 2009 B 3 weeks	−0.3	1.2	74	−0.07	1.11	70	2.8%	−0.20 [−0.53, 0.13]
Woods 2009 H1N1 3 weeks	0.07	1.03	74	0.2	0.96	70	2.8%	−0.13 [−0.46, 0.20]
Kohut 2005 (Panama/2007/99-H3N2-4 week)	−0.25	0.71	14	−0.22	0.47	13	2.3%	−0.05 [−0.80, 0.71]
Hayney 2014 (B/Brisbane-3 week)	41	18.61	47	37.3	17.19	51	2.7%	0.21 [−0.19, 0.60]
Woods 2009 H3N2 3 weeks	0.2	0.98	74	−0.05	1.11	70	2.8%	0.24 [−0.09, 0.57]
Yang 2007 (Influenza-3 week)	2.77	4.94	27	1.57	1.46	23	2.5%	0.31 [−0.25, 0.87]
Monteiro 2020 IgM L-glutamine	−1.77	0.65	26	−2.06	0.76	14	2.4%	0.41 [−0.24, 1.07]
Hayney 2014 (A/Brisbane H3N2-3 week)	184.6	8.18	47	174	22.4	51	2.7%	0.61 [0.21, 1.02]
Monteiro 2020 IgM placebo	−2.29	0.58	27	−2.82	1.02	17	2.5%	0.67 [0.05, 1.29]
Felismino 2021 IgM	−1.84	0.68	49	−2.34	0.66	31	2.6%	0.74 [0.27, 1.20]
Kohut 2004 (B/Yamanashi/166/98-4 week)	−1.95	1.73	14	−3.45	2.13	13	2.2%	0.75 [−0.03, 1.54]
Hayney 2014 (A/Brisbane H1N1-3 week)	58.3	9.85	47	46	17.18	51	2.7%	0.86 [0.45, 1.28]
Kohut 2004 (Panama/2007/99-H3N2-4 week)	−1.11	1.53	14	−3.37	2.1	13	2.2%	1.20 [0.37, 2.03]
Bachi 2013 (Influenza-IgM-30 day)	−2.16	0.45	55	−3.19	0.73	55	2.7%	1.69 [1.25, 2.12]
Kohut 2005 (Caledonia/20/99-H1N1-4 week)	−0.03	0.65	14	−1.82	1.11	13	2.0%	1.93 [0.99, 2.86]
Kohut 2004 (Caledonia/20/99-H1N1-4 week)	−0.5	0.86	14	−4.18	2.39	13	2.0%	2.02 [1.06, 2.97]
Felismino 2021 IgA	−1.79	1.05	49	−4.6	1.48	31	2.5%	2.26 [1.68, 2.83]
Bachi 2013 (Influenza-IgG-30 day)	−1.54	0.23	55	−3.01	0.83	55	2.6%	2.40 [1.90, 2.89]
Monteiro 2020 IgA L-glutamine	1.49	0.71	26	−6.76	2.07	14	1.4%	6.03 [4.50, 7.57]
**Subtotal (95% CI)**			**883**			**750**	**57.6%**	**0.63 [0.21, 1.04]**
Heterogeneity: Tau² = 0.95; Chi² = 330.87, df = 23 (P < 0.00001); I² = 93%								
Test for overall effect: Z = 2.96 (P = 0.003)								
**2.6.2 >4 weeks**								
Kohut 2005 (Panama/2007/99-H3N2-12 week)	−0.29	0.37	14	−0.18	0.39	13	2.3%	−0.28 [−1.04, 0.48]
Woods 2009 B 6 weeks	−0.36	1.22	74	−0.18	1.14	70	2.8%	−0.15 [−0.48, 0.18]
Woods 2009 H1N1 6 weeks	−0.02	1.07	74	0.13	0.99	70	2.8%	−0.14 [−0.47, 0.18]
Hayney 2014 (A/Brisbane H1N1-12 week)	36.6	13.82	47	38.4	16.38	51	2.7%	−0.12 [−0.51, 0.28]
Woods 2009 B 24 weeks	−1.39	1.61	74	−1.45	1.57	70	2.8%	0.04 [−0.29, 0.36]
Hayney 2014 (B/Brisbane -12 week)	31.8	17.22	47	31	19.25	51	2.7%	0.04 [−0.35, 0.44]
Woods 2009 H1N1 24 weeks	−0.82	1.36	74	−1.03	1.4	70	2.8%	0.15 [−0.18, 0.48]
Woods 2009 H3N2 6 weeks	0.07	1.03	74	−0.13	1.18	70	2.8%	0.18 [−0.15, 0.51]
Yang 2007 (Influenza-6 week)	2.32	3.33	27	1.53	2.09	23	2.5%	0.27 [−0.28, 0.83]
Yang 2007 (Influenza-20 week)	2.1	4.3	27	1.1	0.82	23	2.5%	0.31 [−0.25, 0.87]
Whitham 2003 (Influenza IgG-12 month)	0.45	0.15	7	0.39	0.1	3	1.5%	0.39 [−0.98, 1.76]
Hayney 2014 (A/Brisbane H3N2-12 week)	121.4	7.98	47	112.2	22.72	51	2.7%	0.53 [0.12, 0.93]
Kohut 2004 (B/Yamanash/166/98-3 month)	−4.13	2.29	14	−5.48	2.56	13	2.3%	0.54 [−0.23, 1.31]
Woods 2009 H3N2 24 weeks	−0.3	1.17	74	−1.04	1.5	70	2.8%	0.55 [0.22, 0.88]
Kohut 2004 (H3N2-3 month)	−1.67	1.68	14	−3.42	2.12	13	2.2%	0.89 [0.09, 1.69]
Kohut 2004 (H1N1-3 month)	−2.11	1.72	14	−4.06	2.25	13	2.2%	0.95 [0.15, 1.75]
Kohut 2005 (Caledonia/20/99-H1N1-12 week)	−0.49	0.88	14	−3.94	1.81	13	1.9%	2.38 [1.36, 3.40]
**Subtotal (95% CI)**			**716**			**687**	**42.4%**	**0.26 [0.07, 0.46]**
Heterogeneity: Tau² = 0.10; Chi² = 45.75, df = 16 (P = 0.0001); I² = 65%								
Test for overall effect: Z = 2.65 (P = 0.008)								
**Total (95% CI)**			**1599**			**1437**	**100.0%**	**0.49 [0.25, 0.73]**
Heterogeneity: Tau² = 0.53; Chi² = 389.67, df = 40 (P < 0.00001); I² = 90%								
Test for overall effect: Z = 3.95 (P < 0.0001)								
Test for subgroup differences: Chi² = 2.43, df = 1 (P = 0.12), I² = 58.9%								

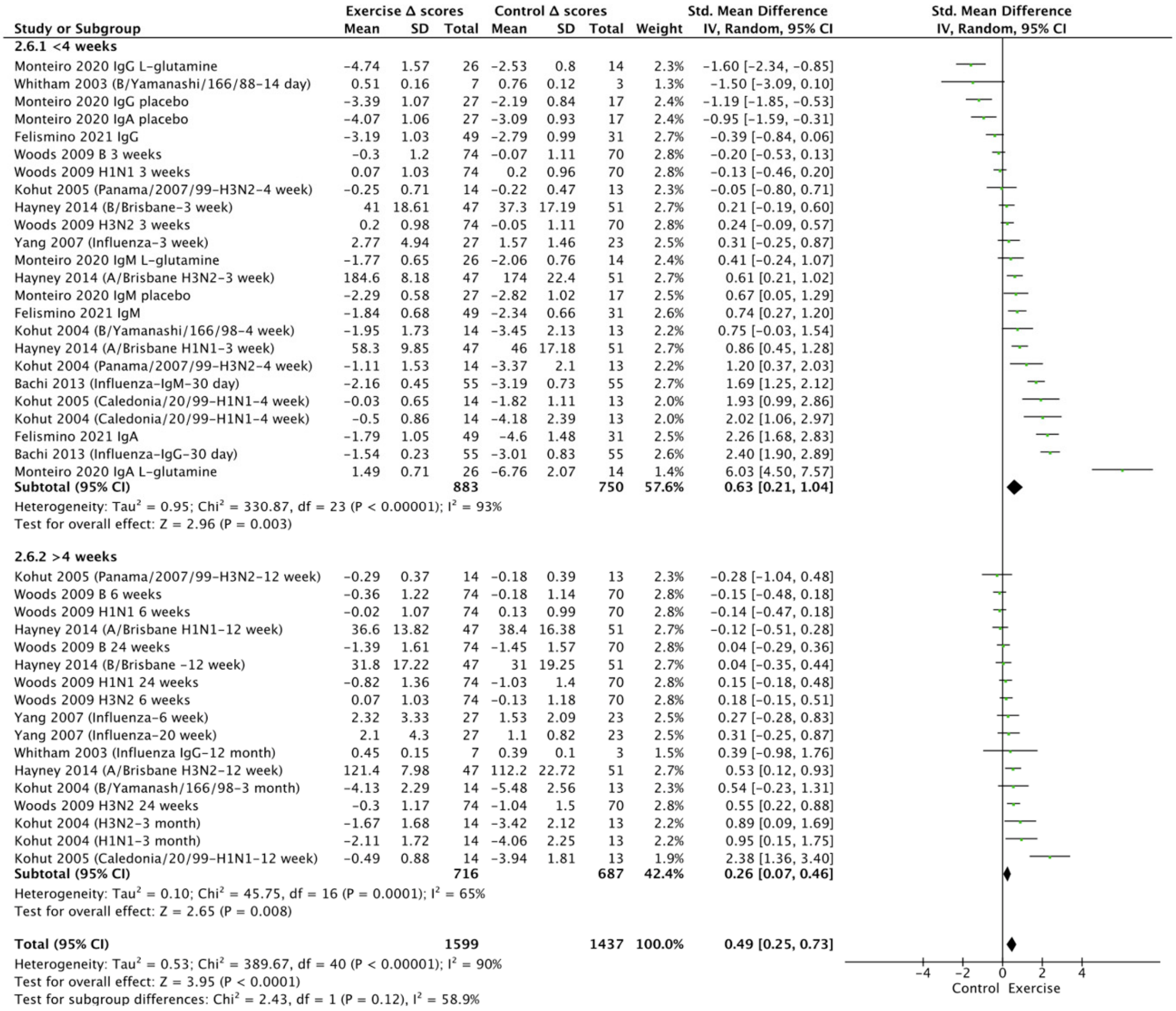

Supplementary Figure S15: Forest plot of the effects of chronic exercise on influenza vaccine antibodies (subgroup analysis for measurements of <4 weeks vs. >4 weeks from vaccination). Δ scores: post intervention – baseline; SD: standard deviation; 95% CI: 95% confidence interval.

Study or Subgroup	Exercise Δ scores Mean	SD	Total	Control Δ scores Mean	SD	Total	Weight	Std. Mean Difference IV, Random, 95% CI
**2.5.1 H1N1**								
Woods 2009 H1N1 6 weeks	−0.02	1.07	74	0.13	0.99	70	2.8%	−0.14 [−0.47, 0.18]
Woods 2009 H1N1 3 weeks	0.07	1.03	74	0.2	0.96	70	2.8%	−0.13 [−0.46, 0.20]
Hayney 2014 (A/Brisbane H1N1−12 week)	36.6	13.82	47	38.4	16.38	51	2.7%	−0.12 [−0.51, 0.28]
Woods 2009 H1N1 24 weeks	−0.82	1.36	74	−1.03	1.4	70	2.8%	0.15 [−0.18, 0.48]
Whitham 2003 (Influenza IgG−12 month)	0.45	0.15	7	0.39	0.1	3	1.5%	0.39 [−0.98, 1.76]
Hayney 2014 (A/Brisbane H1N1−3 week)	58.3	9.85	47	46	17.18	51	2.7%	0.86 [0.45, 1.28]
Kohut 2004 (H1N1−3 month)	−2.11	1.72	14	−4.06	2.25	13	2.2%	0.95 [0.15, 1.75]
Kohut 2005 (Caledonia/20/99−H1N1−4 week)	−0.03	0.65	14	−1.82	1.11	13	2.0%	1.93 [0.99, 2.86]
Kohut 2004 (Caledonia/20/99−H1N1−4 week)	−0.5	0.86	14	−4.18	2.39	13	2.0%	2.02 [1.06, 2.97]
Kohut 2005 (Caledonia/20/99−H1N1−12 week)	−0.49	0.88	14	−3.94	1.81	13	1.9%	2.38 [1.36, 3.40]
**Subtotal (95% CI)**			**379**			**367**	**23.5%**	**0.70 [0.25, 1.15]**
Heterogeneity: Tau² = 0.40; Chi² = 67.96, df = 9 (P < 0.00001); I² = 87%								
Test for overall effect: Z = 3.03 (P = 0.002)								
**2.5.2 H3N2**								
Kohut 2005 (Panama/2007/99−H3N2−12 week)	−0.29	0.37	14	−0.18	0.39	13	2.3%	−0.28 [−1.04, 0.48]
Kohut 2005 (Panama/2007/99−H3N2−4 week)	−0.25	0.71	14	−0.22	0.47	13	2.3%	−0.05 [−0.80, 0.71]
Woods 2009 H3N2 6 weeks	0.07	1.03	74	−0.13	1.18	70	2.8%	0.18 [−0.15, 0.51]
Woods 2009 H3N2 3 weeks	0.2	0.98	74	−0.05	1.11	70	2.8%	0.24 [−0.09, 0.57]
Hayney 2014 (A/Brisbane H3N2−12 week)	121.4	7.98	47	112.2	22.72	51	2.7%	0.53 [0.12, 0.93]
Woods 2009 H3N2 24 weeks	−0.3	1.17	74	−1.04	1.5	70	2.8%	0.55 [0.22, 0.88]
Hayney 2014 (A/Brisbane H3N2−3 week)	184.6	8.18	47	174	22.4	51	2.7%	0.61 [0.21, 1.02]
Kohut 2004 (H3N2−3 month)	−1.67	1.68	14	−3.42	2.12	13	2.2%	0.89 [0.09, 1.69]
Kohut 2004 (Panama/2007/99−H3N2−4 week)	−1.11	1.53	14	−3.37	2.1	13	2.2%	1.20 [0.37, 2.03]
**Subtotal (95% CI)**			**372**			**364**	**22.8%**	**0.41 [0.20, 0.62]**
Heterogeneity: Tau² = 0.04; Chi² = 14.36, df = 8 (P = 0.07); I² = 44%								
Test for overall effect: Z = 3.79 (P = 0.0002)								
**2.5.3 B**								
Whitham 2003 (B/Yamanashi/166/88−14 day)	0.51	0.16	7	0.76	0.12	3	1.3%	−1.50 [−3.09, 0.10]
Woods 2009 B 3 weeks	−0.3	1.2	74	−0.07	1.11	70	2.8%	−0.20 [−0.53, 0.13]
Woods 2009 B 6 weeks	−0.36	1.22	74	−0.18	1.14	70	2.8%	−0.15 [−0.48, 0.18]
Woods 2009 B 24 weeks	−1.39	1.61	74	−1.45	1.57	70	2.8%	0.04 [−0.29, 0.36]
Hayney 2014 (B/Brisbane −12 week)	31.8	17.22	47	31	19.25	51	2.7%	0.04 [−0.35, 0.44]
Hayney 2014 (B/Brisbane−3 week)	41	18.61	47	37.3	17.19	51	2.7%	0.21 [−0.19, 0.60]
Kohut 2004 (B/Yamanash/166/98−3 month)	−4.13	2.29	14	−5.48	2.56	13	2.3%	0.54 [−0.23, 1.31]
Kohut 2004 (B/Yamanashi/166/98−4 week)	−1.95	1.73	14	−3.45	2.13	13	2.2%	0.75 [−0.03, 1.54]
**Subtotal (95% CI)**			**351**			**341**	**19.6%**	**0.03 [−0.18, 0.25]**
Heterogeneity: Tau² = 0.04; Chi² = 12.14, df = 7 (P = 0.10); I² = 42%								
Test for overall effect: Z = 0.31 (P = 0.75)								
**2.5.4 All strains**								
Monteiro 2020 IgG L-glutamine	−4.74	1.57	26	−2.53	0.8	14	2.3%	−1.60 [−2.34, −0.85]
Monteiro 2020 IgG placebo	−3.39	1.07	27	−2.19	0.84	17	2.4%	−1.19 [−1.85, −0.53]
Monteiro 2020 IgA placebo	−4.07	1.06	27	−3.09	0.93	17	2.4%	−0.95 [−1.59, −0.31]
Felismino 2021 IgG	−3.19	1.03	49	−2.79	0.99	31	2.7%	−0.39 [−0.84, 0.06]
Yang 2007 (Influenza−6 week)	2.32	3.33	27	1.53	2.09	23	2.5%	0.27 [−0.28, 0.83]
Yang 2007 (Influenza−20 week)	2.1	4.3	27	1.1	0.82	23	2.5%	0.31 [−0.25, 0.87]
Yang 2007 (Influenza−3 week)	2.77	4.94	27	1.57	1.46	23	2.5%	0.31 [−0.25, 0.87]
Monteiro 2020 IgM L-glutamine	−1.77	0.65	26	−2.06	0.76	14	2.4%	0.41 [−0.24, 1.07]
Monteiro 2020 IgM placebo	−2.29	0.58	27	−2.82	1.02	17	2.5%	0.67 [0.05, 1.29]
Felismino 2021 IgM	−1.84	0.68	49	−2.34	0.66	31	2.6%	0.74 [0.27, 1.20]
Bachi 2013 (Influenza−IgM−30 day)	−2.16	0.45	55	−3.19	0.73	55	2.7%	1.69 [1.25, 2.12]
Felismino 2021 IgA	−1.79	1.05	49	−4.6	1.48	31	2.5%	2.26 [1.68, 2.83]
Bachi 2013 (Influenza−IgG−30 day)	−1.54	0.23	55	−3.01	0.83	55	2.6%	2.40 [1.90, 2.89]
Monteiro 2020 IgA L-glutamine	1.49	0.71	26	−6.76	2.07	14	1.4%	6.03 [4.50, 7.57]
**Subtotal (95% CI)**			**497**			**365**	**34.1%**	**0.70 [0.01, 1.39]**
Heterogeneity: Tau² = 1.63; Chi² = 260.62, df = 13 (P < 0.00001); I² = 95%								
Test for overall effect: Z = 1.99 (P = 0.05)								
**Total (95% CI)**			**1599**			**1437**	**100.0%**	**0.49 [0.25, 0.73]**
Heterogeneity: Tau² = 0.53; Chi² = 389.67, df = 40 (P < 0.00001); I² = 90%								
Test for overall effect: Z = 3.95 (P < 0.0001)								
Test for subgroup differences: Chi² = 11.23, df = 3 (P = 0.01), I² = 73.3%								

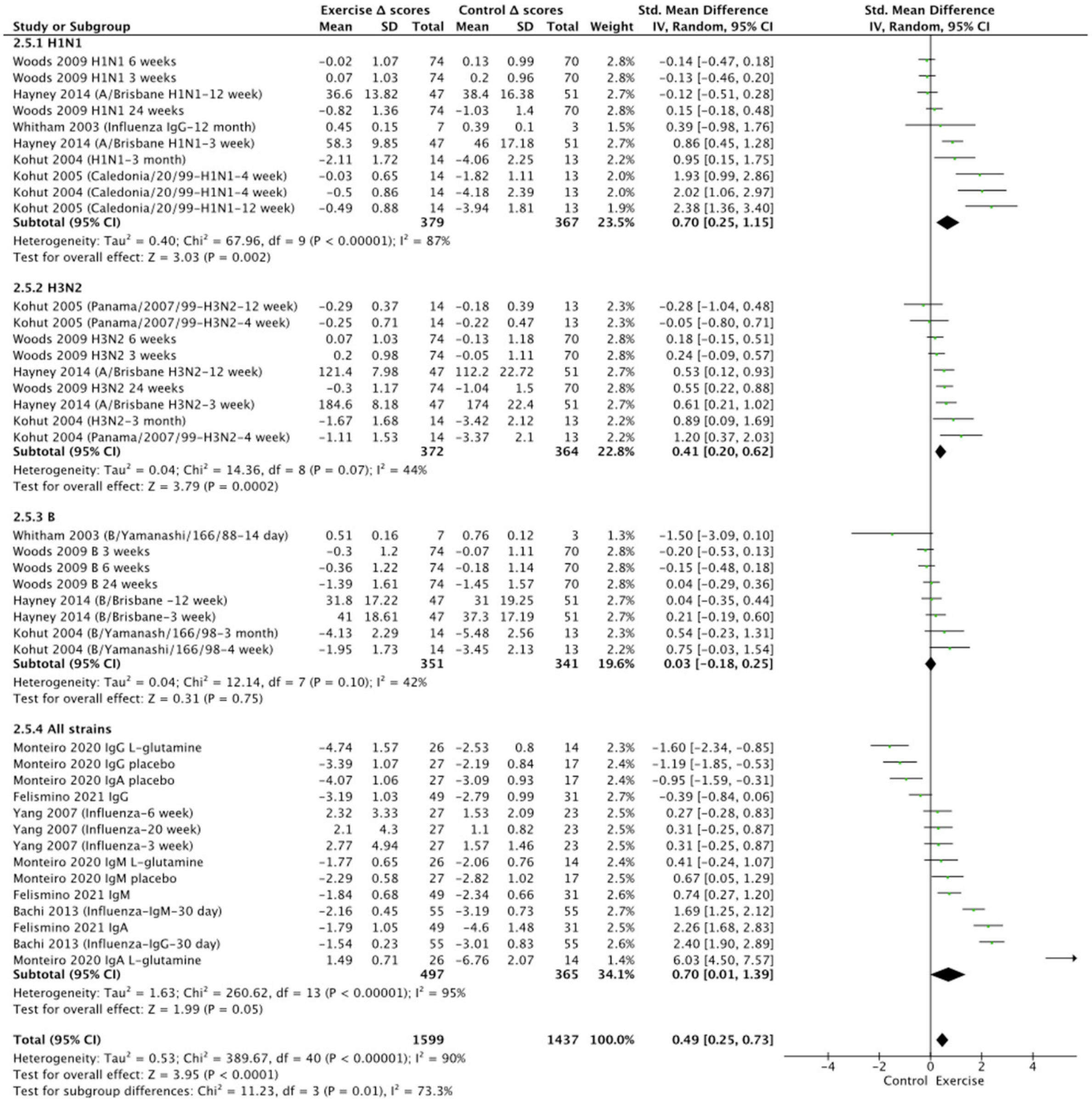

Supplementary Figure S16: Forest plot of the effects of chronic exercise on influenza vaccine antibodies (subgroup analysis for antibodies type). Δ scores: post intervention – baseline; SD: standard deviation; 95% CI: 95% confidence interval.

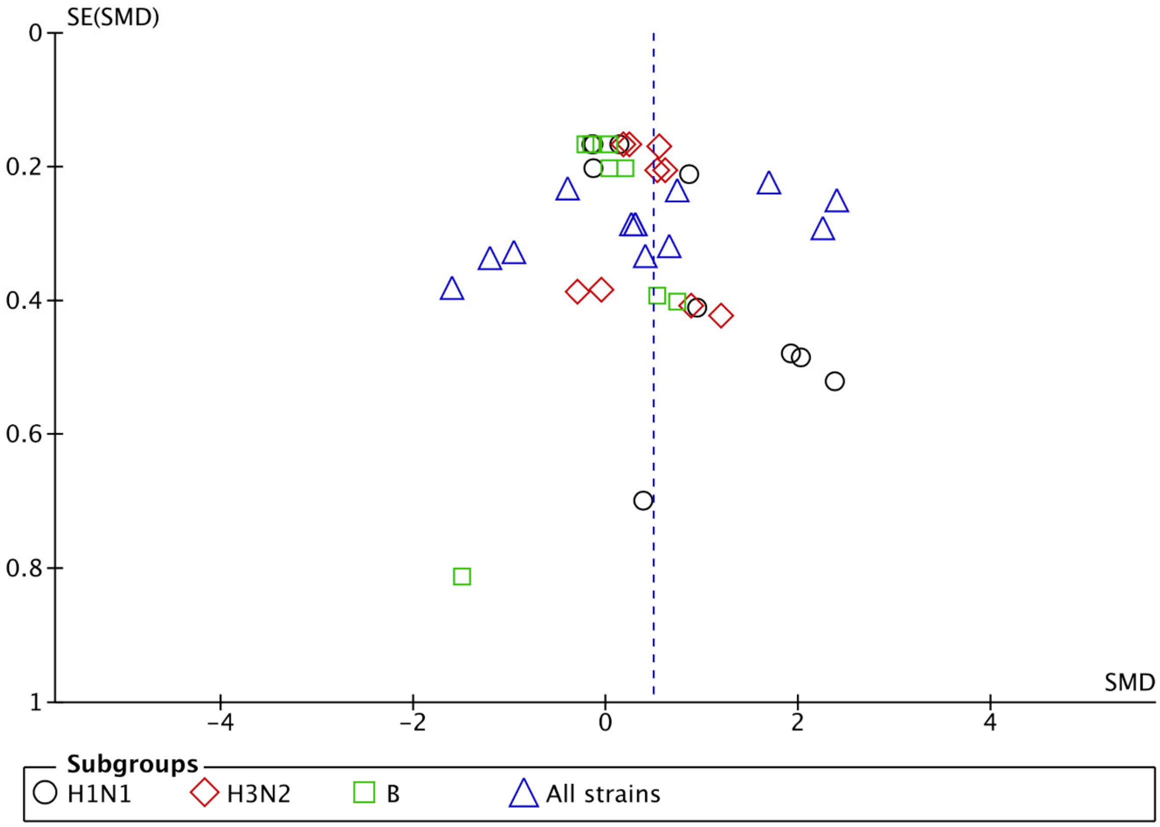

Supplementary Figure S17. Funnel plot of the effects of chronic exercise on influenza vaccine antibodies (subgroup analysis for antibodies type). SMD: standardized mean difference; SE: standard error

Study or Subgroup	Exercise Δ scores Mean	SD	Total	Control Δ scores Mean	SD	Total	Weight	Std. Mean Difference IV, Random, 95% CI
**2.8.1 H1N1**								
Woods 2009 H1N1 6 weeks	−0.02	1.07	74	0.13	0.99	70	4.8%	−0.14 [−0.47, 0.18]
Woods 2009 H1N1 3 weeks	0.07	1.03	74	0.2	0.96	70	4.8%	−0.13 [−0.46, 0.20]
Hayney 2014 (A/Brisbane H1N1-12 week)	36.6	13.82	47	38.4	16.38	51	4.5%	−0.12 [−0.51, 0.28]
Woods 2009 H1N1 24 weeks	−0.82	1.36	74	−1.03	1.4	70	4.8%	0.15 [−0.18, 0.48]
Hayney 2014 (A/Brisbane H1N1-3 week)	58.3	9.85	47	46	17.18	51	4.4%	0.86 [0.45, 1.28]
Kohut 2004 (H1N1-3 month)	−2.11	1.72	14	−4.06	2.25	13	2.8%	0.95 [0.15, 1.75]
Kohut 2005 (Caledonia/20/99-H1N1-4 week)	−0.03	0.65	14	−1.82	1.11	13	2.4%	1.93 [0.99, 2.86]
Kohut 2004 (Caledonia/20/99-H1N1-4 week)	−0.5	0.86	14	−4.18	2.39	13	2.3%	2.02 [1.06, 2.97]
Kohut 2005 (Caledonia/20/99-H1N1-12 week)	−0.49	0.88	14	−3.94	1.81	13	2.1%	2.38 [1.36, 3.40]
**Subtotal (95% CI)**			**372**			**364**	**33.1%**	**0.72 [0.25, 1.19]**
Heterogeneity: $Tau^2 = 0.42$; $Chi^2 = 67.92$, df = 8 (P < 0.00001); $I^2 = 88\%$								
Test for overall effect: Z = 3.00 (P = 0.003)								
**2.8.2 H3N2**								
Kohut 2005 (Panama/2007/99-H3N2-12 week)	−0.29	0.37	14	−0.18	0.39	13	3.0%	−0.28 [−1.04, 0.48]
Kohut 2005 (Panama/2007/99-H3N2-4 week)	−0.25	0.71	14	−0.22	0.47	13	3.0%	−0.05 [−0.80, 0.71]
Woods 2009 H3N2 6 weeks	0.07	1.03	74	−0.13	1.18	70	4.8%	0.18 [−0.15, 0.51]
Woods 2009 H3N2 3 weeks	0.2	0.98	74	−0.05	1.11	70	4.8%	0.24 [−0.09, 0.57]
Hayney 2014 (A/Brisbane H3N2-12 week)	121.4	7.98	47	112.2	22.72	51	4.5%	0.53 [0.12, 0.93]
Woods 2009 H3N2 24 weeks	−0.3	1.17	74	−1.04	1.5	70	4.8%	0.55 [0.22, 0.88]
Hayney 2014 (A/Brisbane H3N2-3 week)	184.6	8.18	47	174	22.4	51	4.5%	0.61 [0.21, 1.02]
Kohut 2004 (H3N2-3 month)	−1.67	1.68	14	−3.42	2.12	13	2.8%	0.89 [0.09, 1.69]
Kohut 2004 (Panama/2007/99-H3N2-4 week)	−1.11	1.53	14	−3.37	2.1	13	2.7%	1.20 [0.37, 2.03]
**Subtotal (95% CI)**			**372**			**364**	**34.9%**	**0.41 [0.20, 0.62]**
Heterogeneity: $Tau^2 = 0.04$; $Chi^2 = 14.36$, df = 8 (P = 0.07); $I^2 = 44\%$								
Test for overall effect: Z = 3.79 (P = 0.0002)								
**2.8.3 B**								
Whitham 2003 (B/Yamanashi/166/88-14 day)	0.51	0.16	7	0.76	0.12	3	1.1%	−1.50 [−3.09, 0.10]
Woods 2009 B 3 weeks	−0.3	1.2	74	−0.07	1.11	70	4.8%	−0.20 [−0.53, 0.13]
Woods 2009 B 6 weeks	−0.36	1.22	74	−0.18	1.14	70	4.8%	−0.15 [−0.48, 0.18]
Woods 2009 B 24 weeks	−1.39	1.61	74	−1.45	1.57	70	4.8%	0.04 [−0.29, 0.36]
Hayney 2014 (B/Brisbane -12 week)	31.8	17.22	47	31	19.25	51	4.5%	0.04 [−0.35, 0.44]
Hayney 2014 (B/Brisbane-3 week)	41	18.61	47	37.3	17.19	51	4.5%	0.21 [−0.19, 0.60]
Whitham 2003 (Influenza IgG-12 month)	0.45	0.15	7	0.39	0.1	3	1.4%	0.39 [−0.98, 1.76]
Kohut 2004 (B/Yamanash/166/98-3 month)	−4.13	2.29	14	−5.48	2.56	13	2.9%	0.54 [−0.23, 1.31]
Kohut 2004 (B/Yamanashi/166/98-4 week)	−1.95	1.73	14	−3.45	2.13	13	2.9%	0.75 [−0.03, 1.54]
**Subtotal (95% CI)**			**358**			**344**	**31.9%**	**0.04 [−0.16, 0.24]**
Heterogeneity: $Tau^2 = 0.03$; $Chi^2 = 12.44$, df = 8 (P = 0.13); $I^2 = 36\%$								
Test for overall effect: Z = 0.37 (P = 0.71)								
**Total (95% CI)**			**1102**			**1072**	**100.0%**	**0.37 [0.18, 0.56]**
Heterogeneity: $Tau^2 = 0.17$; $Chi^2 = 108.09$, df = 26 (P < 0.00001); $I^2 = 76\%$								
Test for overall effect: Z = 3.78 (P = 0.0002)								
Test for subgroup differences: $Chi^2 = 10.11$, df = 2 (P = 0.006), $I^2 = 80.2\%$								

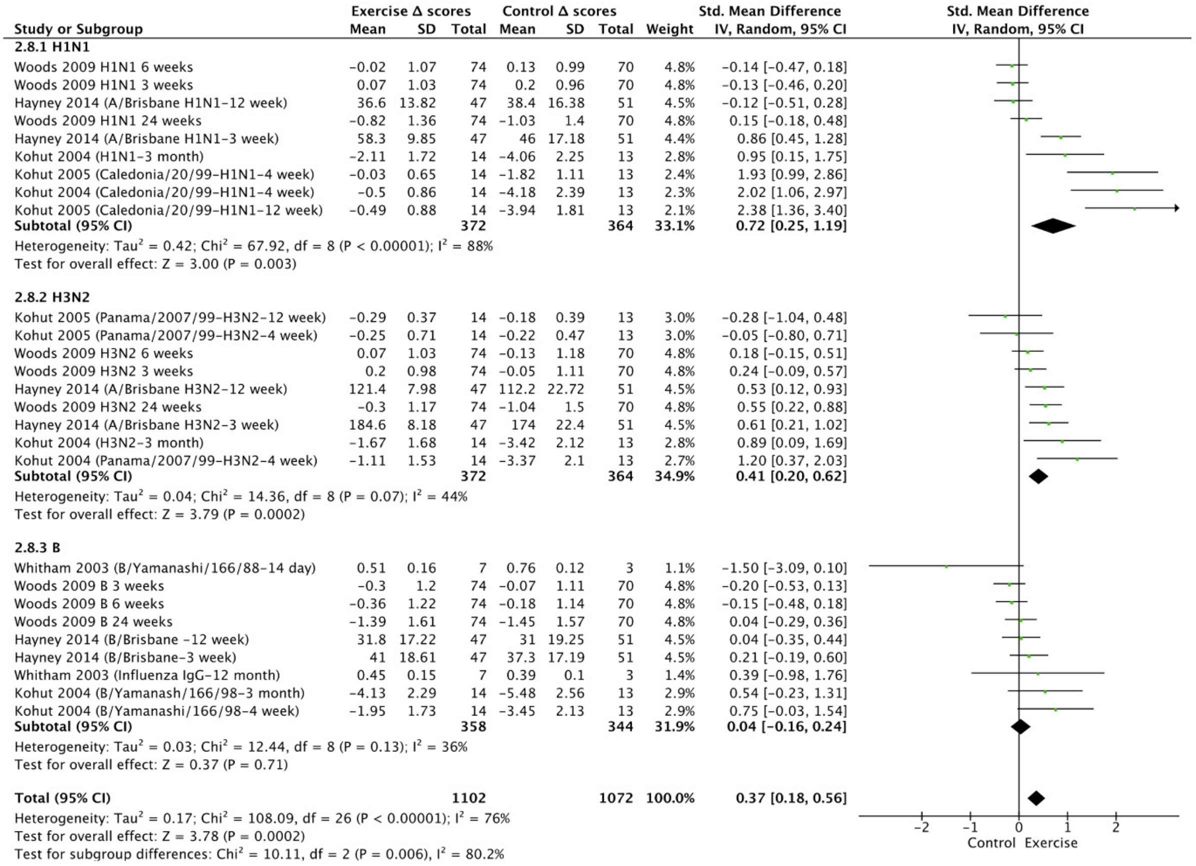

Supplementary Figure S18. Forest plot of the effects of chronic aerobic exercise on influenza antibodies (subgroup analysis for antibodies type). Δ scores: post intervention – baseline; SD: standard deviation; 95% CI: 95% confidence interval.

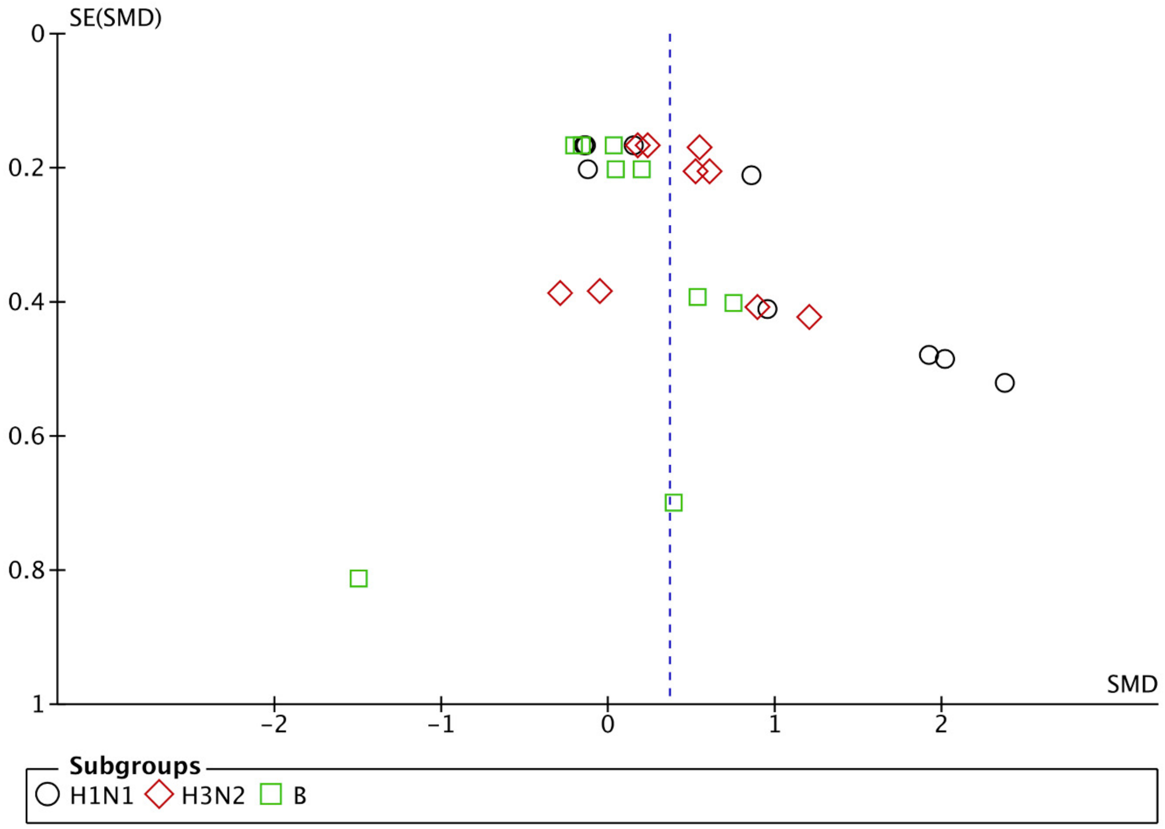

Supplementary Figure S19. Funnel plot of the effects of chronic aerobic exercise on influenza antibodies (subgroup analysis for antibodies type). SMD: standardized mean difference; SE: standard error**.**

Study or Subgroup	Active Δ scores Mean	SD	Total	Inactive/low act Δ scores Mean	SD	Total	Weight	Std. Mean Difference IV, Random, 95% CI
Gleeson 1996 (Pneumococcal-4-14 day)	3,352	4,021	20	4,463	7,600.24	19	14.6%	−0.18 [−0.81, 0.45]
Gleeson 1996 (Pneumococcal-14-14 day)	4,241	6,024.1	20	4,955	19,729.94	19	14.6%	−0.05 [−0.68, 0.58]
Turner 2021	83.4187	62.7697	45	82.62	58.02	10	12.3%	0.01 [−0.67, 0.70]
Gleeson 1996 (Pneumococcal-6B-14 day)	1,794	2,881.71	20	1,629	3,147.92	19	14.6%	0.05 [−0.57, 0.68]
Gleeson 1996 (Pneumococcal-18C-14 day)	26,540	41,205.76	20	20,823	59,646.4	19	14.6%	0.11 [−0.52, 0.74]
Gleeson 1996 (Pneumococcal-23F-14 day)	5,769	12,186.36	20	4,113	15,634.75	19	14.6%	0.12 [−0.51, 0.74]
Gleeson 1996 (Pneumococcal-8-14 day)	4,687	3,896.43	20	3,857	4,229.18	19	14.6%	0.20 [−0.43, 0.83]
**Total (95% CI)**			**165**			**124**	**100.0%**	**0.04 [−0.20, 0.28]**

Heterogeneity: $Tau^2 = 0.00$; $Chi^2 = 0.91$, $df = 6$ ($P = 0.99$); $I^2 = 0\%$
Test for overall effect: $Z = 0.31$ ($P = 0.76$)

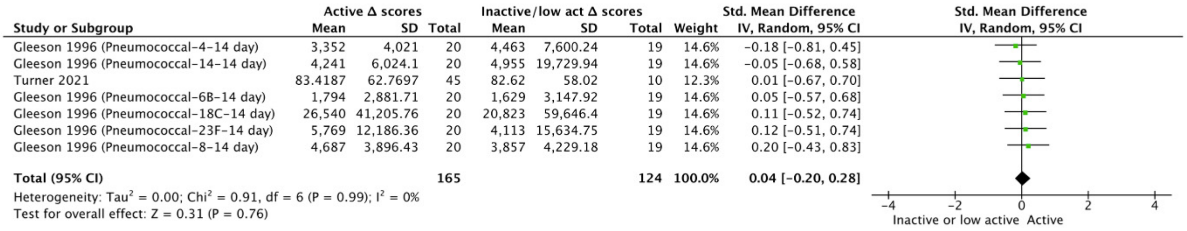

Supplementary Figure S20. Forest plot of the effects of physical activity levels on pneumococcal antibodies. Δ scores: post intervention – baseline; SD: standard deviation; 95% CI: 95% confidence interval

Study or Subgroup	Active Δ scores Mean	SD	Total	Inactive/low act Δ scores Mean	SD	Total	Weight	Std. Mean Difference IV, Random, 95% CI
Schuler 1999 (H1-4 week)	2	29.57	13	46.1	49.97	31	1.5%	-0.96 [-1.64, -0.28]
Schuler 1999 (Moderate levels-H1-4 week)	8.5	36.11	31	43.2	41.69	13	1.5%	-0.90 [-1.58, -0.22]
Schuler 1999 (H1a-4 week)	7.5	42.9	23	43.2	41.69	13	1.4%	-0.82 [-1.53, -0.11]
Schuler 1999 (Moderate levels-H1-2 week)	3.3	28.58	31	29.9	39.86	13	1.5%	-0.81 [-1.48, -0.14]
Schuler 1999 (Moderate fitness-H1-1 week)	3.6	37.98	23	33.8	43.19	31	1.6%	-0.72 [-1.28, -0.17]
Schuler 1999 (Moderate fitness-H1-4 week)	13.2	40.07	23	46.1	49.97	31	1.6%	-0.70 [-1.26, -0.15]
Schuler 1999 (H1-6 week)	-4.4	27.5	13	14.6	27.59	31	1.5%	-0.68 [-1.34, -0.01]
Schuler 1999 (H1-2 week)	5.2	30.57	13	24.6	27.85	31	1.5%	-0.66 [-1.33, -0.00]
Schuler 1999 (H1a-6 week)	-6.6	32.01	23	13.9	28.73	13	1.4%	-0.65 [-1.35, 0.05]
Schuler 1999 (Moderate fitness-H1-6 week)	-4.3	34.62	23	14.6	27.59	31	1.6%	-0.61 [-1.16, -0.05]
Schuler 1999 (H1a-2 week)	7.2	35.48	23	29.9	39.86	13	1.5%	-0.60 [-1.29, 0.10]
Schuler 1999 (Moderate levels-H1-6 week)	-3.8	30.56	31	13.9	28.73	13	1.5%	-0.58 [-1.24, 0.08]
Schuler 1999 (H1-1 week)	9.2	38.79	13	33.8	43.19	31	1.5%	-0.58 [-1.24, 0.08]
Kohut 2002 (Moderate-Infuenza-IgM-1:125-14 days)	-1.2	0.75	25	-0.76	0.75	15	1.5%	-0.58 [-1.23, 0.08]
Schuler 1999 (Moderate fitness-H1-2 week)	7.5	38.8	23	24.6	27.85	31	1.6%	-0.51 [-1.06, 0.04]
Schuler 1999 (H3a-4 week)	37.9	34.97	23	64.5	86.25	13	1.5%	-0.44 [-1.13, 0.24]
Kohut 2002 (Moderate-Infuenza-IgG-1:25-14 days)	1.11	0.08	25	1.14	0.04	15	1.5%	-0.43 [-1.08, 0.22]
Schuler 1999 (Moderate levels-H3-4 week)	38.5	64.38	31	64.5	86.25	13	1.5%	-0.36 [-1.01, 0.29]
Schuler 1999 (Moderate levels-H3-2 week)	39.7	49.69	31	55.4	52.5	13	1.5%	-0.31 [-0.96, 0.35]
Schuler 1999 (Moderate levels-H1-1 week)	9	43.6	31	17.2	24.38	13	1.5%	-0.21 [-0.86, 0.44]
Schuler 1999 (H1a-1 week)	10.5	49.53	23	17.2	24.38	13	1.5%	-0.15 [-0.84, 0.53]
Kohut 2002 (Infuenza-IgM-1:25-14 days)	-0.01	0.56	25	0.06	0.55	15	1.5%	-0.12 [-0.76, 0.52]
Kohut 2002 (Moderate-Infuenza-IgG-1:125-14 days)	0.97	0.11	25	0.98	0.13	15	1.5%	-0.08 [-0.72, 0.56]
Schuler 1999 (H3-2 week)	40.8	52.49	13	43.9	41.05	31	1.5%	-0.07 [-0.72, 0.58]
Schuler 1999 (Moderate levels-H3-1 week)	14.4	39.7	31	15.4	14.83	13	1.5%	-0.03 [-0.68, 0.62]
de Araujo 2015 (H1N1-6 w post vaccination)	2.35	2.65	22	2.38	1.85	16	1.5%	-0.01 [-0.66, 0.63]
Schuler 1999 (H3a-6 week)	64.3	70.17	23	62.3	58.89	13	1.5%	0.03 [-0.65, 0.71]
Wong 2019 (B/Massachusetts/02/2012-1-28 day)	3,595.34	2,106.52	28	3,515.91	1,997.41	28	1.6%	0.04 [-0.49, 0.56]
Schuler 1999 (Moderate levels-H3-6 week)	66.7	92.68	31	62.3	58.89	13	1.5%	0.05 [-0.60, 0.70]
de Araujo 2015 (Influenza B-6 m post vaccination)	0.3	2.55	22	0.17	1.73	16	1.5%	0.06 [-0.59, 0.70]
Wong 2019 (A/California/07/2009-2-28 day)	419.3	297.36	14	401	268.53	11	1.3%	0.06 [-0.73, 0.85]
Schuler 1999 (Moderate fitness-H3-4 week)	39	62.91	23	35.1	39.06	31	1.6%	0.08 [-0.46, 0.62]
Wong 2019 (A/California/07/2009-1-28 day)	478.13	366.57	28	437.86	334.42	28	1.6%	0.11 [-0.41, 0.64]
de Araujo 2015 (H1N1-Moderate-6 week)	2.65	1.92	23	2.38	1.85	16	1.5%	0.14 [-0.50, 0.78]
Schuler 1999 (H3-1 week)	28.4	69.67	13	20.8	43.65	31	1.5%	0.14 [-0.51, 0.79]
Schuler 1999 (H3-6 week)	52	61.48	13	42.3	41.45	31	1.5%	0.20 [-0.45, 0.85]
Wong 2019 (A/Switzerland/97/2013-2-28 day)	2,556.88	1,715.98	14	2,193.26	1,786.68	11	1.3%	0.20 [-0.59, 0.99]
de Araujo 2015 (H1N1-6 m post vaccination)	1.18	3.43	22	0.59	1.33	16	1.5%	0.21 [-0.44, 0.86]
Schuler 1999 (Moderate fitness-H3-1 week)	29.7	37.97	23	20.8	43.65	31	1.6%	0.21 [-0.33, 0.75]
Ledo 2020 Brisbane	1.53	0.3	45	1.46	0.28	25	1.7%	0.24 [-0.25, 0.73]
de Araujo 2015 (H1N1-Moderate-6 month)	1.08	2.28	23	0.59	1.33	16	1.5%	0.25 [-0.39, 0.89]
Wong 2019 (B/Phuket/3073/2013-2-28 day)	2,063.17	1,774.63	14	1,542.44	1,910.17	11	1.3%	0.27 [-0.52, 1.07]
Schuler 1999 (H3a-2 week)	71.9	58.49	23	55.4	52.5	13	1.5%	0.29 [-0.40, 0.97]
Wong 2019 (A/Texas/50/2012-1-28 day)	4,144.44	2,319.4	26	3,379.6	2,614.43	28	1.6%	0.30 [-0.23, 0.84]
Schuler 1999 (H3-4 week)	52	71.57	13	35.1	39.06	31	1.5%	0.33 [-0.32, 0.98]
Ledo 2020 Phuket	1.54	0.31	45	1.42	0.41	25	1.7%	0.34 [-0.15, 0.83]
de Araujo 2015 (H3N2-Moderate-6 month)	1.21	2.42	23	0.43	1.85	16	1.5%	0.35 [-0.30, 0.99]
Schuler 1999 (Moderate fitness-H3-2 week)	68.2	92.77	23	43.9	41.05	31	1.6%	0.35 [-0.19, 0.90]
Schuler 1999 (H3a-1 week)	52.2	93.74	23	15.4	14.83	13	1.5%	0.47 [-0.22, 1.16]
Kohut 2002 (Infuenza-IgG-1:125-14 days)	1.05	0.12	16	0.98	0.13	15	1.4%	0.55 [-0.17, 1.26]
Kohut 2002 (Infuenza-IgM-1:125-14 days)	-0.39	0.53	16	-0.76	0.75	15	1.4%	0.56 [-0.16, 1.28]
Ledo 2020 H1N1	1.66	0.29	45	1.5	0.25	25	1.7%	0.57 [0.07, 1.07]
de Araujo 2015 (Influenza B-Moderate-6 week)	2.49	2.54	23	1.06	1.96	16	1.5%	0.60 [-0.05, 1.26]
de Araujo 2015 (H3N2-Moderate-6 week)	3.11	2.56	23	1.55	2.49	16	1.5%	0.60 [-0.05, 1.26]
Schuler 1999 (Moderate fitness-H3-6 week)	87.3	99.27	23	42.3	41.45	31	1.6%	0.62 [0.06, 1.17]
de Araujo 2015 (H3N2-6 m post vaccination)	2	2.64	22	0.43	1.85	16	1.5%	0.66 [-0.01, 1.32]
Ledo 2020 H3N2	1.62	0.3	45	1.4	0.35	25	1.7%	0.68 [0.18, 1.19]
de Araujo 2015 (Influenza B-6 w post vaccination)	2.83	2.54	22	1.06	1.96	16	1.5%	0.75 [0.08, 1.42]
de Araujo 2015 (H3N2-6 w post vaccination)	3.64	2.7	22	1.55	2.49	16	1.5%	0.78 [0.11, 1.45]
de Araujo 2015 (Influenza B-Moderate-6 month)	1.85	2.3	23	0.17	1.73	16	1.5%	0.79 [0.12, 1.45]
Kohut 2002 (Active-Infuenza-IgM-1:25-14 days)	0.48	0.34	16	0.06	0.55	15	1.4%	0.90 [0.16, 1.65]
Kohut 2002 (Infuenza-IgG-1:25-14 days)	1.2	0.06	16	1.14	0.04	15	1.4%	1.14 [0.37, 1.91]
Keylock 2007 (H3N2-6 week)	0.35	0.55	13	-0.88	0.73	13	1.2%	1.84 [0.90, 2.78]
Keylock 2007 (B Hong Kong/1434/2002-6 month)	0.37	0.44	13	-1.75	1.15	13	1.1%	2.36 [1.32, 3.39]
Keylock 2007 (B Hong Kong/1434/2002-6 week)	0.78	0.32	13	-0.6	0.72	13	1.1%	2.40 [1.35, 3.44]
Keylock 2007 (H1N1-6 month)	0.67	0.34	13	-0.83	0.71	13	1.0%	2.61 [1.52, 3.70]
Keylock 2007 (H1N1-6 week)	1.02	0.27	13	-0.27	0.52	13	1.0%	3.02 [1.84, 4.19]
Keylock 2007 (H3N2-6 month)	-0.004	0.69	13	-8.39	2.75	13	0.8%	4.05 [2.63, 5.47]
**Total (95% CI)**			**1536**			**1311**	**100.0%**	**0.18 [0.02, 0.34]**

Heterogeneity: $Tau^2 = 0.34$; $Chi^2 = 275.86$, df = 67 ($P < 0.00001$); $I^2 = 76\%$
Test for overall effect: Z = 2.21 (P = 0.03)

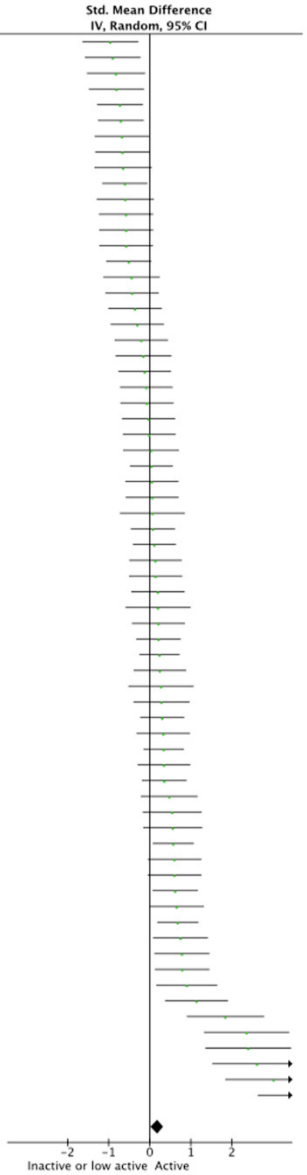

Supplementary Figure S21. Forest plot of the effects of physical activity levels on influenza antibodies. Δ scores: post intervention – baseline; SD: standard deviation; 95% CI: 95% confidence interval.

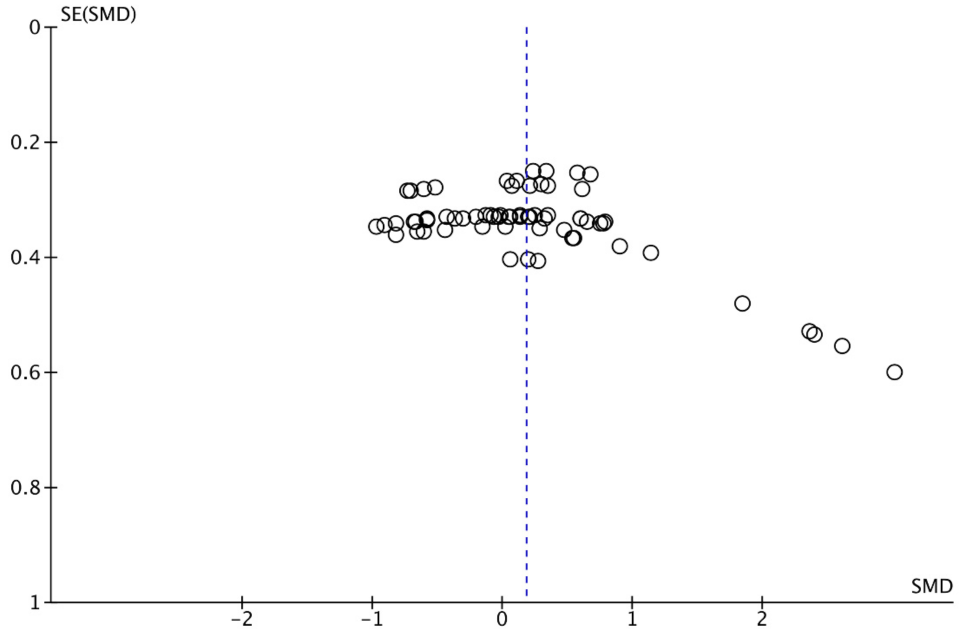

Supplementary Figure S22. Funnel plot of the effects of physical activity levels on influenza antibodies. SMD: standardized mean difference; SE: standard error.

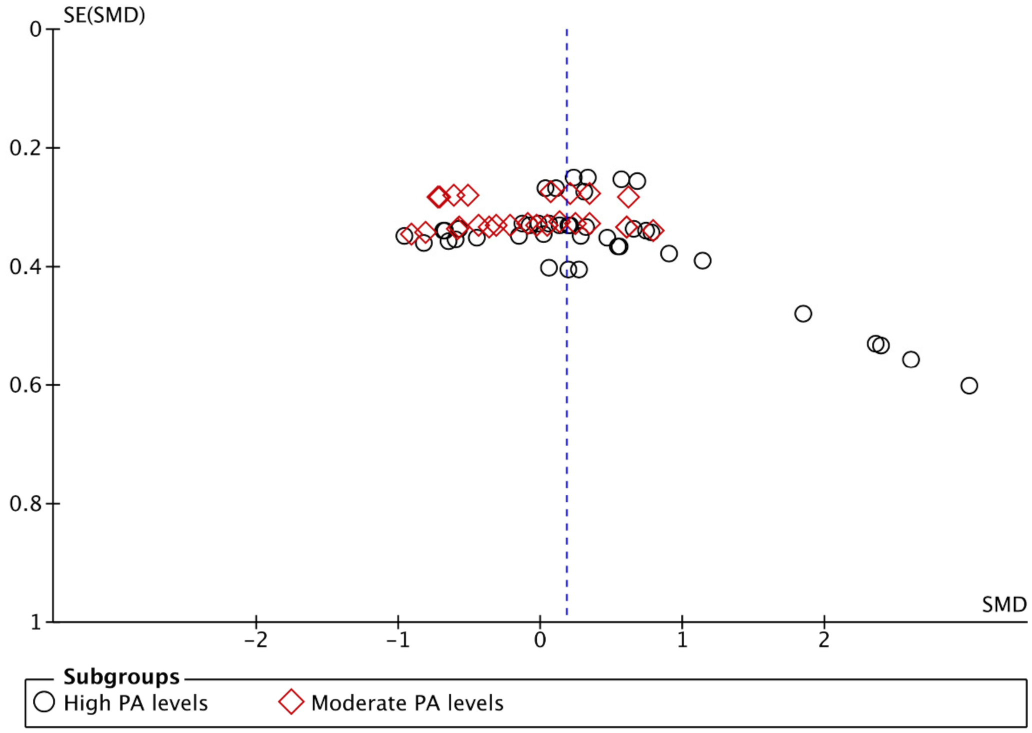

Supplementary Figure S23: Funnel plot of the effect of physical activity levels on influenza vaccine antibodies (sub-group analysis for high and moderate physical activity levels). SMD: standardized mean difference; SE: standard error.

Study or Subgroup	Active Δ scores Mean	SD	Total	Inactive/low act Δ scores Mean	SD	Total	Weight	Std. Mean Difference IV, Random, 95% CI
**3.6.1 <4 weeks**								
Schuler 1999 (H1-4 week)	2	29.57	13	46.1	49.97	31	1.5%	-0.96 [-1.64, -0.28]
Schuler 1999 (Moderate levels-H1-4 week)	8.5	36.11	31	43.2	41.69	13	1.5%	-0.90 [-1.58, -0.22]
Schuler 1999 (H1a-4 week)	7.5	42.9	23	43.2	41.69	13	1.4%	-0.82 [-1.53, -0.11]
Schuler 1999 (Moderate levels-H1-2 week)	3.3	28.58	31	29.9	39.86	13	1.5%	-0.81 [-1.48, -0.14]
Schuler 1999 (Moderate fitness-H1-1 week)	3.6	37.98	23	33.8	43.19	31	1.6%	-0.72 [-1.28, -0.17]
Schuler 1999 (Moderate fitness-H1-4 week)	13.2	40.07	23	46.1	49.97	31	1.6%	-0.70 [-1.26, -0.15]
Schuler 1999 (H1-2 week)	5.2	30.57	13	24.6	27.85	31	1.5%	-0.66 [-1.33, -0.00]
Schuler 1999 (H1a-2 week)	7.2	35.48	23	29.9	39.86	13	1.5%	-0.60 [-1.29, 0.10]
Schuler 1999 (H1-1 week)	9.2	38.79	13	33.8	43.19	31	1.5%	-0.58 [-1.24, 0.08]
Kohut 2002 (Moderate-Infuenza-IgM-1:125-14 days)	-1.2	0.75	25	-0.76	0.75	15	1.5%	-0.58 [-1.23, 0.08]
Schuler 1999 (Moderate fitness-H1-2 week)	7.5	38.8	23	24.6	27.85	31	1.6%	-0.51 [-1.06, 0.04]
Schuler 1999 (H3a-4 week)	37.9	34.97	23	64.5	86.25	13	1.5%	-0.44 [-1.13, 0.24]
Kohut 2002 (Moderate-Infuenza-IgG-1:25-14 days)	1.11	0.08	25	1.14	0.04	15	1.5%	-0.43 [-1.08, 0.22]
Schuler 1999 (Moderate levels-H3-4 week)	38.5	64.38	31	64.5	86.25	13	1.5%	-0.36 [-1.01, 0.29]
Schuler 1999 (Moderate levels-H3-2 week)	39.7	49.69	31	55.4	52.5	13	1.5%	-0.31 [-0.96, 0.35]
Schuler 1999 (Moderate levels-H1-1 week)	9	43.6	31	17.2	24.38	13	1.5%	-0.21 [-0.86, 0.44]
Schuler 1999 (H1a-1 week)	10.5	49.53	23	17.2	24.38	13	1.5%	-0.15 [-0.84, 0.53]
Kohut 2002 (Infuenza-IgM-1:25-14 days)	-0.01	0.56	25	0.06	0.55	15	1.5%	-0.12 [-0.76, 0.52]
Kohut 2002 (Moderate-Infuenza-IgG-1:125-14 days)	0.97	0.11	25	0.98	0.13	15	1.5%	-0.08 [-0.72, 0.56]
Schuler 1999 (H3-2 week)	40.8	52.49	13	43.9	41.05	31	1.5%	-0.07 [-0.72, 0.58]
Schuler 1999 (Moderate levels-H3-1 week)	14.4	39.7	31	15.4	14.83	13	1.5%	-0.03 [-0.68, 0.62]
Wong 2019 (B/Massachusetts/02/2012-1-28 day)	3,595.34	2,106.52	28	3,515.91	1,997.41	28	1.6%	0.04 [-0.49, 0.56]
Wong 2019 (A/California/07/2009-2-28 day)	419.3	297.36	14	401	268.53	11	1.3%	0.06 [-0.73, 0.85]
Schuler 1999 (Moderate fitness-H3-4 week)	39	62.91	23	35.1	39.06	31	1.6%	0.08 [-0.46, 0.62]
Wong 2019 (A/California/07/2009-1-28 day)	478.13	366.57	28	437.86	334.42	28	1.6%	0.11 [-0.41, 0.64]
Schuler 1999 (H3-1 week)	28.4	69.67	13	20.8	43.65	31	1.5%	0.14 [-0.51, 0.79]
Wong 2019 (A/Switzerland/97/2013-2-28 day)	2,556.88	1,715.98	14	2,193.26	1,786.68	11	1.3%	0.20 [-0.59, 0.99]
Schuler 1999 (Moderate fitness-H3-1 week)	29.7	37.97	23	20.8	43.65	31	1.6%	0.21 [-0.33, 0.75]
Ledo 2020 Brisbane	1.53	0.3	45	1.46	0.28	25	1.7%	0.24 [-0.25, 0.73]
Wong 2019 (B/Phuket/3073/2013-2-28 day)	2,063.17	1,774.63	14	1,542.44	1,910.17	11	1.3%	0.27 [-0.52, 1.07]
Schuler 1999 (H3a-2 week)	71.9	58.49	23	55.4	52.5	13	1.5%	0.29 [-0.40, 0.97]
Wong 2019 (A/Texas/50/2012-1-28 day)	4,144.44	2,319.4	26	3,379.6	2,614.43	28	1.6%	0.30 [-0.23, 0.84]
Schuler 1999 (H3-4 week)	52	71.57	13	35.1	39.06	31	1.5%	0.33 [-0.32, 0.98]
Ledo 2020 Phuket	1.54	0.31	45	1.42	0.41	25	1.7%	0.34 [-0.15, 0.83]
Schuler 1999 (Moderate fitness-H3-2 week)	68.2	92.77	23	43.9	41.05	31	1.6%	0.35 [-0.19, 0.90]
Schuler 1999 (H3a-1 week)	52.2	93.74	23	15.4	14.83	13	1.5%	0.47 [-0.22, 1.16]
Kohut 2002 (Infuenza-IgG-1:125-14 days)	1.05	0.12	16	0.98	0.13	15	1.4%	0.55 [-0.17, 1.26]
Kohut 2002 (Infuenza-IgM-1:125-14 days)	-0.39	0.53	16	-0.76	0.75	15	1.4%	0.56 [-0.16, 1.28]
Ledo 2020 H1N1	1.66	0.29	45	1.5	0.25	25	1.7%	0.57 [0.07, 1.07]
Ledo 2020 H3N2	1.62	0.3	45	1.4	0.35	25	1.7%	0.68 [0.18, 1.19]
Kohut 2002 (Active-Infuenza-IgM-1:25-14 days)	0.48	0.34	16	0.06	0.55	15	1.4%	0.90 [0.16, 1.65]
Kohut 2002 (Infuenza-IgG-1:25-14 days)	1.2	0.06	16	1.14	0.04	15	1.4%	1.14 [0.37, 1.91]
**Subtotal (95% CI)**			**1008**			**865**	**63.7%**	**-0.05 [-0.20, 0.10]**
Heterogeneity: $Tau^2 = 0.15$; $Chi^2 = 101.02$, df = 41 ($P < 0.00001$); $I^2 = 59\%$								
Test for overall effect: Z = 0.65 (P = 0.51)								
**3.6.2 >4 weeks**								
Schuler 1999 (H1-6 week)	-4.4	27.5	13	14.6	27.59	31	1.5%	-0.68 [-1.34, -0.01]
Schuler 1999 (H1a-6 week)	-6.6	32.01	23	13.9	28.73	13	1.4%	-0.65 [-1.35, 0.05]
Schuler 1999 (Moderate fitness-H1-6 week)	-4.3	34.62	23	14.6	27.59	31	1.6%	-0.61 [-1.16, -0.05]
Schuler 1999 (Moderate levels-H1-6 week)	-3.8	30.56	31	13.9	28.73	13	1.5%	-0.58 [-1.24, 0.08]
de Araujo 2015 (H1N1-6 w post vaccination)	2.35	2.65	22	2.38	1.85	16	1.5%	-0.01 [-0.66, 0.63]
Schuler 1999 (H3a-6 week)	64.3	70.17	23	62.3	58.89	13	1.5%	0.03 [-0.65, 0.71]
Schuler 1999 (Moderate levels-H3-6 week)	66.7	92.68	31	62.3	58.89	13	1.5%	0.05 [-0.60, 0.70]
de Araujo 2015 (Influenza B-6 m post vaccination)	0.3	2.55	22	0.17	1.73	16	1.5%	0.06 [-0.59, 0.70]
de Araujo 2015 (H1N1-Moderate-6 week)	2.65	1.92	23	2.38	1.85	16	1.5%	0.14 [-0.50, 0.78]
Schuler 1999 (H3-6 week)	52	61.48	13	42.3	41.45	31	1.5%	0.20 [-0.45, 0.85]
de Araujo 2015 (H1N1-6 m post vaccination)	1.18	3.43	22	0.59	1.33	16	1.5%	0.21 [-0.44, 0.86]
de Araujo 2015 (H1N1-Moderate-6 month)	1.08	2.28	23	0.59	1.33	16	1.5%	0.25 [-0.39, 0.89]
de Araujo 2015 (H3N2-Moderate-6 month)	1.21	2.42	23	0.43	1.85	16	1.5%	0.35 [-0.30, 0.99]
de Araujo 2015 (Influenza B-Moderate-6 week)	2.49	2.54	23	1.06	1.96	16	1.5%	0.60 [-0.05, 1.26]
de Araujo 2015 (H3N2-Moderate-6 week)	3.11	2.56	23	1.55	2.49	16	1.5%	0.60 [-0.05, 1.26]
Schuler 1999 (Moderate fitness-H3-6 week)	87.3	99.27	23	42.3	41.45	31	1.6%	0.62 [0.06, 1.17]
de Araujo 2015 (H3N2-6 m post vaccination)	2	2.64	22	0.43	1.85	16	1.5%	0.66 [-0.01, 1.32]
de Araujo 2015 (Influenza B-6 w post vaccination)	2.83	2.54	22	1.06	1.96	16	1.5%	0.75 [0.08, 1.42]
de Araujo 2015 (H3N2-6 w post vaccination)	3.64	2.7	22	1.55	2.49	16	1.5%	0.78 [0.11, 1.45]
de Araujo 2015 (Influenza B-Moderate-6 month)	1.85	2.3	23	0.17	1.73	16	1.5%	0.79 [0.12, 1.45]
Keylock 2007 (H3N2-6 week)	0.35	0.55	13	-0.88	0.73	13	1.2%	1.84 [0.90, 2.78]
Keylock 2007 (B Hong Kong/1434/2002-6 month)	0.37	0.44	13	-1.75	1.15	13	1.1%	2.36 [1.32, 3.39]
Keylock 2007 (B Hong Kong/1434/2002-6 week)	0.78	0.32	13	-0.6	0.72	13	1.1%	2.40 [1.35, 3.44]
Keylock 2007 (H1N1-6 month)	0.67	0.34	13	-0.83	0.71	13	1.0%	2.61 [1.52, 3.70]
Keylock 2007 (H1N1-6 week)	1.02	0.27	13	-0.27	0.52	13	1.0%	3.02 [1.84, 4.19]
Keylock 2007 (H3N2-6 month)	-0.004	0.69	13	-8.39	2.75	13	0.8%	4.05 [2.63, 5.47]
**Subtotal (95% CI)**			**528**			**446**	**36.3%**	**0.64 [0.30, 0.98]**
Heterogeneity: $Tau^2 = 0.63$; $Chi^2 = 148.94$, df = 25 ($P < 0.00001$); $I^2 = 83\%$								
Test for overall effect: Z = 3.72 (P = 0.0002)								
**Total (95% CI)**			**1536**			**1311**	**100.0%**	**0.18 [0.02, 0.34]**
Heterogeneity: $Tau^2 = 0.34$; $Chi^2 = 275.86$, df = 67 ($P < 0.00001$); $I^2 = 76\%$								
Test for overall effect: Z = 2.21 (P = 0.03)								
Test for subgroup differences: $Chi^2 = 13.40$, df = 1 (P = 0.0003), $I^2 = 92.5\%$								

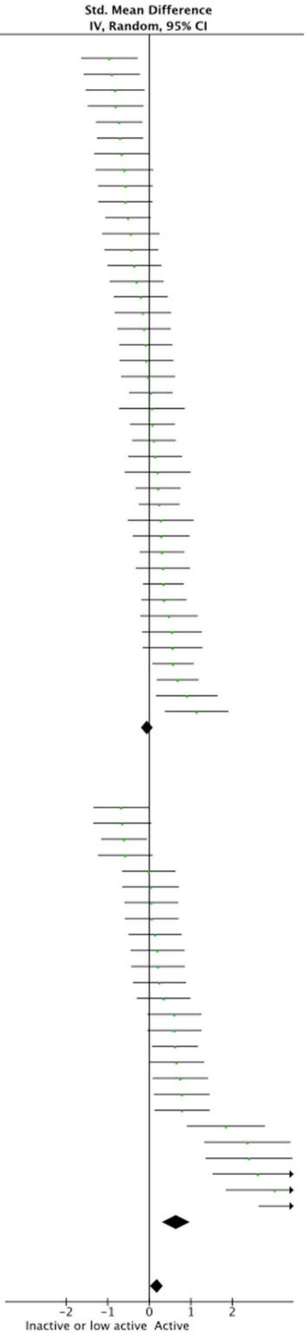

Supplementary Figure S24. Forest plot of the effects of physical activity levels on influenza antibodies (subgroup analysis for high vs. moderate physical activity levels). Δ scores: post intervention – baseline; SD: standard deviation; 95% CI: 95% confidence interval.

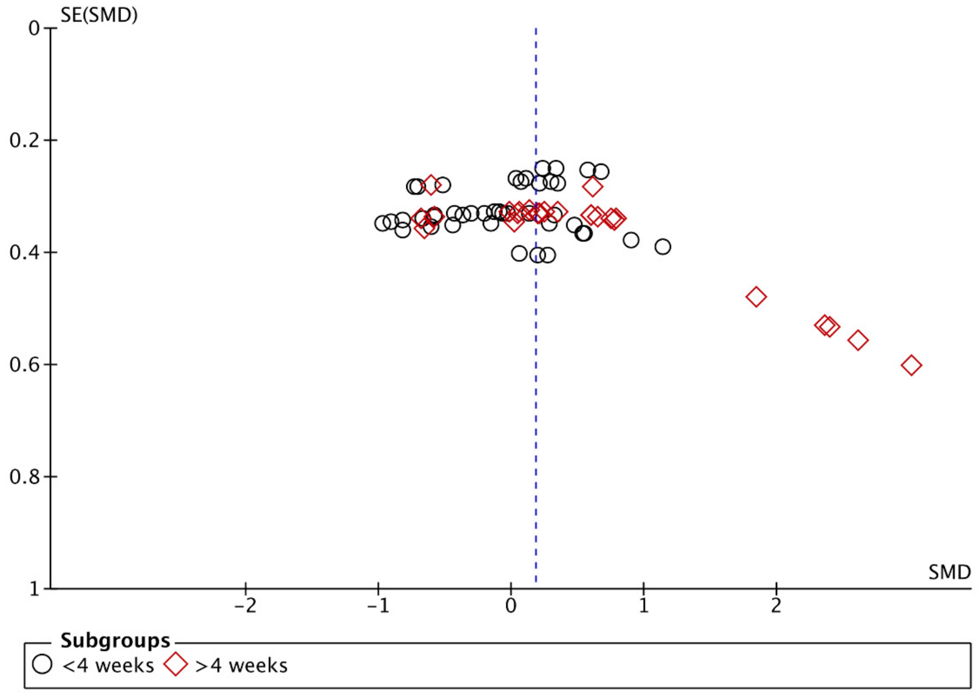

Supplementary Figure S25. Funnel plot of the effects of physical activity levels on influenza antibodies (subgroup analysis for high vs. moderate physical activity levels). SMD: standardized mean difference; SE: standard error.

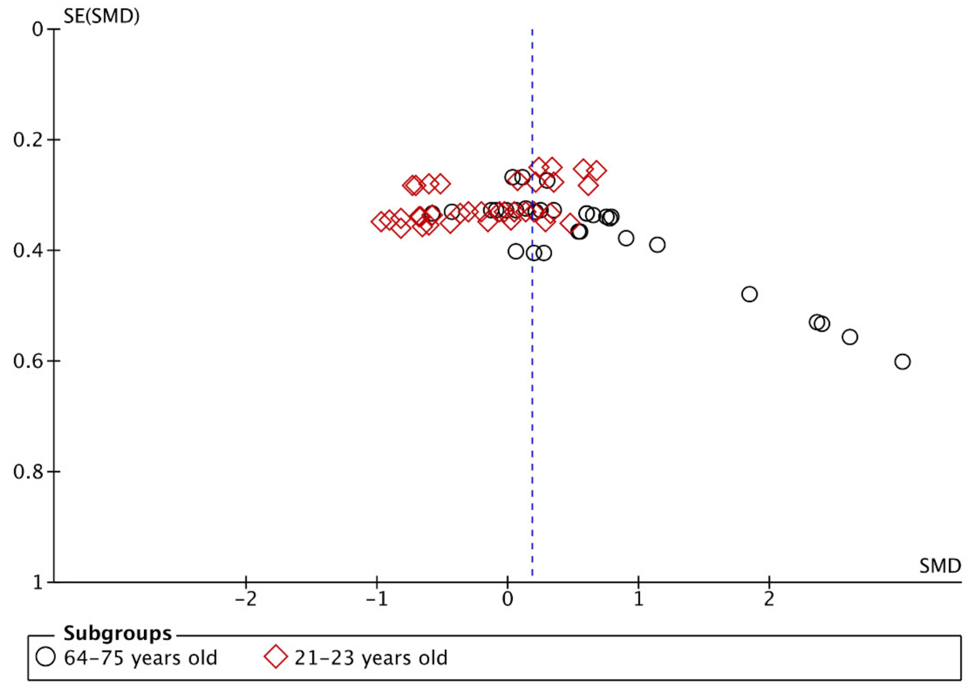

Supplementary Figure S26: Funnel plot of the effect of physical activity levels on influenza vaccine antibodies (subgroup analysis for age). SMD: standardized mean difference; SE: standard error.

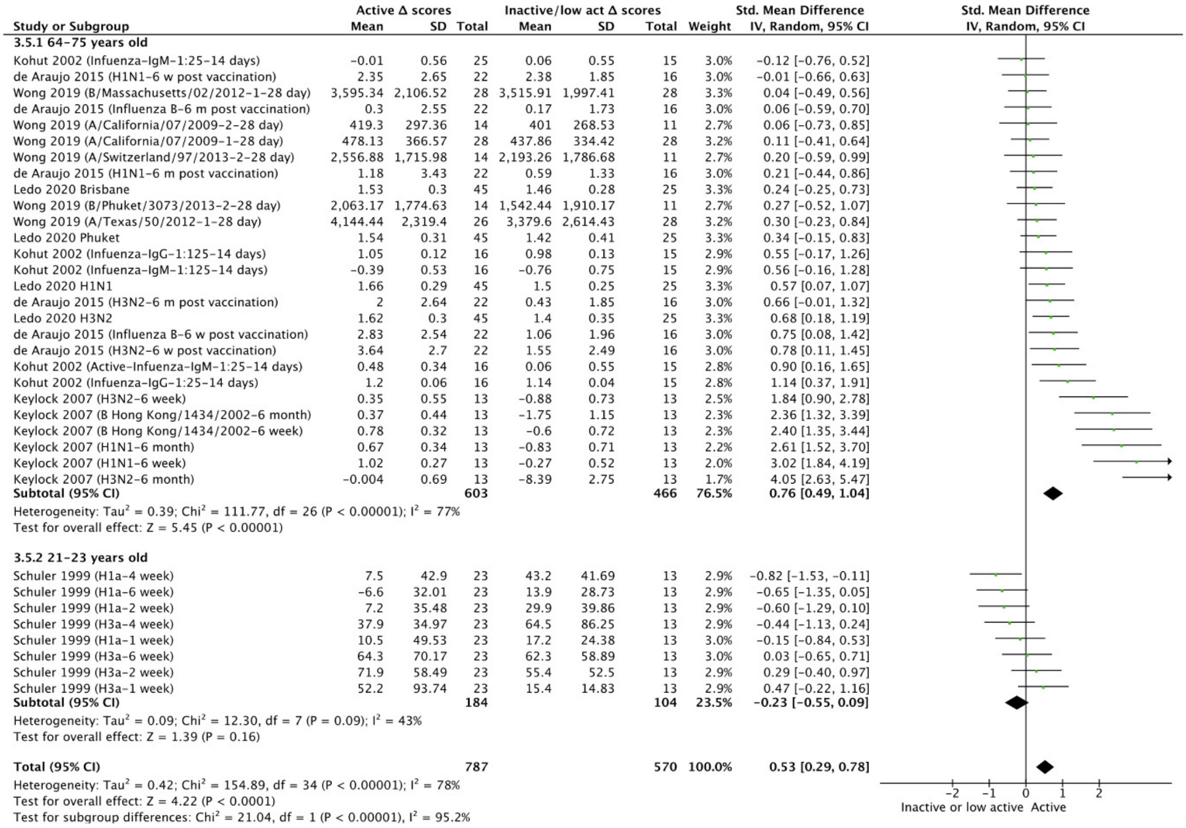

Supplementary Figure S27. Forest plot of the effects of high physical activity levels on influenza vaccine antibodies (subgroup analysis for age). Δ scores: post intervention – baseline; SD: standard deviation; 95% CI: 95% confidence interval.

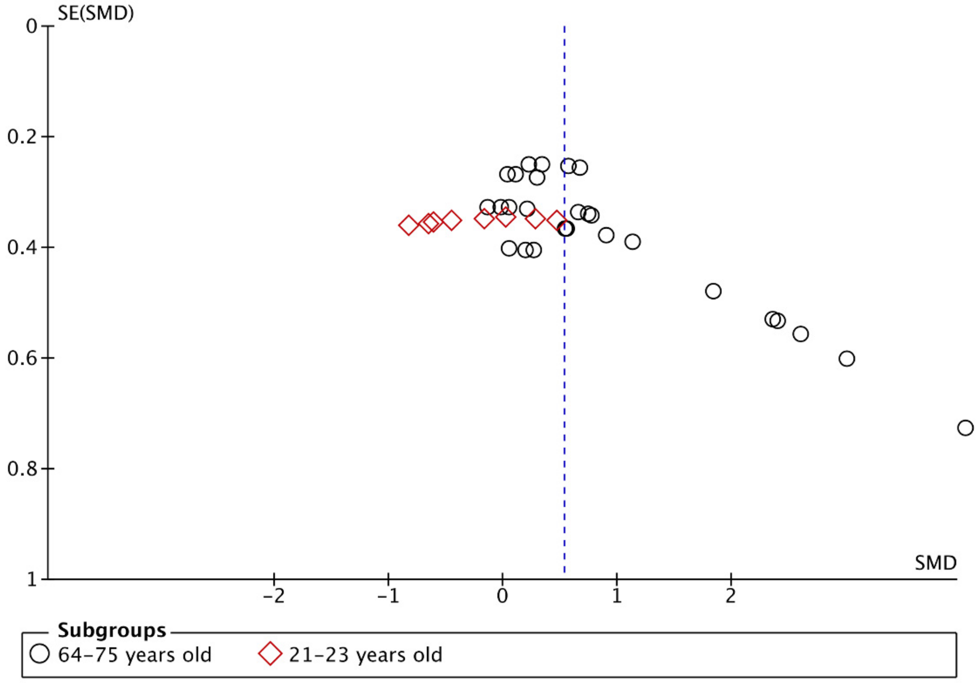

Supplementary Figure S28. Funnel plot of the effects of high physical activity levels on influenza vaccine antibodies (subgroup analysis for age). SMD: standardized mean difference; SE: standard error.

Study or Subgroup	Active Δ scores Mean	SD	Total	Inactive/low act Δ scores Mean	SD	Total	Weight	Std. Mean Difference IV, Random, 95% CI
**3.7.1 H1N1**								
Schuler 1999 (H1-4 week)	2	29.57	13	46.1	49.97	31	1.5%	-0.96 [-1.64, -0.28]
Schuler 1999 (Moderate levels-H1-4 week)	8.5	36.11	31	43.2	41.69	13	1.5%	-0.90 [-1.58, -0.22]
Schuler 1999 (H1a-4 week)	7.5	42.9	23	43.2	41.69	13	1.4%	-0.82 [-1.53, -0.11]
Schuler 1999 (Moderate levels-H1-2 week)	3.3	28.58	31	29.9	39.86	13	1.5%	-0.81 [-1.48, -0.14]
Schuler 1999 (Moderate fitness-H1-1 week)	3.6	37.98	23	33.8	43.19	31	1.6%	-0.72 [-1.28, -0.17]
Schuler 1999 (Moderate fitness-H1-4 week)	13.2	40.07	23	46.1	49.97	31	1.6%	-0.70 [-1.26, -0.15]
Schuler 1999 (H1-6 week)	-4.4	27.5	13	14.6	27.59	31	1.5%	-0.68 [-1.34, -0.01]
Schuler 1999 (H1-2 week)	5.2	30.57	13	24.6	27.85	31	1.5%	-0.66 [-1.33, -0.00]
Schuler 1999 (H1a-6 week)	-6.6	32.01	23	13.9	28.73	13	1.4%	-0.65 [-1.35, 0.05]
Schuler 1999 (Moderate fitness-H1-6 week)	-4.3	34.62	23	14.6	27.59	31	1.6%	-0.61 [-1.16, -0.05]
Schuler 1999 (H1a-2 week)	7.2	35.48	23	29.9	39.86	13	1.5%	-0.60 [-1.29, 0.10]
Schuler 1999 (Moderate levels-H1-6 week)	-3.8	30.56	31	13.9	28.73	13	1.5%	-0.58 [-1.24, 0.08]
Schuler 1999 (H1-1 week)	9.2	38.79	13	33.8	43.19	31	1.5%	-0.58 [-1.24, 0.08]
Schuler 1999 (Moderate fitness-H1-2 week)	7.5	38.8	23	24.6	27.85	31	1.6%	-0.51 [-1.06, 0.04]
Schuler 1999 (Moderate levels-H1-1 week)	9	43.6	31	17.2	24.38	13	1.5%	-0.21 [-0.86, 0.44]
Schuler 1999 (H1a-1 week)	10.5	49.53	23	17.2	24.38	13	1.5%	-0.15 [-0.84, 0.53]
de Araujo 2015 (H1N1-6 w post vaccination)	2.35	2.65	22	2.38	1.85	16	1.5%	-0.01 [-0.66, 0.63]
Wong 2019 (A/California/07/2009-2-28 day)	419.3	297.36	14	401	268.53	11	1.3%	0.06 [-0.73, 0.85]
Wong 2019 (A/California/07/2009-1-28 day)	478.13	366.57	28	437.86	334.42	28	1.6%	0.11 [-0.41, 0.64]
de Araujo 2015 (H1N1-Moderate-6 week)	2.65	1.92	23	2.38	1.85	16	1.5%	0.14 [-0.50, 0.78]
de Araujo 2015 (H1N1-6 m post vaccination)	1.18	3.43	22	0.59	1.33	16	1.5%	0.21 [-0.44, 0.86]
de Araujo 2015 (H1N1-Moderate-6 month)	1.08	2.28	23	0.59	1.33	16	1.5%	0.25 [-0.39, 0.89]
Ledo 2020 H1N1	1.66	0.29	45	1.5	0.25	25	1.7%	0.57 [0.07, 1.07]
Keylock 2007 (H1N1-6 month)	0.67	0.34	13	-0.83	0.71	13	1.0%	2.61 [1.52, 3.70]
Keylock 2007 (H1N1-6 week)	1.02	0.27	13	-0.27	0.52	13	1.0%	3.02 [1.84, 4.19]
**Subtotal (95% CI)**			**563**			**506**	**36.9%**	**-0.20 [-0.47, 0.07]**
Heterogeneity: $Tau^2 = 0.36$; $Chi^2 = 103.15$, df = 24 ($P < 0.00001$); $I^2 = 77\%$								
Test for overall effect: Z = 1.46 (P = 0.14)								
**3.7.2 H3N2**								
Schuler 1999 (H3a-4 week)	37.9	34.97	23	64.5	86.25	13	1.5%	-0.44 [-1.13, 0.24]
Schuler 1999 (Moderate levels-H3-4 week)	38.5	64.38	31	64.5	86.25	13	1.5%	-0.36 [-1.01, 0.29]
Schuler 1999 (Moderate levels-H3-2 week)	39.7	49.69	31	55.4	52.5	13	1.5%	-0.31 [-0.96, 0.35]
Schuler 1999 (H3-2 week)	40.8	52.49	13	43.9	41.05	31	1.5%	-0.07 [-0.72, 0.58]
Schuler 1999 (Moderate levels-H3-1 week)	14.4	39.7	31	15.4	14.83	13	1.5%	-0.03 [-0.68, 0.62]
Schuler 1999 (H3a-6 week)	64.3	70.17	23	62.3	58.89	13	1.5%	0.03 [-0.65, 0.71]
Schuler 1999 (Moderate levels-H3-6 week)	66.7	92.68	31	62.3	58.89	13	1.5%	0.05 [-0.60, 0.70]
Schuler 1999 (Moderate fitness-H3-4 week)	39	62.91	23	35.1	39.06	31	1.6%	0.08 [-0.46, 0.62]
Schuler 1999 (H3-1 week)	28.4	69.67	13	20.8	43.65	31	1.5%	0.14 [-0.51, 0.79]
Schuler 1999 (H3-6 week)	52	61.48	13	42.3	41.45	31	1.5%	0.20 [-0.45, 0.85]
Wong 2019 (A/Switzerland/97/2013-2-28 day)	2,556.88	1,715.98	14	2,193.26	1,786.68	11	1.3%	0.20 [-0.59, 0.99]
Schuler 1999 (Moderate fitness-H3-1 week)	29.7	37.97	23	20.8	43.65	31	1.6%	0.21 [-0.33, 0.75]
Schuler 1999 (H3a-2 week)	71.9	58.49	23	55.4	52.5	13	1.5%	0.29 [-0.40, 0.97]
Wong 2019 (A/Texas/50/2012-1-28 day)	4,144.44	2,319.4	26	3,379.6	2,614.43	28	1.6%	0.30 [-0.23, 0.84]
Schuler 1999 (H3-4 week)	52	71.57	13	35.1	39.06	31	1.5%	0.33 [-0.32, 0.98]
de Araujo 2015 (H3N2-Moderate-6 month)	1.21	2.42	23	0.43	1.85	16	1.5%	0.35 [-0.30, 0.99]
Schuler 1999 (Moderate fitness-H3-2 week)	68.2	92.77	23	43.9	41.05	31	1.6%	0.35 [-0.19, 0.90]
Schuler 1999 (H3a-1 week)	52.2	93.74	23	15.4	14.83	13	1.5%	0.47 [-0.22, 1.16]
de Araujo 2015 (H3N2-Moderate-6 week)	3.11	2.56	23	1.55	2.49	16	1.5%	0.60 [-0.05, 1.26]
Schuler 1999 (Moderate fitness-H3-6 week)	87.3	99.27	23	42.3	41.45	31	1.6%	0.62 [0.06, 1.17]
de Araujo 2015 (H3N2-6 m post vaccination)	2	2.64	22	0.43	1.85	16	1.5%	0.66 [-0.01, 1.32]
Ledo 2020 H3N2	1.62	0.3	45	1.4	0.35	25	1.7%	0.68 [0.18, 1.19]
de Araujo 2015 (H3N2-6 w post vaccination)	3.64	2.7	22	1.55	2.49	16	1.5%	0.78 [0.11, 1.45]
Keylock 2007 (H3N2-6 week)	0.35	0.55	13	-0.88	0.73	13	1.2%	1.84 [0.90, 2.78]
Keylock 2007 (H3N2-6 month)	-0.004	0.69	13	-8.39	2.75	13	0.8%	4.05 [2.63, 5.47]
**Subtotal (95% CI)**			**561**			**506**	**37.0%**	**0.34 [0.13, 0.54]**
Heterogeneity: $Tau^2 = 0.17$; $Chi^2 = 61.08$, df = 24 ($P < 0.0001$); $I^2 = 61\%$								
Test for overall effect: Z = 3.19 (P = 0.001)								
**3.7.3 B**								
Wong 2019 (B/Massachusetts/02/2012-1-28 day)	3,595.34	2,106.52	28	3,515.91	1,997.41	28	1.6%	0.04 [-0.49, 0.56]
de Araujo 2015 (Influenza B-6 m post vaccination)	0.3	2.55	22	0.17	1.73	16	1.5%	0.06 [-0.59, 0.70]
Ledo 2020 Brisbane	1.53	0.3	45	1.46	0.28	25	1.7%	0.24 [-0.25, 0.73]
Wong 2019 (B/Phuket/3073/2013-2-28 day)	2,063.17	1,774.63	14	1,542.44	1,910.17	11	1.3%	0.27 [-0.52, 1.07]
Ledo 2020 Phuket	1.54	0.31	45	1.42	0.41	25	1.7%	0.34 [-0.15, 0.83]
de Araujo 2015 (Influenza B-Moderate-6 week)	2.49	2.54	23	1.06	1.96	16	1.5%	0.60 [-0.05, 1.26]
de Araujo 2015 (Influenza B-6 w post vaccination)	2.83	2.54	22	1.06	1.96	16	1.5%	0.75 [0.08, 1.42]
de Araujo 2015 (Influenza B-Moderate-6 month)	1.85	2.3	23	0.17	1.73	16	1.5%	0.79 [0.12, 1.45]
Keylock 2007 (B Hong Kong/1434/2002-6 month)	0.37	0.44	13	-1.75	1.15	13	1.1%	2.36 [1.32, 3.39]
Keylock 2007 (B Hong Kong/1434/2002-6 week)	0.78	0.32	13	-0.6	0.72	13	1.1%	2.40 [1.35, 3.44]
**Subtotal (95% CI)**			**248**			**179**	**14.5%**	**0.67 [0.27, 1.07]**
Heterogeneity: $Tau^2 = 0.29$; $Chi^2 = 33.00$, df = 9 ($P = 0.0001$); $I^2 = 73\%$								
Test for overall effect: Z = 3.28 (P = 0.001)								
**3.7.4 All strains**								
Kohut 2002 (Moderate-Infuenza-IgM-1:125-14 days)	-1.2	0.75	25	-0.76	0.75	15	1.5%	-0.58 [-1.23, 0.08]
Kohut 2002 (Moderate-Infuenza-IgG-1:25-14 days)	1.11	0.08	25	1.14	0.04	15	1.5%	-0.43 [-1.08, 0.22]
Kohut 2002 (Infuenza-IgM-1:25-14 days)	-0.01	0.56	25	0.06	0.55	15	1.5%	-0.12 [-0.76, 0.52]
Kohut 2002 (Moderate-Infuenza-IgG-1:125-14 days)	0.97	0.11	25	0.98	0.13	15	1.5%	-0.08 [-0.72, 0.56]
Kohut 2002 (Infuenza-IgG-1:125-14 days)	1.05	0.12	16	0.98	0.13	15	1.4%	0.55 [-0.17, 1.26]
Kohut 2002 (Infuenza-IgM-1:125-14 days)	-0.39	0.53	16	-0.76	0.75	15	1.4%	0.56 [-0.16, 1.28]
Kohut 2002 (Active-Infuenza-IgM-1:25-14 days)	0.48	0.34	16	0.06	0.55	15	1.4%	0.90 [0.16, 1.65]
Kohut 2002 (Infuenza-IgG-1:25-14 days)	1.2	0.06	16	1.14	0.04	15	1.4%	1.14 [0.37, 1.91]
**Subtotal (95% CI)**			**164**			**120**	**11.7%**	**0.22 [-0.21, 0.65]**
Heterogeneity: $Tau^2 = 0.26$; $Chi^2 = 21.71$, df = 7 ($P = 0.003$); $I^2 = 68\%$								
Test for overall effect: Z = 0.99 (P = 0.32)								
**Total (95% CI)**			**1536**			**1311**	**100.0%**	**0.18 [0.02, 0.34]**
Heterogeneity: $Tau^2 = 0.34$; $Chi^2 = 275.86$, df = 67 ($P < 0.00001$); $I^2 = 76\%$								
Test for overall effect: Z = 2.21 (P = 0.03)								
Test for subgroup differences: $Chi^2 = 15.39$, df = 3 ($P = 0.002$), $I^2 = 80.5\%$								

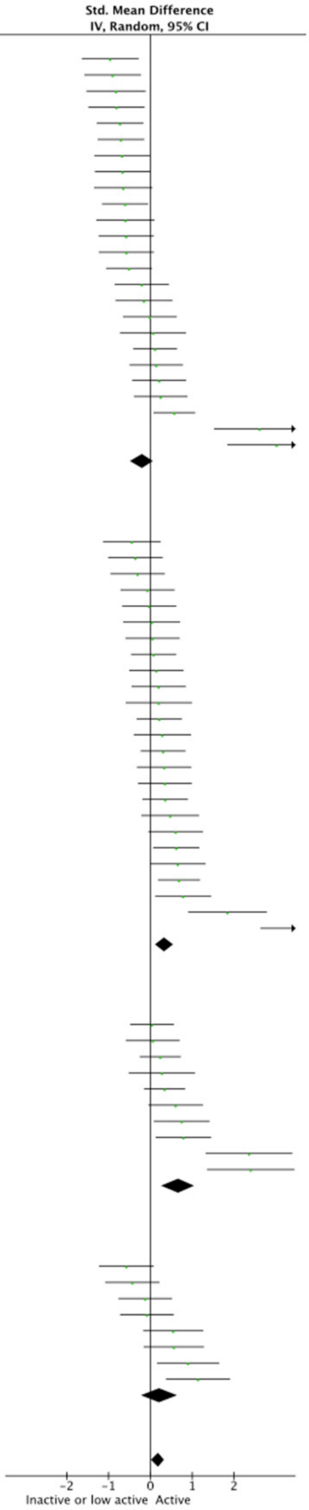

Supplementary Figure S29. Forest plot for the effect of physical activity levels on influenza vaccine antibodies (subgroup for antibodies type). Δ scores: post intervention – baseline; SD: standard deviation; 95% CI: 95% confidence interval.

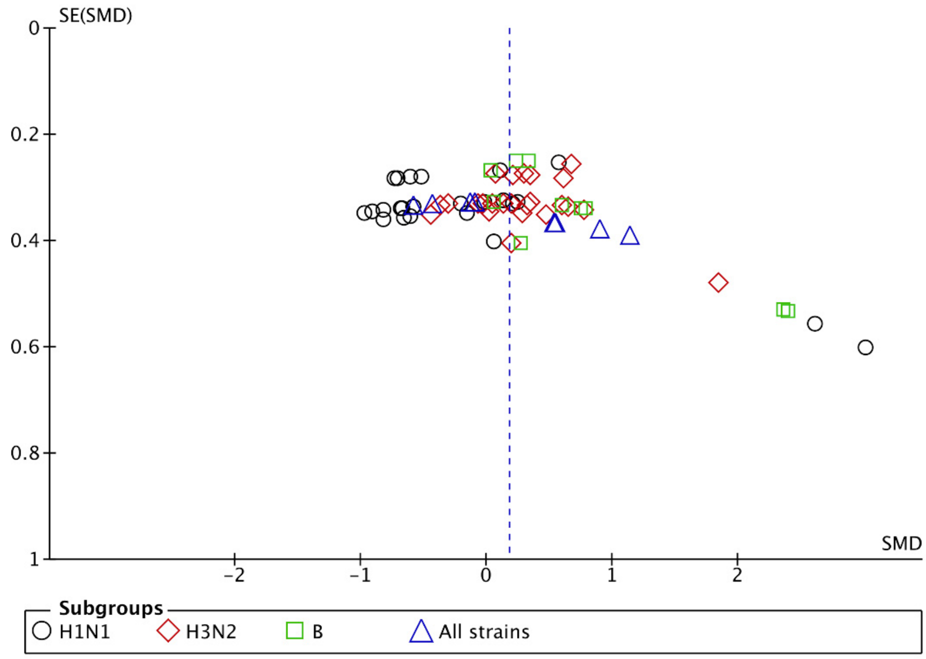

Supplementary Figure S30. Funnel plot for the effect of physical activity levels on influenza vaccine antibodies (subgroup for antibodies type). SMD: standardized mean difference; SE: standard error.

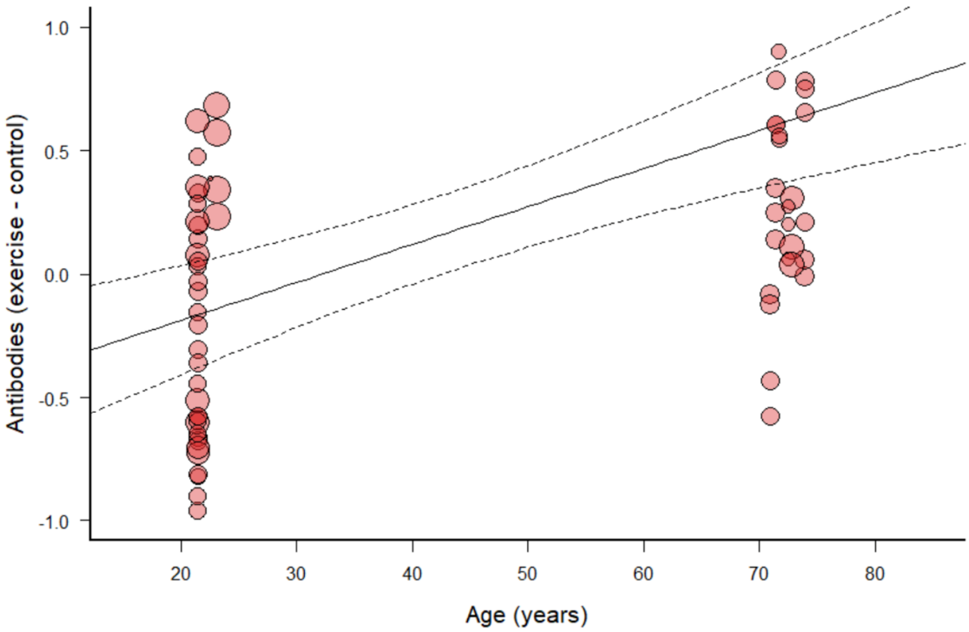

tau^2 (estimated amount of residual heterogeneity):	0.3398
tau (square root of estimated tau^2 value):	0.5829
I^2 (residual heterogeneity / unaccounted variability):	75.32 %
H^2 (unaccounted variability / sampling variability):	4.05 %
R^2 (amount of heterogeneity accounted for):	31.23 %

Test of Moderators (coefficient 2) (p value): 0.000003059

Supplementary Figure S31. Plot of meta-regression analysis of antibodies development of physically active individuals, in response to influenza vaccination in relation to age.

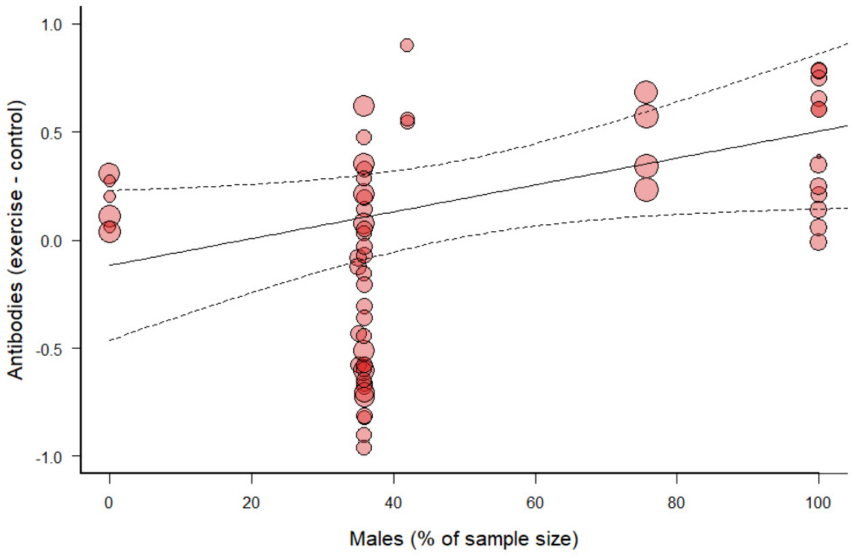

tau² (estimated amount of residual heterogeneity): 0.4525
tau (square root of estimated tau² value): 0.6727
I² (residual heterogeneity / unaccounted variability): 80.24 %
H² (unaccounted variability / sampling variability): 5.06 %
R² (amount of heterogeneity accounted for): 8.42 %

Test of Moderators (coefficient 2) (p value): 0.0462

Supplementary Figure S32. Plot of meta-regression analysis of antibodies development of physically active individuals, in response to influenza vaccination in relation to male gender.

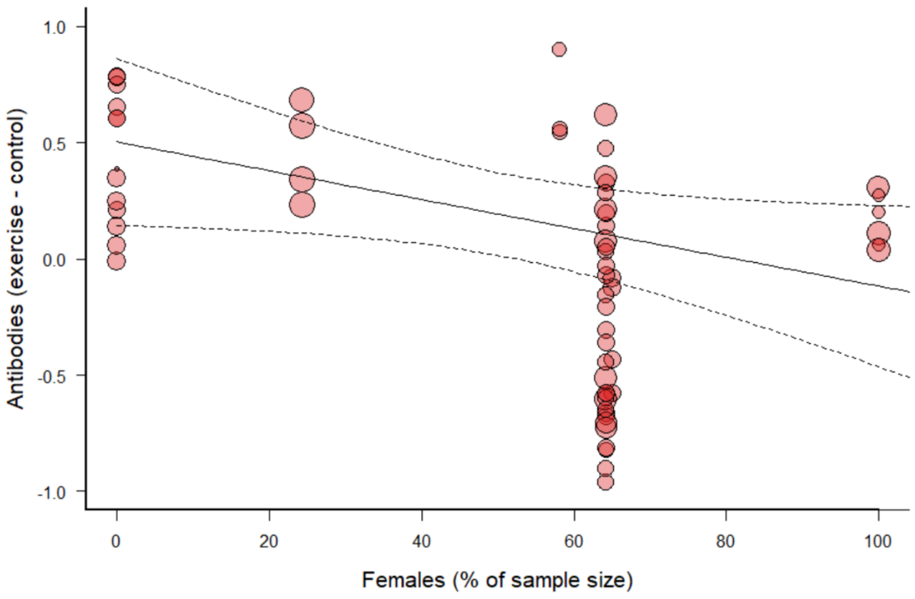

tau² (estimated amount of residual heterogeneity): 0.4525
tau (square root of estimated tau² value): 0.6727
I² (residual heterogeneity / unaccounted variability): 80.24 %
H² (unaccounted variability / sampling variability): 5.06 %
R² (amount of heterogeneity accounted for): 8.42 %

Test of Moderators (coefficient 2) (p value): 0.0462

Supplementary Figure S33. Plot of meta-regression analysis of antibodies development of physically active individuals, in response to influenza vaccination in relation to female gender.

Supplementary Table S3. GRADE analysis.

		Evaluation components to lower quality						Evaluation components to higher quality		
GRADE outcome	Meta-analysis outcome	Methodological design, start point	Risk of bias	Inconsistency of results	Indirectness	Imprecision	Publication bias	Large effect	Dose response	Confounding
Moderate ⊕⊕⊕◯	Effect of chronic exercise (all types) on influenza vaccine antibodies	Randomized controlled trials and controlled trials: Moderate quality	25 components of the included studies display low risk of bias and 24 components some concerns (RoB #1 sheet). High risk of bias is only displayed for randomization process of 2 studies and for outcome and reported results of 1 study. These high risk of bias components is unlikely to increase the overall risk of bias of all 9 included studies. No downgrade	Even though we used a random effect model meta-analysis, we consider heterogeneity as an index of inconsistency. I2=90%, p<0.01, substantial heterogeneity. However, we controlled baseline differences of the measured antibodies of the participants by calculating Δ scores with post measurements data for antibodies. This approach removed the likelihood for inconsistency. No downgrade	All of the studies do display as a primary aim, very similar to the systematic review aim. Therefore, the available evidence is applicable to our research question. No downgrade	1. The overall sample size is large (n=3036), therefore, the optimal information size is met. 2. The confidence interval of the overall effect excludes the "favor control" values. The confidence interval represents the true underlying effect. No downgrade	Most studies in this meta-analysis do not suffer from important limitations, the evidence is direct and consistent. No major funding from the industry. No publication bias in the funnel plots. No downgrade	Given that the data are skewed, we converted SMD to Odds ratio (OR) using the equation LogOR= (π/√3)*SMD and we converted the LogOR into Risk Ratio (RR) using the equation RR= OR/ (1-Absolute Control Risk)*(1-OR). We assumed an absolute control risk reduction of 20% (ACR=0.2). The	No robust evidence for a dose response effect. No upgrade	We found no confounding factors that indicate upgrading

								outcome showed a RR<2. No upgrade		
Moderate ⊕⊕⊕◯	Effect of chronic aerobic exercise on influenza vaccine antibodies	Randomized controlled trials and controlled trials: Moderate quality	The included studies display >50% of low risk of bias. No downgrade	Even though we used a random effect model meta-analysis, we consider heterogeneity as an index of inconsistency. I2=76%, p<0.01, substantial heterogeneity. However, we controlled baseline differences of the measured antibodies of the participants by calculating Δ scores with post measurements data for antibodies. This approach removed the likelihood for inconsistency. No downgrade	All of the studies do display as a primary aim, very similar to the systematic review aim. Therefore, the available evidence is applicable to our research question. No downgrade	1. The overall sample size is large (n=2174), therefore, the optimal information size is met. 2. The confidence interval of the overall effect excludes the "favor control" values. The confidence interval represents the true underlying effect. No downgrade	Most studies in this meta-analysis do not suffer from important limitations, the evidence is direct and consistent. No major funding from the industry. No publication bias in the funnel plots. No downgrade	Given that the data are skewed, we converted SMD to Odds ratio (OR) using the equation LogOR= (π/√3)*SMD and we converted the LogOR into Risk Ratio (RR) using the equation RR= OR/ (1-Absolute Control Risk)*(1-OR). We assumed an absolute control risk reduction of 20% (ACR=0.2). The outcome showed a	No robust evidence for a dose response effect. No upgrade	We found no confounding factors that indicate upgrading

								RR<2. No upgrade		
Low ⊕⊕◯◯	Effect of physical activity levels on influenza vaccine antibodies	Cross-sectional studies: Low quality	The included studies display >50% of low risk of bias. No downgrade	Even though we used a random effect model meta-analysis, we consider heterogeneity as an index of inconsistency. I2=76%, $p<0.01$, substantial heterogeneity. However, we controlled baseline differences of the measured antibodies of the participants by calculating Δ scores with post measurements data for antibodies. This approach removed the likelihood for inconsistency. No downgrade	All of the studies do display as a primary aim, very similar to the systematic review aim. Therefore, the available evidence is applicable to our research question. No downgrade	1. The overall sample size is large (n=2847), therefore, the optimal information size is met. 2. The confidence interval of the overall effect excludes the "favor control" values. The confidence interval represents the true underlying effect. No downgrade	Most studies in this meta-analysis do not suffer from important limitations, the evidence is direct and consistent. No major funding from the industry. No publication bias in the funnel plots. No downgrade	Given that the data are skewed, we converted SMD to Odds ratio (OR) using the equation LogOR= $(\pi/\sqrt{3})$*SMD and we converted the LogOR into Risk Ratio (RR) using the equation RR= OR/ (1-Absolute Control Risk)*(1-OR). We assumed an absolute control risk reduction of 20% (ACR=0.2). The outcome showed a RR<2. No upgrade	No robust evidence for a dose response effect. No upgrade	We found no confounding factors that indicate upgrading

Low ⊕⊕◯◯	Effect of high physical activity levels on influenza vaccine antibodies	Cross-sectional studies: Low quality	The included studies display >50% of low risk of bias. No downgrade	Even though we used a random effect model meta-analysis, we consider heterogeneity as an index of inconsistency. $I2=78\%$, $p<0.01$, substantial heterogeneity. However, we controlled baseline differences of the measured antibodies of the participants by calculating Δ scores with post measurements data for antibodies. This approach removed the likelihood for inconsistency. No downgrade	All of the studies do display as a primary aim, very similar to the systematic review aim. Therefore, the available evidence is applicable to our research question. No downgrade	1. The overall sample size is large (n=1357), therefore, the optimal information size is met. 2. The confidence interval of the overall effect excludes the "favor inactive or low active" values. The confidence interval represents the true underlying effect. No downgrade	Most studies in this meta-analysis do not suffer from important limitations, the evidence is direct and consistent. No major funding from the industry. No publication bias in the funnel plots. No downgrade	Given that the data are skewed, we converted SMD to Odds ratio (OR) using the equation $LogOR=(\pi/\sqrt{3})*SMD$ and we converted the LogOR into Risk Ratio (RR) using the equation $RR= OR/(1-\text{Absolute Control Risk})*(1-OR)$. We assumed an absolute control risk reduction of 20% (ACR=0.2). The outcome showed a $RR<2$. No upgrade	No robust evidence for a dose response effect. No upgrade	We found no confounding factors that indicate upgrading

Supplementary Table S4. PRISMA checklist.

Section and Topic	Item #	Checklist item	Location where item is reported
**TITLE**			
Title	1	Identify the report as a systematic review.	Page 1
**ABSTRACT**			
Abstract	2	See the PRISMA 2020 for Abstracts checklist.	Page 1
**INTRODUCTION**			
Rationale	3	Describe the rationale for the review in the context of existing knowledge.	Pages 1-2
Objectives	4	Provide an explicit statement of the objective(s) or question(s) the review addresses.	Page 2
**METHODS**			
Eligibility criteria	5	Specify the inclusion and exclusion criteria for the review and how studies were grouped for the syntheses.	Page 2
Information sources	6	Specify all databases, registers, websites, organisations, reference lists and other sources searched or consulted to identify studies. Specify the date when each source was last searched or consulted.	Page 2
Search strategy	7	Present the full search strategies for all databases, registers and websites, including any filters and limits used.	Page 2, Supplement
Selection process	8	Specify the methods used to decide whether a study met the inclusion criteria of the review, including how many reviewers screened each record and each report retrieved, whether they worked independently, and if applicable, details of automation tools used in the process.	Page 2
Data collection process	9	Specify the methods used to collect data from reports, including how many reviewers collected data from each report, whether they worked independently, any processes for obtaining or confirming data from study investigators, and if applicable, details of automation tools used in the process.	Pages 3-4
Data items	10a	List and define all outcomes for which data were sought. Specify whether all results that were compatible with each outcome domain in each study were sought (e.g. for all measures, time points, analyses), and if not, the methods used to decide which results to collect.	Pages 3-4

Section and Topic	Item #	Checklist item	Location where item is reported
	10b	List and define all other variables for which data were sought (e.g. participant and intervention characteristics, funding sources). Describe any assumptions made about any missing or unclear information.	Pages 3-4
Study risk of bias assessment	11	Specify the methods used to assess risk of bias in the included studies, including details of the tool(s) used, how many reviewers assessed each study and whether they worked independently, and if applicable, details of automation tools used in the process.	Page 3
Effect measures	12	Specify for each outcome the effect measure(s) (e.g. risk ratio, mean difference) used in the synthesis or presentation of results.	Pages 3-4
Synthesis methods	13a	Describe the processes used to decide which studies were eligible for each synthesis (e.g. tabulating the study intervention characteristics and comparing against the planned groups for each synthesis (item #5)).	Page 2
	13b	Describe any methods required to prepare the data for presentation or synthesis, such as handling of missing summary statistics, or data conversions.	Pages 3-4
	13c	Describe any methods used to tabulate or visually display results of individual studies and syntheses.	Pages 3-4
	13d	Describe any methods used to synthesize results and provide a rationale for the choice(s). If meta-analysis was performed, describe the model(s), method(s) to identify the presence and extent of statistical heterogeneity, and software package(s) used.	Pages 3-4
	13e	Describe any methods used to explore possible causes of heterogeneity among study results (e.g. subgroup analysis, meta-regression).	Page 4
	13f	Describe any sensitivity analyses conducted to assess robustness of the synthesized results.	NA
Reporting bias assessment	14	Describe any methods used to assess risk of bias due to missing results in a synthesis (arising from reporting biases).	NA
Certainty assessment	15	Describe any methods used to assess certainty (or confidence) in the body of evidence for an outcome.	Page 4
**RESULTS**			
Study selection	16a	Describe the results of the search and selection process, from the number of records identified in the search to the number of studies included in the review, ideally using a flow diagram.	Page 5
	16b	Cite studies that might appear to meet the inclusion criteria, but which were excluded, and explain why they were excluded.	Supplement
Study characteristics	17	Cite each included study and present its characteristics.	Page 5, Supplement

Section and Topic	Item #	Checklist item	Location where item is reported
Risk of bias in studies	18	Present assessments of risk of bias for each included study.	Page 5, Supplement
Results of individual studies	19	For all outcomes, present, for each study: (a) summary statistics for each group (where appropriate) and (b) an effect estimate and its precision (e.g. confidence/credible interval), ideally using structured tables or plots.	Pages 5-12, Supplement
Results of syntheses	20a	For each synthesis, briefly summarise the characteristics and risk of bias among contributing studies.	Pages 5-12, Supplement
	20b	Present results of all statistical syntheses conducted. If meta-analysis was done, present for each the summary estimate and its precision (e.g. confidence/credible interval) and measures of statistical heterogeneity. If comparing groups, describe the direction of the effect.	Pages 5-12, Supplement
	20c	Present results of all investigations of possible causes of heterogeneity among study results.	NA
	20d	Present results of all sensitivity analyses conducted to assess the robustness of the synthesized results.	NA
Reporting biases	21	Present assessments of risk of bias due to missing results (arising from reporting biases) for each synthesis assessed.	NA
Certainty of evidence	22	Present assessments of certainty (or confidence) in the body of evidence for each outcome assessed.	Page 12, supplement
**DISCUSSION**			
Discussion	23a	Provide a general interpretation of the results in the context of other evidence.	Pages 13-14
	23b	Discuss any limitations of the evidence included in the review.	Page 14
	23c	Discuss any limitations of the review processes used.	Page 14
	23d	Discuss implications of the results for practice, policy, and future research.	Page 15
**OTHER INFORMATION**			
Registration and protocol	24a	Provide registration information for the review, including register name and registration number, or state that the review was not registered.	Page 2
	24b	Indicate where the review protocol can be accessed, or state that a protocol was not prepared.	Page 2

Section and Topic	Item #	Checklist item	Location where item is reported
	24c	Describe and explain any amendments to information provided at registration or in the protocol.	Pages 14-15
Support	25	Describe sources of financial or non-financial support for the review, and the role of the funders or sponsors in the review.	Page 15
Competing interests	26	Declare any competing interests of review authors.	Page 15
Availability of data, code and other materials	27	Report which of the following are publicly available and where they can be found: template data collection forms; data extracted from included studies; data used for all analyses; analytic code; any other materials used in the review.	Pages 5 and 15 , Supplement

List of Excluded Publications

<u>Of the total 2589 retrieved publications 845 duplicates and 35 were eligible. Therefore the full list includes 1719 publications, which were excluded.</u>

1. Guidelines for the assessment and management of chronic obstructive pulmonary disease. Canadian Thoracic Society Workshop Group. Cmaj 1992; 147(4): 420-8.
2. Adult pneumococcal vaccination guideline. SAMA-SA Pulmonology Society Working Group. S Afr Med J 1999; 89(11 Suppl): 1222-30.
3. Taste for Victory. Sports Illustrated for Women 2001; 3(4): 28.
4. Some factors affecting prevalence of and immune responses to Schistosoma mansoni in schoolchildren in Gorgora, northwest Ethiopia. Ethiopian medical journal 2002; 40(4): 345-52.
5. HEALTH WATCH. Yoga Journal 2002; (171): 36.
6. New developments in the treatment of scleroderma. Drugs and therapy perspectives 2002; 18(2): 19-21.
7. Omalizumab: anti-IgE monoclonal antibody E25, E25, humanised anti-IgE MAb, IGE 025, monoclonal antibody E25, Olizumab, Xolair, rhuMAb-E25. BioDrugs 2002; 16(5): 380-6.
8. ABSTRACTS. Journal of Orthopaedic & Sports Physical Therapy 2003; 33(11): 694-701.
9. Tai chi boosts immune system function. Research Review (International Council on Active Aging) 2007; 7(11): 2-3.
10. STRESSED STRENGTH: How stress can reduce the effectiveness of your training. Journal of Pure Power 2009; 4(1): 23-5.
11. Continuous Subcutaneous Insulin Infusion (CSII) Pumps for Type 1 and Type 2 Adult Diabetic Populations: An Evidence-Based Analysis. Ont Health Technol Assess Ser 2009; 9(20): 1-58.
12. Mexican flu: risk perception in the general public, precautionary measures and trust in information provided by the government. Nederlands tijdschrift voor geneeskunde 2010; 154: A1686.
13. A gut feeling. Ultra-Fit Magazine 2011; 21(3): 60-1.
14. MY DAY ON A PLATE. Triathlete 2011; (325): 138-.
15. POSTER PRESENTATIONS. Pacing & Clinical Electrophysiology 2011; 34(11): 1362-451.
16. SHORT CUTS. BMJ: British Medical Journal (Overseas & Retired Doctors Edition) 2011; 342(7795): 466-7.
17. Continuous glucose monitoring for patients with diabetes: an evidence-based analysis. Ont Health Technol Assess Ser 2011; 11(4): 1-29.
18. T Cell Vaccination Benefits Relapsing Progressive Multiple Sclerosis Patients: A Randomized, Double-Blind Clinical Trial. PLoS ONE 2012; 7(12): e50478.
19. Vulnerability, distress, and immune response to vaccination in older adults. Brain, Behavior, and Immunity 2012; 26(5): 747-53.
20. Health and economic impact of PHiD-CV in Canada and the UK: A Markov modelling exercise. Journal of Medical Economics 2012; 15(1): 61-76.
21. Noninvasive positive pressure ventilation for chronic respiratory failure patients with stable chronic obstructive pulmonary disease (COPD): an evidence-based analysis. Ont Health Technol Assess Ser 2012; 12(9): 1-51.
22. Long-term oxygen therapy for patients with chronic obstructive pulmonary disease (COPD): an evidence-based analysis. Ont Health Technol Assess Ser 2012; 12(7): 1-64.
23. Pulmonary rehabilitation for patients with chronic pulmonary disease (COPD): an evidence-based analysis. Ont Health Technol Assess Ser 2012; 12(6): 1-75.
24. Induction and regulation of T-cell immunity by the novel tuberculosis vaccine M72/AS01 in South African adults. American Journal of Respiratory and Critical Care Medicine 2013; 188(4): 492-502.
25. Leptin predicts a decline in moderate to vigorous physical activity in minority female children at risk for obesity. Pediatric Obesity 2013; 8(1): 70-7.

26. Progress in Pediatrics in 2012: Choices in allergy, endocrinology, gastroenterology, hematology, infectious diseases, neurology, nutrition and respiratory tract illnesses. Italian Journal of Pediatrics 2013; 39(1): 26.
27. Don't Miss Free Online Content from Medicine & Science in Sports & Exercise®. Sports Medicine Bulletin 2014: 7-.
28. Flu shot linked to lower risk of heart problems, say Harvard researchers. Harvard Health Letter 2014; 39(3): 8-.
29. IN THE JOURNALS. Harvard Men's Health Watch 2015; 19(12): 8-.
30. THE GREAT VACCINE DEBATE. Horse Sport 2016; 49(7): 58-.
31. Phase II multicenter, randomized, double-blind controlled study of efficacy and safety of umbilical cord-derived mesenchymal stromal cells in the prophylaxis of chronic graft-versus-host disease after HLA-haploidentical stem-cell transplantation. Journal of clinical oncology 34 (24) (pp 2843-2850), 2016 Date of publication: 20 aug 2016 2016.
32. Exercise strategies to protect against the impact of short-term reduced physical activity on muscle function and markers of health in older men: study protocol for a randomised controlled trial. Trials 17 (1) (no pagination), 2016 Article number: 381 Date of publication: 02 aug 2016 2016.
33. Investigation of food allergy especially wheat dependent exercise induced anaphylaxis (WDEIA) in population of Sindh, Pakistan. Rawal Medical Journal 2017; 42(1): 98-101.
34. The Observatory. New Studies. MLO: Medical Laboratory Observer 2017; 49(2): 6-.
35. Nutritional intervention based on mediterranean diet reduces blood pressure and immune cells response after immune stimulation. Annals of nutrition & metabolism 2017; Conference: 10th Annual Conference of the International Symposium on Immunonutrition. Spain. 71(1-2): 70-1.
36. Efficacy of dietary supplement contained proteoglycan extracted from salmon nasal cartilage on knee uncomfortableness in healthy volunteers -A randomized, double-blind placebo-controlled intergroup trial. Japanese pharmacology and therapeutics 2017; 45(11): 1795-808.
37. A self-affirmation exercise does not improve intentions to vaccinate among parents with negative vaccine attitudes (and may decrease intentions to vaccinate). Plos one 2017; 12(7).
38. Effectiveness of specialized and intensive adl training in patients with idiopathic inflammatory myopathies - preliminary results of a one-year controlled study. Annals of the rheumatic diseases 2019; 78: 1804-.
39. Mindfulness and cardiovascular health: outcomes, mechanisms & individual differences. Psychosomatic medicine 2019; 81(4): A166-A7.
40. Water immersion methods do not alter muscle damage and inflammation biomarkers after high-intensity sprinting and jumping exercise. European journal of applied physiology 2020.
41. High-intensity interval training in allogeneic adoptive T-cell immunotherapy - A big HIT? Journal of translational medicine 2020; 18(1).
42. The effect of propolis supplementation on inflammatory factors and oxidative status in women with rheumatoid arthritis: design and research protocol of a double-blind, randomized controlled. Contemporary clinical trials communications 2021; 23.
43. Variance of concern. Diabetic Medicine 2022; 39(2): 1-2.
44. Abaraogu U, Dall PM, Duncan E, et al. Effects of Regular Physical Activity on the Immune System, Vaccination and Risk of Community-Acquired Infectious Disease in the General Population: Systematic Review and Meta-Analysis. Sports medicine (Auckland, NZ) 2021; 51(8): 1673-86.
45. Abdi H, Ghaffarian-Zirak R, Barati E, Rohban M, Ghazizadeh H, Ghayour-Mobarhan M. Effect of body and ear acupuncture on obesity. Obesity Medicine 2020; 19: 100257.
46. Abed Elhadi Shahbari N, Gesser-Edelsburg A, Mesch GS. Case of Paradoxical Cultural Sensitivity: Mixed Method Study of Web-Based Health Informational Materials About the Human Papillomavirus Vaccine in Israel. Journal of medical Internet research 2019; 21(5): e13373.

47. Abidin NZ, Yusof A, George A, Udani J. Efficacy and safety of Eurycoma longifolia (Physta®) water extract plus multivitamins on quality of life, mood and stress: a randomized placebo-controlled and parallel study. Food & Nutrition Research 2018; 62: 1-16.
48. Actrn. Kunzea oil for the management of mild to moderate psoriasis (a common, chronic, relapsing, immune-mediated skin disease: a pilot randomised controlled trial. https://trialsearchwhoint/Trial2aspx?TrialID=ACTRN12614001014640 2014.
49. Actrn. The effectiveness of a home based strengthening exercise programme combined with oral nutrition in improving muscle mass, strength and function in people aged 65 years and over. https://trialsearchwhoint/Trial2aspx?TrialID=ACTRN12618001916235 2018.
50. Actrn. Physical activity adherence, psychological health and immunological outcomes (PAPHIO study) in breast cancer survivors. https://trialsearchwhoint/Trial2aspx?TrialID=ACTRN12619001271190 2019.
51. Actrn. Preoperative exercise therapy for patients with cancer: a randomised-controlled trial. https://trialsearchwhoint/Trial2aspx?TrialID=ACTRN12619000214134 2019.
52. Actrn. Sirolimus in Inclusion Body Myositis (IBM). https://trialsearchwhoint/Trial2aspx?TrialID=ACTRN12620001226998 2020.
53. Actrn. Effectiveness of exercise in Systemic Lupus Erythematosus. https://trialsearchwhoint/Trial2aspx?TrialID=ACTRN12621000970842 2021.
54. Actrn. A pilot randomised controlled trial: joint Walkers, a Group of Rheumatology Outdoor Walkers (GROW Study). https://trialsearchwhoint/Trial2aspx?TrialID=ACTRN12621000945820 2021.
55. Adams H, Horrevoets WM, Adema SM, et al. Reprint of "Inhibition of biofilm formation by Camelid single-domainantibodies against the flagellum of Pseudomonas aeruginosa". J Biotechnol 2014; 191: 131-8.
56. Adams J, Giles EL, McColl E, Sniehotta FF. Carrots, sticks and health behaviours: a framework for documenting the complexity of financial incentive interventions to change health behaviours. Health Psychol Rev 2014; 8(3): 286-95.
57. Agar AM, Roizen G, Fajre X, et al. Five years follow up of a patient with chronic urticaria on omalizumab treatment. World Allergy Organization Journal 2020; 13(8): 100318.
58. Aghili SMM, Ebrahimpur M, Arjmand B, et al. Obesity in COVID-19 era, implications for mechanisms, comorbidities, and prognosis: a review and meta-analysis. Int J Obes (Lond) 2021; 45(5): 998-1016.
59. Agmon-Levin N, Arango MT, Kivity S, et al. Immunization with hepatitis b vaccine accelerates SLE-like disease in an animal model. Arthritis and Rheumatism 2013; 65(SUPPL. 10): S247.
60. Ahirwar AK, Singh S, Asia P, Gopal N, Kaim K, Ahirwar P. COVID-19 and neurology perspective. Hormone Molecular Biology and Clinical Investigation 2021; 42(1): 69-75.
61. Ahn J-O, Chung J-Y, Kim DH, Im W, Kim SH. Differences of RNA Expression in the Tendon According to Anatomic Outcomes in Rotator Cuff Repair. American Journal of Sports Medicine 2018; 46(13): 2995-3003.
62. Ahokas EK, Kyrolainen H, Mero AA, Walker S, Hanstock HG, Ihalainen JK. Water immersion methods do not alter muscle damage and inflammation biomarkers after high-intensity sprinting and jumping exercise. European journal of applied physiology 2020; 120(12): 2625-34.
63. Aiello AE, Haan MN, Pierce CM, Simanek AM, Liang J. Persistent Infection, Inflammation, and Functional Impairment in Older Latinos. Journals of Gerontology Series A: Biological Sciences & Medical Sciences 2008; 63(6): 610-8.
64. Al-Aidaroos AYA, Standaert B, Meszaros K, Shibl AM. Economic assessment of rotavirus vaccination in Saudi Arabia. Journal of Infection and Public Health 2017; 10(5): 564-71.
65. Al-Magsoosi HHE, Al-Bayati HSJ, Al-Timmemi HAK. IMMUNO-HEMATOLOGICAL RESPONSE TO RADIAL NERVE INJURY AND HUMAN UMBILICAL CORD-MESENCHYMAL STEM CELLS (HUC-MSCS) THERAPY IN DOGS, IRAQ. Biochemical and Cellular Archives 2020; 20(2): 6447-56.

66. Al-Musa HM. Early onset type 2 diabetes mellitus in a Saudi child misdiagnosed as type 1 diabetic: A case report. J Family Med Prim Care 2019; 8(1): 313-5.
67. Alavi S, Kord Valeshabad A, Moradveisi B, Aminasnafi A, Arzanian MT. Clinical responses to rituximab in a case of neuroblastoma with refractory opsoclonus myoclonus ataxia syndrome. Case Rep Oncol Med 2012; 2012: 164082.
68. Albrecht E, Norheim F, Thiede B, et al. Irisin - a myth rather than an exercise-inducible myokine. Scientific reports 2015; 5: 8889.
69. Aldekwer S, Desiderio A, Farges M-C, et al. Vitamin D supplementation associated with physical exercise promotes a tolerogenic immune environment without effect on mammary tumour growth in C57BL/6 mice. European Journal of Nutrition 2021; 60(5): 2521-35.
70. Alekseyev K, Udani J, Patel J, Ross M. Autoimmune induced necrotizing myopathy. PM and R 2016; 8(9 Supplement): S209.
71. Alexandrow V, Alexandrow A, Alexandrowa N, Shilova L, Nikitin M. Peculiarites of correction of antioxidant status in patients with osteoarthrosis in the rehabilitation process. Osteoporosis International 2018; 29(1 Supplement 1): S400.
72. Allison DJ, Chapma B, Wolfe D, Sequeira K, Hayes K, Ditor DS. Effects of a Functional Electrical Stimulation--Assisted Cycling Program on Immune and Cardiovascular Health in Persons with Spinal Cord Injury. Topics in Spinal Cord Injury Rehabilitation 2016; 22(1): 71-8.
73. Allison DJ, Ditor DS. The common inflammatory etiology of depression and cognitive impairment: A therapeutic target. Journal of Neuroinflammation 2014; 11(1): 151.
74. Alrumayyan N, McAlpine S, Issekutz T, et al. Prolidase deficiency: Description of its presentation and detailed immunological assessment. LymphoSign Journal 2020; 7(4): 129-33.
75. Alshammari E, Shafi S, Nurmi-Lawton J, Burut DF, Lanham-New S, Ferns G. Markers of inflammation, endothelial activation and autoimmunity in adolescent female gymnasts. J Sports Sci Med 2010; 9(4): 538-46.
76. Alshawi HNH, Abdulsada ZA. The relationship of mental fatigue (Flim) with the level of hormone cortisone and the performance of running (100) meters for young players. Journal of Global Pharma Technology 2017; 9(9PartB): 196-200.
77. AlTahan AM, Berger T, AlOrainy IA, AlTahan H. Progressive multifocal leukoencephalopathy in the absence of typical radiological changes: Can we make a diagnosis? American Journal of Case Reports 2019; 20: 101-5.
78. Alzaman N, Siegel RD, Schaefer EJ. Prolonged honeymoon period in type 1 diabetic patient with low carbohydrate intake. Endocrine Reviews 2014; 35(SUPPL. 3).
79. Amarnani A, Rosenthal KS, Mercado JM, Brodell RT. Concurrent treatment of chronic psoriasis and asthma with ustekinumab. Journal of Dermatological Treatment 2014; 25(1): 63-6.
80. Amati R, Taglioli M, Comeglio M, Italiani G, Rossini D. Considerations on a case of eosynophilic granulomatosis with heart involvement. European Heart Journal, Supplement 2020; 22(SUPPL G): G168-G9.
81. Amato AA. Ongoing developments in IBM. Journal of Neuromuscular Diseases 2016; 3(Supplement 1): S47.
82. Ambler W, Hammond M, Taylor R, Sansing L, Ai Y. Alpha-4 integrin mediates inflammatory monocyte trafficking to the brain after intracerebral hemorrhage. Stroke 2014; 45(SUPPL. 1).
83. Amico AP, Terlizzi A, Damiani S, Ranieri M, Megna M, Fiore P. Immunopharmacology of the main herbal supplements: A review. Endocrine, Metabolic and Immune Disorders - Drug Targets 2013; 13(4): 283-8.
84. Amruthesh S. Role of ayurveda and yoga in prevention of cancer and management of cancer. Journal of Carcinogenesis 2011; 10: S2.
85. Anand P, Guillaumet-Adkins A, Dimitrova V, et al. Single-cell RNA-seq reveals developmental plasticity with coexisting oncogenic states and immune evasion programs in ETP-ALL. Blood 2021; 137(18): 2463-80.

86. Anderson C, Riehle C, Khan F, Guidon A, Kolb N. Lambert eaton myasthenic syndrome in a patient with small cell lung cancer on immune checkpoint inhibitor therapy. Muscle and Nerve 2020; 62(SUPPL 1): S47.
87. Andia I, Maffulli N. Mesenchymal stromal cell products for intra-articular knee injections for conservative management of osteoarthritis. Ther Adv Musculoskelet Dis 2021; 13: 1759720x21996953.
88. Andrade C, Radhakrishnan R. The prevention and treatment of cognitive decline and dementia: An overview of recent research on experimental treatments. Indian Journal of Psychiatry 2009; 51(1): 12-25.
89. Andrawes WF, Bussy C, Belmin J. Prevention of cardiovascular events in elderly people. Drugs and Aging 2005; 22(10): 859-76.
90. Anisuzzaman, Frahm S, Prodjinotho UF, Bhattacharjee S, Verschoor A, Prazeres da Costa C. Host-Specific Serum Factors Control the Development and Survival of Schistosoma mansoni. Front Immunol 2021; 12: 635622.
91. Anonymous. Omalizumab: anti-IgE monoclonal antibody E25, E25, humanised anti-IgE MAb, IGE 025, monoclonal antibody E25, Olizumab, Xolair, rhuMAb-E25. BioDrugs : clinical immunotherapeutics, biopharmaceuticals and gene therapy 2002; 16(5): 380-6.
92. Anonymous. Food allergy: A practice parameter. Annals of Allergy, Asthma and Immunology 2006; 96(3 SUPPL. 2): S1-S68.
93. Anonymous. Canadian Society of Allergy and Clinical Immunology Annual Scientific Meeting 2010. Allergy, Asthma and Clinical Immunology 2010; 6(SUPPL. 2).
94. Anonymous. Hot Topic Abstracts from ICO 2010 11th International Congress on Obesity. Obesity Reviews 2010; 11(11).
95. Anonymous. Australian Rheumatology Association in Conjunction with Rheumatology Health Professionals Association 52nd Annual Scientific Meeting. Internal Medicine Journal 2011; 41(SUPPL. 1).
96. Anonymous. 25th National Biochemistry Congress. Turkish Journal of Biochemistry 2013; 38(spec. issue 1).
97. Anonymous. 2nd National Congress on Medicinal Plants. Iranian Journal of Pharmaceutical Research 2013; 12(Supplement 2): 43.
98. Anonymous. ACTRIMS Forum 2016. Multiple Sclerosis 2016; 22(1 SUPPL. 1).
99. Anonymous. Oman Medical Specialty Board Career and Research Forum 2018: Abstracts. Oman Medical Journal 2018; 33(1).
100. Anonymous. 5th Pediatric Allergy and Asthma Meeting, PAAM. Clinical and Translational Allergy 2018; 8(Supplement 2).
101. Anonymous. Society for Vascular Medicine Abstracts. Vascular Medicine 2018; 23(3).
102. Anonymous. Journal of Preventive Medicine and Hygiene 2019; 60(3 Supplement 1).
103. Anonymous. Abstracts of the 27th European Workshop on Neonatology. Journal of Neonatal-Perinatal Medicine 2019; 12(3).
104. Anonymous. ISCT 2020 Paris Virtual. Cytotherapy 2020; 22(5 Supplement): S1-S206.
105. Anonymous. The Changing Practice Landscape. Annals of Allergy, Asthma and Immunology 2020; 125(5 Supplement): S1-S136.
106. Anonymous. ITOC7 - 7th Leading International Cancer Immunotherapy Conference in Europe. Journal for ImmunoTherapy of Cancer 2020; 8(SUPPL 2).
107. Anonymous. Abstracts of the 20th Biennial European Society for Organ Transplantation (ESOT) Congress. Transplant International 2021; 34(SUPPL 1).
108. Anthony SA, Thurtell MJ, Leigh RJ. Miller Fisher syndrome mimicking ocular myasthenia gravis. Optometry and vision science : official publication of the American Academy of Optometry 2012; 89(12): e118-23.

109. Aoki K, Shikama YS, Kokado A, Yoshida T, Kuroiwa Y. Enzyme-linked immunosorbent assay and latex agglutination inhibition reaction test for morphine in urine. Forensic Sci Int 1996; 81(2-3): 125-32.
110. Aouina H, Bamri A, Vesin A, et al. Oscillococcinum® for upper respiratory tract infections and exacerbations in COPD: an observational, prospective study (OXITUNIS). Drugs in context 2021; 10.
111. Apte SM, Vadhan-Raj S, Cohen L, et al. Cytokines, GM-CSF and IFNgamma administered by priming and post-chemotherapy cycling in recurrent ovarian cancer patients receiving carboplatin. J Transl Med 2006; 4: 16.
112. Arango MT, Blank M, Tomljenovic L, Shoenfeld Y. HPV vaccination of NZBXW/F1 mice. Arthritis and Rheumatology 2015; 67(SUPPL. 10).
113. Arcangeli ML, Bardin F, Chabannon C, Aurrand-Lions M, Adams R. Role of JAM-B/JAM-C interaction in the circulation of hematopoietic stem cells and their retention in the bone marrow. Haematologica 2012; 97(SUPPL. 1): 476.
114. Arena R, Bond S, Calvo IR, et al. Shelter from the cytokine storm: Healthy living is a vital preventative strategy in the COVID-19 era. Prog Cardiovasc Dis 2021.
115. Arleevskaya M, Takha E, Petrov S, et al. Causal risk and protective factors in rheumatoid arthritis: A genetic update. J Transl Autoimmun 2021; 4: 100119.
116. Arnold DM, Clare R, Salib M, et al. The McMaster ITP registry: Assessing the prevalence, clinical and laboratory features of immune thrombocytopenia. Blood 2014; 124(21).
117. Arnold DM, Nazi I, Toltl LJ, et al. Antibody binding to megakaryocytes in vivo in patients with immune thrombocytopenia. Eur J Haematol 2015; 95(6): 532-7.
118. Arroll B, Young D, Flicker L, Jolley D, Kerse NM. Improving the health behaviours of elderly people: Randomised controlled trial of a general practice education programme. British Medical Journal 1999; 319(7211): 683-7.
119. Arshad S, Angeles J, Arabelo HA. Poems syndrome: A rare cause of gait imbalance. Journal of the American Geriatrics Society 2012; 60(SUPPL. 4): S78.
120. Arya SC. Human immunization in developing countries: Practical and theoretical problems and prospects. Vaccine 1994; 12(15): 1423-35.
121. Asamoah JKK, Okyere E, Abidemi A, et al. Optimal control and comprehensive cost-effectiveness analysis for COVID-19. Results Phys 2022; 33: 105177.
122. Ashcraft KA, Dewhirst MW, Warner AB, Jones LW. Exercise as Adjunct Therapy in Cancer. Seminars in Radiation Oncology 2019; 29(1): 16-24.
123. Ashigbie PG, Aziz N, Manjunatha UH, et al. Use-case scenarios for an anti-cryptosporidium therapeutic. PLoS Neglected Tropical Diseases 2021; 15(3): e0009057.
124. Ashraf S, Alsharedi M. COVID-19 induced immune thrombocytopenic purpura: case report. Stem Cell Investig 2021; 8: 14.
125. Ataca Atilla P, McKenna MK, Watanabe N, Atilla E, Mamonkin M, Brenner MK. Combinatorial Antigen Targeting Strategy for Acute Myeloid Leukemia. Blood 2020; 136(Supplement 1): 22-3.
126. Atkins D, DiGuiseppi CG. Broadening the evidence base for evidence-based guidelines: A research agenda based on the work of the U.S. preventive services task force. American Journal of Preventive Medicine 1998; 14(4): 335-44.
127. Aujla S, Chaudhry S, Mahmoud M. A rare case of EBV cholestatic hepatitis. Critical Care Medicine 2021; 49(1 SUPPL 1): 283.
128. Avorn J, Gurwitz JH. Drug use in the nursing home. Annals of Internal Medicine 1995; 123(3): 195-204.
129. Awasthi S, Singhal SS. Targeting melanoma through RALBP1/RLIP76. Pigment Cell and Melanoma Research 2012; 25(6): 843.

130. Bach Jr BR, Aadalen KJ, Dennis MG, et al. Primary Anterior Cruciate Ligament Reconstruction Using Fresh-Frozen, Nonirradiated Patellar Tendon Allograft: Minimum 2-Year Follow-up. American Journal of Sports Medicine 2005; 33(2): 284-92.
131. Badawi N-R, Erhart H, Kepplinger B, Baran H, Sedlnitzky-Semler B. Stochastic resonance therapy (SRT) and tryptophan metabolism. Amino Acids 2011; 41(SUPPL. 1): S33-S4.
132. Badawy SM, Kuhns LM. Texting and Mobile Phone App Interventions for Improving Adherence to Preventive Behavior in Adolescents: A Systematic Review. JMIR Mhealth Uhealth 2017; 5(4): e50.
133. Badr CE, Silver DJ, Siebzehnrubl FA, Deleyrolle LP. Metabolic heterogeneity and adaptability in brain tumors. Cell Mol Life Sci 2020; 77(24): 5101-19.
134. Bahrke MS, Morgan WP. Evaluation of the Ergogenic Properties of Ginseng: An Update. Sports Medicine 2000; 29(2): 113-33.
135. Baigis J, Korniewicz DM, Chase G, Butz A, Jacobson D, Wu AW. Effectiveness of a home-based exercise intervention for HIV-infected adults: a randomized trial. The Journal of the Association of Nurses in AIDS Care : JANAC 2002; 13(2): 33-45.
136. Baker F, Bigley AB, Simpson RJ, Hussain M, Peek MK, Stowe RP. Cardiorespiratory fitness is associated with better control of latent herpesvirus infections in a large ethnically diverse community sample: Evidence from the Texas City Stress and Health Study. Brain, Behavior, and Immunity 2017; 66(Supplement 1): e35.
137. Bala A, Kumar R, Harjai K. Inhibition of quorum sensing in Pseudomonas aeruginosa by azithromycin and its effectiveness in urinary tract infections. J Med Microbiol 2011; 60(Pt 3): 300-6.
138. Ball TM. Structural integration-based fascial release efficacy in systemic lupus erythematosus (SLE): Two case studies. Journal of Bodywork & Movement Therapies 2011; 15(2): 217-25.
139. Bamgbola OF. Urinary schistosomiasis. Pediatric Nephrology 2014; 29(11): 2113-20.
140. Bang P, Brandt J, Degerblad M, et al. Exercise-induced changes in insulin-like growth factors and their low molecular weight binding protein in healthy subjects and patients with growth hormone deficiency. European Journal of Clinical Investigation 1990; 20(3): 285-92.
141. Baranova DE, Chen L, Destrempes M, Meade H, Mantis NJ. Passive Immunity to Vibrio cholerae O1 Afforded by a Human Monoclonal IgA1 Antibody Expressed in Milk. Pathog Immun 2020; 5(1): 89-116.
142. Barbas CF, 3rd, Burton DR. Selection and evolution of high-affinity human anti-viral antibodies. Trends Biotechnol 1996; 14(7): 230-4.
143. Barbieri EA, Bawle EB, Toder DS, et al. A syndrome of hypoplastic sinuses, hydrocephalus, bronchiectasis, and hypogammaglobulinemia with functional antibody deficiency in twin girls. Annals of Allergy, Asthma and Immunology 2005; 94(6): 693-9.
144. Bardet A, Ibrahimi N, Imbert A, et al. Delivering adapted physical activity by videoconference to patients with fatigue under immune checkpoint inhibitors: Lessons learned from the PACTIMe-FEAS feasibility study. Journal of telemedicine and telecare 2021: 1357633X211021743.
145. Barker W, Duara R, Loewenstein D, Bain L. The basis for disease-modifying treatments for Alzheimer's disease: The Sixth Annual Mild Cognitive Impairment Symposium. Alzheimer's and Dementia 2009; 5(1): 66-74.
146. Barrett HL, Callaway LK, Nitert MD. Probiotics: a potential role in the prevention of gestational diabetes? Acta diabetologica 2012: 1-13.
147. Bartley JM, Stearns RL, Munoz C, et al. Effects of cold water immersion on circulating inflammatory markers at the Kona Ironman World Championship. Physiologie appliquee, nutrition et metabolisme [Applied physiology, nutrition, and metabolism] 2021.
148. Barug D, Berbers GAM, Kuijer M, et al. Infant antibody levels following 10-valent pneumococcal-protein D conjugate and DTaP-Hib vaccinations in the first year of life after maternal Tdap vaccination: An open-label, parallel, randomised controlled trial. Vaccine 2020; 38(29): 4632-9.

149. Barug D, Pronk I, Knol MJ, et al. Maternal pertussis vaccination and its effects on the immune response of infants aged up to 12 months in the Netherlands: an open-label, parallel, randomised controlled trial. The Lancet Infectious Diseases 2019; 19(4): 392-401.
150. Bassaganya-Riera J, Pogranichniy RM, Jobgen SC, et al. Conjugated linoleic acid ameliorates viral infectivity in a pig model of virally induced immunosuppression. Journal of Nutrition 2003; 133(10): 3204-14.
151. Bastani R, Glenn BA, Herrmann AK, et al. Community-based intervention to reduce liver cancer disparities in Asian Americans: A cluster randomized trial. Cancer Prevention Research 2010; 3(12 SUPPL. 2).
152. Basu N, Paudyal P, Macfarlane MV, et al. Multi-disciplinary management of fatigue in anca-associated vasculitis: A pilot study. Rheumatology (United Kingdom) 2012; 51(SUPPL. 3).
153. Bateman RM, Sharpe MD, Jagger JE, et al. 36th International Symposium on Intensive Care and Emergency Medicine : Brussels, Belgium. 15-18 March 2016. Crit Care 2016; 20(Suppl 2): 94.
154. Bauer ME, Trujillo D, Brown C, et al. What Do We Know? Teaching Medical Students to Deal with Uncertainty as a Pandemic Unfolds. Open Forum Infectious Diseases 2020; 7(SUPPL 1): S597.
155. Baum A, Herberman H, Cohen L. Managing stress and managing illness: Survival and quality of life in chronic disease. J Clin Psychol Med Settings 1995; 2(4): 309-33.
156. Baxter S, Sanderson K, Venn AJ, Blizzard CL, Palmer AJ. The relationship between return on investment and quality of study methodology in workplace health promotion programs. Am J Health Promot 2014; 28(6): 347-63.
157. Beaston-Blaakman A, Shepard DS, Stone N, Shevitz AH. Cost-effectiveness of clinical interventions for AIDS wasting. AIDS care - psychological and socio-medical aspects of AIDS/HIV 2007; 19(8): 996-1001.
158. Beau F, Berlioz-Arthaud A, Melix G, et al. Human leptospirosis in French polynesia. Epidemiological, clinical and bacteriological features. Medecine Tropicale 2007; 67(2): 137-44.
159. Beaulieu M-C, Robindaine J, Tremblay H, Dubuc SD, Liang P, Gervais F. Living and adjusting to a diagnosis of rheumatoid arthritis (RA) with an interdisciplinary team. Arthritis and Rheumatology 2019; 71(Supplement 10): 5279.
160. Bechini A, Paolini D, Pieralli F, et al. Do Tuscan people adhere to meningococcal C vaccination during an emergency campaign? J Prev Med Hyg 2018; 59(3): E187-e93.
161. Beck KL, von Hurst PR, O'Brien WJ, Badenhorst CE. Micronutrients and athletic performance: A review. Food and Chemical Toxicology 2021; 158: 112618.
162. Bedford FL. Perceptual mindfulness and imagery for chronic pain and skin disorders in a busy college population. Journal of Pain Management 2015; 8(1): 55-63.
163. Belalcazar V, Segura J, Pascual JA, et al. Anti-EPO and anti-NESP antibodies raised against synthetic peptides that reproduce the minimal amino acid sequence differences between EPO and NESP. Analytical and Bioanalytical Chemistry 2007; 388(7): 1531-8.
164. Belcher BR, Chou CP, Nguyen-Rodriguez ST, et al. Leptin predicts a decline in moderate to vigorous physical activity in minority female children at risk for obesity. Pediatr Obes 2013; 8(1): 70-7.
165. Belizário JE, Fontes-Oliveira CC, Borges JP, Kashiabara JA, Vannier E. Skeletal muscle wasting and renewal: a pivotal role of myokine IL-6. Springerplus 2016; 5: 619.
166. Bellinger DL, Lorton D. Strategies for reversing age-related sympathetic neuropathy loss in immune organs. Advances in Neuroimmune Biology 2013; 4(2): 97-123.
167. Belvederi Murri M, Folesani F, Zerbinati L, et al. Physical Activity Promotes Health and Reduces Cardiovascular Mortality in Depressed Populations: A Literature Overview. Int J Environ Res Public Health 2020; 17(15).
168. Benoit A, Beran J, Devaster JM, et al. Hemagglutination Inhibition Antibody Titers as a Correlate of Protection Against Seasonal A/H3N2 Influenza Disease. Open Forum Infect Dis 2015; 2(2): ofv067.

169. Berens-Norman H, Khatter S, Kohrt WM, et al. Anti-CCP3.1 and anti-CCP3-IgA antibodies are associated with increasing age in subjects without rheumatoid arthritis. Arthritis and Rheumatology 2016; 68(Supplement 10): 746-7.
170. Berera S, Naik J, Peyton A. A rare case of macro-AST. American Journal of Gastroenterology 2013; 108(SUPPL. 1): S343.
171. Berrih-Aknin S, Le Panse R. Myasthenia gravis and autoantibodies: Pathophysiology of the different subtypes. Revue de Medecine Interne 2014; 35(7): 413-20.
172. Besdine RW, Wetle TF. Improving health for elderly people: An international health promotion and disease prevention agenda. Aging Clinical and Experimental Research 2010; 22(3): 219-30.
173. Beth Smith ME, Haney E, McDonagh M, et al. Treatment of myalgic encephalomyelitis/chronic fatigue syndrome: A systematic review for a National Institutes of health pathways to prevention workshop. Annals of Internal Medicine 2015; 162(12): 841-50.
174. Bettini M, Gonorazky H, Chaves M, et al. Immune-mediated rippling muscle disease and myasthenia gravis. Journal of Neuroimmunology 2016; 299: 59-61.
175. Bettini M, Gonorazky H, Chaves M, et al. Clinical, serological and pathological findings of three patients with myasthenia gravis and immune rippling muscle disease in Argentina. Neurology 2013; 80(1 MeetingAbstracts).
176. Bettini M, Gonorazky H, Chaves M, et al. Immune-mediated rippling muscle disease and myasthenia gravis. J Neuroimmunol 2016; 299: 59-61.
177. Bezjak K, Matkovic U, Mis K, et al. Ouabain suppresses IL-6 signalling in cultured skeletal muscle cells. FEBS Open Bio 2021; 11(SUPPL 1): 221.
178. Bharadwaj A, Agrawal DK. Immunomodulation in asthma: A distant dream or a close reality? International Immunopharmacology 2004; 4(4): 495-511.
179. Bhatt VY, Stermer C, Hsu V, Sharma R. Oropharyngeal dysphagia: Rare presenting symptom of statin-induced hmg coa reductase necrotizing autoimmune myopathy. Journal of General Internal Medicine 2017; 32(2 Supplement 1): S556.
180. Bhattacharjee I, Bandyopadhyay A. Effects of acute supplementation of honey on endurance performance in Male university students. Indian Journal of Physiology and Pharmacology 2020; 64(1): 27-37.
181. Bhide M. It's all in the crescents-rapidly progressive glomerulonephritis. Journal of General Internal Medicine 2015; 30(SUPPL. 2): S416.
182. Bi F, Cady S, Yang K, et al. Effects of exercise on ovarian cancer initiation and progression. Clinical Cancer Research 2020; 26(13 SUPPL).
183. Bienek DR, Biagini RE, Charlton DG, Smith JP, Sammons DL, Robertson SA. Rapid point-of-care test to detect broad ranges of protective antigen-specific immunoglobulin G concentrations in recipients of the U.S.-licensed anthrax vaccine. Clin Vaccine Immunol 2008; 15(4): 644-9.
184. Biermann J, Herrmann W, Israel S. Changes of the immunoglobulins A, G and M in the serum by a four-week perseverance training in patients with cardiovascular diseases. Zeitschrift fur Physiotherapie 1989; 41(2): 103-7.
185. Bigley AB, Simpson RJ. NK cells and exercise: Implications for cancer immunotherapy and survivorship. Discovery Medicine 2015; 19(107): 433-45.
186. Bilò MB, Martini M, Tontini C, Corsi A, Antonicelli L. Anaphylaxis. Eur Ann Allergy Clin Immunol 2021; 53(1): 4-17.
187. Bimbetov B, Shanazarov N, Zhangabylov A, Aitbaeva S, Bakytzhanuly A. Mare's milk as a sport nutrition. International Journal of Pharmaceutical Research 2020; 12(4): 3368-72.
188. Bischoff W, Turner J, Russell GB, Blevins M, Stehle J. Evaluation of a novel powered air-purifying respirator (PAPR) vs. a N95 respirator mask for the protection against influenza in a human exposure model. Open forum infectious diseases 2017; 4: S168-.

189. Bishop NC, Billany R, Smith AC. Participant acceptability of exercise in kidney disease (PACE-KD): A feasibility study protocol in renal transplant recipients. BMJ Open 2017; 7(9): e017494.
190. Biver E. HIV and bone. Osteoporosis International 2018; 29(1 Supplement 1): S100.
191. Bjork S, Minkin R. LERONLIMAB AND THE ROLE OF CCR5 SUPRESSION IN COVID-19 TREATMENT. Chest 2021; 160(4 Supplement): A452.
192. Bjorklund G, Dadar M, Anderson G, Chirumbolo S, Maes M. Preventive treatments to slow substantia nigra damage and Parkinson's disease progression: A critical perspective review. Pharmacological Research 2020; 161: 105065.
193. Blajchman MA, Vamvakas EC. Prestorage versus poststorage white cell reduction for the prevention of the deleterious immunomodulatory effects of allogeneic blood transfusion. Transfusion Medicine Reviews 2000; 14(1): 23-33.
194. Blancou P, Chenciner N, Ho Tsong Fang R, et al. Simian immunodeficiency virus promoter exchange results in a highly attenuated strain that protects against uncloned challenge virus. J Virol 2004; 78(3): 1080-92.
195. Blazek A, Anderson P, Brichler J, et al. The effects of simulated intermittent altitude on mucosal immunity. FASEB Journal 2015; 29(Meeting Abstracts).
196. Block KI, Block PB, Gyllenhaal C. Integrative Treatment for Colorectal Cancer: A Comprehensive Approach. Journal of Alternative and Complementary Medicine 2018; 24(9-10): 890-901.
197. Blomberg J, Rizwan M, Böhlin-Wiener A, et al. Antibodies to Human Herpesviruses in Myalgic Encephalomyelitis/Chronic Fatigue Syndrome Patients. Front Immunol 2019; 10: 1946.
198. Bobinski F, Teixeira JM, Sluka KA, Soares Santos AR, Santos ARS. Interleukin-4 mediates the analgesia produced by low-intensity exercise in mice with neuropathic pain. PAIN 2018; 159(3): 437-50.
199. Boehncke WH, Brembilla NC. Autoreactive T-Lymphocytes in Inflammatory Skin Diseases. Front Immunol 2019; 10: 1198.
200. Boeke AJP. Advisory report from the Health Council of the Netherlands to include Human papillomavirus vaccination in the National Immunisation Programme for the prevention of cervical cancer. Nederlands Tijdschrift voor Geneeskunde 2008; 152(17): 981-3.
201. Bol K, Bloemendal M, Schreibelt G, et al. MIND-DC: A randomized phase III trial to assess the efficacy of adjuvant dendritic cell vaccination in comparison to placebo in stage IIIB and IIIC melanoma patients. Annals of Oncology 2020; 31(Supplement 4): S732.
202. Bolotovskii VM, Titova NS, Tamm OM, Vorob'eva AI, Blumberg II. Clinical and serologic diagnosis of measles in children vaccinated against the disease. Zhurnal mikrobiologii, epidemiologii, i immunobiologii 1977; (10): 91-7.
203. Bolz S, Totzeck A, Amann K, Stettner M, Kleinschnitz C, Hagenacker T. CIDP, myasthenia gravis, and membranous glomerulonephritis - three autoimmune disorders in one patient: a case report. BMC Neurology 2018; 18(1): N.PAG-N.PAG.
204. Bonamonte D, Foti C, Angelini G, Lionetti N, Rigano L. Photoprotection: New frontiers. Annali Italiani di Dermatologia Allergologica Clinica e Sperimentale 2009; 63(1): 10-27.
205. Boppart MD, Asp S, Wojtaszewski JF, Fielding RA, Mohr T, Goodyear LJ. Marathon running transiently increases c-Jun NH2-terminal kinase and p38 activities in human skeletal muscle. J Physiol 2000; 526 Pt 3(Pt 3): 663-9.
206. Bordowitz R, Morland K, Reich D. The use of an electronic medical record to improve documentation and treatment of obesity. Fam Med 2007; 39(4): 274-9.
207. Borgwardt L, Lund AM, Guffon N, et al. Impact of anti-drug antibodies (ADA) on safety and efficacy of velmanase alfa (human recombinant alpha-mannosidase) long-term enzyme replacement therapy in patients with alpha-mannosidosis. Journal of Inborn Errors of Metabolism and Screening 2017; 5: 362-3.
208. Bosak J, Pasowicz M, Moczulski Z, Zietek A, Stankiewicz Z, Nalepa P. Mounier-Kuhn syndrome (tracheobronchomegaly). Polski Merkuriusz Lekarski 2005; 19(109): 71-4.

209. Botwe BO, Antwi WK, Adusei JA, Mayeden RN, Akudjedu TN, Sule SD. COVID-19 vaccine hesitancy concerns: Findings from a Ghana clinical radiography workforce survey. Radiography (Lond) 2021.
210. Boyer FC, Tiffreau V, Rapin A, et al. Post-polio syndrome: Pathophysiological hypotheses, diagnosis criteria, drug therapy. Ann Phys Rehabil Med 2010; 53(1): 34-41.
211. Bozlu G, Cobanogullari Direk M, Okuyaz C. Subacute sclerosing panencephalitis with parkinsonian features in a child: A case report. Brain and Development 2015; 37(9): 901-3.
212. Bradford D. Ethan Vernon. Cycling Weekly 2021; (317): 52-3.
213. Braghieri A, Pacelli C, De Rosa G, Girolami A, De Palo P, Napolitano F. Podolian beef production on pasture and in confinement. Animal 2011; 5(6): 927-37.
214. Brainum J. Allergies nothing to sneeze at. Joe Weider's Muscle & Fitness 1993; 54(8): 108.
215. Brand A, Novotny V, Tomson B. Platelet Transfusion Therapy: From 1973 to 2005. Human Immunology 2006; 67(6): 413-8.
216. Braun J, Brandt J, Sieper I. Therapie mit monoklonalen Antikoerpern gegen TNFalpha bei Spondylarthropathien und anderen rheumatischen Erkrankungen. / Therapy with monoclonal antibodies against TNFalpha in ankylosing spondylitis and other rheumatic diseases. Deutsche Zeitschrift fuer Sportmedizin 2001; 52(2): 50-6.
217. Brembilla NC, Boehncke W-H. Autoreactive T-lymphocytes in inflammatory skin diseases. Frontiers in Immunology 2019; 10(MAY): 1198.
218. Briët E, Mauser-Bunschoten EP. [Revision consensus hemophilia: treatment and responsibility. Nederlandse Vereniging van Hemophilia Patients]. Ned Tijdschr Geneeskd 1997; 141(52): 2566-71.
219. Briggs N, Bottazzi ME, Beaumier C, Sastry J, Hotez P. Immunological characterization of human hosts to Ascaris lumbricoides and Trichuris trichiura infection in a population living in the rural municipality of Colomoncagua, Honduras (MPF6P.654). Journal of Immunology 2015; 194(1 SUPPL. 1).
220. Broadbent S, Coutts R. The protocol for a randomised controlled trial comparing intermittent and graded exercise to usual care for chronic fatigue syndrome patients. BMC Sports Science, Medicine and Rehabilitation 2013; 5(1): 16.
221. Brockmann SO, Dreweck C, Wagner-Wiening C, et al. Serological and epidemiological analysis of an outbreak of gastroenteritis among military recruits in Germany caused by Cryptosporidium parvum. Infection 2008; 36(5): 450-7.
222. Broomfield MA, Doyle EK, Kahn LP, Smith WD, Walkden-Brown SW. A simplified Barbervax® vaccination regimen in lambs to evoke immunological protection to Haemonchus contortus. Vet Parasitol 2020; 287: 109243.
223. Brown LD, Vasquez D, Salinas JJ, Tang X, Balcázar H. Evaluation of Healthy Fit: A Community Health Worker Model to Address Hispanic Health Disparities. Prev Chronic Dis 2018; 15: E49.
224. Brown P, Rutherford B, Roose S, Haroon E. Slowing, inflammation, and depression: Implications for assessment and treatment of older depressed individuals. American Journal of Geriatric Psychiatry 2016; 24(3 Supplement 1): S9-S10.
225. Brzezіańska E, Domańska D, Jegier A. GENE DOPING IN SPORT - PERSPECTIVES AND RISKS. Biology of Sport 2014; 31(4): 251-9.
226. Bubnov RV, Babenko L, Kalika L. Dynamic ultrasound for multilevel evaluation of motion and posture in lower extremity and spine. Annals of the Rheumatic Diseases 2018; 77(Supplement 2): 1699.
227. Bubnov RV, Spivak MY. Short-term probiotic treatment improve gut function, increase bowel wall thickness and alleviate abdominal signs of metabolic syndrome. Neurogastroenterology and Motility 2019; 31(Supplement 4).

228. Buczylko K. The role of avoidance of seasonal allergens in allergies management. Alergia Astma Immunologia 2011; 16(1): 17-23.
229. Buford TW, Willoughby DS. Impact of DHEA(S) and cortisol on immune function in aging: A brief review. Applied Physiology, Nutrition and Metabolism 2008; 33(3): 429-33.
230. Bulechek GM, McCloskey JC. Nursing interventions classification (NIC). Medinfo 1995; 8 Pt 2: 1368.
231. Bults M, Beaujean DJ, de Zwart O, et al. [Mexican flu: risk perception in the general public, precautionary measures and trust in information provided by the government]. Ned Tijdschr Geneeskd 2010; 154: A1686.
232. Burgess T, Richard S, Collins L, et al. Pragmatic assessment of influenza vaccine effectiveness in the DOD (PAIVED): influenza-like-illness rates in year 1. Open forum infectious diseases 2019; 6: S990-.
233. Burke DT, Al-Adawi S, Lee YT, Audette J. Martial arts as sport and therapy. Journal of Sports Medicine & Physical Fitness 2007; 47(1): 96-102.
234. Burnett R, Hamish Simpson A, Harasymowicz NS, Salter DM. Articular adipose tissue macrophages at the crossroad between obesity and osteoarthritis. Journal of Orthopaedic Research 2016; 34(Supplement 1).
235. Burns VE, Ring C, Drayson M, Carroll D. Cortisol and cardiovascular reactions to mental stress and antibody status following hepatitis B vaccination: A preliminary study. Psychophysiology 2002; 39(3): 361-8.
236. Buss LA, Williams T, Hock B, et al. Effects of exercise and anti-PD-1 on the tumour microenvironment. Immunol Lett 2021; 239: 60-71.
237. Butler SA, Heyer N, Burczynska B, Iles RK. Human antiserum against hCGbeta induces bladder cancer cell death in vitro. Tumor Biology 2011; 32(SUPPL. 1): S108-S9.
238. Butnaru D, Chapman J. The impact of self-replicating proteins on inflammation, autoimmunity and neurodegeneration-An untraveled path. Autoimmunity Reviews 2019; 18(3): 231-40.
239. Byles JE. How do the psychosocial consequences of ageing affect asthma management? The Medical journal of Australia 2005; 183(1 Suppl): S30-2.
240. Cabanillas F, Horning S, Kaminski M, Champlin R. Managing Indolent Lymphomas in Relapse: Working Our Way Through a Plethora of Options. Hematology Am Soc Hematol Educ Program 2000: 166-79.
241. Cacione DG, Baptista-Silva JC, Macedo CR. Pharmacological treatment for Buerger's disease. Cochrane Database Syst Rev 2016; 2: Cd011033.
242. Cacione DG, Macedo CR, do Carmo Novaes F, Baptista-Silva JC. Pharmacological treatment for Buerger's disease. Cochrane Database Syst Rev 2020; 5(5): Cd011033.
243. Cacione DG, Moreno DH, do Carmo Novaes F. Stem cell therapy for treatment of thromboangiitis obliterans (Buerger's disease). Cochrane Database of Systematic Reviews 2018; 2018(10): CD012794.
244. Cadden JJ, Loomis KA, Kallia R, Dube MP, Louie S. Anti-inflammatory effects of arabinoxylan rice bran supplementation in participants with treated, suppressed HIV infection and inadequate immune reconstitution: a randomized, doubleblind trial. Antiviral Therapy 2020; 25(SUPPL 1): A26.
245. Caffarelli C, Santamaria F, Vottero A, Bernasconi S. Progress in Pediatrics in 2012: choices in allergy, endocrinology, gastroenterology, hematology, infectious diseases, neurology, nutrition and respiratory tract illnesses. Ital J Pediatr 2013; 39: 26.
246. Calabrese LH, Kleiner SM, Barna BP, et al. The effects of anabolic steroids and strength training on the human immune response. / Les effets des steroides anabolisants et de l'entrainement de musculation sur la reponse immunitaire chez l'homme. Medicine & Science in Sports & Exercise 1989; 21(4): 386-92.

247. Calder PC, Kew S. The immune system: A target for functional foods? British Journal of Nutrition 2002; 88(SUPPL. 2): S165-S76.
248. Campagnolo M, Cagnin A, Zara G, et al. Acute onset of CMT1A. Journal of the Peripheral Nervous System 2011; 16(SUPPL. 2): S6.
249. Cannon J, Micalos P, Pak S. A study protocol to evaluate a fermented rice bran supplement and resistance training on immune function and muscle performance in healthy older people. Journal of Science and Medicine in Sport 2019; 22(Supplement 2): S79.

250. Capurso L. Thirty Years of Lactobacillus rhamnosus GG A Review. Journal of Clinical Gastroenterology 2019; 53(Supplement 1): S1-S41.
251. Cardoso CRL, Leite NC, Carlos FO, Loureiro AA, Viegas BB, Salles GF. Efficacy and Safety of Diacerein in Patients With Inadequately Controlled Type 2 Diabetes: a Randomized Controlled Trial. Diabetes care 2017; 40(10): 1356-63.
252. Carling C, Gregson W, McCall A, Moreira A, Wong D, Bradley P. Match Running Performance During Fixture Congestion in Elite Soccer: Research Issues and Future Directions. Sports Medicine 2015; 45(5): 605-13.
253. Carmona MS, Tillman R, Forbes L, et al. Granulomatous lymphocytic interstitial lung disease in a pediatric case of common variable immunodeficiency. American Journal of Respiratory and Critical Care Medicine 2017; 195.
254. Carrasco E, Gomez-Gutierrez P, Campos PM, Vega M, Messeguer A, Perez JJ. Discovery of novel 2,3,5-trisubstituted pyridine analogs as potent inhibitors of IL-1β via modulation of the p38 MAPK signaling pathway. Eur J Med Chem 2021; 223: 113620.
255. Carron T, Bridevaux PO, Lörvall K, et al. Feasibility, acceptability and effectiveness of integrated care for COPD patients: a mixed methods evaluation of a pilot community-based programme. Swiss Med Wkly 2017; 147: w14567.
256. Carson B, Shetty N, Trikamji B. A unique manifestation of GD1A antibody associated acute motor sensory axonal neuropathy. Annals of Neurology 2021; 90(SUPPL 27): S205.
257. Cary TL, Ortiz-Santaliestra ME, Karasov WH. Immunomodulation in post-metamorphic northern leopard frogs, Lithobates pipiens, following larval exposure to polybrominated diphenyl ether. Environ Sci Technol 2014; 48(10): 5910-9.
258. Casadevall A, Pirofski L-A. The potential of antibody-mediated immunity in the defence against biological weapons. Expert Opinion on Biological Therapy 2005; 5(10): 1359-72.
259. Castaneda-Orjuela C, De la Hoz-Restrepo F, Romero M, et al. Using standardized tools to improve immunization costing data for program planning: The cost of the Colombian Expanded Program on Immunization. Vaccine 2013; 31(SUPPL.3): C72-C9.
260. Catherine C, Seese R, Waters J. Acute disseminated encephalomyelitis following mycoplasma pneumoniae infection in a young adult collegiate athlete. Neurology 2019; 92(15 Supplement 1).
261. Catotti V, Sardo G, Di Martino G, Ciancio N, Di Maria G. The efficacy of COPD patients education: A paired matched case-control study. European Respiratory Journal 2014; 44(SUPPL. 58).
262. Cavus H, Aydinlik S, Iseri P. Bickerstaff's brainstem encephalitis. European Journal of Neurology 2016; 23(SUPPL. 2): 337.
263. Cernak CR, Odell RH, Marriott E, Silvani B. Combination electrochemical therapy (CET) to treat patients with diabetic neuropathy. Regional Anesthesia and Pain Medicine 2010; 35(5).
264. Cha DS, McIntyre RS, Filteau M-J, et al. Treatment-resistant depression: Definitions, review of the evidence, and algorithmic approach. Journal of Affective Disorders 2014; 156: 1-7.
265. Chaiyakunapruk N, Somkrua R, Hutubessy R, et al. Cost effectiveness of pediatric pneumococcal conjugate vaccines: a comparative assessment of decision-making tools. BMC Medicine 2011; 9(1): 53-.
266. Chamberlain P, Kurki P. Immunogenicity Assessment of Biosimilars: A Multidisciplinary Perspective. AAPS Advances in the Pharmaceutical Sciences Series 2018; 34: 489-542.

267. Chamorro-Viña C, Ruiz JR, Santana-Sosa E, et al. Exercise during Hematopoietic Stem Cell Transplant Hospitalization in Children. Medicine & Science in Sports & Exercise 2010; 42(6): 1045-53.
268. Chan CL, Wang CW, Ho RT, et al. A systematic review of the effectiveness of qigong exercise in supportive cancer care. Support Care Cancer 2012; 20(6): 1121-33.
269. Chan FWK, Wong CK, Chan PKS, et al. Chronic psychosocial stress: Does it modulate immunity to the influenza vaccine in Hong Kong Chinese elderly caregivers? Age 2013; 35(4): 1479-93.
270. Chang Y-J, Avitia Y, Godbole S, et al. Circulating extracellular vesicles as novel biomarkers of cardiovascular risk in older women with different sitting time patterns. Circulation Research 2019; 125(Supplement 1).
271. Chapman KR. Therapeutic algorithm for chronic obstructive pulmonary disease. American Journal of Medicine 1991; 91(4 A): 4A-23S.
272. Chapman KR, Bowie DM, Goldstein RS, et al. Guidelines for the assessment and management of chronic obstructive pulmonary disease. CMAJ 1992; 147(4): 420-8.
273. Charles C, Bardet A, Ibrahimi N, et al. Delivering adapted physical activity by videoconference to patients with fatigue under immune checkpoint inhibitors: Lessons learned from the PACTIMe-FEAS feasibility study. J Telemed Telecare 2021: 1357633x211021743.
274. Charlton DG, Biagini RE, Smith JP, Sammons DL, Robertson SA, Bienek DR. Rapid point-of-care test to detect broad ranges of protective antigen-specific immunoglobulin G concentrations in recipients of the U.S.-licensed anthrax vaccine. Clinical and Vaccine Immunology 2008; 15(4): 644-9.
275. Chastin SFM, Abaraogu U, Bourgois JG, et al. Effects of Regular Physical Activity on the Immune System, Vaccination and Risk of Community-Acquired Infectious Disease in the General Population: Systematic Review and Meta-Analysis. Sports Medicine 2021; 51(8): 1673-86.
276. Chavez JC, Goldschmidt N, Samaniego F, et al. The Combination of Umbralisib Plus Ublituximab Is Active in Patients with Relapsed or Refractory Marginal Zone Lymphoma (MZL): Results from the Phase 2 Global Unity-NHL Trial. Blood 2021; 138(Supplement 1): 45.
277. Cheent K, Nolan J, Shariq S, Kiho L, Pal A, Arnold J. Case Report: Fatal case of disseminated BCG infection in an infant born to a mother taking infliximab for Crohn's disease. J Crohns Colitis 2010; 4(5): 603-5.
278. Chen M, Raj R, Fox L, et al. Is there a role for physical activity when treating patients with cancer with immune checkpoint inhibitors? Protocol for a scoping review. BMJ Open 2021; 11(10): e046052.
279. Chen S, Fan X-R, He S, Zhang J-W, Li S-J. Watch out for neuromyelitis optica spectrum disorder after inactivated virus vaccination for COVID-19. Neurological Sciences 2021; 42(9): 3537-9.
280. Chen X, Mao G, Leng SX. Frailty syndrome: An overview. Clinical Interventions in Aging 2014; 9: 433-41.
281. Chen Z, Gu K, Zheng Y, Zheng W, Lu W, Shu XO. The use of complementary and alternative medicine among Chinese women with breast cancer. Journal of Alternative & Complementary Medicine 2008; 14(8): 1049-55.
282. Cherif Y, Mrouki M, Ben Dahmen F, Abdallah M, Amri R. Anti signal recognition particle antibodies: Not that bad outcome! Osteoporosis International 2018; 29(1 Supplement 1): S395-S6.
283. Cherqaoui B, Kone-Paut I, Bader-Meunier B, Delacourt C, Hadchouel-Duverge A. Characterization of patients with polyarticular idiopathic juvenile arthritis and infiltrative lung disease. Pediatric Rheumatology 2018; 16(Supplement 2).
284. Chevalier K, Noel N, Benoudiba F, Chrétien P, Hacein-Bey-Abina S, Lambotte O. Anti-Ma2 antibody encephalitis associated with Sjogren's syndrome. Rev Med Interne 2021; 42(8): 575-8.
285. ChiCtr. A randomized, double-blind, placebo-controlled, single-center trial for the efficacy and safety of Bufei Huoxue Capsule in the treatment of chronic obstructive pulmonary disease. https://trialsearchwhoint/Trial2aspx?TrialID=ChiCTR1800018910 2018.

286. ChiCtr. A randomized, controlled, open, multi-center clinical study on the effectiveness and safety of Kangai injection in the treatment of cancer-induced fatigue. https://trialsearchwhoint/Trial2aspx?TrialID=ChiCTR2100043661 2021.
287. Chien KT, Chen CC, Chu CM, Chan YL, Chung HY, Chang TK. The Determination of Efficacy of CircuCare on Blood Circulation and Metabolism: An Animal Model Study. Comput Math Methods Med 2021; 2021: 9934107.
288. Childress P, Bidwell JP, Robling AG. Nmp4/CIZ: Road block at the intersection of PTH and load. Bone 2010; 46(2): 259-66.
289. Chin Leong L, Mackinnon LT. The Roles of Exercise-Induced Immune System Disturbances in the Pathology of Heat Stroke: The Dual Pathway Model of Heat Stroke. Sports Medicine 2006; 36(1): 39-64.
290. Ching-Lung L, Man-Fung Y. Entecavir: A Viewpoint by Ching-Lung Lai and Man-Fung Yuen. Drugs 2006; 66(12): 1623-4.
291. Chinta VR, Murgod U, Maloo R. Autoimmune glutamic acid decarboxylase 65 antibody associated cerebellar ataxia. Annals of Indian Academy of Neurology 2019; 22(SUPPL 1): S45.
292. Cho HJ, Perumal D, Parekh S, et al. Genomic and Immunologic Analysis of Cmaf and Hypermutated Multiple Myeloma: Implications for Immunologic Therapy. Blood 2019; 134(Supplement 1): 3093.
293. Chokshi K, Jajoo P. Jack all of trades, master of all: Cannabis for management of COVID-19 long haul sequelae. a case report. PM and R 2021; 13: S166-S7.
294. Choudhuri D, Huda T, Theodoratou E, et al. An evaluation of emerging vaccines for childhood meningococcal disease. BMC Public Health 2011; 11 Suppl 3(Suppl 3): S29.
295. Chowdhury MA, Hossain N, Kashem MA, Shahid MA, Alam A. Immune response in COVID-19: A review. Journal of Infection and Public Health 2020; 13(11): 1619-29.
296. Christian LM. Optimizing benefits of influenza virus vaccination during pregnancy: Potential behavioral risk factors and interventions. Vaccine 2014; 32(25): 2958-64.
297. Chuansumrit A, Sirachainan N, Kadegasem P, et al. Effectiveness of Monthly Low-Dose Emicizumab Prophylaxis without 4-Week Loading Doses Among Patients with Hemophilia a with and without Inhibitor: A Case Series Report. Blood 2021; 138(Supplement 1): 2116.
298. Chung Y, Hsiao YT, Huang WC. Physiological and Psychological Effects of Treadmill Overtraining Implementation. Biology (Basel) 2021; 10(6).
299. Church MK, Máspero JF, Maurer M, Ryan D. The Scope of Pharmacological and Clinical Effects of Modern Antihistamines, With a Special Focus on Rupatadine: Proceedings from a Satellite Symposium held at the 21st World Allergy Congress, Buenos Aires, December 8, 2009. World Allergy Organ J 2010; 3(4 Suppl): S1-s16.
300. Cianferoni A. Wheat allergy: Diagnosis and management. Journal of Asthma and Allergy 2016; 9: 13-25.
301. Cicchella A, Stefanelli C, Massaro M. Upper Respiratory Tract Infections in Sport and the Immune System Response. A Review. Biology (Basel) 2021; 10(5).
302. Cinel G, Emiralioglu N, Kiper N, et al. When to suspect hypersensitivity pneumonia? American Journal of Respiratory and Critical Care Medicine 2015; 191(MeetingAbstracts).
303. Civoli F, Kasinath A, Cai XY, et al. Recommendations for the Development and Validation of Immunogenicity Assays in Support of Biosimilar Programs. Aaps j 2019; 22(1): 7.
304. Clark AL, Boroujerdi-Rad L. Acute myopericarditis after tetanus, diptheria, and pertussis vaccination in a healthy adult. Journal of General Internal Medicine 2014; 29(SUPPL. 1): S298.
305. Clements CJ, Nshimirimanda D, Gasasira A. Using immunization delivery strategies to accelerate progress in Africa towards achieving the Millennium Development Goals. Vaccine 2008; 26(16): 1926-33.
306. Cluskey MD. Maternal self-efficacy and maternal perception of health as predictors of healthy lifestyles in young children from low income families: Catholic University of America; 1999.

307. Coates C, Meisman A, Gordon C, Chandler E, Braverman P. Evaluation of A Health Education Module About Accessing Community Based Healthcare Services For Youth In A Juvenile Detention Facility. Journal of Adolescent Health 2019; 64(2 Supplement): S58-S9.
308. Cockrell GE, Helm RM, Burks AW, Kelso JM. Common allergens in avian meats. Journal of Allergy and Clinical Immunology 1999; 104(1): 202-4.
309. Cohen S, Pressman S, Barkin A, Rabin BS, Treanor JJ, Miller GE. Psychological Stress and Antibody Response to Influenza Vaccination: When Is the Critical Period for Stress, and How Does It Get Inside the Body? Psychosomatic Medicine 2004; 66(2): 215-23.
310. Cohen-Solal JF, Cassard L, Fournier EM, Loncar SM, Fridman WH, Sautès-Fridman C. Metastatic melanomas express inhibitory low affinity fc gamma receptor and escape humoral immunity. Dermatol Res Pract 2010; 2010: 657406.
311. Colbath AC, Frisbie DD, Dow SW, Kisiday JD, McIlwraith CW, Goodrich LR. Equine Models for the Investigation of Mesenchymal Stem Cell Therapies in Orthopaedic Disease. Operative Techniques in Sports Medicine 2017; 25(1): 41-9.
312. Coleman MD. Dapsone toxicity: Some current perspectives. General Pharmacology 1995; 26(7): 1461-7.
313. Collins M, Kong W, Jung I, et al. A Failure to Start: Aborted Activation of CAR T Cells in Chronic Lymphocytic Leukemia. Blood 2019; 134(Supplement 1): 681.
314. Colpo GD, Leboyer M, Dantzer R, Trivedi MH, Teixeira AL. Immune-based strategies for mood disorders: facts and challenges. Expert Rev Neurother 2018; 18(2): 139-52.
315. Cong M-H, Zou B-H, Yu L. Mechanisms of anorexia cancer cachexia syndrome and potential benefits of traditional medicine and natural herbs. Current Pharmaceutical Biotechnology 2016; 17(13): 1147-52.
316. Constantine GM, Ferre E, Lionakis M. Specific functional gammopathy underlying infectious susceptibility in a patient with autoimmune-polyendocrinopathy-candidiasis-ectodermal dystrophy (APECED). Journal of Clinical Immunology 2019; 39(Supplement 1): S111-S2.
317. Cooksley G, John TJ, Chan VF, et al. Hepatitis B vaccine boosters: Is there a clinical need in high endemicity populations? Journal of Gastroenterology and Hepatology (Australia) 2005; 20(1): 5-10.
318. Corcillo A, Fountoulakis N, Maltese G, et al. Painless foot drop: An unusual acute presentation of new onset type 1 diabetes mellitus. Endocrinology, Diabetes and Metabolism Case Reports 2021; 2021(1): 21-0012.
319. Cornelia E, Andrea K, Andreas W, Patricia H. Unusual presentation of myasthenia gravis with facial and bulbar symptoms. Swiss Medical Weekly 2019; 149(Supplement 235): 27S.
320. Costello N, McKenna J, Sutton L, Deighton K, Jones B. Using contemporary behavior change science to design and implement an effective nutritional intervention within professional rugby league. International Journal of Sport Nutrition and Exercise Metabolism 2018; 28(5): 553-7.
321. Coudert C, Beau F, Berlioz-Arthaud A, et al. [Human leptospirosis in French Polynesia. Epidemiological, clinical and bacteriological features]. Med Trop (Mars) 2007; 67(2): 137-44.
322. Couri B, Kasi R. Parsonage-turner syndrome secondary to influenza vaccine: A case report. PM and R 2012; 4(10 SUPPL. 1): S289-S90.
323. Covaleski APPM, Mota VVDL, Lins OG, Marques W. A case of neuromyotonia associated with a chronic polyradiculoneuropathy. Journal of Neuromuscular Diseases 2016; 3(Supplement 1): S88-S9.
324. Cox AJ, Gleeson M, Pyne DB, Saunders PU, Clancy RL, Fricker PA. Valtrex therapy for Epstein-Barr virus reactivation and upper respiratory symptoms in elite runners. Medicine & Science in Sports & Exercise 2004; 36(7): 1104-10.
325. Cramer H, Lauche R, Klose P, Dobos G, Langhorst J. A systematic review and meta-analysis of exercise interventions for colorectal cancer patients. European journal of cancer care 2014; 23(1): 3-14.

326. Critchlow C, Vittinghoff E, Coletti AS, et al. Home collection for frequent HIV testing: Acceptability of oral fluids, dried blood sports and telephone results. AIDS 2000; 14(12): 1819-28.
327. Crooks GM, Mackall C, Weinberg K. Immune reconstitution: From stem cells to lymphocytes. Biology of Blood and Marrow Transplantation 2006; 12(SUPPL. 1): 42-6.
328. Cruz-Jentoft AJ, Romero-Yuste S, Chamizo Carmona E, Nolla JM. Sarcopenia, immune-mediated rheumatic diseases, and nutritional interventions. Aging Clinical and Experimental Research 2021; 33(11): 2929-39.
329. Ctri. Evaluation of hydrotherapy versus land-based exercise on rheumatoid arthritis. https://trialsearchwhoint/Trial2aspx?TrialID=CTRI/2021/03/032360 2021.
330. Cuchet D, Epstein AL, Ferrera R, Lomonte P. Characterization of antiproliferative and cytotoxic properties of the HSV-1 immediate-early ICPo protein. Journal of Gene Medicine 2005; 7(9): 1187-99.
331. Cunningham AJH, Fazleabas AT, Braundmeier AG, Markham R, Fraser IS, Berbic M. Immune cell mapping in the baboon endometriosis model. Journal of Endometriosis 2012; 4(3): 166.
332. Cunningham-Erves J, Campbell L, Barlow C, et al. Reducing HPV Associated Cancers and Disparities: Engaging African American Men to Develop a Culturally-Appropriate Program that Addresses their Needs. American Journal of Health Education 2021; 52(4): 194-206.
333. Curin Serbec V. Toward a therapy of prion diseases. Vox Sanguinis 2010; 99(SUPPL. 1): 318.
334. Curinserbec V, Hartman KP, Vranac T, et al. Vaccine development in prion diseases. Immunology 2012; 137(SUPPL. 1): 771-2.
335. Curran D, Standaert BA, Postma MJ. Budget constraint and vaccine dosing: A mathematical modelling exercise. Cost Effectiveness and Resource Allocation 2014; 12(1): 3.
336. Cuschieri K, Cubie H, Moore C, et al. Effect of HPV assay choice on perceived prevalence in a population-based sample. Diagnostic Molecular Pathology 2013; 22(2): 85-90.
337. Cusi K. Role of obesity and lipotoxicity in the development of nonalcoholic steatohepatitis: Pathophysiology and clinical implications. Gastroenterology 2012; 142(4): 711.
338. Cutler EC. THE RELATION OF THE HYPOPHYSIS TO ANTIBODY PRODUCTION. J Exp Med 1922; 35(2): 243-56.
339. Cuzzubbo S, Mangsbo S, Nagarajan D, Habra K, Pockley AG, McArdle SEB. Cancer Vaccines: Adjuvant Potency, Importance of Age, Lifestyle, and Treatments. Frontiers in Immunology 2020; 11: 615240.
340. D'Arienzo M, Marrese C, Sergiacomi P, Baroni D. Clinical effects of docosahexanoic acid (DHA) as corticosteroids-sparing drug in PMR patients at high risk for CS-related adverse events: Preliminary results. Osteoporosis International 2015; 26(1 SUPPL. 1): S211-S2.
341. Da Silva Rodrigues De Araujo C, De Araujo AAC, MacHado BA, et al. Severe intravenous immunoglobulin-induced hemolysis: A case report. Transfusion 2018; 58(Supplement 2): 201A.
342. Dacko C, Holley JL. The influence of nutritional status, dialysis adequacy, and residual renal function on the response to hepatitis B vaccination in peritoneal dialysis patients. Advances in peritoneal dialysis Conference on Peritoneal Dialysis 1996; 12: 315-7.
343. Dahan A, Dunne A, Swartjes M, et al. ARA 290 improves symptoms in patients with sarcoidosis-associated small nerve fiber loss and increases corneal nerve fiber density. Molecular medicine (Cambridge, Mass) 2013; 19: 334-45.
344. Dahlui M, Alkoshi S, Maimaiti N, Baudouin S. Cea of introducing rotavirus vaccine in Libya. Value in Health 2014; 17(7): A679.
345. Dai H, Han J, Lichtfouse E. Smarter cures to combat COVID-19 and future pathogens: a review. Environ Chem Lett 2021: 1-13.
346. Dalakas MC. Pathogenetic mechanisms of post-polio syndrome: Morphological, electrophysiological, virological, and immunological correlations. Annals of the New York Academy of Sciences 1995; 753: 167-85.

347. Dallari D, Stagni C, Rani N, et al. Ultrasound-Guided Injection of Platelet-Rich Plasma and Hyaluronic Acid, Separately and in Combination, for Hip Osteoarthritis. American Journal of Sports Medicine 2016; 44(3): 664-71.
348. Daly L, Cushen S, Ryan A, et al. The impact of body composition parameters on ipilimumab toxicity in metastatic melanoma and longitudinal changes in body composition during treatment. European Journal of Cancer 2015; 51(SUPPL. 3): S679.
349. Daniilidis M, Kouidi E, Giagoudaki F, et al. The immune response in hemodialysis patients following physical training. Sport Sciences for Health 2004; 1(1): 11-6.
350. Dapp U, Anders J, Meier-Baumgartner HP, v Renteln-Kruse W. Geriatric health promotion and prevention for independently living senior citizens: programmes and target groups. Zeitschrift fur Gerontologie und Geriatrie 2007; 40(4): 226-40.
351. Darras BT, Urion DK, Ghosh PS. Dystrophinopathies. In: Adam MP, Ardinger HH, Pagon RA, et al., eds. GeneReviews(®). Seattle (WA): University of Washington, Seattle
Copyright © 1993-2022, University of Washington, Seattle. GeneReviews is a registered trademark of the University of Washington, Seattle. All rights reserved.; 1993.
352. David E, At the B, Giladi A, et al. XCR1+ type 1 conventional dendritic cells drive liver pathology in non-alcoholic steatohepatitis. Nature Medicine 2021; 27(6): 1043-54.
353. Davis JM, Weaver JA, Kohut ML, Colbert LH, Ghaffar CA, Mayer EP. Immune system activation and fatigue during treadmill running: role of interferon. / Activation du systeme immunitaire et fatigue pendant une course sur tapis roulant: role de l ' interferon. Medicine & Science in Sports & Exercise 1998; 30(6): 863-8.
354. Day CL, Tameris M, Mansoor N, et al. Induction and regulation of T-cell immunity by the novel tuberculosis vaccine M72/AS01 in South African adults. American Journal of Respiratory & Critical Care Medicine 2013; 188(4): 492-502.
355. de Araújo AL, Silva LC, Fernandes JR, Benard G. Preventing or reversing immunosenescence: can exercise be an immunotherapy? Immunotherapy 2013; 5(8): 879-93.
356. De Gomensoro E, Del Giudice G, Doherty MT. Challenges in adult vaccination. European Geriatric Medicine 2018; 9(Supplement 1): S80.
357. De Haas M, Van Der Schoot CE, Van Der Ploeg CPB, Abbink F. Noninvasive prenatal screening for RHD in The Netherlands: One test for targeted antenatal and postnatal anti-d prophylaxis. Vox Sanguinis 2012; 103(SUPPL. 1): 33.
358. de Jesus Leite MA, Gonçalves Á, Portari G, et al. Application of physical exercise therapies in breast cancer survivors and their effects on the inflammatory profile: A narrative review. Journal of Bodywork & Movement Therapies 2020; 24(4): 536-45.
359. De Kok IMCM, Habbema JDF, Coebergh JWW, Mourits MJE, Van Leeuwen FE. Insufficient basis for the inclusion of Human papillomavirus vaccination in the National Immunisation Programme in the Netherlands. Nederlands Tijdschrift voor Geneeskunde 2008; 152(37): 2001-4.
360. de Lejarazu-Leonardo RO, Montomoli E, Wojcik R, et al. Estimation of reduction in influenza vaccine effectiveness due to egg-adaptation changes-systematic literature review and expert consensus. Vaccines 2021; 9(11): 1255.
361. De Silva M, Tjoelker L, Chang CF, Reeves JJ. Ovarian response to active immunization against luteinizing hormone in the cow. Theriogenology 1986; 26(1): 89-100.
362. De Sousa Fernandes MS, De Lira CTC, De Lucena Simoes e Silva L, et al. Can Physical Activity Help in Hepatitis C Treatment? A Mini Review. Journal of Gastroenterology and Hepatology Research 2019; 8(1): 2789-92.
363. De Souza Bezerra RL, De Medeiros KN. Follow up of a patient with antiphospholipid syndrome (APS): A case report. Advances in Rheumatology 2018; 58(Supplement 1).
364. de Souza JM, de Oliveira DS, Perin LA, et al. Feasibility, safety and efficacy of exercise training in immune-mediated necrotising myopathies: a quasi-experimental prospective study. Clinical and experimental rheumatology 2019; 37(2): 235-41.

365. De Visser M. Progress in the treatment of idiopathic inflammatory myopathies? Journal of Neuromuscular Diseases 2018; 5(Supplement 1): S10-S1.
366. Debas HT, Laxminarayan R, Straus SE. Complementary and Alternative Medicine. In: Jamison DT, Breman JG, Measham AR, et al., eds. Disease Control Priorities in Developing Countries. Washington (DC)
New York: The International Bank for Reconstruction and Development / The World Bank
Oxford University Press
Copyright © 2006, The International Bank for Reconstruction and Development/The World Bank Group.; 2006.
367. Deczkowska A, David E, Ramadori P, et al. XCR1(+) type 1 conventional dendritic cells drive liver pathology in non-alcoholic steatohepatitis. Nat Med 2021; 27(6): 1043-54.
368. DeGroot LJ. Graves' Disease and the Manifestations of Thyrotoxicosis. In: Feingold KR, Anawalt B, Boyce A, et al., eds. Endotext. South Dartmouth (MA): MDText.com, Inc.
Copyright © 2000-2022, MDText.com, Inc.; 2000.
369. Degu G, Mengistu G, Jones J. Some factors affecting prevalence of and immune responses to Schistosoma mansoni in schoolchildren in Gorgora, northwest Ethiopia. Ethiop Med J 2002; 40(4): 345-52.
370. Dejardin LM, Arnoczky SP, Ewers BJ, Haut RC, Clarke RB. Tissue-engineered rotator cuff tendon using porcine small intestine submucosa: histologic and mechanical evaluation in dogs. American Journal of Sports Medicine 2001; 29(2): 175-84.
371. Dekker J, Prins J, Jassim G, Bourke L, Boinon D, Aaronson N. The evidence base for psychosocial interventions in oncology-the way forward. Psycho-Oncology 2016; 25(Supplement 3): 18-9.
372. Del Molino Del Barrio I, Hayday TS, Laing AG, Hayday AC, Di Rosa F. COVID-19: Using high-throughput flow cytometry to dissect clinical heterogeneity. Cytometry A 2021.
373. Delgado L, Moreira A, Kekkonen RA, Fonseca J, Korpela R, Haahtela T. Nutritional modulation of exercise-induced immunodepression in athletes: A systematic review and meta-analysis. European Journal of Clinical Nutrition 2007; 61(4): 443-60.
374. Deshpande RP, Sharma S, Watabe K. The Confounders of Cancer Immunotherapy: Roles of Lifestyle, Metabolic Disorders and Sociological Factors. Cancers 2020; 12(10): 2983.
375. Dhaiban S, Al-Ani M, Elemam NM, Maghazachi AA. Targeting chemokines and chemokine receptors in multiple sclerosis and experimental autoimmune encephalomyelitis. Journal of Inflammation Research 2020; 13: 619-33.
376. Di Raimondo D, Musiari G, Miceli G, Arnao V, Pinto A. Preventive and Therapeutic Role of Muscle Contraction Against Chronic Diseases. Curr Pharm Des 2016; 22(30): 4686-99.
377. Di Santo A, Pilato F, Capone F, Di Lazzaro V, Africa L. AchR myasthenia gravis (MG) atypical presentation in human immunodeficiency virus (HIV) positive patient. Journal of the Neurological Sciences 2021; 429(Supplement): 118363.
378. Dias KA, Link MS, Levine BD. Exercise Training for Patients With Hypertrophic Cardiomyopathy: JACC Review Topic of the Week. Journal of the American College of Cardiology 2018; 72(10): 1157-65.
379. Dias S, Matos C, Dias M, Pedrosa R. Paraneoplastic opsoclonus-myoclonus-ataxia syndrome in a patient with an occult thymoma. Journal of Neurology 2013; 260(SUPPL. 1): S171.
380. Diekmann R, Romagny C, Benyacoub J, et al. Influenza vaccine response in community-dwelling German prefrail and frail individuals. Immunity and Ageing 2017; 14(1): 17.
381. Dietert RR. Microbiome First Approaches to Rescue Public Health and Reduce Human Suffering. Biomedicines 2021; 9(11).
382. Dimauro I, Grazioli E, Lisi V, et al. Systemic Response of Antioxidants, Heat Shock Proteins, and Inflammatory Biomarkers to Short-Lasting Exercise Training in Healthy Male Subjects. Oxidative Medicine and Cellular Longevity 2021; 2021: 1938492.

383. Dimauro I, Grazioli E, Lisi V, et al. Endurance exercise and immune function: role of redox homeostasis and inflammatory biomarkers in systemic adaptation. Free Radical Biology and Medicine 2021; 165(Supplement 1): 31.
384. Divyashree S, Shrivastava A, Sontakke T, Venkitachalam A. Therapeutic immunosupression to treat rabies encephalitis. Open Forum Infectious Diseases 2018; 5(Supplement 1): S22.
385. Dixon N. Trash talking, respect for opponents and good competition. Sport, Ethics & Philosophy 2007; 1(1): 96-106.
386. Do A, Curran D. Clindamycin causes a maculopapular rash without eosinophilia in primary infectious mononucleosis. Journal of General Internal Medicine 2014; 29(SUPPL. 1): S334.
387. Dobreva A, Cogan NG, Paus R. Analysing the dynamics of a model for alopecia areata as an autoimmune disorder of hair follicle cycling. Mathematical medicine and biology : a journal of the IMA 2018; 35(3): 387-407.
388. Docheva D, Dex S. Running far and fast: An emerging role of tenomodulin. Journal of Orthopaedic Research 2017; 35(Supplement 1).
389. Doerr HW, Buxbaum S, Rabenau HF, Baatz H, Preiser W. Acute Retinal Necrosis Six Years after Herpes Simplex Encephalitis: An Elusive Immune Deficit Suggested by Insufficient Test Sensitivity. Journal of Medical Virology 2004; 73(2): 250-5.
390. Donovan T, Bain AL, Tu W, Pyne DB, Rao S. Influence of Exercise on Exhausted and Senescent T Cells: A Systematic Review. Front Physiol 2021; 12: 668327.
391. Dore M-F, Laaban J-P. Nutritional assessment in chronic respiratory failure. Nutrition Clinique et Metabolisme 1998; 12(4): 251-60.
392. Dorian D, Chatterjee D, Banks L, et al. A NOVEL ARRHYTHMOGENIC RIGHT VENTRICULAR CARDIOMYOPATHY (ARVC) BIOMARKER ANTI-DSG2 IS ABSENT IN ATHLETES WITH RIGHT VENTRICULAR ENLARGEMENT. Canadian Journal of Cardiology 2019; 35(10 Supplement): S2-S3.
393. Dorian D, Chatterjee D, Connelly KA, et al. A Novel Arrhythmogenic Right Ventricular Cardiomyopathy (ARVC) Biomarker-Anti-DSG2-Is Absent in Athletes With Right Ventricular Enlargement. CJC Open 2021; 3(12): 1413-8.
394. Dotzauer A, Heitmann A, Laue T, et al. The role of immunoglobulin A in prolonged and relapsing hepatitis A virus infections. J Gen Virol 2012; 93(Pt 4): 754-60.
395. Douillard C, Jannin A, Vantyghem MC. Rare causes of hypoglycemia in adults. Ann Endocrinol (Paris) 2020; 81(2-3): 110-7.
396. Drago-Serrano ME, GodÃ-nez-Victoria M, Lara-Padilla E, et al. Moderate Exercise Enhances Expression of SIgA in Mouse Ileum. International Journal of Sports Medicine 2012; 33(12): 1020-5.
397. Drexler AM. Tumor necrosis factor: its role in HIV/AIDS. STEP Perspect 1995; 7(1): 13-5.
398. Drexler H, Kutting B. Evaluation of skin-protective means against acute and chronic effects of ultraviolet radiation from sunlight. Skin Protection: Practical Applications in the Occupational Setting 2007; 34: 87-97.
399. Drks. A ketogenic approach to Rheumatoid Diseases (MIKARA). https://trialsearchwhoint/Trial2aspx?TrialID=DRKS00025413 2021.
400. Du H, Chen G, Wang S, et al. Immunological screening and characterization of highly specific monoclonal antibodies against 20 kDa hGH. Bioanalysis 2012; 4(17): 2161-8.
401. Duara R, Barker W, Loewenstein D, Bain L. The basis for disease-modifying treatments for Alzheimer's disease: the Sixth Annual Mild Cognitive Impairment Symposium. Alzheimers Dement 2009; 5(1): 66-74.
402. Dubey V. Efficacy of yoga therapy in rheumatoid arthritis patients with normal values on measures of inflammation. International Journal of Rheumatic Diseases 2016; 19(Supplement 2): 21.

403. Dudley E, Hornung F, Zheng L, Scherer D, Ballard D, Lenardo M. NF-kappaB regulates Fas/APO-1/CD95- and TCR- mediated apoptosis of T lymphocytes. Eur J Immunol 1999; 29(3): 878-86.
404. Duncan RB. Latency immunity and therapy: A clinical study of latent Epstein Barr Virus Incidence in 297 idiopathic chronic fatigue patients with plausible hypotheses. Journal of Chronic Fatigue Syndrome 1999; 5(2): 77-94.
405. Duncan WC, Rodger FE, Illingworth PJ. The human corpus luteum: Reduction in macrophages during simulated maternal recognition of pregnancy. Human Reproduction 1998; 13(9): 2435-42.
406. Eager JM, Warrender WJ, Deusenbery CB, et al. Distinct Gene Expression Profile in Patients With Poor Postoperative Outcomes After Rotator Cuff Repair: A Case-Control Study. American Journal of Sports Medicine 2021; 49(10): 2760-70.
407. Eboch C. Unplugged. Women's Sports & Fitness 1997; 19(9): 62.
408. Edirappuli SD, Venkatesh A, Zaman R. The effect of nutrition on mental health: A focus on inflammatory mechanisms. Psychiatria Danubina 2020; 32: S114-S20.
409. Edouard P, Richardson A, Murray A, et al. Ten Tips to Hurdle the Injuries and Illnesses During Major Athletics Championships: Practical Recommendations and Resources. Front Sports Act Living 2019; 1: 12.
410. Edwards KM, Burns VE, Carroll D, Drayson M, Ring C. The Acute Stress-Induced Immunoenhancement Hypothesis. Exercise & Sport Sciences Reviews 2007; 35(3): 150-5.
411. Edwards KM, Ziegler MG, Mills PJ. The potential anti-inflammatory benefits of improving physical fitness in hypertension. Journal of Hypertension 2007; 25(8): 1533-42.
412. Ehlers B, Burkhardt S, Goltz M, et al. Genetic and ultrastructural characterization of a European isolate of the fatal endotheliotropic elephant herpesvirus. J Gen Virol 2001; 82(Pt 3): 475-82.
413. Eichmann K. The immune system: cells and molecules for the integration of self and non-self. Int J Sports Med 1991; 12 Suppl 1: S2-4.
414. Eickmeyer SM, Gamble GL, Shahpar S, Do KD. The role and efficacy of exercise in persons with cancer. PM and R 2012; 4(11): 874-81.
415. Eisen HN, Chakraborty AK. Evolving concepts of specificity in immune reactions. Proceedings of the National Academy of Sciences of the United States of America 2010; 107(52): 22373-80.
416. Eisenberg A, Wong ND, Pateo C, Cecere J, Eisenberg H. Efficacy of a cardiovascular behavioral intervention program on measures of stress. Cardiology (switzerland) 2016; 134: 237-.
417. El Khoury G, Salameh P. Influenza vaccination: A cross-sectional survey of knowledge, attitude and practices among the lebanese adult population. International Journal of Environmental Research and Public Health 2015; 12(12): 15486-97.
418. El-Kader SMA. Moderate versus high intensity exercise training on leptin and selected immune system response in obese subjects. European journal of general medicine 2011; 8(4): 268-72.
419. El-Mohamady H, Francis W, Shaheen HI, et al. Detection of fecal and serum antibodies against enterotoxigenic Escherichia coli toxins and colonization factors in deployed U.S. military personnel during Operation Bright Star 2001--Egypt. The Egyptian journal of immunology / Egyptian Association of Immunologists 2006; 13(1): 189-98.
420. Elavarasi A, Goyal V, Vishnu V, Singh M, Srivastava P. Rapid onset hemidystonia-hemichorea in DYT 12: Is "SWAN NECK" limb deformity a clinical sign? Movement Disorders 2018; 33(Supplement 2): S339.
421. Elder PA, Hellemans J, Dawson T, Lewis JG. The effect of a novel deer velvet extract on human steroid metabolism. New Zealand Journal of Sports Medicine 2002; 30(3): P68-p72.
422. Eleutherakis-Papaiakovou E, Terpos E, Christoulas D, et al. Low circulating mannan-binding leptin levels correlate with increased number of febrile episodes in myeloma patients who undergo high dose melphalan with autologous hematopoietic stem cell transplantation and do not receive antibiotic prophylaxis. Blood 2011; 118(21).

423. Elgendy MH, Elsamahy SA, Mostafa M, Hamza MSK. Efficacy of shockwave therapy versus intra-articular platelet-rich plasma injection in management of knee osteoarthritis: a randomized controlled trial. International journal of pharmaceutical research 2020; 12(4): 4283-9.
424. Eliakim A, Schwindt C, Zaldivar F, Casali P, Cooper DM. Reduced tetanus antibody titers in overweight children. Autoimmunity 2006; 39(2): 137-41.
425. Ellsworth MA, Sorensen CD, White AK, Cravens RL. Efficacy of a temperature-sensitive modified-live bovine herpesvirus type-1 vaccine against abortion and stillbirth in pregnant heifers. Journal of the American Veterinary Medical Association 1996; 208(12): 2031-4.
426. Ely JTA, Krone CA. Controlling hyperglycemia as an adjunct to cancer therapy. Integrative Cancer Therapies 2005; 4(1): 25-31.
427. Engstler M, Pfohl T, Herminghaus S, et al. Hydrodynamic flow-mediated protein sorting on the cell surface of trypanosomes. Cell 2007; 131(3): 505-15.
428. Enina TN, Kuznetsov VA, Soldatova AM, et al. Relationship between levels of sex hormones and response to cardiac resynchronisation therapy in men. Kardiologiia 2018; 58(S7): 24-35.
429. Entenfellner J, Gahan J, Garvey M, Walsh C, Venner M, Cullinane A. Response of Sport Horses to Different Formulations of Equine Influenza Vaccine. Vaccines (Basel) 2020; 8(3).
430. Erdeljan M, Davidov I, Rogan D, Potkonjak A, Cutuk R. Analysis of different diagnostis methods of influenza in horses. Acta Scientiae Veterinariae 2018; 44(1): 1345.
431. Erdem S, Deniz G, Unal M. The effects of chronic aerobic and anaerobic exercises n lymphocyte subgroups. Acta Physiologica Hungarica 2005; 92(2): 163-71.
432. Erdogan B, Yimenicioglu S, Yarar C, Carman KB, Ozer E. Diagnostic effectiveness of muscle biopsy in neuromuscular diseases. Four years retrospective critical review. Acta Myologica 2018; 37(2): 156-7.
433. Eriksen EF, Lech O, Nakama GY, O'Gorman DM. Disease-Modifying Adjunctive Therapy (DMAT) in Osteoarthritis-The Biological Effects of a Multi-Mineral Complex, LithoLexal(®) Joint-A Review. Clin Pract 2021; 11(4): 901-13.
434. Esterling BA, Antoni MH, Schneiderman N, et al. Psychosocial modulation of antibody to Epstein-Barr viral capsid antigen and human herpesvirus type-6 in HIV-1-infected and at-risk gay men. Psychosomatic Medicine 1992; 54(3): 354-71.
435. Estrella D, Prata T, Pimenta G, Arreguy L, Cordeiro G. Clinical, radiological and pulmonary function of patients with hypersenbility pneumonia (HP) followed in a Brazilian Hospital. American Journal of Respiratory and Critical Care Medicine 2019; 199(9).
436. Euctr AT. A 12-Week, Double-Blind, Placebo-Controlled Trial of LY2428757 in Patients with Type 2 Diabetes Mellitus. https://trialsearchwhoint/Trial2aspx?TrialID=EUCTR2008-004207-63-AT 2008.
437. Euctr CZ. Evaluation of the Effect of Fostamatinib Dosed at 100mg twice a Day on Blood Pressure when Walking, Moving Around and Living Normal Daily Life in Patients with Rheumatoid Arthritis. https://trialsearchwhoint/Trial2aspx?TrialID=EUCTR2011-006070-73-CZ 2012.
438. Euctr DE. A Two-Stage Randomized Placebo-controlled Ascending Dose Phase I/ IIa Study to Evaluate Safety, Tolerability, Pharmacodynamic Effects and Preliminary Efficacy of an Anti-Interleukin 1 beta Vaccine (CYT013-IL1bQb) in Patients with Type 2 Diabetes Mellitus. https://trialsearchwhoint/Trial2aspx?TrialID=EUCTR2008-007012-15-DE 2009.
439. Euctr DE. Efficacy of everolimus on top to centre specific standard immunosuppressive regimen in lung transplant recipients. https://trialsearchwhoint/Trial2aspx?TrialID=EUCTR2011-001539-21-DE 2011.
440. Euctr DE. GO-AHEAD. https://trialsearchwhoint/Trial2aspx?TrialID=EUCTR2011-000311-34-DE 2011.
441. Euctr ES. Multicenter randomized, double-blind, placebo-controlled parallel clinical trial to assess efficacy and safety of Omalizumab (Xolair®) in a new indication: cholinergic urticaria. https://trialsearchwhoint/Trial2aspx?TrialID=EUCTR2013-002770-43-ES 2013.

442. Euctr SE. Clinical research study to evaluate safety and efficacy of alpha1-proteinase inhibitor (A1-PI) compared to placebo (a product that does not contain the active ingredient) administered intravenously to patients with deficiency of alpha1-proteinase inhibitor and emphysema. https://trialsearchwhoint/Trial2aspx?TrialID=EUCTR2005-003459-12-SE 2007.
443. Faccin Borges G, Teixeira AM, Pedro Ferreira J. Meta-análise do efeito no sistema imunitário da suplementação de hidratos de carbono no exercício físico. / Meta-analysis of the effect on immune system of carbohydrate supplementation on exercise. Motricidade 2012; 8(3): 83-97.
444. Fakhraee F, Badiee A, Alavizadeh SH, et al. Coadminstration of L. major amastigote class I nuclease (rLmaCIN) with LPD nanoparticles delays the progression of skin lesion and the L. major dissemination to the spleen in BALB/c mice-based experimental setting. Acta Trop 2016; 159: 211-8.
445. Falcini F, Lepri G, Bertini F, Matucci Crinic M, Tarantino G, Rigante D. Clinical overview of a cohort of 87 italian patients with pediatric autoimmune neuropsychiatric disorder associated with streptococcus infection (PANDAS). Annals of the Rheumatic Diseases 2014; 73(SUPPL. 2).
446. Falcini F, Lepri G, Bertini F, Rigante D, Matucci Cerinic M. PReS-FINAL-2252: Descriptive analysis of pediatric autoimmune neuropsychiatric disorder associated with streptococcus infection (PANDAS) in a cohort of 65 Italian patients. Pediatric Rheumatology 2013; 11(SUPPL. 2).
447. Fali T, Fabre-Mersseman V, Yamamoto T, et al. Elderly human hematopoietic progenitor cells express cellular senescence markers and are more susceptible to pyroptosis. JCI Insight 2018; 3(13).
448. Falkowitz D, Lee D, Nogar J. Fatality secondary to nivolumab induced hepatitis: A medication error. Clinical Toxicology 2017; 55(7): 724.
449. Fan C, Huang S, Xiang C, An T, Song Y. Identification of key genes and immune infiltration modulated by CPAP in obstructive sleep apnea by integrated bioinformatics analysis. PLoS One 2021; 16(9): e0255708.
450. Farrell K, Issekutz T, Derfalvi B, Brager R. Variable spectrum of organ involvement in siblings with LRBA deficiency. LymphoSign Journal 2019; 6(4): 148-51.
451. Farsani ME, Rube J, Sriwastava SK, Bernitsas E. A Unique Case of a Patient with Tuberous Sclerosis and Recent Diagnosis of Neuromyelitis Optica...2020 Virtual Annual Meeting of the Consortium of Multiple Sclerosis Centers, May 26-29, 2020. International Journal of MS Care 2020; 22(S2): 84-.
452. Feldman C, Klugman K. Adult pneumococcal vaccination guideline. South African Medical Journal 1999; 89(11 II): 1222-30.
453. Ferry A, Renault F, Rieu M. Effet de l'exercice epuisant et du dehydroepiandrosterone (DHEA) sur la production d'anticorps specifiques in vivo chez le rat. / Effect of physical exercise until exhaustion and DHEA on in vovo antibody production in rats. Science & Sports 1998; 13(1): 34-6.
454. Fiala O, Šorejs O, Šustr J, Fínek J. Side Effects and Efficacy of Immunotherapy. Klin Onkol 2020; 33(1): 8-10.
455. Field T. Yoga research review. Complementary therapies in clinical practice 2016; 24: 145-61.
456. Filonenko K, Gavrylov A, Pobedonna T. Specific immunotherapy as a method of asthma control achievement. European Journal of Medical Research 2010; 15(SUPPL. 1): 208.
457. Fink PC, Schedel I, Peter HH, Deicher H. Inhibitory action of antiglobulin preparations and rheumatoid arthritis sera on cytotoxicity of human lymphocytes in vitro. Verhandlungen der Deutschen Gesellschaft fur Rheumatologie 1978; VOL. 5: 130.
458. Fischetto G, Bermon S. From gene engineering to gene modulation and manipulation: can we prevent or detect gene doping in sports? Sports medicine (Auckland, NZ) 2013; 43(10): 965-77.
459. Fisher ES, Ruha M. Neurotoxicity from north American rattlesnake envenomation reversed by crotalidae immune F(ab')2. Journal of Medical Toxicology 2020; 16(2): 159-60.

460. Fistetto G, Iannitti T, Capone S, Torricelli F, Palmieri B. Deep Oscillation: Terapeuthic-rehabilitative experiences with a new electrostatic device. Minerva Medica 2011; 102(4): 277-88.
461. Fiuza-Luces C, Padilla JR, Valentín J, et al. Effects of Exercise on the Immune Function of Pediatric Patients With Solid Tumors: Insights From the PAPEC Randomized Trial. American Journal of Physical Medicine & Rehabilitation 2017; 96(11): 831-7.
462. Fiuza-Luces C, Valenzuela PL, Castillo-Garcia A, Lucia A. Exercise Benefits Meet Cancer Immunosurveillance: Implications for Immunotherapy. Trends in Cancer 2021; 7(2): 91-3.
463. Fleming DM, Crovari P, Wahn U, et al. Comparison of the efficacy and safety of live attenuated cold-adapted influenza vaccine, trivalent, with trivalent inactivated influenza virus vaccine in children and adolescents with asthma. Pediatric infectious disease journal 2006; 25(10): 860-9.
464. Fletcher MP. Biosimilars clinical development program: confirmatory clinical trials: a virtual/simulated case study comparing equivalence and non-inferiority approaches. Biologicals 2011; 39(5): 270-7.
465. Flint-Bretler O, Shochat T, Tzischinsky O. The effects of a parental intervention on electronic media exposure and sleep patterns in adolescents. Sleep Medicine 2013; 14(SUPPL. 1): e126-e7.
466. Floss DM, Mockey M, Zanello G, et al. Expression and immunogenicity of the mycobacterial Ag85B/ESAT-6 antigens produced in transgenic plants by elastin-like peptide fusion strategy. Journal of Biomedicine & Biotechnology 2010: 274346-.
467. Flower G. CANCER AND IMMUNITY: TRADITION MEETS INNOVATION. Alive: Canada's Natural Health & Wellness Magazine 2021; (462): 13-6.
468. Fodor M, Primavesi F, Morell-Hofert D, et al. Non-operative management of blunt hepatic and splenic injuries-practical aspects and value of radiological scoring systems. Eur Surg 2018; 50(6): 285-98.
469. Fomina E, Uskov K, Rykova M, et al. Adaptive immunity as an indicator of optimum physical loads during 520-day isolation. Human Physiology 2017; 43(3): 301-11.
470. Foxall RB, Albuquerque AA, Baptista AP, et al. Frequency of cycling regulatory T cells intwo aids-associated diseases with distinct clinical outcomes: HIV-1 and HIV-2 infections. European Journal of Immunology 2009; 39(SUPPL. 1): S504.
471. Fraile-Ramos A, Kledal TN, Pelchen-Matthews A, Bowers K, Schwartz TW, Marsh M. The human cytomegalovirus US28 protein is located in endocytic vesicles and undergoes constitutive endocytosis and recycling. Molecular Biology of the Cell 2001; 12(6): 1737-49.
472. Francis ME, McNeil M, Dawe NJ, et al. Historical H1N1 Influenza Virus Imprinting Increases Vaccine Protection by Influencing the Activity and Sustained Production of Antibodies Elicited at Vaccination in Ferrets. Vaccines (Basel) 2019; 7(4).
473. Francis O, Su R, Martinez S, et al. A human-mouse xenograft model to evaluate therapies and study the role of TSLP-induced signals in Ph-like ALL. Cancer Research 2014; 74(20 SUPPL. 1).
474. Francis-Oliviero F, Bozoki S, Micsik A, Kieny MP, Lelievre J-D. Research priorities to increase vaccination coverage in Europe (EU joint action on vaccination). Vaccine 2021; 39(44): 6539-44.
475. Franek J. Home telehealth for patients with chronic obstructive pulmonary disease (COPD): an evidence-based analysis. Ont Health Technol Assess Ser 2012; 12(11): 1-58.
476. Freeman ML, Moisi D, Clagett B, Rodriguez B, Lederman MM, Calabrese L. Methotrexate blocks proliferation not inflammation to modulate immunity. Topics in Antiviral Medicine 2020; 28(1): 97.
477. Freitas C, Nguyen J, Rondeau V, et al. Efficient lymphoid differentiation of hematopoietic stem cells requires CXCR4 desensitization. Haematologica 2017; 102(Supplement 2): 453.
478. Friedenreich CM, Orenstein MR. Physical activity and cancer prevention: Etiologic evidence and biological mechanisms. Journal of Nutrition 2002; 132(11 SUPPL.): 3456S-64S.

479. Fujimura T, Mukuno A, Sato N, et al. Reduced expression of dermicidin, a peptide active against Propionibacterum acnes, in the sweat of acne vulgaris patients. Journal of Investigative Dermatology 2010; 130(SUPPL. 1): S123.
480. Fukagawa NK. Protein and amino acid supplementation in older humans. Amino Acids 2013; 44(6): 1493-509.
481. Fukagawa NK. Amino acid metabolism in aging. Amino Acids 2013; 45(3): 568.
482. Furci F, Ricciardi L. Sublingual immunotherapy with 5-grass pollen extract tablets: Can immunization to cross-reactive non-specific lipid-transfer proteins (NsLTPs) occur? Allergy: European Journal of Allergy and Clinical Immunology 2019; 74(Supplement 106): 641-2.
483. Galama JM. [National hepatitis B vaccination closer to implementation, but not soon enough; recommendations from the Dutch Health Council]. Ned Tijdschr Geneeskd 2001; 145(28): 1339-42.
484. Galama JMD. Universal hepatitis b vaccination closer to implementation, but not close enough: Recommendations from the Dutch health council. Nederlands Tijdschrift voor Geneeskunde 2001; 145(28): 1339-42.
485. Galea MP, Dunlop SA, Marshall R, Clark J, Churilov L. Early exercise after spinal cord injury ('Switch-On'): Study protocol for a randomised controlled trial. Trials 2015; 16(1): 7.
486. Gallard J, Franques J, Verschueren A, et al. Acute lower motor neurone paraneoplastic disease associated with neuronal autoantibodies: Report of two cases. Journal of the Peripheral Nervous System 2012; 17(2): 240.
487. Galvao D. Exercise as a synergistic medicine for prostate cancer. Asia-pacific journal of clinical oncology 2016; 12: 30-1.
488. Galvao DA, Hart N, Newton RU, Taaffe D, Spry N. Endogenous exercise medicine: Mechanisms influencing prostate cancer biology. BJU International 2016; 118(Supplement 1): 10.
489. Gammie AJ, Wyn-Jones AP. Does hepatitis A pose a significant health risk to recreational water users? Water Science and Technology 1997; 35(11-12): 171-7.
490. Ganavi YP, Selvan C. Nivolumab, an immune check point inhibitor induced thyroid dysfunction. Indian Journal of Endocrinology and Metabolism 2018; 22(7 Supplement 1): S48.
491. Gandini J, Manto M, Bremova-Ertl T, Feil K, Strupp M. The neurological update: therapies for cerebellar ataxias in 2020. Journal of neurology 2020.
492. Gao X, Yin J. Genetic variation at IL-18 gene is associated with wheat-dependent exercise-induced anaphylaxis in Chinese Han population. Allergy: European Journal of Allergy and Clinical Immunology 2018; 73(Supplement 105): 36.
493. Garcia JJ, Bote E, Hinchado MD, Ortega E. A single session of intense exercise improves the inflammatory response in healthy sedentary women. Journal of Physiology and Biochemistry 2011; 67(1): 87-94.
494. García-Arévalo C, Bermejo-Martín JF, Rico L, et al. Immunomodulatory nanoparticles from elastin-like recombinamers: single-molecules for tuberculosis vaccine development. Mol Pharm 2013; 10(2): 586-97.
495. Gautier B, Berthelot J, Deck M, et al. AAV2/9-mediated silencing of PMP22 prevents the development of pathological features in a rat model of Charcot-Marie-Tooth disease 1 A. Nature Communications 2021; 12(1): 2356.
496. Gautret P, Hoang V-T. Infectious Diseases and Mass Gatherings. Current Infectious Disease Reports 2018; 20(11): 44.
497. Gelberg J, McIvor RA. Overcoming gaps in the management of chronic obstructive pulmonary disease in older patients: New insights. Drugs and Aging 2010; 27(5): 367-75.
498. Gencturk M, Yuksel M, Eren O. Evaluation study for self management of patients with ankylosing spondylitis (AS) through a personal health system. Annals of the Rheumatic Diseases 2015; 74(SUPPL. 2): 550-1.
499. Gene RJ, Giugno ER, Abbate EH, et al. Updated Argentine Consensus on Chronic Obstructive Pulmonary Disease. Medicina 2003; 63(5): 419-46.

500. Geng L, Xu X, Zhang H, et al. Comprehensive expression profile of long non-coding RNAs in Peripheral blood mononuclear cells from patients with neuropsychiatric systemic lupus erythematosus. Annals of Translational Medicine 2020; 8(6): 349.
501. Gentile A, Musella A, De Vito F, et al. Immunomodulatory Effects of Exercise in Experimental Multiple Sclerosis. Front Immunol 2019; 10: 2197.
502. George LA, Sullivan SK, Giermasz A, et al. Spk-9001: Adeno-associated virus mediated gene transfer for hemophilia B achieves sustained mean factor IX activity levels of >30% without immunosuppression. Blood 2016; 128(22).
503. George ST, Ma J, Lai J, Stacey HD, Miller MS, Mullarkey CE. Neutrophils and influenza: A thin line between helpful and harmful. Vaccines 2021; 9(6): 597.
504. Gerard C. Blood receivers: Another point of view-analysis of data from 5,463 patients transfused during 2011. Blood Transfusion 2013; 11(SUPPL. 1): s13-s4.
505. Gerardo CJ, Quackenbush E, Lewis B, et al. The Efficacy of Crotalidae Polyvalent Immune Fab (Ovine) Antivenom Versus Placebo Plus Optional Rescue Therapy on Recovery From Copperhead Snake Envenomation: A Randomized, Double-Blind, Placebo-Controlled, Clinical Trial. Annals of Emergency Medicine 2017; 70(2): 233-44.e3.
506. Ghanemi A, Yoshioka M, St-Amand J. Impact of Adiposity and Fat Distribution, Rather Than Obesity, on Antibodies as an Illustration of Weight-Loss-Independent Exercise Benefits. Medicines (Basel) 2021; 8(10).
507. Ghayomzadeh M, Asadollahi-Amin A, Seyedalinaghi S, Mohraz M, Gharakhanlou R. Effects of resistance training and lifestyle modification on tcd4+cell count and body composition of hiv+ patients. Journal of Mazandaran University of Medical Sciences 2019; 29(173): 40-9.
508. Ghofrani HA, Friese G, Discher T, et al. Inhaled iloprost is a potent acute pulmonary vasodilator in HIV-related severe pulmonary hypertension. European Respiratory Journal 2004; 23(2): 321-6.
509. Giglio D, Berntsson H, Fred A, Ny L. Immune Checkpoint Inhibitor-Induced Polymyositis and Myasthenia Gravis with Fatal Outcome. Case Reports in Oncology 2020; 13(3): 1252-7.
510. Giles EL, Robalino S, Sniehotta FF, Adams J, McColl E. The effectiveness of financial incentives for health behaviour change: Systematic review and meta-analysis. PLoS ONE 2014; 9(3): e90347.
511. Gillard G, Proctor J, Hyzy S, et al. A novel targeted approach to achieve immune system reset: CD45-targeted antibody drug conjugates ameliorate disease in preclinical autoimmune disease models and enable auto-hsct. Annals of the Rheumatic Diseases 2020; 79(SUPPL 1): 190-1.
512. Giménez E, de Bolós C, Belalcazar V, et al. Anti-EPO and anti-NESP antibodies raised against synthetic peptides that reproduce the minimal amino acid sequence differences between EPO and NESP. Anal Bioanal Chem 2007; 388(7): 1531-8.
513. Ginsburg O. Government and civil society efforts in global cancer control. Cancer Research 2018; 78(13 Supplement 1).
514. Giron F, Pizano A, Calvachi P, Cordovez JM. A mathematical model for the population dynamics of intestinal microbiota: obese versus healthy gastrointestinal tract. FASEB Journal 2017; 31(1 Supplement 1).
515. Girouard N, Soucy N. Patient considerations in the management of multiple sclerosis: Development and clinical utility of oral agents. Patient Preference and Adherence 2011; 5: 101-8.
516. Gizinger OA, Khisamova AA. Curcumin in the correction of oxidative and immune disorders during exercises. Voprosy pitaniia 2021; 90(1): 65-73.
517. Glaser R, Padgett DA, Litsky ML, et al. Stress-associated changes in the steady-state expression of latent Epstein-Barr virus: implications for chronic fatigue syndrome and cancer. Brain Behav Immun 2005; 19(2): 91-103.

518. Glass OK, Inman BA, Broadwater G, et al. Effect of aerobic training on the host systemic milieu in patients with solid tumours: An exploratory correlative study. British Journal of Cancer 2015; 112(5): 825-31.
519. Gleeson M. Dosing and efficacy of glutamine supplementation in human exercise and sport training. Journal of Nutrition 2008; 138(10): 2045S-9S.
520. Gleeson M, Pyne DB. Exercise effects on mucosal immunity. Immunology and Cell Biology 2000; 78(5): 536-44.
521. Gleeson M, Pyne DB. Special feature for the Olympics: effects of exercise on the immune system: exercise effects on mucosal immunity. Immunol Cell Biol 2000; 78(5): 536-44.
522. Gleeson M, Pyne DB, Austin JP, et al. Epstein-Barr virus reactivation and upper-respiratory illness in elite swimmers. Med Sci Sports Exerc 2002; 34(3): 411-7.
523. Gleeson M, Pyne DB, Callister R. The missing links in exercise effects on mucosal immunity. Exercise immunology review 2004; 10: 107-28.
524. Gluchowski A, Harris N, Dulson D, Cronin J. Chronic Eccentric Exercise and the Older Adult. Sports medicine (Auckland, NZ) 2015; 45(10): 1413-30.
525. Goddard PJ, Fawcett AR, Macdonald AJ, Reid HW. The behavioural, physiological and immunological responses of lambs from two rearing systems and two genotypes to exposure to humans. Appl Anim Behav Sci 2000; 66(4): 305-21.
526. Goebel WF, Avery OT. CHEMO-IMMUNOLOGICAL STUDIES ON CONJUGATED CARBOHYDRATE-PROTEINS : I. THE SYNTHESIS OFp-AMINOPHENOL beta-GLUCOSIDE, p-AMINOPHENOL beta-GALACTOSIDE, AND THEIR COUPLING WITH SERUM GLOBULIN. J Exp Med 1929; 50(4): 521-31.
527. Goebel-Stengel M, Wang L. Central and peripheral expression and distribution of NUCB2/nesfatin-1. Curr Pharm Des 2013; 19(39): 6935-40.
528. Goh GK, Dunker AK, Uversky VN. Protein intrinsic disorder toolbox for comparative analysis of viral proteins. BMC Genomics 2008; 9 Suppl 2(Suppl 2): S4.
529. Goldstein DB, Need AC, Singh R, Sisodiya SM. Potential Genetic Causes of Heterogeneity of Treatment Effects. American Journal of Medicine 2007; 120(4 SUPPL.): S21-S5.
530. Gomes AL, Delgado-Alves J, Jury EC. High density lipoproteins stabilize antigen presenting cell:T cell conjugates by altering the T cell receptor signalling kinetics. Arthritis and Rheumatism 2011; 63(10 SUPPL. 1).
531. Gomez JA, Lepetic A, Demarteau N. Health economic analysis of human papillomavirus vaccines in women of Chile: perspective of the health care payer using a Markov model. BMC Public Health 2014; 14(1): 1-25.
532. Gonthier D, Basselin P, Boivin JM, Kivits J, Pulcini C. Postponing vaccination in children with an infection: a qualitative study among general practitioners and pediatricians. Fam Pract 2020; 37(4): 541-6.
533. Gooch S. Review: nursing care driven by guidelines improves some process measures and patient outcomes [commentary on Thomas L, Cullum N, McColl E, et al. Clinical guidelines in nursing, midwifery and other professions allied to medicine. (Cochrane Review, latest version 24 Nov 1998) In: Cochrane Library. Oxford: Update Software and Thomas LH, McColl E, Cullum N, et al. Effect of clinical guidelines in nursing, midwifery, and the therapies: a systematic review of evaluations. QUAL HEALTH CARE 1998 Dec;7(4):183-91]. Evidence Based Nursing 1999: 87-.
534. Good-Jacobson K, Di Pietro A, Tempany J, Tarlinton D. Regulation of plasma cell differentiation, migration and class-switch by c-Myb and T-bet. Journal of Immunology 2016; 196(SUPPL. 1).
535. Goodman MF. Better living with hyper-mutation. Environmental and Molecular Mutagenesis 2016.
536. Goodman SN, Sladky JT. A Bayesian approach to randomized controlled trials in children utilizing information from adults: The case of Guillain-Barre syndrome. Clinical Trials 2005; 2(4): 305-10.

537. Goodyear LJ, Boppart MD, Fielding RA, Asp S, Wojtaszewski JFP, Mohr T. Marathon running transiently increases c-Jun NH2-terminal kinase and p38gamma activities in human skeletal muscle. Journal of Physiology 2000; 526(3): 663-9.
538. Gordon GS, Hurliman M, Stinchcomb DT, et al. Effect of formulation ratios and dosing schedules on the safety and immunogenicity of a recombinant live attenuated tetravalent dengue vaccine (DENVax) in healthy adult volunteers. American Journal of Tropical Medicine and Hygiene 2013; 89(5 SUPPL. 1): 320.
539. Govaere O, Palmer JM, Clark JE, et al. Macrophage Scavenger Receptor 1 mediates lipid-induced inflammation in non-alcoholic fatty liver disease. Journal of hepatology 2021.
540. Grabs V, Kersten A, Haller B, et al. Rutoside and Hydrolytic Enzymes Do Not Attenuate Marathon-Induced Inflammation. Medicine & Science in Sports & Exercise 2017; 49(3): 387-95.
541. Grabs V, Nieman DC, Haller B, Halle M, Scherr J. The effects of oral hydrolytic enzymes and flavonoids on inflammatory markers and coagulation after marathon running: study protocol for a randomized, double-blind, placebo-controlled trial. BMC sports science, medicine and rehabilitation 2014; 6(1).
542. Grafetstätter C, Gaisberger M, Prossegger J, et al. Does waterfall aerosol influence mucosal immunity and chronic stress? A randomized controlled clinical trial. J Physiol Anthropol 2017; 36(1): 10.
543. Grais RF, Juan-Giner A. Vaccination in humanitarian crises: satisficing should no longer suffice. Int Health 2014; 6(3): 160-1.
544. Gray AB, Telford RD, Collins M, Baker MS, Weidemann MJ. Granulocyte activation induced by intense interval running. J Leukoc Biol 1993; 53(5): 591-7.
545. Grebenciucova E, Tavee J. A triad of cmt1a hereditary neuropathy, anti-neurofascin-155 igg antibodies and relapsing remitting multiple sclerosis. Neurology 2019; 92(15 Supplement 1).
546. Greco SJ, Bryan M, Pliner LF, Rameshwar P, Patel SA, Banerjee D. AMD3100-mediated production of interleukin-1 from mesenchymal stem cells is key to chemosensitivity of breast cancer cells. American Journal of Cancer Research 2011; 1(6): 701-15.
547. Greeson J, Chin G, Nardi W, Everson-Rose SA, Llabre M. Mindfulness and cardiovascular health: Outcomes, mechanisms & individual differences. Psychosomatic Medicine 2019; 81(4): A166-A7.
548. Grenda A, Krawczyk P. New Dancing Couple: PD-L1 and MicroRNA. Scandinavian Journal of Immunology 2017; 86(3): 130-4.
549. Griese M. Chronic interstitial lung diseases in children: What's new? Pediatric Pulmonology 2014; 49(SUPPL. 37): S21-S3.
550. Griffith RJ, Alsweiler J, Moore AE, et al. Interventions to prevent women from developing gestational diabetes mellitus: an overview of Cochrane Reviews. Cochrane Database Syst Rev 2020; 6(6): Cd012394.
551. Gross N, Levin D. Primary Care of the Patient with Chronic Obstructive Pulmonary Disease-Part 2: Pharmacologic Treatment Across All Stages of Disease. American Journal of Medicine 2008; 121(7 SUPPL. 1): S13-S24.
552. Grubeck-Loebenstein B, Della Bella S, Iorio AM, Michel J, Pawelec G, Solana R. Immunosenescence and vaccine failure in the elderly. Aging Clinical & Experimental Research 2009; 21(3): 201-9.
553. Grudzinska FS, Brodlie M, Scholefield BR, et al. Neutrophils in community-acquired pneumonia: parallels in dysfunction at the extremes of age. Thorax 2020; 75(2): 164-71.
554. Gubio AB, Mamman AI, Abdul M, Olayinka AT. The risk factors of exposure to rubella among pregnant women in Zaria 2013. The Pan African medical journal 2019; 32(Supplement 1): 4.
555. Gueldner SH, Poon LW, Bramlett MH, et al. Long term exercise patterns and immune function in healthy older women. A report of preliminary findings. Mechanisms of Ageing and Development 1997; 93(1-3): 215-22.

556. Guimond M, Fry TJ, Mackall CL. Cytokine signals in T-cell homeostasis. J Immunother 2005; 28(4): 289-94.
557. Guirguis-Blake JM, Senger CA, Webber EM, Mularski R, Whitlock EP. U.S. Preventive Services Task Force Evidence Syntheses, formerly Systematic Evidence Reviews. Screening for Chronic Obstructive Pulmonary Disease: A Systematic Evidence Review for the US Preventive Services Task Force. Rockville (MD): Agency for Healthcare Research and Quality (US); 2016.
558. Gunzer W, Konrad M, Pail E. Exercise-induced immunodepression in endurance athletes and nutritional intervention with carbohydrate, protein and fat-what is possible, what is not? Nutrients 2012; 4(9): 1187-212.
559. Guo S, Huang Y, Zhang Y, Hong S, Huang H, Liu T. Impacts of exercise interventions on different diseases and organ functions in mice. Journal of Sport and Health Science 2020; 9(1): 53-73.
560. Guo Y, Nakamura S, Ando T, Yoneyama H, Kudo S, Isogai E. The inhibition effect of antiserum on the motility of Leptospira. Curr Microbiol 2013; 66(4): 359-64.
561. Gupta S, Shukla T, Khwaja GA. Chronic hepatitis B related CNS demyelination with cerebellar ataxia responsive to steroids - A case report. Annals of Indian Academy of Neurology 2019; 22(SUPPL 1): S138.
562. Gustafson M, Wheatley-Guy C, Gastineau D, et al. Physical fitness influences the composition of human T cell populations. Journal for ImmunoTherapy of Cancer 2019; 7(Supplement 1).
563. Gyure K. West nile virus infection-associated immune reconstitution inflammatory syndrome of the central nervous system. Journal of Neuropathology and Experimental Neurology 2015; 74(6): 628.
564. Haan MN, Pierce CM, Simanek AM, Liang J, Aiello AE. Persistent infection, inflammation, and functional impairment in older latinos. Journals of Gerontology - Series A Biological Sciences and Medical Sciences 2008; 63(6): 610-8.
565. Habers GE, Bos GJ, van Royen-Kerkhof A, et al. Muscles in motion: a randomized controlled trial on the feasibility, safety and efficacy of an exercise training programme in children and adolescents with juvenile dermatomyositis. Rheumatology (Oxford, England) 2016; 55(7): 1251-62.
566. Habeshaw JA, Dalgleish AG, Bountiff L, et al. AIDS pathogenesis: HIV envelope and its interaction with cell proteins. Immunology Today 1990; 11(11): 418-25.
567. Hadrabova M, Janikova A, Stastna J, et al. The importance of physical activity in adult lymphoma survivors-single center's experience with the supervised aerobic and resistance training program. Hematological Oncology 2017; 35(Supplement 2): 307.
568. Hagar A, Wang Z, Koyama S, et al. Endurance training slows breast tumor growth in mice by suppressing Treg cells recruitment to tumors. BMC Cancer 2019; 19(1): N.PAG-N.PAG.
569. Hagen K. Risk of infections among orienteers. Tidsskrift for den Norske laegeforening : tidsskrift for praktisk medicin, ny raekke 2009; 129(13): 1326-8.
570. Hagstrom AD, Marshall PW, Lonsdale C, et al. The effect of resistance training on markers of immune function and inflammation in previously sedentary women recovering from breast cancer: a randomized controlled trial. Breast cancer research and treatment 2016; 155(3): 471-82.
571. Hahne SJM, de Melker HE, Sanders EAM, Knol MJ, Monge S, van der Ende A. Effectiveness of the DTPa-HBV-IPV/Hib vaccine against invasive Haemophilus influenzae type b disease in the Netherlands (2003-16): a case-control study. The Lancet Infectious Diseases 2018; 18(7): 749-57.
572. Halloran PF. Molecular mechanisms of new immunosuppressants. Clin Transplant 1996; 10(1 Pt 2): 118-23.
573. Hama Y, Mori-Yoshimura M, Komaki H, et al. [Childhood-onset anti-3-hydroxy-3-methylglutaryl-coenzyme A reductase (anti-HMGCR) necrotizing myopathy needs to be distinguished from muscular dystrophy: A case study]. Rinsho Shinkeigaku 2017; 57(10): 567-72.
574. Hamilton RG, Adkinson NF, Jr. Mechanisms of acute allergic reactions. Artif Organs 1984; 8(3): 311-7.

575. Hammond NL, El Chami C, Elias A, O'Neill CA. 650 Topical application of a bacterial lysate modulates changes to the fibrillin-rich microfibril network. Journal of Investigative Dermatology 2019; 139(9 Supplement): S326.
576. Han H, Attur M, Abramson SB, Inman JK, Fattah RJ, Mongini PKA. APRIL and BAFF promote increased viability of replicating human B2 cells via mechanism involving cyclooxygenase 2. Journal of Immunology 2006; 176(11): 6736-51.
577. Han LN, Liu HB, Luo LM, et al. Th17-related cytokine gene polymorphisms with heart failure. European Heart Journal 2017; 38(Supplement 1): 908.
578. Hanckel B, Petticrew M, Thomas J, Green J. The use of Qualitative Comparative Analysis (QCA) to address causality in complex systems: a systematic review of research on public health interventions. BMC public health 2021; 21(1): 877.
579. Hansen MP, Hoffmann TC, McCullough AR, van Driel ML, Del Mar CB. Antibiotic Resistance: What are the Opportunities for Primary Care in Alleviating the Crisis? Front Public Health 2015; 3: 35.
580. Hanson PG, Flaherty DK. Immunological responses to training in conditioned runners. Clin Sci (Lond) 1981; 60(2): 225-8.
581. Harari D, Iliffe S, Kharicha K, et al. Promotion of health in older people: A randomised controlled trial of health risk appraisal in British general practice. Age and Ageing 2008; 37(5): 565-71.
582. Hare JM, Fishman JE, Gerstenblith G, et al. Comparison of allogeneic vs autologous bone marrow-derived mesenchymal stem cells delivered by transendocardial injection in patients with ischemic cardiomyopathy: the POSEIDON randomized trial. JAMA - journal of the american medical association 2012; 308(22): 2369-79.
583. Hartley A, Caga-Anan M, Haskard D, et al. The interaction of exercise and percutaneous coronary intervention with dynamic levels of oxidized LDL and anti-oxidized LDL antibodies: A secondary analysis of the ORBITA study. Atherosclerosis 2020; 315: e147.
584. Hartley A, Shun-Shin M, Caga-Anan M, et al. The Placebo-Controlled Effect of Percutaneous Coronary Intervention on Exercise Induced Changes in Anti-Malondialdehyde-LDL Antibody Levels in Stable Coronary Artery Disease: A Substudy of the ORBITA Trial. Front Cardiovasc Med 2021; 8: 757030.
585. Hassali MAA, Harun SN, Hussain R, et al. Pharmacological and non-pharmacological management of COPD; limitations and future prospects: a review of current literature. Journal of Public Health (Germany) 2020; 28(4): 357-66.
586. Hatcher S. Review: cognitive behavioural therapy and graded exercise show the most promise for chronic fatigue syndrome. Evidence Based Mental Health 2002: 54-.
587. Hayday TS, Laing AG, Del Molino Del Barrio I, Hayday AC, Di Rosa F. COVID-19: Using high-throughput flow cytometry to dissect clinical heterogeneity. Cytometry Part A : the journal of the International Society for Analytical Cytology 2021.
588. Hayes J, Schuster M, Grossman F, Rutman O, Itescu S. Mesenchymal stem cell therapy improves pulmonary function and exercise tolerance in patients with chronic obstructive pulmonary disease (copd) and high baseline inflammation. Cytotherapy 2020; 22(5 Supplement): S188-S9.
589. Hayrinen-Immonen R. Immune-activation in recurrent oral ulcers (ROU). Scandinavian journal of dental research 1992; 100(4): 222-7.
590. Haywood BA, Black KE, Baker D, McGarvey J, Healey P, Brown RC. Probiotic supplementation reduces the duration and incidence of infections but not severity in elite rugby union players. Journal of Science & Medicine in Sport 2014; 17(4): 356-60.
591. He TY, Zhang N, Xia Y, Luo Y, Li CR, Yang J. [Short stature, optic nerve atrophy and Pelger-Huët anomaly syndrome with antibody immunodeficiency and aplastic anemia: a case report and literature review]. Zhonghua Er Ke Za Zhi 2017; 55(12): 942-6.
592. Heaney JL, Phillips AC, Drayson MT, Campbell JP. Serum free light chains are reduced in endurance trained older adults: Evidence that exercise training may reduce basal inflammation in older adults. Exp Gerontol 2016; 77: 69-75.

593. Heidaryani L, Keramatimoghadam M, Keramatimoghadam M. PSYCHOLOGICAL CONSEQUENCES OF CORONAVIRUS: SUGGESTED SOLUTIONS FOR ATHLETES. Sport Scientific & Practical Aspects 2021; 18(1): 5-10.
594. Heine M, van de Port I, Rietberg MB, van Wegen EE, Kwakkel G. Exercise therapy for fatigue in multiple sclerosis. Cochrane Database Syst Rev 2015; (9): Cd009956.
595. Heinemann NC, Tischer-Zimmermann S, Heuft H-G, et al. High-intensity interval training in allogeneic adoptive T-cell immunotherapy - A big HIT? Journal of Translational Medicine 2020; 18(1): 148.
596. Hendrix S, Ellison N, Stanworth S, et al. Methodological Aspects of the Phase II Study AFF006 Evaluating Amyloid-beta -Targeting Vaccine AFFITOPE® AD02 in Early Alzheimer's Disease - Prospective Use of Novel Composite Scales. J Prev Alzheimers Dis 2015; 2(2): 91-102.
597. Henrich TJ, Hsue P, Deeks SG, et al. Sirolimus reduces T-cell cycling and immune checkpoint marker expression, ACTG A5337. Topics in Antiviral Medicine 2019; 27(SUPPL 1): 49s.
598. Henry RK, Chaudhari M. Hyperthyrotropinemia : A presentation of secondary adrenal insufficiency. Hormone Research in Paediatrics 2017; 88(Supplement 1): 74.
599. Henson D, Nieman D, Davis JM, et al. Post-i 60-km Race Illness Rates and Decreases in Granulocyte Respiratory Burst and Salivary IgA Output are Not Countered by Quercetin Ingestion. International Journal of Sports Medicine 2008; 29(10): 956-63.
600. Herth FJ, Eberhardt R, Gompelmann D, Criner GJ, Ernst A, Valipour A. The value of pre trial rehabilitation before endoscopic lung volume reduction-analysis of the vent trial. American Journal of Respiratory and Critical Care Medicine 2014; 189(MeetingAbstracts).
601. Hertl MC, Strasberg SR, Mackinnon SE, et al. The dose-related effect of monoclonal antibodies against adhesion molecules ICAM-1 and LFA-1 on peripheral nerve allograft rejection in a rat model. Restor Neurol Neurosci 1996; 10(3): 147-59.
602. Hey-Cunningham AJ, Markham R, Fraser IS, Berbic M, Fazleabas AT, Braundmeier AG. Endometrial and endometriotic lesion immune cell populations in a baboon model of endometriosis. Reproductive Sciences 2013; 20(3 SUPPL. 1): 145A.
603. Hickey R, Wilson EM, Witek RP, et al. Development of a novel rat bioreactor to facilitate manufacture of human hepatocyte cell therapies for the treatment of patients with severe liver diseases. Xenotransplantation 2021; 28(5).
604. Hickey RD, Wilson EM, Witek RP, et al. Human hepatocytes expanded in a novel rat bioreactor can prevent hepatic failure when transplanted into a rodent model of hereditary tyrosinemia type 1. Hepatology 2020; 72(1 SUPPL): 810A-1A.
605. Hidemi F, Hideki A, Akiko F, et al. Mild Hyperbaric Oxygen Exposure Increases Cd16dim-cd56bright Natural Killer Cells In Healthy Individuals. Medicine & Science in Sports & Exercise 2021; 53: 489-.
606. Higginson D, Theodoratou E, Nair H, et al. An evaluation of respiratory administration of measles vaccine for prevention of acute lower respiratory infections in children. BMC public health 2011; 11 Suppl 3: S31.
607. Hilgers A, Frank J. Chronic fatigue syndrome: Immune dysfunction, the role of pathogens and toxic agents and neurological and cardiac alternations. Wiener Medizinische Wochenschrift 1994; 144(16): 399-406.
608. Hirokawa K, Utsuyama M, Hayashi Y, Kitagawa M, Makinodan T, Fulop T. Slower immune system aging in women versus men in the Japanese population. Immun Ageing 2013; 10(1): 19.
609. Ho MM, Baca-Estrada M, Conrad C, Karikari-Boateng E, Kang H-N. Implementation workshop of WHO guidelines on evaluation of malaria vaccines: Current regulatory concepts and issues related to vaccine quality, Pretoria, South Africa 07 Nov 2014. Vaccine 2015; 33(36): 4359-64.
610. Ho RTH, Wang C-W, Ng S-M, et al. The Effect of T'ai Chi Exercise on Immunity and Infections: A Systematic Review of Controlled Trials. Journal of Alternative & Complementary Medicine 2013; 19(5): 389-96.

611. Hoang VT, Gautret P. Infectious Diseases and Mass Gatherings. Curr Infect Dis Rep 2018; 20(11): 44.
612. Hod M, Kapur A, Sacks DA, et al. The International Federation of Gynecology and Obstetrics (FIGO) Initiative on gestational diabetes mellitus: A pragmatic guide for diagnosis, management, and care. International Journal of Gynecology and Obstetrics 2015; 131(Supplement 3): S173-S211.
613. Hodgkinson N, Kruger CA, Abrahamse H. Targeted photodynamic therapy as potential treatment modality for the eradication of colon cancer and colon cancer stem cells. Tumor Biology 2017; 39(10): 1-17.
614. Hoffman JR, Ratamess NA, Kang J, et al. Examination of the efficacy of acute L-alanyl-L-glutamine ingestion during hydration stress in endurance exercise. J Int Soc Sports Nutr 2010; 7: 8.
615. Hoffman-Goetz L, Zajchowski S. In vitro apoptosis of lymphocytes after exposure to levels of corticosterone observed following submaximal exercise. Journal of Sports Medicine & Physical Fitness 1999; 39(4): 269-74.
616. Hoffmann D, Wolfarth B, Hörterer HG, et al. Elevated Epstein-Barr virus loads and lower antibody titers in competitive athletes. J Med Virol 2010; 82(3): 446-51.
617. Hoffmann KF, Wynn TA, Dunne DW. Cytokine-mediated host responses during schistosome infections; walking the fine line between immunological control and immunopathology. Adv Parasitol 2002; 52: 265-307.
618. Hojan K, Milecki P, Kwiatkowska-Borowczyk E, Leporowska E. Physical exercises, inflammation, fatigue and aerobic fitness in prostate cancer patients undergoing antiandrogen-and radiotherapy. Archives of physical medicine and rehabilitation 2015; 96(10): e5.
619. Hojman P, Gehl J, Christensen JF, Pedersen BK. Molecular Mechanisms Linking Exercise to Cancer Prevention and Treatment. Cell Metab 2018; 27(1): 10-21.
620. Holland AE. Physiotherapy management of acute exacerbations of chronic obstructive pulmonary disease. Journal of Physiotherapy (Australian Physiotherapy Association) 2014; 60(4): 181-8.
621. Hollinger H, Carolan C, MacIver A. Tele-health interventions to support self-management in adults with rheumatoid arthritis: a systematic review. Rheumatology International 2021; 41(8): 1399-418.
622. Holz O, Timm P, Koschyk S, et al. Flow cytometric analysis of lymphocyte subpopulations in bronchoalveolar lavage fluid after repeated ozone exposure. International Archives of Occupational and Environmental Health 2001; 74(4): 242-8.
623. Hopker J, Beedie C. Possible implications of nervous system and immune system links in sports rehabilitation. SportEX Dynamics 2005; (3): 19-22.
624. Horneff G, Paetzke I, Neuen-Jacob E. Glycogenosis type V (McArdle's disease) mimicking atypical myositis. Clinical Rheumatology 2001; 20(1): 57-60.
625. Hornung F, Zheng L, Lenardo M, Dudley E, Scherer D, Ballard D. NF-kappaB regulates Fas/APO-1/CD95- and TCR-mediated apoptosis of T lymphocytes. European Journal of Immunology 1999; 29(3): 878-86.
626. Horton S, Gelband H, Jamison D, Levin C, Watkins D, Nugent R. Ranking 93 health interventions for low- and middle-income countries by cost-effectiveness. PLoS ONE 2017; 12(8): e0182951.
627. Hosken N, Trujillo C, Mahmood K, Higgins D, Plikaytis B. A multi-laboratory study of diverse RSV neutralization assays indicates feasibility for harmonization with an international standard. Vaccine 2017; 35(23): 3082-8.
628. Hosseinzadeh A, Stylianou M, Lopes JP, et al. Stable redox-cycling nitroxide tempol has antifungal and immune-modulatory properties. Frontiers in Microbiology 2019; 10(AUG): 1843.
629. Hua Y, Shi X, Ba C, Liu X, Yang Y. The combination of pembrolizumab and bevacizumab in the treatment of cholangiocarcinoma brain metastases: Case report. Gut 2018; 67(Supplement 2): A28-A9.

630. Huang H, Xu H, Zhao J. A Novel Approach for Meniscal Regeneration Using Kartogenin-Treated Autologous Tendon Graft. American Journal of Sports Medicine 2017; 45(14): 3289-97.
631. Huang J, Ou H-Y, Lin J, et al. Hepatitis B vaccination reduces the risk of diabetes by 50%. Diabetes 2014; 63(SUPPL. 1): A388.
632. Huang Y, Liu Z, Liu S, Song F, Jin Y. Studies on the mechanism of Panax Ginseng in the treatment of deficiency of vital energy dementia rats based on urine metabolomics. J Chromatogr B Analyt Technol Biomed Life Sci 2022; 1191: 123115.
633. Huda T, Nair H, Theodoratou E, et al. An evaluation of the emerging vaccines and immunotherapy against staphylococcal pneumonia in children. BMC Public Health 2011; 11 Suppl 3(Suppl 3): S27.
634. Hunt HE, Sadr K, DeYoung AJ, Gortz S, Bugbee WD. The Role of Immunologic Response in Fresh Osteochondral Allografting of the Knee. American Journal of Sports Medicine 2014; 42(4): 886-91.
635. Hunter G, Cooley R, Sawicka K. New onset refractory status epilepticus (NORSE) lasting 110 days resulting in a positive outcome. Neurology 2016; 86(16 SUPPL. 1).
636. Huss DJ, Fontenot JD, Mehta DS, et al. IL-2Rbetagamma-signaling contributes to regulatory T cell maintenance and stability in daclizumab HYP-treated RRMS patients. Multiple Sclerosis 2014; 20(1 SUPPL. 1): 482.
637. Huss DJ, Sharma A, Fontenot JD, et al. In vivo maintenance of human regulatory T cells during CD25 blockade. Journal of Neuroimmunology 2014; 275(1-2): 201.
638. Hussain R, Zacharias T, Zein S, et al. Human aquaporin 4281-300 is the immunodominant linear determinant in the context of HLA-DRB1*03:01: Relevance for diagnosing and monitoring patients with neuromyelitis optica. Archives of Neurology 2012; 69(9): 1125-31.
639. Hyatt A, Gough K, Murnane A, et al. i-Move, a personalised exercise intervention for patients with advanced melanoma receiving immunotherapy: a randomised feasibility trial protocol. BMJ Open 2020; 10(2): e036059.
640. Ibisch C. KN Oncology: New pharmacological targets. Journal of Veterinary Pharmacology and Therapeutics 2015; 38(SUPPL. 1): 68.
641. Idorn M, Thor Straten P. Chemokine Receptors and Exercise to Tackle the Inadequacy of T Cell Homing to the Tumor Site. Cells 2018; 7(8).
642. Ihan A. Physical exercise and the immune system. Zdravniski Vestnik 2014; 83(2): 158-68.
643. Iijima O, Miyake K, Nakamura A, et al. Bone marrow cell based enzyme replacement prolongs survival and improves disease phenotypes in a mouse model of lethal hypophosphatasia. Blood 2013; 122(21).
644. Imai T, Seki S, Dobashi H, Ohkawa T, Habu Y, Hiraide H. Effect of weight loss on T-cell receptor-mediated T-cell function in elite athletes. Medicine and Science in Sports and Exercise 2002; 34(2): 245-50.
645. Imayama S, Shimozono Y, Urabe A, Hori Y. A simple method for measuring the amount of immunoglobulin A secreted onto the skin surface. Acta Dermato-Venereologica 1995; 75(3): 212-7.
646. Ingwersen W. Your client credibility: Are your pharmacy practices helping or hindering? Canadian Veterinary Journal 2004; 45(8): 695-9.
647. Institute of Medicine Committee on Military Nutrition R. Military Strategies for Sustainment of Nutrition and Immune Function in the Field. Washington (DC): National Academies Press (US)
Copyright 1999 by the National Academy of Sciences. All rights reserved.; 1999.
648. Irct2015050113690N. Effect of coping skills training on diabetic. https://trialsearchwhoint/Trial2aspx?TrialID=IRCT2015050113690N4 2015.

649. Irct2015122317994N. Effectiveness and safety of Rosa Canina in treatment of patients with Type 2 Diabetes Mellitus. https://trialsearchwhoint/Trial2aspx?TrialID=IRCT2015122317994N4 2016.
650. Irct20200202046339N. Examining the Effectiveness of solution-focused brief consolation on mothers with gestational diabetes under insulin treatment. https://trialsearchwhoint/Trial2aspx?TrialID=IRCT20200202046339N1 2020.
651. Ishikawa S, Kayaba K, Hayasaka S, et al. Prevalence of Chlamydia pneumoniae in Japanese rural districts: Association of smoking and physical activity with Chlamydia pneumoniae seropositivity. Internal Medicine 2003; 42(10): 960-6.
652. Ishimoto R, Kikuchi K, Ura N, et al. A case of Buerger's disease solitary involved in the left subclavian and axillary artery. Kokyu to junkan Respiration & circulation 1993; 41(5): 475-9.
653. Islam D, Ruamsap N, Aksomboon A, Khantapura P, Srijan A, Mason CJ. Immune responses to Campylobacter (C. jejuni or C. coli) infections: A two-year study of US forces deployed to Thailand. APMIS 2014; 122(11): 1102-13.
654. Isrctn. Pulmonary Hypertension: assessment of Cell Therapy. https://trialsearchwhoint/Trial2aspx?TrialID=ISRCTN14519481 2006.
655. Isrctn. Repeated application of gene therapy in cystic fibrosis patients. https://trialsearchwhoint/Trial2aspx?TrialID=ISRCTN71164341 2012.
656. Isrctn. PERCEPT - myeloma transplant prehab study. https://trialsearchwhoint/Trial2aspx?TrialID=ISRCTN15875290 2019.
657. Isrctn. Preconception-early childhood telephone-based intervention to optimize growth and development among children in Canada: a Healthy Life Trajectory Initiative (HeLTI-Canada). https://trialsearchwhoint/Trial2aspx?TrialID=ISRCTN13308752 2019.
658. Isrctn. Evaluating nasal sprays and physical activity/stress management in reducing respiratory infections in primary care. https://trialsearchwhoint/Trial2aspx?TrialID=ISRCTN17936080 2020.
659. Isrctn. Safety and acceptability of exercise for chronic lymphocytic leukaemia. https://trialsearchwhoint/Trial2aspx?TrialID=ISRCTN55166064 2021.
660. Ixtlahuac JG. I.12 Interventional Radiology and RFA. Journal of Thoracic Oncology 2019; 14(11 Supplement 2): S1161.
661. Jackson JL. Capsule Commentary on Perlman et al., Efficacy and Safety of Massage for Osteoarthritis of the Knee: a Randomized Clinical Trial. JGIM: Journal of General Internal Medicine 2019; 34(3): 444-.
662. Jackson LA, Shi H, Acevedo JF, et al. Gelatin methacryloyl hydrogel as an alternative anchor for mesh augmented prolapse repair. International urogynecology journal 2019; 30(1): S79-S80.
663. Jacob ME, Lo-Ciganic W-H, Simkin-Silverman LR, et al. The preventive services use self-efficacy (PRESS) scale in older women: development and psychometric properties. BMC health services research 2016; 16: 71.
664. Jacobs J-P, Raschdorff B, Grosschupff G, et al. The unenlarged lymph nodes of HIV-1-infected, asymptomatic patients with high CD4 T cell counts are sites for virus replication and CD4 T cell proliferation. The impact of highly active antiretroviral therapy. Journal of Experimental Medicine 1998; 187(6): 949-59.
665. Jäger R, Mohr AE, Carpenter KC, et al. International Society of Sports Nutrition Position Stand: Probiotics. J Int Soc Sports Nutr 2019; 16(1): 62.
666. Jager R, Mohr AE, Pugh JN. Recent advances in clinical probiotic research for sport. Current opinion in clinical nutrition and metabolic care 2020; 23(6): 428-36.
667. Jager R, Purpura M, Mohr AE, et al. International Society of Sports Nutrition Position Stand: Probiotics. Journal of the International Society of Sports Nutrition 2019; 16(1): 62.
668. Jahan S. The Dilemma, Conversion Disorder or Stiff Person Syndrome, a Case Report. CNS spectrums 2021; 26(2): 145-6.

669. Jahnke R, Larkey L, Rogers C, Etnier J, Lin F. A comprehensive review of health benefits of qigong and tai chi. American journal of health promotion 2010; 24(6): e1-e25.
670. Jain D, Natarajan S. Segmental pigmentary anomaly and ataxia telangiectasia. British Journal of Dermatology 2015; 173(SUPPL. 1): 167.
671. Jakobsson J, Cotgreave I, Furberg M, Arnberg N, Svensson M. Potential Physiological and Cellular Mechanisms of Exercise That Decrease the Risk of Severe Complications and Mortality Following SARS-CoV-2 Infection. Sports (Basel) 2021; 9(9).
672. Jannin A, Douillard C, Vantyghem M-C. Rare causes of hypoglycemia in adults. Annales d'Endocrinologie 2020; 81(2-3): 110-7.
673. Janse Van Rensburg C, Fletcher L, Viljoen M, et al. Efficacy ofanexercise programon the functional capacity and disease activity in females with rheumatoid arthritis. Journal of science and medicine in sport 2010; 13: e60.
674. Janssen TWJ, Glaser RM, Shuster DB. Clinical efficacy of electrical stimulation exercise training: Effects on health, fitness, and function. Topics in Spinal Cord Injury Rehabilitation 1998; 3(3): 33-49.
675. Jefferson T, Mugford M, Gray A, Demicheli V. An exercise on the feasibility of carrying out secondary economic analyses. Health economics 1996; 5(2): 155-65.
676. Jegan R, Subha Hency Jose P, Rajalakshmy P, Nimi WS. 2019-nCoV effects, transmission and preventive measures: an overview. Journal of Public Health (Germany) 2021.
677. Jennings I, Kitchen S, Kitchen D, et al. Discrepant interpretation of hit screening results on the same sample-data from a UK neqas for blood coagulation exercise. Research and Practice in Thrombosis and Haemostasis 2020; 4(SUPPL 1): 274-5.
678. Jensen AWP, Carnaz Simoes AM, Holmen Olofsson G, Thor Straten P. Adrenergic signaling in immunotherapy of cancer: Friend or foe? Cancers 2021; 13(3): 1-16.
679. Jensen W, Oechsle K, Baumann HJ, et al. Effects of exercise training programs on physical performance and quality of life in patients with metastatic lung cancer undergoing palliative chemotherapy--a study protocol. Contemporary clinical trials 2014; 37(1): 120-8.
680. Jewett JF, Hecht FM. Preventive health care for adults with HIV infection. Journal of the American Medical Association 1993; 269(9): 1144-53.
681. Jiang Y, Xiao W, Wang H, Zhang Y, Xu X, Zhan H. Effects of Salmeterol/Fluticasone propionate on inflammation and innate immunity in COPD. Respirology 2009; 14(SUPPL. 3): A159.
682. Jicha GA. Is passive immunization for Alzheimer's disease 'alive and well' or 'dead and buried'? Expert Opinion on Biological Therapy 2009; 9(4): 481-91.
683. Johansen HK, Aanaes K, Buchwald CV, et al. Early genetic adaptation associated with chronic pseudomonas aeruginosa airways infection. Pediatric Pulmonology 2012; 47(SUPPL. 35): 328-9.
684. John TJ, Cooksley G. Hepatitis B vaccine boosters: is there a clinical need in high endemicity populations? J Gastroenterol Hepatol 2005; 20(1): 5-10.
685. Jolles S, Michallet M, Agostini C, et al. An international delphi consensus building approach to patient assessment and use of immunoglobulin treatment for secondary immune deficiencies in hematological malignancy. HemaSphere 2020; 4(Supplement 1): 872.
686. Jones C, Smith-MacDonald L, Miguel-Cruz A, et al. Virtual Reality-Based Treatment for Military Members and Veterans With Combat-Related Posttraumatic Stress Disorder: Protocol for a Multimodular Motion-Assisted Memory Desensitization and Reconsolidation Randomized Controlled Trial. JMIR Res Protoc 2020; 9(10): e20620.
687. Jones G, Hawkins K, Mullin R, et al. Understanding how adherence goals promote adherence behaviours: a repeated measure observational study with HIV seropositive patients. BMC Public Health 2012; 12(1): 587-.
688. Jordan BD, Kim A, Kim A. Levetiracetam to enhance functional recovery in opsoclonus-myoclonus syndrome: A case report. PM and R 2013; 5(9 SUPPL. 1): S258.

689. Jprn U. Efficacy of exercise on physical and mental health and beauty. https://trialsearchwhoint/Trial2aspx?TrialID=JPRN-UMIN000034412 2018.
690. Judah G, Bicknell C, Darzi A, et al. A randomised controlled trial on the impact of financial incentives on attendance at diabetic eye screening in London. Diabetologia 2016; 59(1 Supplement 1): S65.
691. Jusi RLG. Predicting the unpredictable pulmonary hypertension: A rare complication of multiple myeloma. Respirology 2011; 16(SUPPL. 2): 297.
692. K L, B L, H E, M H, N W, J S. Vitamin D and Athletic Performance: Perspectives and Pitfalls. / Vitamin D und Leistungssport: Perspektiven und Fallstricke. German Journal of Sports Medicine / Deutsche Zeitschrift fur Sportmedizin 2020; 71(2): 35-41.
693. Kaaijk P, Luytjes W. Are we prepared for emerging flaviviruses in Europe? Challenges for vaccination. Human Vaccines and Immunotherapeutics 2018; 14(2): 337-44.
694. Kaesler S, Skabytska Y, Rocken M, et al. Innate immune signal triggered co-factor dependent anaphylaxis is mediated by TLR ligation. Journal of Investigative Dermatology 2015; 135(SUPPL. 2): S2.
695. Kahl CG, Deas C. Exercise-Induced Anaphylaxis in an Air Force Aviator Taking a HMG-CoA Reductase Inhibitor: A Case Report and Review of the Presentation, Diagnoses, and Treatment. Military medicine 2017; 182(5): e1816-e9.
696. Kallio S, Kukkonen K, Savilahti E, Kuitunen M. Early probiotic prophylaxis reduces allergic symptoms in 13-year follow-up. Allergy 2017; 72: 553-.
697. Kalodera Z, Jagodic J. What should pharmacists know about ginseng. Farmaceutski Glasnik 2003; 59(6): 249-55.
698. Kamimura A, Trinh HN, Weaver S, et al. Knowledge and Perceptions of Influenza Vaccinations Among College Students in Vietnam and the United States. Journal of Preventive Medicine & Public Health 2017; 50(4): 268-73.
699. Kane EM. Achieving clinical equality in an influenza pandemic: patent realities. Seton Hall law review 2009; 39(4): 1137-72.
700. Kapasi ZF, Catlin PA, Adams MA, Glass EG, McDonald BW, Nancarrow AC. Effect of duration of a moderate exercise program on primary and secondary immune responses in mice. Physical Therapy 2003; 83(7): 638-47.
701. Kapasi ZF, Catlin PA, Beck J, Roehling T, Smith K. The Role of Endogenous Opioids in Moderate Exercise Training-Induced Enhancement of the Secondary Antibody Response in Mice. Physical Therapy 2001; 81(11): 1801-9.
702. Kapasi ZF, Catlin PA, Joyner DR, Lewis ML, Schwartz AL, Townsend EL. The effects of intense physical exercise on secondary antibody response in young and old mice. Physical Therapy 2000; 80(11): 1076-86.
703. Karol MH. Respiratory allergy: What are the uncertainties? Toxicology 2002; 181-182: 305-10.
704. Karrow NA, McCay JA, Brown R, Musgrove D, Munson AE, White KL, Jr. Oxymetholone modulates cell-mediated immunity in male B6C3F1 mice. Drug Chem Toxicol 2000; 23(4): 621-44.
705. Karussis D, Shor H, Yachnin J, et al. T cell vaccination benefits relapsing progressive multiple sclerosis patients: a randomized, double-blind clinical trial. PLoS One 2012; 7(12): e50478.
706. Kass-Iliyya L, Snowden JA, Thorpe A, et al. Autologous haematopoietic stem cell transplantation for refractory stiff-person syndrome: the UK experience. J Neurol 2021; 268(1): 265-75.
707. Kastrup J, Haack-Sørensen M, Juhl M, et al. Cryopreserved Off-the-Shelf Allogeneic Adipose-Derived Stromal Cells for Therapy in Patients with Ischemic Heart Disease and Heart Failure-A Safety Study. Stem Cells Transl Med 2017; 6(11): 1963-71.
708. Kathuria KR, Davis MM, Chen B, et al. Maria-I: A Deep-Learning Approach for Accurate Prediction of MHC Class I Tumor Neoantigen Presentation. Blood 2019; 134(Supplement 1): 84.

709. Kaufman J, Attwell K, Hauck Y, et al. Designing a multi-component intervention (P3-MumBubVax) to promote vaccination in antenatal care in Australia. Health Promotion Journal of Australia 2021; 32(3): 391-8.
710. Kaufman J, Attwell K, Tuckerman J, et al. Feasibility and acceptability of the multi-component P3-MumBubVax antenatal intervention to promote maternal and childhood vaccination: A pilot study. Vaccine 2020; 38(24): 4024-31.
711. Kaufman ZA, Kaufman EB, Dringus S, Weiss HA, Delany-Moretlwe S, Ross DA. Baseline results: of a cluster-randomised trial assessing the effectiveness of sport-based HIV Prevention in South African Schools. Sexually transmitted infections 2013; 89.
712. Kaushik D, Shah PK, Mukherjee N, et al. Effects of yoga in men with prostate cancer on quality of life and immune response: a pilot randomized controlled trial. Prostate Cancer Prostatic Dis 2021.
713. Kawakami K, Ohkusa Y, Kuroki R, et al. Effectiveness of pneumococcal polysaccharide vaccine against pneumonia and cost analysis for the elderly who receive seasonal influenza vaccine in Japan. Vaccine 2010; 28(43): 7063-9.
714. Kaye EM, Mendell JR, Rodino-Klapac L, et al. Eteplirsen, a phosphorodiamidate morpholino oligomer (PMO) for the treatment of Duchenne muscular dystrophy (DMD): 3.2 year update on six-minute walk test (6MWT), pulmonary function testing (PFT), and safety. European Journal of Paediatric Neurology 2015; 19(SUPPL. 1): S69.
715. Kct. Human trial study to evaluate the efficacy and safety of ISD on immune function. https://trialsearchwhoint/Trial2aspx?TrialID=KCT0004387 2019.
716. Keast D, Cameron K, Morton AR. Exercise and the immune response. Sports Medicine 1988; 5(4): 248-67.
717. Kelso JM, Cockrell GE, Helm RM, Burks AW. Common allergens in avian meats. J Allergy Clin Immunol 1999; 104(1): 202-4.
718. Kempa K, Sadowska-Krepa E, Jagsz S, Sobczak A, Szoltysek I, Klapcinska B. Evaluation of autoantibodies against oxidized LDL (oLAB) and blood antioxidant status in professional soccer players. International Journal of Sports Medicine 2005; 26(1): 71-8.
719. Kempf W, Koberle M, Biedermann T, et al. Innate immune signal triggered cofactor dependent anaphylaxis is mediated by TLRs. Allergy: European Journal of Allergy and Clinical Immunology 2015; 70(SUPPL. 101): 64-5.
720. Kenanidou E. Intervention program to modify the smoking habit in employee group of Athens' social welfare organization using motivational interviewing techniques and Trans theoretical Model of Behavior Change. Tobacco Induced Diseases 2014; 12(SUPPL. 1).
721. Kenet G. Emicizumab use in pups and children. Haemophilia 2021; 27(SUPPL 2): 11-2.
722. Kenis V, Melchenko E, Petrova D, Kozhevnikov A. Secondary subacute arthritis in children with diastrophic dysplasia and rmed. Annals of the Rheumatic Diseases 2020; 79(SUPPL 1): 1789.
723. Kepinska M, Bednarek J, Teleglow A, Dabrowski Z, Szygula Z. A comparison of the efficiacy of three different cryotherapy treatments used in the athletic recovery of sportspeople - Literature review. Medicina Sportiva 2013; 17(3): 142-6.
724. Kerr C. Translating "mind-in-body": two models of patient experience underlying a randomized controlled trial of Qigong. Culture, Medicine & Psychiatry 2002; 26(4): 419-47.
725. Kerse NM, Flicker L, Jolley D, Arroll B, Young D. Improving the health behaviours of elderly people: randomised controlled trial of a general practice education programme. BMJ (Clinical research ed) 1999; 319(7211): 683-7.
726. Kevere L, Kramina S, Purvina S, et al. Anti-NMDA receptor autoimmune encephalitis in psychiatric practice. European Neuropsychopharmacology 2015; 25(SUPPL. 2): S225.
727. Khalsa SBS, Klatt MD, Park CL. Yoga in the occupational setting: Efficacy research studies. Global Advances in Health and Medicine 2018; 7: 22-3.

728. Khammari A, Limacher J-M, Nguyen J-M, et al. Intra-lesional administrations of TG1042 (adenovirus expressing interferon-) combined with adoptive TIL transfer in patients with metastatic melanoma. Journal of Clinical Oncology 2012; 30(15 SUPPL. 1).
729. Khan ML, Malik A, Ruhi U, Al-Busaidi A. Conflicting attitudes: Analyzing social media data to understand the early discourse on COVID-19 passports. Technol Soc 2022; 68: 101830.
730. Khan MM, Zander DS, Gupta N. Placental transmogrification of the lung in a patient with anti-synthetase syndrome. American Journal of Respiratory and Critical Care Medicine 2018; 197(MeetingAbstracts).
731. Khanferyan R, Radzhabkadiev R, Savtchenko E, DuBuske L. Energy consumption vs cytokine concentrations in elite athletes. Allergy: European Journal of Allergy and Clinical Immunology 2018; 73(Supplement 105): 800-1.
732. Khanli HM, Richardson P, Strong E. A case of opsoclonus-myoclonus syndrome with positive serum antibody against ganglionic (Alpha-3) acetylcholine receptor. Neurology 2020; 94(15 Supplement).
733. Khanna A, Jopson L, Howel D, et al. Rituximab Is Ineffective for Treatment of Fatigue in Primary Biliary Cholangitis: a Phase 2 Randomized Controlled Trial. Hepatology (baltimore, md) 2018.
734. Khondowe O, Nikodem VC, Frantz JM, Harper K. A physical activity programme to improve motor and cognitive development in HIV positive children on antiretroviral therapy: A randomised controlled trial. African Journal for Physical, Health Education, Recreation & Dance 2015; 21(4:1): 1187-99.
735. Khosravi N, Stoner L, Farajivafa V, Hanson ED. Exercise training, circulating cytokine levels and immune function in cancer survivors: A meta-analysis. Brain Behav Immun 2019; 81: 92-104.
736. Khoury EL, Marshall LA. Luteinization of human granulosa cells in vivo is associated with expression of MHC class II antigens. Cell and Tissue Research 1990; 262(2): 217-24.
737. Kiecolt-Glaser JK, Glaser R. Psychoneuroimmunology: can psychological interventions modulate immunity? J Consult Clin Psychol 1992; 60(4): 569-75.
738. Kiefel V, Sachs UJ, Bein G, Santoso S, Kroll H. Report from the 15th international platelet immunology workshop. Vox Sanguinis 2010; 99(SUPPL. 1): 51-2.
739. Kiewiet MBG, Faas MM, de Vos P. Immunomodulatory protein hydrolysates and their application. Nutrients 2018; 10(7): 904.
740. Kikuchi S, Sawada J, Saito T, et al. A case of anti-Th/To antibody-positive systemic sclerosis with muscle symptoms and interstitial pneumonia. Clinical Neurology 2021; 61(4): 228-33.
741. Kiladjian JJ, Mesa RA, Hoffman R. The renaissance of interferon therapy for the treatment of myeloid malignancies. Blood 2011; 117(18): 4706-15.
742. Kilgo WA. A multiple sclerosis-like presentation coincident with myasthenia gravis and autoimmune hepatitis successfully treated with rituximab. Multiple Sclerosis Journal 2019; 25(Supplement 1): 58-9.
743. Kim J. Coexisting case of Systemic erythematosus lupus, myasthenia gravis and Graves' disease. International Journal of Rheumatic Diseases 2018; 21(Supplement 1): 111-4.
744. Kim JH, Kim JH, Sutikno LA, et al. Identification of the minimum region of flatfish myostatin propeptide (Pep45-65) for myostatin inhibition and its potential to enhance muscle growth and performance in animals. PLoS One 2019; 14(4): e0215298.
745. Kim K, Gu MO, Jung JH, et al. Efficacy of a home-based exercise program after thyroidectomy for thyroid cancer patients. Thyroid 2018; 28(2): 236-45.
746. Kim KH, Vucko E, Burton BK, Desai AK, Kishnani P. Development of high sustained IgG antibody titers and corresponding clinical decline in an adolescent with atypical infantile Pompe disease after 11+ years on enzyme replacement therapy with alglucosidase alfa. Molecular Genetics and Metabolism 2020; 129(2): S89.
747. King NJ, Shrestha B, Kesson AM. Immune modulation by flaviviruses. Advances in virus research 2003; 60: 121-55.

748. Kinscherf R, Kirschfink M, Stroberl G, Weicker H. Influence on myoadenylate deaminase function in rat skeletal muscle after homologous and heterologous immunization with the purified enzyme. / Influence d ' une immunisation homologue et heterologue avec l ' enzyme purifiee sur la fonction myodenylate deaminase du muscle squelettique du rat. International Journal of Sports Medicine 1993; 14(4): 214-9.
749. Kinsella P. Review: behavioural interventions show the most promise for chronic fatigue syndrome. Evidence Based Nursing 2002: 46-.
750. Kinzfogl J, Hangoc G, Broxmeyer HE. The neurexin i alpha/neurexophilin axis as an anti-proliferative factor in human cord blood and murine bone marrow hematopoiesis. Blood 2010; 116(21).
751. Klapcinska B, Kempa K, Sobczak A, Sadowska-Krepa E, Jagsz S, Szoltysek I. Evaluation of Autoantibodies Against Oxidized LDL (oLAB) and Blood Antioxidant Status in Professional Soccer Players. International Journal of Sports Medicine 2005; 26(1): 71-8.
752. Klein G. B-cell neoplasia in a developmental framework. International Journal of Developmental Biology 1995; 39(5): 713-8.
753. Klement E, Sela T, Kayouf R, et al. Long-term immunity in young adults after a single dose of inactivated Hepatitis A vaccines. Vaccine 2006; 24(20): 4328-32.
754. Kleynhans J, Treurnicht FK, Cohen C, et al. Outbreak of influenza A in a boarding school in South Africa, 2016. Pan Afr Med J 2019; 33: 42.
755. Knerer G, Ismaila A, Pearce D. Health and economic impact of PHiD-CV in Canada and the UK: a Markov modelling exercise. Journal of Medical Economics 2012; 15(1): 61-76.
756. Knight C, Paisley S, Wight J, Jones ML. Economic modelling of different treatment strategies for haemophilia A with high-responding inhibitors. Haemophilia 2003; 9(4): 521-40.
757. Knight SC, Panoskaltsis N, McCarthy NE. Myelopoiesis of acute inflammation: lessons from TGN1412-induced cytokine storm. Cancer Immunology, Immunotherapy 2021; 70(4): 1155-60.
758. Knopf PM, Petzke MM, McLaren DJ. Immobilization of Schistosoma mansoni miracidia by activation of the alternate complement pathway at unusually high serum dilution. Parasite Immunol 1993; 15(6): 325-37.
759. Kobak S, Sever F, Sivrikoz O. Coexistence of systemic sclerosis and sarcoidosis: A case report. Clinical and Experimental Rheumatology 2014; 32(2 SUPPL. 81): S138.
760. Kohl K, Ross DA. Impact of Health Check-Ups In Early And Mid/Late Adolescence In Low And Middle-Income Countries. Journal of Adolescent Health 2019; 64(2 Supplement): S90-S1.
761. Kohut M, Senchina D, Konopka D, et al. Effect of exercise on immunity and depression vary by body weight status and type of exercise. Brain, Behavior, and Immunity 2011; 25(SUPPL. 2): S241.
762. Kohut ML, Boehm GW, Moynihan JA. Moderate exercise is associated with enhanced antigen-specific cytokine, but not IgM antibody production in aged mice. Mech Ageing Dev 2001; 122(11): 1135-50.
763. Kohut ML, Senchina DS. Reversing age-associated immunosenescence via exercise. Exercise immunology review 2004; 10: 6-41.
764. Kolak A, Kamińska M, Wysokińska E, et al. The problem of fatigue in patients suffering from neoplastic disease. Contemp Oncol (Pozn) 2017; 21(2): 131-5.
765. Koletzko L, Klucker E, Le Thi TG, et al. Following Pediatric and Adult IBD Patients through the COVID-19 Pandemic: Changes in Psychosocial Burden and Perception of Infection Risk and Harm over Time. J Clin Med 2021; 10(18).
766. Kolmykova N, Kiryukhina S, Labunskiy D, Razgadova E, Kustov M. Immune-Mediated and mercury intoxication ataxias: Anti-GAD antibodies and dynamic stabilometric assessment. Movement Disorder 2021; 36(SUPPL 1): S13-S4.
767. Koopman JS, Eckert EA, Greenberg HB, Strohm BC, Isaacson RE, Monto AS. Norwalk virus enteric illness acquired by swimming exposure. Am J Epidemiol 1982; 115(2): 173-7.

768. Korochkin IM, Chukaeva II, Korolev VS, Litvinova SN, Klebanov GI. Informative value of immunologic indicators at different periods of acute myocardial infarction. Kardiologiia 1989; 29(10): 37-9.
769. Kortum P, Edwards C, Richards-Kortum R, Kortum P, Edwards C, Richards-Kortum R. The impact of inaccurate Internet health information in a secondary school learning environment. Journal of Medical Internet Research 2008; 10(2): e17-e.
770. Kovac Z. Integrative power of pathophysiology in postgenomic era of precision medicine. Clujul Medical 2017; 90(Supplement 5): S5-S6.
771. Kovacsovics-Bankowski M, Walker E, Chisholm L, et al. Reshaping CD4 and CD8 memory T cell proliferation by treating cancer patients with an OX40 agonist: Immuno-logic assessment of a phase I clinical trial. Journal of Immunotherapy 2010; 33(8): 912.
772. Kovacsovics-Bankowski M, Warby C, Kim S, et al. A detailed characterization of peripheral blood lymphocytes in patients with myeloproliferative disease treated with pegylated-interferon alpha. Journal for ImmunoTherapy of Cancer 2015; 3(SUPPL. 2).
773. Kraig E, Linehan LA, Liang H, et al. A randomized control trial to establish the feasibility and safety of rapamycin treatment in an older human cohort: immunological, physical performance, and cognitive effects. Experimental gerontology 2018; 105: 53-69.
774. Krieger S. Multiple sclerosis therapeutic pipeline: Opportunities and challenges. Mount Sinai Journal of Medicine 2011; 78(2): 192-206.
775. Krishnan SM, Nordlohne J, Dietz L, et al. Assessing the use of the sgc stimulator bay-747, as a potential treatment for duchenne muscular dystrophy. International Journal of Molecular Sciences 2021; 22(15): 8016.
776. Kristiansson K, Perola M, Gudelj I, et al. Molecular pathways mediating immunosuppression in response to prolonged intensive physical training, low-energy availability, and intensive weight loss. Frontiers in Immunology 2019; 10(MAY): 907.
777. Krone CA, Ely JTA. Controlling hyperglycemia as an adjunct to cancer therapy. Integrative Cancer Therapies 2005; 4(1): 25-31.
778. Kruger K, Volker K, Klocke R, Nikol S, Waltenberger J, Mooren FC. Exercise delays neutrophil apoptosis by a G-CSF-dependent mechanism. Journal of Applied Physiology 2012; 113(7): 1082-90.
779. Kulikova VA, Nedostup AV, Blagova OV, et al. Treatment efficacy of arrhythmias and dilated cardiomyopathy syndrome of immune-inflammatory nature using plasmapheresis. Russian Journal of Cardiology 2018; 23(12): 32-43.
780. Kumar L, Chhibber S, Harjai K. Zingerone inhibit biofilm formation and improve antibiofilm efficacy of ciprofloxacin against Pseudomonas aeruginosa PAO1. Fitoterapia 2013; 90: 73-8.
781. Kunstreich M, Oommen PT, Borkhardt A, et al. Paraneoplastic limbic encephalitis with SOX1 and PCA2 antibodies and relapsing neurological symptoms in an adolescent with Hodgkin lymphoma. European Journal of Paediatric Neurology 2017; 21(4): 661-5.
782. Kunwar S, Parekh JD, Chilukuri RS, Andukuri VA. Necrotizing Autoimmune myopathy: A case report on statin induced rhabdomyolysis requiring immunosuppressive therapy. Drug Discoveries and Therapeutics 2018; 12(5): 315-7.
783. Kurabayashi H, Machida I, Handa H, et al. Effects of physical therapy on cytokines and two color analysis-lymphocyte subsets in patients with cerebrovascular diseases. J Med 1999; 30(1-2): 31-7.
784. Kurbanov NA. The effectiveness of glutathione on the immunological status of patients with dilated cardiomyopathy. European Journal of Heart Failure 2015; 17(SUPPL. 1): 89.
785. Kurihara S, Shibakusa T, Tanaka KA. Cystine and theanine: amino acids as oral immunomodulative nutrients. Springerplus 2013; 2: 635.
786. Kuriyama Y, Yoshida Y. Efficacy of dietary supplement contained proteoglycan extracted from salmon nasal cartilage on knee uncomfortableness in healthy volunteers -A randomized,

double-blind placebo-controlled intergroup trial. Japanese Pharmacology and Therapeutics 2017; 45(11): 1795-808.
787. Kurtoglu A, Manegold S, Minkwitz S, et al. Histological and molecular analysis of acute vs. Chronic ruptures of Achilles tendons. Journal of Orthopaedic Research 2017; 35(Supplement 1).
788. Kury CM, Silva JPd, Vitral CL, Pinto MA, Cruz OG. Hepatitis A seroprevalence in public school children in Campos dos Goytacazes, Rio de Janeiro State, Brazil, prior to the introduction of the hepatitis A universal childhood vaccination. Cadernos de saude publica 2016; 32(11): e00175614.
789. Kütting B, Drexler H. Evaluation of skin-protective means against acute and chronic effects of ultraviolet radiation from sunlight. Curr Probl Dermatol 2007; 34: 87-97.
790. Kwak T, Al Zoubi M, Bhavith A, Rueda Rios C, Kumar S. Acute myocarditis in bodybuilder from coxsackievirus and thyrotoxicosis. Journal of Cardiology Cases 2016; 14(4): 123-6.
791. Kwak T, Rios CR, Aruni B, Kumar S. Acute myocarditis in a bodybuilder due to coxsackievirus infection and thyroid hormone abuse. Journal of the American College of Cardiology 2016; 67(13 SUPPL. 1): 1232.
792. Kwetkat A, Endres A-S, Leischker A, Heppner HJ. Vaccination in older adults: Compulsory or voluntary exercise? Deutsche Medizinische Wochenschrift 2020; 145(16): 1133-7.
793. Kwetkat A, Heppner HJ. Comorbidities in the Elderly and Their Possible Influence on Vaccine Response. Interdisciplinary topics in gerontology and geriatrics 2020; 43: 73-85.
794. La Sala L, Prattichizzo F, De Nigris V, et al. Inflammageing and metaflammation: The yin and yang of type 2 diabetes. Ageing Research Reviews 2018; 41: 1-17.
795. La Seta Catamancio S, Arnaudova R, Morandi P, et al. Manufacturing of HSV-TK engineered donor lymphocytes: A comparability study to improve the production process. Human Gene Therapy 2013; 24(12): A98.
796. La Torre G, Federici A. How to not detonate the bomb: the case of the Italian National Health Service. Public Health 2017; 153: 178-80.
797. Łagowska K, Bajerska J. Probiotic Supplementation and Respiratory Infection and Immune Function in Athletes: Systematic Review and Meta-Analysis of Randomized Controlled Trials. Journal of Athletic Training (Allen Press) 2021; 56(11): 1213-23.
798. Łagowska K, Bajerska J. Effects of probiotic supplementation on respiratory infection and immune function in athletes: systematic review and meta-analysis of randomized controlled trials. J Athl Train 2021; 56(11): 1213-23.
799. Lai HC, Chu K-H, Yong S-B. Anaphylaxis due to food-related anaphylaxis treated with omalizumab. Biomedical Research (India) 2018; 29(12): 2627-30.
800. Lakier Smith L. Overtraining, excessive exercise, and altered immunity: is this a T helper-1 versus T helper-2 lymphocyte response? Sports Med 2003; 33(5): 347-64.
801. Lam J, Wulff H. The lymphocyte potassium channels Kv1.3 and KCa3.1 as targets for immunosuppression. Drug Development Research 2011; 72(7): 573-84.
802. LaManca JJ, Sisto SA, Zhou XD, et al. Immunological response in chronic fatigue syndrome following a graded exercise test to exhaustion. J Clin Immunol 1999; 19(2): 135-42.
803. Lambrechts H, Needham T, Hoffman LC. Extending the interval between second vaccination and slaughter: I. Effects on growth, scrotal size and stress responses of immunocastrated ram lambs. Animal : an international journal of animal bioscience 2019; 13(9): 1952-61.
804. Lampe JW. Diet and Cancer Prevention Research: From Mechanism to Implementation. J Cancer Prev 2020; 25(2): 65-9.
805. Landgren CA, Hendrich S, Kohut ML. Low-level dietary deoxynivalenol and acute exercise stress result in immunotoxicity in BALB/c mice. J Immunotoxicol 2006; 3(4): 173-8.
806. Lane MA, Whitham RH. Tumor necrosis factor-alpha inhibitor (TNFi) associated demyelination: Under-recognized and not always acute. Multiple Sclerosis 2017; 23(Supplement 1): 81.
807. Lang T, Van Loon J, Bloomfield S, et al. Towards human exploration of space: the THESEUS review series on muscle and bone research priorities. NPJ Microgravity 2017; 3: 8.

808. LaPerriere AR, Antoni MH, Schneiderman N, et al. Exercise intervention attenuates emotional distress and natural killer cell decrements following notification of positive serologic status for HIV-1. Biofeedback and self-regulation 1990; 15(3): 229-42.
809. Larenas-Linnemann D, Rodríguez-Pérez N, Arias-Cruz A, et al. Enhancing innate immunity against virus in times of COVID-19: Trying to untangle facts from fictions. World Allergy Organ J 2020; 13(11): 100476.
810. Larun L, Brurberg KG, Odgaard-Jensen J, Price JR. Exercise therapy for chronic fatigue syndrome. Cochrane Database Syst Rev 2019; 10(10): Cd003200.
811. Larun L, Dalsbø TK, Hafstad E, Reinar LM. NIPH Systematic Reviews: Executive Summaries. Effects of Interventions for Prevention of Sick Leave and Disability for Health Personnel. Oslo, Norway: Knowledge Centre for the Health Services at The Norwegian Institute of Public Health (NIPH)
Copyright ©2014 by The Norwegian Institute of Public Health (NIPH). 2014.
812. Lattimore D. On the sidelines: An athlete's perspective of injury recovery. Sport & Exercise Psychology Review 2017; 13(2): 13-21.
813. Lau YN, Ng J, Lee SY, et al. A brief report on the clinical trial on neural mobilization exercise for joint pain in patients with rheumatoid arthritis. Z Rheumatol 2019; 78(5): 474-8.
814. Lautermilch J, Doyle-Baker P. The athlete and the flu vaccine: Melodrama, common sense or ignorance? Journal of Science and Medicine in Sport 2014; 18(SUPPL. 1): e54.
815. Lavery LA, Higgins KR, La Fontaine J, Zamorano RG, Constantinides GP, Kim PJ. Randomised clinical trial to compare total contact casts, healing sandals and a shear-reducing removable boot to heal diabetic foot ulcers. International wound journal 2015; 12(6): 710-5.
816. Le Guennec D, Goepp M, Rouge S, et al. Spontaneous physical activity in obese conditions modulates tissue hormonal signals leading to reduced mammary tumour growth. Clinical Nutrition ESPEN 2020; 40: 417.
817. Leal de Araujo J, Tizard I, Guo J, Heatley JJ, Rodrigues Hoffmann A, Rech RR. Are anti-ganglioside antibodies associated with proventricular dilatation disease in birds? PeerJ 2017; 5: e3144.
818. Leal-Lima A, Ferreira TC, Ferreira TMV, Nunes-Pinheiro DCS, Coelho PC. Successful Treatment and Management of Canine Ehrlichiosis-Leishmaniosis-Heartworm Comorbidity. Acta Scientiae Veterinariae 2021; 49: 687.
819. Leddy JJ, Chutkow JG. Myasthenia gravis in a collegiate football player. / Myasthenie grave chez un joueur universitaire de football americain. Medicine & Science in Sports & Exercise 2000; 32(12): 1975-9.
820. Lee AL, Goldstein RS. The role of telemedicine. ERS Monograph 2015; 2015(9781849840644): 269-96.
821. Lee J, Lee S, Park Y, et al. The Detection of Bovine Estrus by Lactoferrin Monoclonal Antibody. Animals (Basel) 2021; 11(6).
822. Lee LJ, DiSilvestro D, Ziouzenkova O. The effect of thermogenic and appetite suppressing capsules on mice fed a high fat diet. FASEB Journal 2014; 28(1 SUPPL. 1).
823. Lee M, Lee JS, Kim K, Kim C. Efficacy of immune-strengthening functional drinks in top-level athletes: a questionnaire survey-based research. Phys Act Nutr 2021; 25(3): 23-7.
824. Lee S, Ali S, Lee J, Peng H, Wang B. Battle of the lymphocytes a case of lymphoid interstitial pneumonia. American Journal of Respiratory and Critical Care Medicine 2019; 199(9).
825. Lee VY, Bohn-Goldbaum E, Fong J, Barr IG, Booy R, Edwards KM. Analgesic and adjuvant properties of exercise with vaccinations in healthy young population. Hum Vaccin Immunother 2021; 17(7): 2058-64.
826. Lee YT, Audette J, Al-Adawi S, Burke DT. Martial arts as sport and therapy. Journal of Sports Medicine and Physical Fitness 2007; 47(1): 96-102.
827. Lee YY, Yang YP, Huang PI, et al. Exercise suppresses COX-2 pro-inflammatory pathway in vestibular migraine. Brain Res Bull 2015; 116: 98-105.

828. Leite GSF, Resende Master Student AS, West NP, Lancha AH, Jr. Probiotics and sports: A new magic bullet? Nutrition 2019; 60: 152-60.
829. Lerner RA. Antibodies of predetermined specificity in biology and medicine. Advances in Immunology 1984; VOL. 36: 1-44.
830. Lesourd B, Mazari L. Nutrition and immunity in the elderly. Proceedings of the Nutrition Society 1999; 58(3): 685-95.
831. Lessler J, Metcalf CJE, Grenfell BT, Cutts FT. Impact on Epidemic Measles of Vaccination Campaigns Triggered by Disease Outbreaks or Serosurveys: A Modeling Study. PLoS Medicine 2016; 13(10): e1002144.
832. Levitt C, Shaw E, Wong S, et al. Systematic review of the literature on postpartum care: methodology and literature search results. Birth: Issues in Perinatal Care 2004; 31(3): 196-202.
833. Lévy-Bruhl D. [Basis for vaccine recommendations]. Med Sci (Paris) 2007; 23(4): 404-8.
834. Lewicki R, Tchorzewski H, Majewska E, Nowak Z, Baj Z. Effect of maximal physical exercise on T-Lymphocyte subpopulations and on Interleukin 1 (IL 1) and Interleukin 2 (IL 2) production in vitro. / IL1) and interleukin 2 .. (IL2) production in vitro .. (Effet d' un entrainement physique maximal sur les sous-populations de lymphocyte T et sur la production in vitro d' interleukine I et II. International Journal of Sports Medicine 1988; 9(2): 114-7.
835. Lewis AL, Eves FF. Prompts to Increase Stair Climbing in Stations: The Effect of Message Complexity. Journal of Physical Activity & Health 2012; 9(7): 954-61.
836. Li EK, Tam L, Tomlinson B. Leflunomide in the treatment of rheumatoid arthritis. Clinical Therapeutics 2004; 26(4): 447-59.
837. Li H, Yang P, Chen X, et al. A comparative study of sublingual and subcutaneous immunotherapy in mite-sensitive asthmatic children: a single center experience of 90 Chinese patients. International journal of clinical and experimental medicine 2016; 9(3): 6743-50.
838. Li N, Tian YW, Gao L, Xu Y, Gao X, Xu ZQ. Clinical efficacy of TP regimen chemotherapy combined with intravenous and intraperitoneal thermal cycling perfusion in the treatment of malignant seroperitoneum effusion patients with advanced ovarian cancer. Chinese journal of cancer prevention and treatment 2017; 24(2): 119-23.
839. Li QS, Tanaka S, Kisenge RR, Toyoda H, Azuma E, Komada Y. Activation-induced T cell death occurs at G1A phase of the cell cycle. Eur J Immunol 2000; 30(11): 3329-37.
840. Li XY, Shi ZH, Guan YL, Ji Y. Anti-N-methyl-D-aspartate-receptor antibody encephalitis combined with syphilis: A case report. World J Clin Cases 2020; 8(12): 2603-9.
841. Li Y, Issabekova A, Ogay V, Sekenova A, Saparov A. The therapeutic potential of mesenchymal stem cells in the treatment of atherosclerosis. Current Stem Cell Research and Therapy 2021; 16(7): 897-913.
842. Li Y, Wang T, Huang H, Yi H-L. Comparison of clinical data of childhood asthma in different periods in Changsha. Chinese Journal of Contemporary Pediatrics 2007; 9(6): 540-2.
843. Liang M, Liwen Z, Juan D, Yun Z, Yanbo D, Jianping C. Dysregulated TFR and TFH cells correlate with B-cell differentiation and antibody production in autoimmune hepatitis. J Cell Mol Med 2020; 24(7): 3948-57.
844. Liang W, Wang C, Chen W, Jong Y, Suzuki S, Nishino I. P.13Treatment experience of Taiwanese patients with anti-HMGCR myopathy. Neuromuscular Disorders 2019; 29(Supplement 1): S45.
845. Liang YY, Chen SH, Yun B. Clinical study on the effect regulating and tonifying lung and kidney method on comprehensive prognostic indicators of COPD in stable phase. World chinese medicine [shi jie zhong yi yao] 2015; 10(11): 1712-6.
846. Libicz S, Mercier B, Bigou N, Le Gallais D, Castex F. Salivary IgA Response of Triathletes Participating in the French Iron Tour. International Journal of Sports Medicine 2006; 27(5): 389-94.
847. Lichtenstein B. Psychoneuroimmunology and HIV. STEP Perspect 1995; 7(2): 6-9.

848. Liebert A, Bicknell B, Markman W, Kiat H. A potential role for photobiomodulation therapy in disease treatment and prevention in the era of COVID-19. Aging and Disease 2020; 11(6): 1352-62.
849. Lim CL, Mackinnon LT. The roles of exercise-induced immune system disturbances in the pathology of heat stroke: the dual pathway model of heat stroke. Sports Medicine 2006; 36(1): 39-64.
850. Lin B-J, Zhang H-Z. Research progress on emotional disorders in patients with malignant tumors. Chinese Journal of Cancer Prevention and Treatment 2020; 27(24): 2006-12.
851. Lin J, Feng W, Samoa R, et al. Hepatitis a and b are associated with an increased risk for diabetes mellitus. Endocrine Reviews 2013; 34(3 SUPPL. 1).
852. Lincoln DT, Singal PK, Al-Banaw A. Growth hormone in vascular pathology: Neovascularization and expression of receptors is associated with cellular proliferation. Anticancer Research 2007; 27(6 B): 4201-18.
853. Lindahl JF, Vrentas CE, Deka RP, et al. Brucellosis in India: results of a collaborative workshop to define One Health priorities. Tropical animal health and production 2020; 52(1): 387-96.
854. Lindley MR, Montgomery GS, Mickleborough TD. Effect of fish oil-derived omega-3 polyunsaturated fatty acid supplementation on exercise-induced bronchoconstriction and immune function in athletes. Physician and Sportsmedicine 2008; 36(1): 11-7.
855. Lira-Albarran S, Gonzalez L, Barrera D, et al. Ulipristal acetate administration at midcycle changes the receptivity gene expression profiling of endometrial biopsies taken during the window of implantation of the human menstrual cycle. Endocrine Reviews 2017; 38(3 Supplement 1).
856. Lisco A, Wong CS, Lage SL, et al. Identification of rare HIV-1-infected patients with extreme CD4+ T cell decline despite ART-mediated viral suppression. JCI Insight 2019; 4(8).
857. Litskevich L, Shpacovskaya N, Solodovnicova V, Skrahin A, Isaikina J. Effectiveness of mesenchimal stem cells in the treatment of patients with chronical obstructive pulmonary disease. European Respiratory Journal 2017; 50(Supplement 61).
858. Liu J, Qu M. Preparation and characterization of monoclonal antibody against collagen. Chinese Journal of Sports Medicine 1994; 13(2): 68-74.
859. Liu J, Tian X, Deng Y, et al. Risk factors associated with dengue virus infection in Guangdong province: A community-based case-control study. International Journal of Environmental Research and Public Health 2019; 16(4): 617.
860. Liu P, You J, Loo WTY, et al. The efficacy of Guolin-Qigong on the body-mind health of Chinese women with breast cancer: a randomized controlled trial. Qual Life Res 2017; 26(9): 2321-31.
861. Livingston J, Jackson M, Alrajihi Z, McGuire C, Klaassen RJ, Kirby-Allen M. Evaluating the Impact of Thrombopoietin Receptor Agonists Medications on Patient Outcomes and Quality of Life in Pediatric Immune Thrombocytopenia. Blood 2021; 138(Supplement 1): 4072.
862. Liwski DR, Liwski RS, Wong I. Donor-Specific Human Leukocyte Antigen Antibody Formation After Allograft Glenoid Reconstruction Occurs But Does Not Impact Clinicoradiographic Outcomes. American Journal of Sports Medicine 2021; 49(5): 1175-82.
863. Lockwood CM, Moon JR, Smith AE, et al. Low-calorie energy drink improves physiological response to exercise in previously sedentary men: a placebo-controlled efficacy and safety study [corrected] [published erratum appears in J STRENGTH CONDITION RES (LIPPINCOTT WILLIAMS WILKINS) 2010 Sep;24(9):2574-5]. Journal of Strength & Conditioning Research 2010; 24(8): 2227-38.
864. Logacheva IV, Leshchinskii LA, Zvorygin IA. Immunological characteristics of patients with acute coronary syndrome (unstable angina and myocardial infarction). Klinicheskaia meditsina 1999; 77(4): 23-5.
865. Lohwasser S, Andreesen R, Arndt H, Bross K, Wagner HM. Mediastinal space-occupying lesion in a patient with Graves' disease and history of osteosarcoma. Deutsche Medizinische Wochenschrift 1997; 122(36): 1070-4.

866. Lohwasser S, Wagner HM, Arndt H, Bross K, Andreesen R. [Space occupying lesion in the anterior mediastinum in a patient with Basedow disease and previously diagnosed osteosarcoma]. Dtsch Med Wochenschr 1997; 122(36): 1070-4.
867. Long BC. Aerobic conditioning (jogging) and stress inoculation interventions: an exploratory study of coping. International Journal of Sport Psychology 1993; 24(2): 94-109.
868. Lopatin S, Zabar S, Weinshel E, Gillespie C, Malter L. The use of an observed structured clinical examination to teach communication skills surrounding therapeutic drug monitoring. American Journal of Gastroenterology 2019; 114(Supplement 1): S21.
869. Lopes-Virella MF, Mironova M, Stephan E, Durazo-Arvizu R, Virella G. Role of simvastatin as an immunomodulator in type 2 diabetes. Diabetes Care 2004; 27(4): 908-13.
870. Lopez J, Richardson E, Tiozzo E, et al. The effect of exercise training on disease progression, fitness, quality of life, and mental health in people living with HIV on antiretroviral therapy: a systematic review. J Clin Transl Res 2015; 1(3): 129-39.
871. Lopez MD, Wise C. Acute ataxia in a 4-year-old boy: a case of Lyme disease neuroborreliosis. American Journal of Emergency Medicine 2008; 26(9): e5-1069.
872. López-Campos JL, Rodríguez DA, Quintana-Gallego E, Martínez-Llorens J, Carrasco Hernández L, Barreiro E. Ten Research Questions for Improving COPD Care in the Next Decade. Copd 2019; 16(5-6): 311-20.
873. López-Morales CA, Miranda-Hernández MP, Juárez-Bayardo LC, et al. Physicochemical and Biological Characterization of a Biosimilar Trastuzumab. BioMed Research International 2015; 2015: 1-10.
874. Lorenzoni PJ, Scola RH, Kay CS, Parolin SF, Werneck LC. Non-paraneoplastic Lambert-Eaton myasthenic syndrome: a brief review of 10 cases. Arq Neuropsiquiatr 2010; 68(6): 849-54.
875. Loukas A, McManus DP. Current status of vaccines for schistosomiasis. Clinical Microbiology Reviews 2008; 21(1): 225-42.
876. Loutan L, Chappuis F, Bovier P. Vaccines for travellers: Necessity and choice. Medecine et Hygiene 2001; 59(2348): 1192-8.
877. Lu J, Aljahani N, Molinari R. Case report: Pembrolizumab-associated psoriasis exacerbation. Journal of the Dermatology Nurses' Association 2020; 12(2).
878. Lu L, Hu Y, Wang C, Jiang F, Wu C. Methylation and Expression of the Exercise-Related TLR1 Gene Is Associated With Low Grade Glioma Prognosis and Outcome. Front Mol Biosci 2021; 8: 747933.
879. Lucas C, Macario F, Santos-Araujo C, et al. Time-dependent evolution of IgG antibody levels after first and second dose of mRNA-based SARS-CoV-2 vaccination in hemodialysis patients: a multicenter study. Nephrology, dialysis, transplantation : official publication of the European Dialysis and Transplant Association - European Renal Association 2021.
880. Lucchese A, Mittelman A, Lin MS, Kanduc D, Sinha AA. Epitope definition by proteomic similarity analysis: identification of the linear determinant of the anti-Dsg3 MAb 5H10. J Transl Med 2004; 2(1): 43.
881. Lundahl J, Denburg JA, O'Byrne PM, Liang H, Upham JW, Snider DP. Simplified quantitation of myeloid dendritic cells in peripheral blood using flow cytometry. Cytometry 2000; 40(1): 50-9.
882. Luo Z, Ma L, Zhang L, et al. Key differences in B cell activation patterns and immune correlates among treated HIV-infected patients versus healthy controls following influenza vaccination. Vaccine 2016; 34(16): 1945-55.
883. Luo Z, Martin L, Kilby M, et al. Influenza vaccine induces anti-nuclear and anti-double strand DNA IgG antibodies and their relates to levels of microbial translocation in antiretroviral-treated aviremic HIV-infected patients but not in healthy controls. Journal of Immunology 2016; 196(SUPPL. 1).
884. Luo Z, Ogunrinde E, Li M, et al. Increased influenza-specific antibody avidity in HIV-infected women compared with HIV-infected men on antiretroviral therapy. AIDS 2019; 33(1): 33-44.

885. Luo Z, Zhang L, Li Z, et al. Key differences in B cell activation patterns and immune correlates among treated HIV-infected patients versus healthy controls following influenza vaccination. Vaccine 2016; 34(16): 1945-55.
886. Lupia M, Cavallaro U, Sachsenmeier KF, Colombo N, Bianchi F. CD73: A new driver and a therapeutic target in ovarian cancer stem cells. Clinical Cancer Research 2019; 25(22).
887. Lutokhina YA, Blagova OV, Sedov VP, Nedostup AV, Zaydenov VA. Combination of chronic myocarditis and progressive coronary artery disease: Differential diagnosis and stepwise treatment. Russian Journal of Cardiology 2020; 25(11): 131-6.
888. Lv J, Lu X, Jiang XD, et al. [Prokaryotic expression, purification of human LINGO-1(aa76-319) and preparation of its polyclonal antibody]. Nan Fang Yi Ke Da Xue Xue Bao 2009; 29(11): 2175-8.
889. Lynn T, Rosati P, Santos GL, Endo PT. Sorting the healthy diet signal from the social media expert noise: Preliminary evidence from the healthy diet discourse on twitter. International Journal of Environmental Research and Public Health 2020; 17(22): 1-28.
890. Ma Z, Fan HJ, Lu CP. Molecular cloning and analysis of the UDP-Glucose Pyrophosphorylase in Streptococcus equi subsp. zooepidemicus. Mol Biol Rep 2011; 38(4): 2751-60.
891. Mabweazara SZ, Leach LL, Ley C, Smith M. A six week contextualised physical activity intervention for women living with HIV and AIDS of low socioeconomic status: a pilot study. AIDS care - psychological and socio-medical aspects of AIDS/HIV 2018; 30: 61-5.
892. Maccarone MC, Magro G, Solimene U, Scanu A, Masiero S. From in vitro research to real life studies: an extensive narrative review of the effects of balneotherapy on human immune response. Sport Sciences for Health 2021; 17(4): 817-35.
893. Macdonald JW, Dagless MD, McMartin DA, et al. Field observations on serological responses to vaccine strains of infectious bronchitis virus administered by coarse spray and via the drinking water. Avian Pathol 1982; 11(4): 537-46.
894. Machida I, Handa H, Yoshida Y, et al. Effects of physical therapy on cytokines and two color analysis- lymphocyte subsets in patients with cerebrovascular diseases. Journal of Medicine 1999; 30(1-2): 31-7.
895. MacIver A, Hollinger H, Carolan C. Tele-health interventions to support self-management in adults with rheumatoid arthritis: a systematic review. Rheumatol Int 2021; 41(8): 1399-418.
896. Mack C, Velentgas P, Franke K, Jablonski R, Parmenter L, Dreyer NA. Delivering actionable results for observational studies through rapid evaluation processes. Value in Health 2016; 19(3): A85.
897. Mackinnon LT. Exercise and immunology. Champaign, Ill.;: Human Kinetics Books; 1992.
898. Mackinnon LT. Advances in exercise immunology. Champaign, Ill.;: Human Kinetics; 1999.
899. Mackinnon LT, Chin LL. The roles of exercise-induced immune system disturbances in the pathology of heat stroke: The dual pathway model of heat stroke. Sports Medicine 2006; 36(1): 39-64.
900. Madhusoodanan M, Oomman A. Tropical spastic paraparesis in Kerala. Neurology India 2003; 51(4): 493-6.
901. Magrath I. The epidemiology and pathogenesis of Burkitt lymphoma. British Journal of Haematology 2015; 171(SUPPL. 1): 1.
902. Mahendran R, Feng L, Fam J, et al. Mindful awareness practice for the prevention of dementia: a randomised controlled trial. Annals of the academy of medicine singapore 2014; 43(10): S66-S7.
903. Mahmood Z, Lundberg A, Jonasson L, Back M. Enhanced interleukin-6 expression and impaired cortisol response in patients with coronary artery disease. European Heart Journal 2018; 39(Supplement 1): 538.

904. Maillard E, Sigrist S, Meyer L, Jeandidier N. Smart insulins and bioartificial pancreas in T1D: actors for tomorrow, really? Medecine des Maladies Metaboliques 2021; 15(3 Supplement): 3S65-3S75.
905. Maillard S, Pilkington C. Anti SRP+Ve myositis in childhood. Presentation and physiotherapy treatment of this rare childhood myositis. Arthritis and Rheumatology 2015; 67(SUPPL. 10).
906. Maisch B, Selmayer N, Brugger E, et al. Cardiac sarcoidosis - Clinical and immunoserologic studies. European Heart Journal 1987; 8(SUPPL. J): 63-71.
907. Majid U, Hussain SAS, Wasim A, Farhana N, Saadat P. A Systematic Map of Non-Clinical Evidence Syntheses Published Globally on COVID-19. Disaster Med Public Health Prep 2021: 1-6.
908. Makaryus MN, Hassid B, Makaryus AN. Falsely elevated cardiac troponin I levels. Clinical Cardiology 2007; 30(2): 92-4.
909. Makela M. Milk and wheat allergy, and celiac disease. Clinical and Translational Allergy 2011; 1(SUPPL. 1).
910. Makin RD, Argyle D, Nagasaka Y, et al. Voluntary exercise attenuates choroidal neovascularization in mice. Investigative Ophthalmology and Visual Science 2020; 61(7).
911. Malliaras K, Li TS, Luthringer D, et al. Safety and efficacy of allogeneic cell therapy in infarcted rats transplanted with mismatched cardiosphere-derived cells. Circulation 2012; 125(1): 100-12.
912. Mammucari M, Paolucci T, Russo D, et al. A Call to Action by the Italian Mesotherapy Society on Scientific Research. Drug Des Devel Ther 2021; 15: 3041-7.
913. Manfredi R, Calza L, Chiodo F. Prolonged statin administration does not act on the cell-mediated immunity of HIV-infected dyslipidemic patients treated with a steady and effective highly active antiretroviral therapy. A two-year prospective study of statin versus fibrate administration. Journal of biological regulators and homeostatic agents 2006; 20(1-2): 1-9.
914. Manganaro L, Johnson J, Krogan N, et al. Multidimensional profiling of HIV-infected human CD4 T memory stem cells. Topics in Antiviral Medicine 2016; 24(E-1): 97.
915. Manjarrez-Orduno N, Quach TD, Sanz I. B cells and immunological tolerance. Journal of Investigative Dermatology 2009; 129(2): 278-88.
916. Mansoori A, Ramezani Ahmadi A, Bahreini M, Rayyani E. The effect of glutamine supplementation on athletic performance, body composition, and immune function: A systematic review and a meta-analysis of clinical trials. Clinical Nutrition 2019; 38(3): 1076-91.
917. Manyazewal T, Mekonnen A, Demelew T, et al. Improving immunization capacity in Ethiopia through continuous quality improvement interventions: a prospective quasi-experimental study. Infect Dis Poverty 2018; 7(1): 119.
918. Marchal S, Janicot J, Salicis J, et al. Quick-Wee versus bladder stimulation to collect midstream urine from precontinent infants under 1 year of age: a study protocol for a randomised controlled trial (ES.Stimquick.U). BMJ Open 2021; 11(9): e046324.

919. Marco AM-G, Fernández-García B, Alonso-Arias R, Rodriguez-Alonso M, García Francisco MS, López-Larrea C. EFFECTS OF MAINTAINED INTENSE EXERCISE THROUGHOUT THE LIFE ON THE ADAPTIVE IMMUNE RESPONSE IN ELDERLY AND YOUNG ATHLETES. British Journal of Sports Medicine 2013; 47(10): 9-.
920. Marcos A, Nova E, Montero A. Changes in the immune system are conditioned by nutrition. Eur J Clin Nutr 2003; 57 Suppl 1: S66-9.
921. Markova EV, Knyazheva MA. Immune cells as a potential therapeutic agent in the treatment of depression. Medical Immunology (Russia) 2021; 23(4): 699-704.
922. Marnocha SK. Chronic stressors increased susceptibility to colds [commentary on Cohen S, Frank E, Doyle WJ, et al. Types of stressors that increase susceptibility to the common cold in healthy adults. HEALTH PSYCHOLOGY 1998 May;17:214-23]. Evidence Based Nursing 1999: 54-.

923. Marodon G, Burlion A, Salomon B, et al. Anti-ICOS immunotherapy in humanized mice. Immunology 2012; 137(SUPPL. 1): 184.
924. Marques Zecchin-Oliveira A, Domiciano RAM, Barbosa Ribeiro V, Fuini Puggina E. TRAINING ROUTINE AND MOTIVATION AMONG CROSSFIT® PARTICIPANTS IN BRAZIL AND PORTUGAL DURING THE COVID-19 PANDEMIC: AN OBSERVATIONAL STUDY. / Rotina de treinamento e motivação entre participantes de crossfit® no brasil e em portugal durante a pandêmica covid-19: um estudo observacional. Revista Brasileira de Prescrição e Fisiologia do Exercício 2020; 14(94): 907-16.
925. Marshall GD. Psychological stress, immunity, and asthma: developing a paradigm for effective therapy and prevention. Current Opinion in Behavioral Sciences 2019; 28: 14-9.
926. Marsland AL, Cohen S, Rabin BS, Manuck SB. Trait positive affect and antibody response to hepatitis B vaccination. Brain, Behavior, and Immunity 2006; 20(3): 261-9.
927. Martin L. Orofacial herpes and other localizations (genital herpes and neonatal herpes excluded). Annales de Dermatologie et de Venereologie 2002; 129(4 II): 494-506.
928. Martin Munoz MF. Efficacy of immunotherapy in the treatment of asthma. Allergologia et Immunopathologia 2004; 32(3): 133-41.
929. MartÍNez AC, FernÁNdez-LÁZaro D. New trends in biological aids to recovery after exercise: Immunomodulators. Journal of Human Sport & Exercise 2018; 13(1): 116-28.
930. Martinez AJ, Janitschke K. Acanthamoeba, an opportunistic microorganism: A review. Infection 1985; 13(6): 251-6.
931. Martinez-Lavin M. Dorsal root ganglia: fibromyalgia pain factory? Clinical Rheumatology 2021; 40(2): 783-7.
932. Martins AT, Ledur GR, Carvalho A, Queiroga LB, de Castro Beck CA, Trindade-Gerardi AB. Immune-Mediated Hemolytic Anemia in a Bitch Triggered by Drugs. Acta Scientiae Veterinariae 2021; 49: 733.
933. Martos-Moreno GA, Gonzalez-Vicent M, Sebastian E, Argente J. Successful immune tolerance induction in the first case of neutralizing antibody mediated loss of efficacy of asfotase alfa treatment in hypophosphatasia. Hormone Research in Paediatrics 2018; 90(Supplement 1): 61.
934. Marzin T, Lorkowski G, Reule C, et al. Effects of a systemic enzyme therapy in healthy active adults after exhaustive eccentric exercise: a randomised, two-stage, double-blinded, placebo-controlled trial. BMJ Open Sport Exerc Med 2016; 2(1): e000191.

935. Mase WA, Jones SD, Bickford B, Thomas CL, Bisesi M. After-action review of the 2009-10 H1N1 Influenza Outbreak Response: Ohio's Public Health System's performance. Journal of emergency management (Weston, Mass) 2017; 15(5): 325-34.
936. Masood F, Wlodkowski P, Chaus A. ALL PAIN AND NO GAIN: STATIN IMMUNE MEDIATED NECROTIZING MYOPATHY. Journal of the American College of Cardiology 2020; 75(11): 2672.
937. Mathias M, Khair K, Liesner R. Immune tolerance with plasma derived FVIII/VWD concentrate in boys with severe haemophilia a and resistant inhibitors. Haematologica 2011; 96(SUPPL. 2): 298.
938. Matricardi PM, Dal Negro RW, Nisini R. The first, holistic immunological model of COVID-19: Implications for prevention, diagnosis, and public health measures. Pediatric Allergy and Immunology 2020; 31(5): 454-70.
939. Mauel C, Merten C, Bewermeyer H. [A case of vaccination-induced polio]. Med Klin (Munich) 1998; 93(1): 39-42.
940. Mayer F, Bonaventura K, Cassel M, et al. Medical results of preparticipation examination in adolescent athletes. British journal of sports medicine 2012; 46(7): 524-30.
941. McCarty KS, Jr., Sasso R, Budwit D, Georgiade GS, Seigler HF. Immunoglobulin localization in the normal human mammary gland: variation with the menstrual cycle. Am J Pathol 1982; 107(3): 322-6.

942. McCormack R, Lee K. Effectiveness of an Influenza Immunization Program for the Canadian National Team at a Winter Multisport Games. (Abstract). Clinical Journal of Sport Medicine 2007; 17(2): 166-.
943. McCormick F, Cole BJ, Nwachukwu B, Harris JD, Adkisson Iv HD, Farr J. Treatment of Focal Cartilage Defects With a Juvenile Allogeneic 3-Dimensional Articular Cartilage Graft. Operative Techniques in Sports Medicine 2013; 21(2): 95-9.
944. McCulley M, Huson J, Menteer JD, Ward S, Ghuman A. Rapid recovery from respiratory failure after cessation of sirolimus in a post-transplant adolescent. Critical Care Medicine 2013; 41(12 SUPPL. 1): A290.
945. McFarlin BK, O'Connor DP, Simpson RJ, et al. Senescent phenotypes and telomere lengths of peripheral blood T-cells mobilized by acute exercise in humans. Exercise Immunology Review 2010; 16: 40-55.
946. McFarlin BK, Venable AS, Henning AL, Carpenter KC, Ogenstad S. Oral supplementation with Baker's yeast beta glucan is associated with altered monocytes, T cells and cytokines following a bout of strenuous exercise. Frontiers in Physiology 2017; 8(OCT): 786.
947. McFarlin BK, Venable AS, Henning AL, Williams RR, Prado EA. Assessment of Granulocyte Subset Activation: New Information from Image-Based Flow Cytometry. Methods Mol Biol 2016; 1389: 177-85.
948. McGing JJ, Radford SJ, Francis ST, Serres S, Greenhaff PL, Moran GW. Review article: The aetiology of fatigue in inflammatory bowel disease and potential therapeutic management strategies. Aliment Pharmacol Ther 2021; 54(4): 368-87.
949. McIntyre RS, Filteau MJ, Martin L, et al. Treatment-resistant depression: definitions, review of the evidence, and algorithmic approach. J Affect Disord 2014; 156: 1-7.
950. McKenna SG. What effect does exercise have on sleep in RMD? Annals of the Rheumatic Diseases 2018; 77(Supplement 2): 45-6.
951. McKune AJ, Smith LL, Semple SJ, Wadee AA. Influence of ultra-endurance exercise on immunoglobulin isotypes and subclasses. British Journal of Sports Medicine 2005; 39(9): 665-70.
952. McLaughlin VV, Archer SL, Badesch DB, et al. ACCF/AHA 2009 Expert Consensus Document on Pulmonary Hypertension. A Report of the American College of Cardiology Foundation Task Force on Expert Consensus Documents and the American Heart Association Developed in Collaboration With the American College of Chest Physicians; American Thoracic Society, Inc.; and the Pulmonary Hypertension Association. Journal of the American College of Cardiology 2009; 53(17): 1573-619.
953. McManus DP, Loukas A. Current status of vaccines for schistosomiasis. Clin Microbiol Rev 2008; 21(1): 225-42.
954. McMillan CLD, Young PR, Watterson D, Chappell KJ. The next generation of influenza vaccines: Towards a universal solution. Vaccines 2021; 9(1): 1-20.
955. McSharry C, Lynch PP, Banham SW, Boyd G. Seasonal variation of antibody levels among pigeon fanciers. Clinical Allergy 1983; 13(4): 293-9.
956. Meeus M, van Cauwenbergh D, Nijs J, et al. Pain in patients with chronic fatigue syndrome: Time for specific pain treatment? Pain Physician 2012; 15(5): E677-E86.
957. Mehal WZ. The gordian knot of dysbiosis, obesity and nafld. Nature Reviews Gastroenterology and Hepatology 2013; 10(11): 637-44.
958. Mehndiratta P, Preston DC, Mehta S, Manjila SV, Kammer GM, Cohen ML. Isolated necrotizing myopathy associated with ANTI-PL12 antibody. Muscle and Nerve 2012; 46(2): 282-6.
959. Mehta K, Tayama T, Marsteller D, et al. Population pharmacokinetic-pharmacodynamic analysis of KHK2455 in patients with locally advanced or metastatic solid tumors. Cancer Research 2021; 81(13 SUPPL).
960. Meijboom M, Rozenbaum M, Benedictus A, et al. Cost utility of infant vaccination against respiratory syncytial virus infection in the Netherlands. Value in Health 2011; 14(7): A279-A80.

961. Meijboom MJ, Rozenbaum MH, Benedictus A, et al. Cost-effectiveness of potential infant vaccination against respiratory syncytial virus infection in The Netherlands. Vaccine 2012; 30(31): 4691-700.
962. Mekonnen A, Demelew T, Mengestu S, et al. Improving immunization capacity in Ethiopia through continuous quality improvement interventions: A prospective quasi-experimental study 11 Medical and Health Sciences 1117 Public Health and Health Services. Infectious Diseases of Poverty 2018; 7(1): 119.
963. Melnikov IY, Zhurilo OV, Komarova IA, Sashenkov SL. Humoral factors of immunity depending on the skill levels of athletes. Gazzetta Medica Italiana Archivio per le Scienze Mediche 2018; 177(3 Supplement 1-3): 56-61.
964. Mendell JR, Rodino-Klapac L, Sahenk Z, et al. Eteplirsen, a phosphorodiamidate morpholino oligomer (PMO) for the treatment of duchenne muscular dystrophy (DMD): 168 week update on six-minute walk test (6MWT), pulmonary function testing (PFT), and safety. Molecular Therapy 2015; 23(SUPPL. 1): S16.
965. Meng H, Ba Z, Lee Y, et al. Consumption of Bifidobacterium animalis subsp. lactis BB-12 in yogurt reduced expression of TLR-2 on peripheral blood-derived monocytes and pro-inflammatory cytokine secretion in young adults. European journal of nutrition 2017; 56(2): 649-61.
966. Menge AC, Christman GM, Ohl DA, Naz RK. Fertilization antigen-1 removes antisperm autoantibodies from spermatozoa of infertile men and results in increased rates of acrosome reaction. Fertility and Sterility 1999; 71(2): 256-60.
967. Mercer C, Datta J, Weatherburn P, Reid D, Wayal S, Hughes G. Which sexually transmitted infections do gay and bisexual men find most scary and why? a qualitative study. Sexually Transmitted Infections 2017; 93(Supplement 2): A224.
968. Mercuriali F. The role of recombinant human erythropoietin (rHuEPO) in surgical oncology. Tumori 1997; 83(4 SUPPL. 2): S16-S9.
969. Meremikwu MM. Sickle cell disease. BMJ clinical evidence 2009; 2009.
970. Meremikwu MM, Okomo U. Sickle cell disease. BMJ clinical evidence 2011; 2011.
971. Merten C, Bewermeyer H, Mauel C. A case of polio by vaccination. Medizinische Klinik 1998; 93(1): 39-42.
972. Michalickova D, Minic R, Kotur-Stevuljevic J, et al. CHANGES IN PARAMETERS OF OXIDATIVE STRESS, IMMUNITY, AND BEHAVIOR IN ENDURANCE ATHLETES DURING A PREPARATION PERIOD IN WINTER. Journal of Strength & Conditioning Research 2020; 34(10): 2965-73.
973. Michalickova DM, Kostic-Vucicevic MM, Vukasinovic-Vesic MD, et al. LACTOBACILLUS HELVETICUS LAFTI L10 SUPPLEMENTATION MODULATES MUCOSAL AND HUMORAL IMMUNITY IN ELITE ATHLETES: A RANDOMIZED, DOUBLE-BLIND, PLACEBO-CONTROLLED TRIAL. Journal of Strength & Conditioning Research (Lippincott Williams & Wilkins) 2017; 31(1): 62-70.
974. Michaud DS, Izard J, Rubin Z, et al. Lifestyle, dietary factors, and antibody levels to oral bacteria in cancer-free participants of a European cohort study. Cancer Causes Control 2013; 24(11): 1901-9.
975. Mickleborough TD, Lindley MR, Montgomery GS. Effect of fish oil-derived omega-3 polyunsaturated Fatty Acid supplementation on exercise-induced bronchoconstriction and immune function in athletes. Phys Sportsmed 2008; 36(1): 11-7.
976. Mika A, Fleshner M. Early-life exercise may promote lasting brain and metabolic health through gut bacterial metabolites. Immunology and Cell Biology 2016; 94(2): 151-7.
977. Mikkelsen K, Stojanovska L, Polenakovic M, Bosevski M, Apostolopoulos V. Exercise and mental health. Maturitas 2017; 106: 48-56.
978. Milecki P, Kwiatkowska-Borowczyk E, Leporowska E, Hojan K. Physical exercises, inflammation, fatigue and aerobic fitness in prostate cancer patients undergoing antiandrogen-and radiotherapy. Archives of Physical Medicine and Rehabilitation 2015; 96(10): e5.

979. Millar NL, Silbernagel KG, Thorborg K, et al. Tendinopathy. Nat Rev Dis Primers 2021; 7(1): 1.
980. Miller GE, Cohen S, Pressman S, et al. Psychological stress and antibody response to influenza vaccination: when is the critical period for stress, and how does it get inside the body? Psychosomatic Medicine 2004; 66(2): 215-23.
981. Miller MK, Dowd D, Humiston SG, et al. A brief intervention to reduce adolescent sexual risk behaviors: Feasibility and impact. Journal of Adolescent Health 2014; 54(2 SUPPL. 1): S11.
982. Mills PJ, Ziegler MG, Edwards KM. The potential anti-inflammatory benefits of improving physical fitness in hypertension. Journal of Hypertension 2007; 25(8): 1533-42.
983. Ming-Hua C, Bao-Hua Z, Lei Y. Mechanisms of Anorexia Cancer Cachexia Syndrome and Potential Benefits of Traditional Medicine and Natural Herbs. Curr Pharm Biotechnol 2016; 17(13): 1147-52.
984. Mironova M, Stephan E, Durazo-Arvizu R, Virella G, Lopes-Virella MF. Role of Simvastatin as an Immunomodulator in Type 2 Diabetes. Diabetes Care 2004; 27(4): 908-13.
985. Mitić R. Bioenergijska priprava v športu – optimizacija danih sposobnosti. / Bioenergy conditioning in sports - optimization of given abilities. Sport: Revija Za Teoreticna in Prakticna Vprasanja Sporta 2021; 69(3/4): 10-4.
986. Mizooka M, Ishikawa S. Prevalence of chlamydia pneumoniae in Japanese rural districts; association of smoking and physical activity with Chlamydia pneumoniae seropositivity. Intern Med 2003; 42(10): 960-6.
987. Moghe A, Kaldas H. The hook effect: Don't take the bait! Journal of General Internal Medicine 2015; 30(SUPPL. 2): S463.
988. Mohajeri MH, Leuba G. Prevention of age-associated dementia. Brain Research Bulletin 2009; 80(4-5): 315-25.
989. Mohammad SS, Ramanathan S, Brilot F, Dale RC. Autoantibody-associated movement disorders. Neuropediatrics 2013; 44(6): 336-45.
990. Moiola L, D'Angelo A, Martinelli V, Comi G, Pisa M. First reported case of aquired hemophilia A (AHA) as secondary autoimmune disease following alemtuzumab treatment in multiple sclerosis. Multiple Sclerosis Journal 2017; 23(3 Supplement 1): 379-80.
991. Moises Caetano Bottini DA, Vieira de Assis P, Rippel Salgado P, Domingos JA, Christofoletti G, Gomes de Souza Pegorare AB. Treinamento do assoalho pélvico com ou sem eletroterapia no tratamento dos sintomas da hiperatividade do detrusor em mulheres com esclerose múltipla e mielopatia associada ao HTLV-I (HAM/TSP): um ensaio clínico randomizado. Fisioterapia Brasil 2019; 20(4): 500-8.
992. Monge S, Hahné SJ, de Melker HE, Sanders EA, van der Ende A, Knol MJ. Effectiveness of the DTPa-HBV-IPV/Hib vaccine against invasive Haemophilus influenzae type b disease in the Netherlands (2003-16): a case-control study. Lancet Infect Dis 2018; 18(7): 749-57.
993. Mongini PK, Inman JK, Han H, Fattah RJ, Abramson SB, Attur M. APRIL and BAFF promote increased viability of replicating human B2 cells via mechanism involving cyclooxygenase 2. J Immunol 2006; 176(11): 6736-51.
994. Monteiro MP. Basic considerations, patient selection and indication for treatment. CardioVascular and Interventional Radiology 2015; 38(3 SUPPL. 1): S40-S1.
995. Moore MAS. Cytokine and chemokine networks influencing stem cell proliferation, differentiation, and marrow homing. Journal of cellular biochemistry Supplement 2002; 38: 29-38.
996. Mooren FC, Blöming D, Lechtermann A, Lerch MM, Völker K. Lymphocyte apoptosis after exhaustive and moderate exercise. J Appl Physiol (1985) 2002; 93(1): 147-53.
997. Mooren FC, Völker K, Klocke R, Nikol S, Waltenberger J, Krüger K. Exercise delays neutrophil apoptosis by a G-CSF-dependent mechanism. J Appl Physiol (1985) 2012; 113(7): 1082-90.
998. Moreira A, Kekkonen RA, Delgado L, Fonseca J, Korpela R, Haahtela T. Nutritional modulation of exercise-induced immunodepression in athletes: a systematic review and meta-analysis. Eur J Clin Nutr 2007; 61(4): 443-60.

999. Morimoto K, Sekiguchi K, Watanabe S, Noda Y, Matsumoto R. O2-093 Treatable pes equinus in a case of chronic inflammatory demyelinating polyradiculopathy (CIDP) with anti-neurofascin-155 antibody. Clinical Neurophysiology 2020; 131(10): e264.
1000. Morita Y. Classification of bronchial asthma based on immunological mechanisms. Nihon Kyobu Shikkan Gakkai zasshi 1995; 33 Suppl: 100-3.
1001. Morland K, Reich D, Bordowitz R. The use of an electronic medical record to improve documentation and treatment of obesity. Family Medicine 2007; 39(4): 274-9.
1002. Moro-Garcia MA, Alonso-Arias R, Lopez-Larrea C, Fernandez-Garcia B. Effects of prolonged intense exercise on the adaptive immune response in elderly and young athletes. Immunology 2012; 137(SUPPL. 1): 319.
1003. Morrison BN, McKinney J, Isserow S, et al. Assessment of cardiovascular risk and preparticipation screening protocols in masters athletes: the Masters Athlete Screening Study (MASS): a cross-sectional study. BMJ Open Sport Exerc Med 2018; 4(1): e000370.
1004. Morrison BN, Warburton DER, Isserow S, et al. Masters Athlete Cardiac Health (MACH) study: Insights into pre-participation screening and cardiovascular risk in masters athletes. European Journal of Preventive Cardiology 2017; 24(1 Supplement 1): S15.
1005. Morrow-Sutton MA, Creer M, Shike H, et al. Use of therapeutic plasma exchange in the treatment of acute flaccid myelitis in a thirteen year old male with recent fever and upper respiratory illness. Journal of Clinical Apheresis 2019; 34(2): 157.
1006. Moudgal NR, Jeyakumar M, Krishnamurthy HN, Sridhar S, Krishnamurthy H, Martin F. Development of male contraceptive vaccine--a perspective. Hum Reprod Update 1997; 3(4): 335-46.
1007. Mourgues C, Blanquet M, Gerbaud L, Soubrier M, Dougados M. Economic analysis of a nurse-led programme for comorbidities management of rheumatoid arthritis patients. Joint, bone, spine 2018; 85(5): 573-6.
1008. Mu W, Song Y-L, Zhang S, Zhang L, Fu M, Shang H-C. Cordyceps sinensis for chronic obstructive pulmonary diseases: A systematic review. Chinese Journal of Evidence-Based Medicine 2013; 13(11): 1373-81.
1009. Muelas MMN, Marti P, Vilchez JJ, et al. Statin-induced myopathies: Beyond immuno-mediated necrotizing myopathies. European Journal of Neurology 2018; 25(Supplement 2): 86.
1010. Muhle C, Zenker M, Chuzhanova N, Schneider H. Recurrent inversion with concomitant deletion and insertion events in the coagulation factor VIII gene suggests a new mechanism for X-chromosomal rearrangements causing hemophilia A. Human mutation 2007; 28(10): 1045.
1011. Muley SA, Kelkar P, Parry GJ. Treatment of chronic inflammatory demyelinating polyneuropathy with pulsed oral steroids. Arch Neurol 2008; 65(11): 1460-4.
1012. Muller L, Pawelec G. Aging and immunity - Impact of behavioral intervention. Brain, Behavior, and Immunity 2014; 39: 8-22.
1013. Munoz Gonzalez A, Contreras Chicote A, Vales Montero M, et al. Antiglycine receptor antibodies and rapidly progressive corticobasal syndrome. Movement Disorders 2018; 33(Supplement 2): S402-S3.
1014. Murad H, Assaad JM, Al-Shemali R, Abbady AQ. Exploiting nanobodies in the detection and quantification of human growth hormone via phage-sandwich enzyme-linked immunosorbent assay. Frontiers in Endocrinology 2017; 8(MAY): 115.
1015. Murakami M, Yasutaka T, Onishi M, et al. Living with COVID-19: mass gatherings and minimizing risk. QJM : monthly journal of the Association of Physicians 2021; 114(7): 437-9.
1016. Murphree CR, Olson SR, DeLoughery TG, Shatzel JJ. When to consider targeted therapies in thrombotic microangiopathies in the modern era: walking the tightrope between cost, safety, and efficacy. Journal of Thrombosis and Thrombolysis 2020; 49(4): 602-5.
1017. Murray J. The effects of exercise on humoral immunity in BALB/c mice. Eugene, Ore.;: Microform Publications, Int'l Inst for Sport & Human Performance, University of Oregon; 1997.

1018. Murri MB, Folesani F, Zerbinati L, et al. Physical activity promotes health and reduces cardiovascular mortality in depressed populations: A literature overview. International Journal of Environmental Research and Public Health 2020; 17(15): 1-18.
1019. Musumeci O, Barca E, Toscano A. Therapeutic approaches in the late onset form of GSD II. Acta Myologica 2011; 30(DECEMBER): 206-7.
1020. Mylonakis E, Dickinson BP, Rich JD. Influenza vaccination for athletes: facts and controversies. American Journal of Medicine & Sports 2003; 5(1): 67-71.
1021. Nadarajan VS. Basic aspects, applications and platforms currently available for molecular blood grouping of donor and patient populations. Vox Sanguinis 2017; 112(Supplement 2): 32.
1022. Naimimohasses S, Norris S, O'Gorman P, et al. Significant reductions in intrahepatic Mucosal Associated Invariant T cells with increased terminal activation marker expression amongst NAFLD patients following a 12 week aerobic exercise program: a paired liver biopsy study. Journal of Hepatology 2019; 70(1): e801.
1023. Nair H, Lau E, Brooks W, et al. An evaluation of the emerging vaccines against influenza in children. BMC Public Health 2013; 13 Suppl 3(Suppl 3): S14.
1024. Nair H, Verma VR, Theodoratou E, et al. An evaluation of the emerging interventions against Respiratory Syncytial Virus (RSV)-associated acute lower respiratory infections in children. BMC Public Health 2011; 11(Suppl 3): S30-S.
1025. Nakagawa N, Hosokawa K, Espinoza L, et al. Relatively low sensitivity of CD109(-) hematopoietic stem/progenitor cells (HSPCs) to TGF-beta: A possible mechanism responsible for the preferential commitment of piga mutant HSPCs in immune-mediated bone marrow failure. Blood 2016; 128(22).
1026. Nalepa P, Pasowicz M, Moczulski Z, Zietek A, Bosak J, Stankiewicz Z. [Mounier-Kuhn syndrome (tracheobronchomegaly)]. Pol Merkur Lekarski 2005; 19(109): 71-4.
1027. Namazova-Baranova LS, Efendieva KE, Sadeqi N. New evidence on the evolution of the COVID-19 pandemic: Literature review. Pediatricheskaya Farmakologiya 2021; 18(4): 314-9.
1028. Nameni F. Effect of 2 months endurance training on immune cells and humoral. Journal of Science and Medicine in Sport 2011; 14(SUPPL. 1): e90.
1029. Nasolodin VV, Rusin VI, Gladkin IP. Sravnitel'naia effektivnost' mikroelementnykh dobavok k pitaniiu sportsmenov pri trenirovke v raznoe vremia goda. / Comparative effectiveness of microelements added to the diet of athletes during training in different seasons. Voprosy Pitaniia/Problems of Nutrition 1984; (6): 33-8.
1030. Nasrullayeva G, Mammadova V. Family case of ataxiatelangiectasiya. Journal of Clinical Immunology 2012; 32(SUPPL. 1): S398-S9.
1031. Natalucci V, Virgili E, Calcagnoli F, et al. Cancer Related Anemia: An Integrated Multitarget Approach and Lifestyle Interventions. Nutrients 2021; 13(2).
1032. Nathwani N, Bell JA, Cherepanov D, et al. Patient perspectives on treatment experience and health-related quality of life in patients with relapsed/refractory multiple myeloma. Blood 2020; 136(SUPPL 1): 29-30.
1033. Nattagh-Eshtivani E, Jokar M, Tabesh H, et al. The effect of propolis supplementation on inflammatory factors and oxidative status in women with rheumatoid arthritis: Design and research protocol of a double-blind, randomized controlled. Contemp Clin Trials Commun 2021; 23: 100807.
1034. Navalta J, McFarlin B, Simpson R, et al. Finger-stick blood sampling methodology for the determination of exercise-induced lymphocyte apoptosis. Journal of visualized experiments : JoVE 2011; (48).
1035. Nazi I, Toltl LJ, Smith JW, et al. Antibody binding to megakaryocytes in vivo in patients with immune thrombocytopenia. European Journal of Haematology 2015; 95(6): 532-7.
1036. Nct. Safety and Efficacy Study of Oral Fampridine-SR in Patients With Multiple Sclerosis. https://clinicaltrialsgov/show/NCT00053417 2003.

1037. Nct. Subcutaneous Immunoglobulin Treatment for Multifocal Motor Neuropathy. https://clinicaltrialsgov/show/NCT00268788 2005.
1038. Nct. Beryllium Infliximab Study: clinical Interventional Trial. https://clinicaltrialsgov/show/NCT00111917 2005.
1039. Nct. Motivating HIV+ Women: risk Reduction and ART Adherence. https://clinicaltrialsgov/show/NCT00253045 2005.
1040. Nct. Valganciclovir (Valcyte) for Chronic Fatigue Syndrome Patients Who Have Elevated Antibody Titers Against Human Herpes Virus 6 (HHV-6)and Epstein-Barr Virus (EBV). https://clinicaltrialsgov/show/NCT00478465 2007.
1041. Nct. The Effect of Vitamin D and Calcium on Bone in Pediatric HIV. https://clinicaltrialsgov/show/NCT00724178 2008.
1042. Nct. Efficacy of Lipid-Based Nutrient Supplements (LNS) for Pregnant and Lactating Women and Their Infants. https://clinicaltrialsgov/show/NCT00970866 2009.
1043. Nct. Impact of Aerobic Exercise on Asthma Morbidity. https://clinicaltrialsgov/show/NCT00953342 2009.
1044. Nct. T Cell Vaccination in Patients With Progressive Multiple Sclerosis. https://clinicaltrialsgov/show/NCT01448252 2011.
1045. Nct. Study to Assess Safety, Tolerability and MTD of a Central Pattern Generator-activating Tritherapy (SPINALON) in Patients With Chronic Spinal Cord Injury. https://clinicaltrialsgov/show/NCT01484184 2011.
1046. Nct. University of Wisconsin Meditation & Exercise Cold Study. https://clinicaltrialsgov/show/NCT01654289 2012.
1047. Nct. Can Shoulder Arthroscopy Work. https://clinicaltrialsgov/show/NCT01623011 2012.
1048. Nct. Polyphenols, Exercise, and Metabolomics. https://clinicaltrialsgov/show/NCT01775384 2013.
1049. Nct. Effects of DHA on Pro-resolving Anti-inflammatory Mediators in Obese Patients Undergoing Weight-loss Treatment. https://clinicaltrialsgov/show/NCT01865448 2013.
1050. Nct. Enhanced Natural Killer Cell Activity and RBAC. https://clinicaltrialsgov/show/NCT02019628 2013.
1051. Nct. Study of the Efficacy and Safety of Immune Globulin Intravenous (Human) Flebogamma® 5% DIF in Patients With Post-polio Syndrome. https://clinicaltrialsgov/show/NCT02176863 2014.
1052. Nct. The Effectiveness of Autonomic Nervous System, Sleep Quality, and Immune Regulation for Shift Nurses Practicing Alternative Nostril Breathing. https://clinicaltrialsgov/show/NCT02242708 2014.
1053. Nct. Benefits and Tolerance of Exercise in Patients With Generalized and Stabilized Myasthenia Gravis. https://clinicaltrialsgov/show/NCT02066519 2014.
1054. Nct. Thymosin-α1 in Cancer-Related Fatigue. https://clinicaltrialsgov/show/NCT02127268 2014.
1055. Nct. Botswana Vitamin D Supplementation Study in HIV/AIDS. https://clinicaltrialsgov/show/NCT02189902 2014.
1056. Nct. Yogic Breathing Changes Salivary Components. https://clinicaltrialsgov/show/NCT02108769 2014.
1057. Nct. Effectiveness of DIM Supplements to Increase 2-OHE1/16 Ratio. https://clinicaltrialsgov/show/NCT02525159 2015.
1058. Nct. the Evaluation of Four Non-operative Treatments for Degenerative Lumbar Spinal Stenosis. https://clinicaltrialsgov/show/NCT02592642 2015.
1059. Nct. Clinical Trial to Evaluate the Potential Efficacy and Safety of Human Umbilical Cord Blood and Plasma. https://clinicaltrialsgov/show/NCT02418013 2015.

1060. Nct. Acute Intermittent Hypoxia and Body Weight Supported Treadmill Training for Incomplete Spinal Cord Injury Patients. https://clinicaltrialsgov/show/NCT02441179 2015.
1061. Nct. Acceptance and Commitment Therapy (ACT) in Inflammatory Bowel Disease. https://clinicaltrialsgov/show/NCT02350920 2015.
1062. Nct. A Sleep Program to Improve Sleep Quality in People With HIV. https://clinicaltrialsgov/show/NCT02571595 2015.
1063. Nct. Do NSAIDS or Executing Exercise Decrease Local Erythema, Site Swelling & Pain After INoculation. https://clinicaltrialsgov/show/NCT02807623 2016.
1064. Nct. The MATCH Study: mindfulness And Tai Chi for Cancer Health. https://clinicaltrialsgov/show/NCT02801123 2016.
1065. Nct. Therapeutic Exercise in Cancer-Related Fatigue in Women After Breast Cancer Treatment. https://clinicaltrialsgov/show/NCT02828189 2016.
1066. Nct. Aspirin and Renal Disease Progression in Patients With Type 2 Diabetes. https://clinicaltrialsgov/show/NCT02895113 2016.
1067. Nct. Mood and Influenza Vaccine Response: a Feasibility Trial. https://clinicaltrialsgov/show/NCT03144518 2017.
1068. Nct. An RCT Promoting HPV Vaccination Among Chinese MSM. https://clinicaltrialsgov/show/NCT03286907 2017.
1069. Nct. Reducing Sedentary Time in Rheumatoid Arthritis: the Take a STAND for Health Study. https://clinicaltrialsgov/show/NCT03186924 2017.
1070. Nct. The Impact of Exercise on Stress, Fatigue, and Quality of Life in Individuals With Primary Immunodeficiency Disease. https://clinicaltrialsgov/show/NCT03211689 2017.
1071. Nct. Watermelon Focused Dietary Inflammatory Index Intervention. https://clinicaltrialsgov/show/NCT03158740 2017.
1072. Nct. Aerobic Exercise and Tai-chi Interventions for Improving Survival in Lung Cancer Patients. https://clinicaltrialsgov/show/NCT03482323 2018.
1073. Nct. Effects of Exercise on Young Adult Women With ACEs: an Integrative Pilot Study. https://clinicaltrialsgov/show/NCT03521401 2018.
1074. Nct. Flu and Mood in Older Adults. https://clinicaltrialsgov/show/NCT03956329 2019.
1075. Nct. A Study Investigating the Safety and Efficacy of Bio-Active Silver Hydrosol™ in Providing Immune Support. https://clinicaltrialsgov/show/NCT04144673 2019.
1076. Nct. Semaglutide's Efficacy in Achieving Weight Loss for Those With HIV. https://clinicaltrialsgov/show/NCT04174755 2019.
1077. Nct. Using a Humanoid Robot to Distract Children With Cancer or Chronic Immune Deficiency Undergoing Painful Procedures. https://clinicaltrialsgov/show/NCT04003701 2019.
1078. Nct. Improving Survival in Lung Cancer Patients: a Trial of Aerobic Exercise and Tai-chi. https://clinicaltrialsgov/show/NCT04119778 2019.
1079. Nct. Physical Activity Intervention in ELderly Patients With Myocardial INfarction. https://clinicaltrialsgov/show/NCT04183465 2019.
1080. Nct. Impact of Cardiac Coherence on Anxiety in Patients Operated on for a Peritoneal Carcinosis. https://clinicaltrialsgov/show/NCT04024917 2019.
1081. Nct. Non-ischaemic Heart Preservation Versus Standard Cold Storage in Human Heart Transplantation. https://clinicaltrialsgov/show/NCT04066127 2019.
1082. Nct. Efficacy of Prebiotic and Probiotic Dietary Modulation in Schizophrenic Disorders. https://clinicaltrialsgov/show/NCT04366401 2020.
1083. Nct. The Effectiveness of Video-Based Exercises in Young Adults. https://clinicaltrialsgov/show/NCT04346966 2020.
1084. Nct. Organization of Pulmonary Rehabilitation of Post-COVID-19 Patient With Sequelae (REHABCOVID). https://clinicaltrialsgov/show/NCT04634318 2020.
1085. Nct. Benefits of Exercise Program in Pediatric HSCT. https://clinicaltrialsgov/show/NCT04663503 2020.

1086. Nct. Healthy Lifestyle Intervention in Patients With Systemic Lupus Erythematosus: the Living Well With Lupus Study. https://clinicaltrialsgov/show/NCT04431167 2020.
1087. Nct. Reducing Sedentary Time in Bariatric: the Take a STAND for Health Study. https://clinicaltrialsgov/show/NCT04517591 2020.
1088. Nct. INTERVAL - Intense Exercise Trial for Men With Metastatic Castrate-Resistant Prostate Cancer. https://clinicaltrialsgov/show/NCT04507698 2020.
1089. Nct. Nurse-led COPD Self-management Intervention. https://clinicaltrialsgov/show/NCT04459546 2020.
1090. Nct. A Trial of Durvalumab (MEDI 4736) in Combination With Extended Neoadjuvant Regimens in Rectal Cancer. https://clinicaltrialsgov/show/NCT04621370 2020.
1091. Nct. Impact of a Yoga Intervention on Pain and Multiomics in Participants With IBS. https://clinicaltrialsgov/show/NCT04315714 2020.
1092. Nct. Intralesional Diode Laser Treatment of Fistulas in Hidradenitis Suppurativa. https://clinicaltrialsgov/show/NCT04508374 2020.
1093. Nct. Fighting Immunosenescence and Promoting Immunity by a Fasting-mimicking Diet Elderly. https://clinicaltrialsgov/show/NCT04928963 2021.
1094. Nct. Efficacy of a Novel Sleep Intervention in Short Sleepers. https://clinicaltrialsgov/show/NCT04697680 2021.
1095. Nct. Efficacy of Adaptogens in Patients With Long COVID-19. https://clinicaltrialsgov/show/NCT04795557 2021.
1096. Nct. The Effect of NMES Training in Patients With Hematological Cancer. https://clinicaltrialsgov/show/NCT04755465 2021.
1097. Nct. Immune Checkpoint Inhibitor PD-1 Antibody Combined With Chemotherapy in the Perioperative Treatment of Locally Advanced Resectable Gastric or Gastroesophageal Junction Adenocarcinoma. https://clinicaltrialsgov/show/NCT04908566 2021.
1098. Nct. Cost-utility and Physiological Effects of LDN in Patients With Fibromyalgia. https://clinicaltrialsgov/show/NCT04739995 2021.
1099. Nct. Acute Timed Exercise and 24h Metabolism. https://clinicaltrialsgov/show/NCT05073068 2021.
1100. Nct. A Nurse-Community Health Worker-Family Partnership Model: addressing Uptake of COVID-19 Testing and Control Measures. https://clinicaltrialsgov/show/NCT04832919 2021.
1101. Nct. Aerobic Fitness or Muscle Mass Training to Improve Colorectal Cancer Outcomes. https://clinicaltrialsgov/show/NCT04754672 2021.
1102. Nct. Modifying Metabolic Syndrome and Cardiovascular Risk for Prostate Cancer Patients on ADT Using a Risk Factor Modification Program and Continuous Fitbit Monitoring. https://clinicaltrialsgov/show/NCT05054296 2021.
1103. Nct. Linkage, Empowerment, and Access to Prevent Hypertension. https://clinicaltrialsgov/show/NCT05061108 2021.
1104. Nct. Degenerative Meniscus Without Osteoarthritis : arthroscopic Partial Menisectomy Versus Platelet Rich Plasma. https://clinicaltrialsgov/show/NCT04972331 2021.
1105. Nct. Advancing DSME/S and COVID-19 Prevention and Protection Through "emPOWERed to Change" Program. https://clinicaltrialsgov/show/NCT04993326 2021.
1106. Nct. The Effect of Education Based on the Chronic Care Model in COPD Patients. https://clinicaltrialsgov/show/NCT05029557 2021.
1107. Needham T, Lambrechts H, Hoffman LC. Extending the interval between second vaccination and slaughter: I. Effects on growth, scrotal size and stress responses of immunocastrated ram lambs. Animal 2019; 13(9): 1952-61.
1108. Needleman I, Ashley P, Fine P, et al. Consensus statement: Oral health and elite sport performance. British dental journal 2014; 217(10): 587-90.
1109. Needleman I, Ashley P, Fine P, et al. Oral health and elite sport performance. British journal of sports medicine 2015; 49(1): 3-6.

1110. Neill J, Belan I, Ried K. Effectiveness of non-pharmacological interventions for fatigue in adults with multiple sclerosis, rheumatoid arthritis, or systemic lupus erythematosus: A systematic review. Journal of Advanced Nursing 2006; 56(6): 617-35.
1111. Neimark AI, Bondarev EN. Efficacy of natural therapeutic factors of Belokurikha health resort in rehabilitation of patients with chronic prostatitis. Urologiia (Moscow, Russia : 1999) 2005; (5): 53-6.
1112. Nelson M, Marcus M, Parnes M, Risen S, Muscal E. Striatal changes on MRI in young children with NMDAR: A single center experience. Neurology 2018; 90(15 Supplement 1).
1113. Ngwa MC, Wondimagegnehu A, Okudo I, et al. The multi-sectorial emergency response to a cholera outbreak in Internally Displaced Persons camps in Borno State, Nigeria, 2017. BMJ Glob Health 2020; 5(1): e002000.
1114. Nieman DC. Exercise Is Medicine for Immune Function: Implication for COVID-19. Current Sports Medicine Reports 2021; 20(8): 395-401.
1115. Nieman DC, Lila MA, Gillitt ND. Immunometabolism: A Multi-Omics Approach to Interpreting the Influence of Exercise and Diet on the Immune System. Annual review of food science and technology 2019; 10: 341-63.
1116. Nieman DC, Nehlsen-Cannarella SL. The effects of acute and chronic exercise of immunoglobulins. Sports Med 1991; 11(3): 183-201.
1117. Nijs J, Crombez G, Meeus M, et al. Pain in patients with chronic fatigue syndrome: time for specific pain treatment? Pain Physician 2012; 15(5): E677-86.
1118. Nijs J, Fremont M. Intracellular immune dysfunction in myalgic encephalomyelitis/chronic fatigue syndrome: State of the art and therapeutic implications. Expert Opinion on Therapeutic Targets 2008; 12(3): 281-9.
1119. Nisar MK. Combining secukinumab and fingolimod to successfully treat ankylosing spondylitis and multiple sclerosis: A novel approach. Rheumatology (United Kingdom) 2019; 58(Supplement 3).
1120. Nisar MK, Packianathan CI. Chikungunya and bilateral sacroiliitis-is there a link? Annals of the Rheumatic Diseases 2015; 74(SUPPL. 2): 1213.
1121. Nitert MD, Barrett HL, Callaway LK. Probiotics: A potential role in the prevention of gestational diabetes? Acta Diabetologica 2012; 49(1 SUPPL.): S1-S13.
1122. Niu ZY, Wei FX, Liu FZ, Qin XG, Min YN, Gao YP. Dietary vitamin A can improve immune function in heat-stressed broilers. Animal 2009; 3(10): 1442-8.
1123. Njemini R, CaoDinh H, Onyema OO, et al. Training-induced decrease in senescence-prone T-lymphocytes in peripheral blood: Does cytomegalovirus playa role? Osteoporosis International 2018; 29(1 Supplement 1): S503.
1124. Njemini R, Dinh HC, Onyema OO, et al. Role of cytomegalovirus serostatus on exerciseinduced decrease in senescence-prone T-lymphocytes in peripheral blood. Journal of Cachexia, Sarcopenia and Muscle 2018; 9(6): 1173.
1125. Nl. Multimodal intensive prehabilitation in high impact surgery to reduce postoperative complications. https://trialsearchwhoint/Trial2aspx?TrialID=NL8699 2020.
1126. Nna VU, McGrowder D, Nwokocha C. Nutraceutical management of metabolic syndrome as a palliative and a therapeutic to coronavirus disease (COVID) crisis. Archives of physiology and biochemistry 2021: 1-20.
1127. Noe R, Dimitrov J, Roumenina L, et al. Prevalence and functional characterization of autoantibodies against complement component C3 in patients with lupus nephritis. Molecular Immunology 2015; 67(1): 165.
1128. Noguera Hernando E, Kreisler M, Durantez A, Larrea Gayarre A, de Landazuri MO, Cruz Martínez J. [Inhibition of expontaneous cytotoxicity and antibody dependency by rheumatoid synovial fluid]. Allergol Immunopathol (Madr) 1978; 6(3): 253-62.
1129. Nomura S, Yanabu M, Miyake T, et al. Effect of cepharanthin and cytochalasin D on platelet internalization of anti-glycoprotein IIb/IIIa antibodies. Autoimmunity 1994; 18(1): 23-9.

1130. Nowak-Kornicka J, Borkowska B, Pawłowski B. Masculinity and immune system efficacy in men. PLoS One 2020; 15(12): e0243777.
1131. Nowalk MP, Saul S, Susick M, et al. Physical health and mental health functional status during and following hospitalization for an acute respiratory illness. Brain, Behavior, and Immunity - Health 2020; 1: 100004.
1132. Nsouli TM, Firestone LE, Diliberto NZ, Nsouli ST, Bellanti JA. Immunomodulatory therapy of recurrent and recalcitrant warts: Efficacy using candida albicans antigens. Annals of Allergy, Asthma and Immunology 2014; 113(5 SUPPL. 1): A98.
1133. Ntr. Onderzoek naar de kosten en werkzaamheid van injectiekuren (immunotherapie) bij patiënten met een allergie voor boompollen, graspollen en/of huisstofmijt. https://trialsearchwhoint/Trial2aspx?TrialID=NTR2692 2011.
1134. Nuntawong P, Tanaka H, Sakamoto S, Morimoto S. ELISA for the Detection of the Prohibited Doping Agent Higenamine. Planta Medica 2020; 86(11): 760-6.
1135. Nwokoro E, Leach R, Årdal C, Baraldi E, Ryan K, Plahte J. An assessment of the future impact of alternative technologies on antibiotics markets. J Pharm Policy Pract 2016; 9: 34.
1136. O'Brien K, Nixon S, Tynan AM, Glazier RH. Effectiveness of aerobic exercise in adults living with HIV/AIDS: systematic review. Medicine and science in sports and exercise 2004; 36(10): 1659-66.
1137. O'Bryan TA. Association of ppd positivity and BCG vaccine with cardiovascular disease and markers of inflammation. Journal of General Internal Medicine 2011; 26(SUPPL. 1): S338.
1138. O'Bryant CP, O'Sullivan M, Raudensky J. Socialization of Prospective Physical Education Teachers: The Story of New Blood. Sport, Education & Society 2000; 5(2): 177-93.
1139. O'Donnell LA, Meng WS, Andrick BJ, Borello AM. A Bioinformatics Practicum to Develop Student Understanding of Immunological Rejection of Protein Drugs. Am J Pharm Educ 2016; 80(9): 147.
1140. O'Neill L, Guinan E, Brennan L, et al. ReStOre@Home: Feasibility study of a virtually delivered 12-week multidisciplinary rehabilitation programme for survivors of upper gastrointestinal (UGI) cancer - study protocol. HRB Open Res 2020; 3: 86.
1141. O'Sullivan J, Kaufman J, Tuckerman J, et al. Feasibility and acceptability of the multi-component P3-MumBubVax antenatal intervention to promote maternal and childhood vaccination: A pilot study. Vaccine 2020; 38(24): 4024-31.
1142. Ochi H. Clinical trials for multiple sclerosis: Outcome measures and impact on cognitive function. Clinical and Experimental Neuroimmunology 2019; 10(3): 169-79.
1143. Ogay V, Sekenova A, Li Y, Issabekova A, Saparov A. The Therapeutic Potential of Mesenchymal Stem Cells in the Treatment of Atherosclerosis. Curr Stem Cell Res Ther 2021; 16(7): 897-913.
1144. Okoye-Okafor UC, Barreyro L, Will B, et al. Molecular and functional characterization of the novel protein-coding gene TIHL (translocated in hodgkin's lymphoma) in hematopoiesis. Blood 2013; 122(21).
1145. Oldenburg J, Alvarez Roman MT, Castaman G, et al. Real-world effectiveness and safety of bay 94-9027 (damoctocog alfa pegol) in previously treated patients with haemophilia A (HEM-POWR): Online patient portal and life-active sub-study. Haemophilia 2020; 26(Supplement 2): 100-1.
1146. Oldenburg J, Roman MTA, Castaman G, et al. Real-world effectiveness and safety of BAY 94-9027 (Damoctocog Alfa Pegol) in previously treated patients with hemophilia A (HEM-POWR): Online patient portal and LIFE-ACTIVE sub-study. Blood 2019; 134(Supplement 1).
1147. Olsen MF, Abdissa A, Kaestel P, et al. Effects of nutritional supplementation for HIV patients starting antiretroviral treatment: randomised controlled trial in Ethiopia. BMJ (online) 2014; 348.
1148. Omodior O, Pennington-Gray L, Donohoe H. Efficacy of the Theory of Planned Behavior in Predicting the Intention to Engage in Tick-Borne Disease Personal Protective Behavior

Amongst Visitors to an Outdoor Recreation Center. Journal of Park & Recreation Administration 2015; 33(2): 37-53.
1149. Ong T, Marshall SG, Karczeski BA, Sternen DL, Cheng E, Cutting GR. Cystic Fibrosis and Congenital Absence of the Vas Deferens. In: Adam MP, Ardinger HH, Pagon RA, et al., eds. GeneReviews(®). Seattle (WA): University of Washington, Seattle
Copyright © 1993-2022, University of Washington, Seattle. GeneReviews is a registered trademark of the University of Washington, Seattle. All rights reserved.; 1993.
1150. Ongradi J, Stercz B, Kovesdi V, Vertes L. Immunosenescence and vaccination of the elderly II. New strategies to restore age-related immune impairment. Acta Microbiologica et Immunologica Hungarica 2009; 56(4): 301-12.
1151. Ontiveros ST, Minns A. Acute hypersensitivity reaction from administration of crotalidae immune f(AB')2 antivenom. Clinical Toxicology 2020; 58(11): 1193-4.
1152. Onyema OO, Liberman K, De Dobbeleer L, et al. Six weeks of strength endurance training decreases circulating senescence-prone T-lymphocytes in cytomegalovirus seropositive but not seronegative older women. Immunity and Ageing 2019; 16(1): 17.
1153. Oomman A, Madhusoodanan M. Tropical spastic paraparesis in Kerala. Neurol India 2003; 51(4): 493-6.
1154. Oreska S, Storkanova H, Rathouska A, et al. Efficacy of an intensive 24-week physiotherapy programme in myositis patients - preliminary data from a single-center controlled study. Annals of the Rheumatic Diseases 2017; 76(Supplement 2): 1280-1.
1155. Oreska S, Storkanova H, Rathouska A, et al. Efficacy of an intensive 24-week physiotherapy programme in patients with idiopathic inflammatory myopathies-preliminary data from a single-center controlled study. Arthritis and Rheumatology 2017; 69(Supplement 10).
1156. Orr N, Klement E, Gillis D, et al. Long-term immunity in young adults after a single dose of inactivated Hepatitis A vaccines. Vaccine 2006; 24(20): 4328-32.
1157. Ortiz A, Ramirez-Marrero F, Garcia G, Flores A, Venegas-Rios H. Characterization of body composition, physical fitness and lipid profile among adults living with hiv who participate in a fitness program. Physiotherapy (United Kingdom) 2011; 97(SUPPL. 1): eS943-eS4.
1158. Ortiz de Lejarazu-Leonardo R, Montomoli E, Wojcik R, et al. Estimation of Reduction in Influenza Vaccine Effectiveness Due to Egg-Adaptation Changes-Systematic Literature Review and Expert Consensus. Vaccines (Basel) 2021; 9(11).
1159. Orysiak J, Malczewska-Lenczowska J, Szygula Z, Pokrywka A. THE ROLE OF SALIVARY IMMUNOGLOBULIN A IN THE PREVENTION OF THE UPPER RESPIRATORY TRACT INFECTIONS IN ATHLETES - AN OVERVIEW. Biology of Sport 2012; 29(4): 311-5.
1160. Ottenweller JE, Cook S, Peckerman A, et al. Immunological response in chronic fatigue syndrome following a graded exercise test to exhaustion. Journal of Clinical Immunology 1999; 19(2): 135-42.
1161. Ozdemir C, Akcelik N, Neslihan Ozdemir F, et al. The role of bcsE gene in the pathogenicity of Salmonella. Pathogens and Disease 2021; 79(6): ftab037.
1162. Packer N, Hoffman-Goetz L, Ward G. Does physical activity affect quality of life, disease symptoms and immune measures in patients with inflammatory bowel disease? A systematic review. Journal of Sports Medicine and Physical Fitness 2010; 50(1): 1-18.
1163. Pahlavani MA. Intervention in the aging immune system: Influence of dietary restriction, dehydroepiandrosterone, melatonin, and exercise. Age 1998; 21(4): 153-73.
1164. Palazzo RP, Siqueira IR, Cechinel LR. Circulating extracellular vesicles delivering beneficial cargo as key players in exercise effects. Free Radical Biology and Medicine 2021; 172: 273-85.
1165. Pande R, Bagad M, Dubey V, Ghosh AR. Prospectus of probiotics in modern age diseases. Asian Pacific Journal of Tropical Biomedicine 2012; 2(3 SUPPL.): S1963-S74.
1166. Panos G, Sargianou M, Watson D, et al. Emerging virus coinfections of the CNS: HTLV I/II as a common denominator. BMC Infectious Diseases 2014; 14(Supplement 2).

1167. Panoskaltsis N, McCarthy NE, Knight SC. Myelopoiesis of acute inflammation: lessons from TGN1412-induced cytokine storm. Cancer Immunol Immunother 2021; 70(4): 1155-60.
1168. Parkash V, Jones G, Martin N, et al. Assessing public perception of a sand fly biting study on the pathway to a controlled human infection model for cutaneous leishmaniasis. Res Involv Engagem 2021; 7(1): 33.
1169. Parker PA, Banerjee SC, Matasar MJ, et al. Protocol for a cluster randomised trial of a communication skills intervention for physicians to facilitate survivorship transition in patients with lymphoma. BMJ Open 2016; 6(6): e011581.
1170. Parker PA, Banerjee SC, Matasar MJ, et al. Efficacy of a survivorship-focused consultation versus a time-controlled rehabilitation consultation in patients with lymphoma: a cluster randomized controlled trial. Cancer 2018; 124(23): 4567-76.
1171. Parkin D, McNamee P, Jacoby A, Miller P, Thomas S, Bates D. A cost-utility analysis of interferon beta for multiple sclerosis. Health Technology Assessment 1998; 2(4): 1-45.
1172. Parra-Guillen ZP, Berraondo P, Ribba B, Trocóniz IF. Modeling tumor response after combined administration of different immune-stimulatory agents. J Pharmacol Exp Ther 2013; 346(3): 432-42.
1173. Parry GJ, Kelkar P, Muley SA. Treatment of chronic inflammatory demyelinating polyneuropathy with pulsed oral steroids. Archives of Neurology 2008; 65(11): 1460-4.
1174. Pascoe AR, Fiatarone Singh MA, Edwards KM. The effects of exercise on vaccination responses: a review of chronic and acute exercise interventions in humans. Brain Behav Immun 2014; 39: 33-41.
1175. Patel C, Iweala OI. 'Doc, will I ever eat steak again?': diagnosis and management of alpha-gal syndrome. Current opinion in pediatrics 2020; 32(6): 816-24.
1176. Patel KK, Wininger DA. Self-assessment of performance: A novel curriculum for quality improvement education. Journal of General Internal Medicine 2013; 28(SUPPL. 1): S478.
1177. Patel NB, Galani VJ, Patel BG. Antistress activity of Argyreia speciosa roots in experimental animals. J Ayurveda Integr Med 2011; 2(3): 129-36.
1178. Pattison R, Akiode O, Perez-Guerra F, Ghamande S, Beissner R, Best A. IgG4 related disease emerging. Chest 2015; 148(4 MEETING ABSTRACT).
1179. Paudel M, Buikema AR, Korrer S, et al. Relative vaccine efficacy of high-dose vs. Standard dose influenza vaccines in preventing probable influenza in a US medicare fee-for-service population. Open Forum Infectious Diseases 2019; 6(Supplement 2): S967.
1180. Pavlasova G, Borsky M, Svobodova V, et al. BCR signalling proficient chronic lymphocytic leukaemia B cells are prone to rituximab mediated elimination in vivo. Haematologica 2017; 102(Supplement 2): 412.
1181. Payne C, Wiffen PJ, Martin S. Interventions for fatigue and weight loss in adults with advanced progressive illness. Cochrane Database Syst Rev 2012; 1: Cd008427.
1182. Payne C, Wiffen PJ, Martin S. WITHDRAWN: Interventions for fatigue and weight loss in adults with advanced progressive illness. Cochrane Database Syst Rev 2017; 4(4): Cd008427.
1183. Peake J, Peiffer JJ, Abbiss CR, Nosaka K, Laursen PB, Suzuki K. Carbohydrate gel ingestion and immunoendocrine responses to cycling in temperate and hot conditions. International Journal of Sport Nutrition and Exercise Metabolism 2008; 18(3): 229-46.
1184. Pearn ML, Niesman IR, Egawa J, et al. Pathophysiology Associated with Traumatic Brain Injury: Current Treatments and Potential Novel Therapeutics. Cell Mol Neurobiol 2017; 37(4): 571-85.
1185. Pedersen BK, Bruunsgaard H, Hartkopp A, et al. In vivo cell-mediated immunity and vaccination response following prolonged, intense exercise. Medicine and Science in Sports and Exercise 1997; 29(9): 1176-81.
1186. Pedersen BK, Oritsland TR, Bahr R, Kjeldsen-Kragh J, Ronsen O. Leukocyte counts and lymphocyte responsiveness associated with repeated bouts of strenuous endurance exercise. Journal of Applied Physiology 2001; 91(1): 425-34.

1187. Pellkofer H, Schubart AS, Höftberger R, et al. Modelling paraneoplastic CNS disease: T-cells specific for the onconeuronal antigen PNMA1 mediate autoimmune encephalomyelitis in the rat. Brain 2004; 127(Pt 8): 1822-30.
1188. Pence BD, Ryerson MR, Bravo Cruz AG, Woods JA, Shisler JL. Voluntary Wheel Running Does Not Alter Mortality to or Immunogenicity of Vaccinia Virus in Mice: A Pilot Study. Front Physiol 2017; 8: 1123.
1189. Peretz Y, Cameron C, Sékaly RP. Dissecting the HIV-specific immune response: a systems biology approach. Curr Opin HIV AIDS 2012; 7(1): 17-23.
1190. Perez JM, Gu J, Tannir NM, et al. Genomic DNA hypomethylation as an independent risk factor for renal cell carcinoma. Cancer Research 2015; 75(15 SUPPL. 1).
1191. Perez SG, Nuccio AG, Stripling AM. A Rapid Review of the Detrimental Impact of Loneliness and Social Isolation in Caregivers of Older Adults. American Journal of Geriatric Psychiatry 2021; 29(4 Supplement): S122-S3.
1192. Perin EC, Borow KM, Silva GV, et al. A Phase II Dose-Escalation Study of Allogeneic Mesenchymal Precursor Cells in Patients With Ischemic or Nonischemic Heart Failure. Circulation research 2015; 117(6): 576-84.
1193. Perisetti A, Goyal H, Tharian B, Inamdar S, Mehta JL. Aspirin for prevention of colorectal cancer in the elderly: friend or foe? Annals of gastroenterology 2021; 34(1): 1-11.
1194. Perkin OJ, Travers RL, Gonzalez JT, et al. Exercise strategies to protect against the impact of short-term reduced physical activity on muscle function and markers of health in older men: study protocol for a randomised controlled trial. Trials 2016; 17: 1-11.
1195. Perry C, Pick M, Bdolach N, et al. Endurance exercise diverts the balance between Th17 cells and regulatory T cells. PLoS One 2013; 8(10): e74722.
1196. Perry Jr C, Gleeson M, Clancy RL, McDonald WA, Fricker PA, Pyne DB. Training strategies to maintain immunocompetence in athletes. International Journal of Sports Medicine, Supplement 2000; 21(1): s51-s60.
1197. Pershin BB, Kuz'min SN, Kochkurkin VN, Sukhachevskii AB, Filatov NN. The local immunity reactions of swimmers on the Russian all-star team. Zhurnal mikrobiologii, epidemiologii, i immunobiologii 1996; (1): 53-7.
1198. Perzanowski MS, Ng'Ang'A LW, Carter MC, et al. Atopy, asthma, and antibodies to Ascaris among rural and urban children in Kenya. Journal of Pediatrics 2002; 140(5): 582-8.
1199. Peters-Futre EM. Vitamin C, neutrophil function, and upper respiratory tract infection risk in distance runners: The missing link. Exercise Immunology Review 1997; 3: 32-52.
1200. Petkovic J, Simeon R, Yoganathan M, et al. Behavioural interventions delivered through interactive social media for health behaviour change, health outcomes, and health equity in the adult population. Cochrane Database of Systematic Reviews 2021; 2021(5): CD012932.
1201. Petrochenko SN, Bobrova ZV, Myagkova MA, et al. [The detection of antibodies to endogenous bioregulators for evaluating functional condition of health of portsmen.]. Klin Lab Diagn 2017; 62(6): 346-50.
1202. Petrou P, Kassis I, Levin N, et al. Beneficial effects of autologous mesenchymal stem cell (MSC) transplantation in progressive multiple sclerosis: report of a randomized phase II doubleblind trial. Multiple sclerosis journal 2020; 26(1 SUPPL): 39-.

1203. Petrov RV, Kabanov VA, Khaitov RM, Nekrasov AV, Ataullakhanov RI. Conjugated polymer-subunit immunogens and vaccines. Allergy & Clinical Immunology International 2003; 15(2): 56-61.
1204. Petrovas C, Yamamoto T, Gerner MY, et al. CD4 T follicular helper cell dynamics during SIV infection. Journal of Clinical Investigation 2012; 122(9): 3281-94.
1205. Phan HT, Conrad U. Plant-Based Vaccine Antigen Production. Methods Mol Biol 2016; 1349: 35-47.

1206. Phillips AC, Carroll D, Burns VE, Drayson M. Neuroticism, cortisol reactivity, and antibody response to vaccination. Psychophysiology 2005; 42(2): 232-8.
1207. Phillips AC, Gallagher S, Carroll D, Drayson M. Preliminary evidence that morning vaccination is associated with an enhanced antibody response in men. Psychophysiology 2008; 45(4): 663-6.
1208. Phillips AC, Long JE, Drayson MT, et al. Morning vaccination enhances antibody response over afternoon vaccination: A cluster-randomised trial of older adults. Brain, Behavior, and Immunity 2016; 57(Supplement 1): e3.
1209. Phillips LD, Fasolo B, Zafiropoulous N, et al. Modelling the risk-benefit impact of H1N1 influenza vaccines. Eur J Public Health 2013; 23(4): 674-8.
1210. Phinizy PA, Kattan M. Common variable immunodeficiency (CVID) masquerading as asthma. American Journal of Respiratory and Critical Care Medicine 2015; 191(MeetingAbstracts).
1211. Piantadosi S. Rationale and design of the national emphysema treatment trial: a prospective randomized trial of lung volume reduction surgery. Chest 1999; 116(6): 1750-61.
1212. Piantadosi S, Rodarte J, Miller Iii C, et al. Rationale and design of the national emphysema treatment trial (NETT): a prospective randomized trial of lung volume reduction surgery. Journal of thoracic and cardiovascular surgery 1999; 118(3): 518-28.
1213. Pieri L, Sassoli C, Romagnoli P, Domenici L. Use of periodate-lysine-paraformaldehyde for the fixation of multiple antigens in human skin biopsies. Eur J Histochem 2002; 46(4): 365-75.
1214. Pilotto S, Trestini I, Tregnago D, et al. Force (focus on research and care): A comprehensive lifestyle teamwork intervention to modulate immunological status and treatment outcome in nonsmall cell lung cancer (NSCLC). Tumori 2019; 105(6 Supplement): 104.
1215. Piotrowska A, Pilch W, Tota Ł, Nowak G. [Biological significance of chromium III for the human organism]. Med Pr 2018; 69(2): 211-23.
1216. Piscosquito G, Salsano E, Ciano C, Palamara L, Morbin M, Pareyson D. Double hit on peripheral nerves: Anti-mag neuropathy in cmt1a. Journal of the Peripheral Nervous System 2013; 18(SUPPL. 1): S25-S6.
1217. Piscosquito G, Salsano E, Ciano C, Palamara L, Morbin M, Pareyson D. Anti-MAG neuropathy in CMT1A: Double hit on peripheral nerves. Journal of Neurology 2013; 260(SUPPL. 1): S152-S3.
1218. Piskin DY, Ertunc G, Bas G, Gazeroglu M, Cetin G, Ozsezikli AB. THE EFFECT OF VIRTUAL REALITY TECHNOLOGIES ON THE BALANCE AND WALKING: CASE REPORT. Journal of Exercise Therapy & Rehabilitation 2018; 5: S143-S.
1219. Plat MJ, Frings-Dresen MHW, Sluiter JK. A systematic review of job-specific workers' health surveillance activities for fire-fighting, ambulance, police and military personnel. International Archives of Occupational and Environmental Health 2011; 84(8): 839-57.
1220. Platts-Mills TAE, Carter MC, Heymann PW. Specific and nonspecific obstructive lung disease in childhood: Causes of changes in the prevalence of asthma. Environmental Health Perspectives 2000; 108(SUPPL. 4): 725-31.
1221. Pogancev MK. [Juvenile myasthenia]. Medicinski pregled 2011; 64(5-6): 295-8.
1222. Pohunek P, Svobodova T. Protracted bacterial bronchitis and chronic wet cough. Pediatric Pulmonology 2017; 52(Supplement 46): S54-S6.
1223. Popkin J, Kushniruk A, Borycki E, et al. The eFOSTr PROJECT: design, implementation and evaluation of a web-based Personal Health Record to support health professionals and families of children undergoing transplants. Studies in health technology and informatics 2009; 143: 358-63.
1224. Porat Y, Niven M, Bytner S. Therapeutic Blood-derived stem/progenitor cells specifically activated by dendritic cells. Cytotherapy 2020; 22(5 Supplement): S55-S6.
1225. Pot M, Ruiter RAC, Paulussen T, et al. Systematically Developing a Web-Based Tailored Intervention Promoting HPV-Vaccination Acceptability Among Mothers of Invited Girls Using Intervention Mapping. Front Public Health 2018; 6: 226.

1226. Potluri R, Ranjan S, Khurana R, Lele AM, Prabhakar V, Bhandari H. Evaluation of average cost-effectiveness ratios of standards of care across different indications. Value in Health 2015; 18(7): A575.
1227. Potter PC, Mather S, Lockey P, Ainslie G, Cadman A. IgE specific immune responses to an African grass (Kikuyu, Pennisetum clandestinum). Clin Exp Allergy 1993; 23(7): 581-6.
1228. Pottgiesser T, Schumacher YO, Wolfarth B, Schmidt-Trucksäss A, Bauer G. Longitudinal observation of Epstein-Barr virus antibodies in athletes during a competitive season. J Med Virol 2012; 84(9): 1415-22.
1229. Powderly JD, Hurwitz H, Ryan DP, et al. A phase 1, first-in-human, open label, dose escalation study of MGD007, a humanized gpA33 x CD3 DART molecule, in patients with relapsed/refractory metastatic colorectal carcinoma. Journal of Clinical Oncology 2016; 34(Supplement 15).
1230. Prados G, Miro E. [Fibromyalgia and sleep: a review]. Rev Neurol 2012; 54(4): 227-40.
1231. Pramod S. Therapy approaches for multiple sclerosis. Journal of Neuroimmunology 2014; 275(1-2): 225.
1232. Prattichizzo F, De Nigris V, Spiga R, et al. Inflammageing and metaflammation: The yin and yang of type 2 diabetes. Ageing Res Rev 2018; 41: 1-17.
1233. Preiser W, Doerr HW, Buxbaum S, Rabenau HF, Baatz H. Acute retinal necrosis six years after herpes simplex encephalitis: an elusive immune deficit suggested by insufficient test sensitivity. J Med Virol 2004; 73(2): 250-5.
1234. Prescott SL. Early-life environmental determinants of allergic diseases and the wider pandemic of inflammatory noncommunicable diseases. Journal of Allergy and Clinical Immunology 2013; 131(1): 23-30.
1235. Price S. Talk to Patients About: Diphtheria. Texas medicine 2019; 115(7): 47.
1236. Priyanka G, Anil Kumar B, Lakshman M, Manvitha V, Kala Kumar B. Adaptogenic and Immunomodulatory Activity of Ashwagandha Root Extract: An Experimental Study in an Equine Model. Front Vet Sci 2020; 7: 541112.
1237. Pudkasam S, Pitcher M, Fisher M, et al. The PAPHIO study protocol: a randomised controlled trial with a 2 x 2 crossover design of physical activity adherence, psychological health and immunological outcomes in breast cancer survivors. BMC public health 2020; 20(1): 696.
1238. Pugliese M, Biondi V, Quartuccio M, et al. Use of gnrh agonist in dogs affected with leishmaniosis. Animals 2021; 11(2): 1-10.
1239. Pung MA, Tomfohr LM, Mills PJ, et al. Acute exercise enhancement of pneumococcal vaccination response: A randomised controlled trial of weaker and stronger immune response. Vaccine 2012; 30(45): 6389-95.
1240. Purdy S, Huntley A. Predicting and preventing avoidable hospital admissions: A review. Journal of the Royal College of Physicians of Edinburgh 2013; 43(4): 340-4.
1241. Pyne DB, Donovan T, Bain AL, Tu W, Rao S. Influence of Exercise on Exhausted and Senescent T Cells: A Systematic Review. Frontiers in Physiology 2021; 12: 668327.
1242. Pyne DB, Gleeson M, Gotovtseva EP. Effects of intensive exercise training on immunity in athletes. International Journal of Sports Medicine, Supplement 1998; 19(3): S183-S94.
1243. Pyne DB, Gleeson M, McDonald WA, Clancy RL, Perry C, Jr., Fricker PA. Training strategies to maintain immunocompetence in athletes. Int J Sports Med 2000; 21 Suppl 1: S51-60.
1244. Pyne DB, West NP, Cox AJ, Cripps AW. Probiotics supplementation for athletes – Clinical and physiological effects. European Journal of Sport Science 2015; 15(1): 63-72.
1245. Pyöriä L, Valtonen M, Luoto R, et al. Survey of Viral Reactivations in Elite Athletes: A Case-Control Study. Pathogens 2021; 10(6).
1246. qjcdd RBR. START Trial: randomized, controlled, open and parellel trial to evaluate the efficiency and safety of intra-articular infiltration of Autologous Bone Marrow Concentrate (BMC) or Mesenchymal Stem Cell (MSC) associated with Platelet Rich Plasma (PRP) in patients with primary knee osteoarthritis. https://trialsearchwhoint/Trial2aspx?TrialID=RBR-7qjcdd 2019.

1247. Qu YY, Pu XM, Kang XJ, An CX. A comparison study on the clinical effects of foscarnet sodium injection and interferon on human immunodeficiency virus-infected patients complicated with herpes zoster. Pakistan journal of medical sciences 2015; 31(2): 309-13.
1248. Quak JJ, Van Dongen G, Koken MAE, et al. Identification of a 43-kDa nuclear antigen associated with proliferation by monoclonal antibody K 112. International Journal of Cancer 1990; 46(1): 50-5.
1249. Rabbitt L, Mulkerrin EC, O'Keeffe ST. A review of nocturnal leg cramps in older people. Age & Ageing 2016; 45(6): 776-82.
1250. Rabson A. Enumeration of T-cells subsets in patients with HIV infection. AIDS Clin Care 1995; 7(1): 1-3.
1251. Rahim M, Ooi FK, Wan Abdul Hamid WZ. Blood immune function parameters in response to combined aerobic dance exercise and honey supplementation in adult women. J Tradit Complement Med 2017; 7(2): 165-71.
1252. Rahnavardi M, Gholamin S, Razavi S-M, et al. Antibody response to influenza immunization in coronary artery disease patients: A controlled trial. Vaccine 2009; 28(1): 110-3.
1253. Rajasurya V, Bhavsar S, Gudivada SD, Sharma V. M-mode ultrasound in the diagnosis and follow-up of diaphragmatic dysfunction in the setting of shrinking lung syndrome: A case report. Chest 2014; 146(4 MEETING ABSTRACT).
1254. Ramanathan S, Brilot F, Mohammad SS, Dale RC. Autoantibody-associated movement disorders. Neuropediatrics 2013; 44(6): 336-45.
1255. Ramchandani C, Quwatli W, Abdel-Gadir K. Parsonage-turner syndrome: An unusual presentation. Journal of General Internal Medicine 2014; 29(SUPPL. 1): S406.
1256. Ramezani Ahmadi A, Rayyani E, Bahreini M, Mansoori A. The effect of glutamine supplementation on athletic performance, body composition, and immune function: A systematic review and a meta-analysis of clinical trials. Clinical Nutrition 2019; 38(3): 1076-91.
1257. Ramirez Varela A, Sallis R, Rowlands AV, Sallis JF. Physical Inactivity and COVID-19: When Pandemics Collide. Journal of Physical Activity & Health 2021; 18(10): 1159-60.
1258. Ranchordas MK, Bannock L, Robinson SL. Case Study: Nutritional and Lifestyle Support to Reduce Infection Incidence in an International-Standard Premier League Soccer Player. International Journal of Sport Nutrition & Exercise Metabolism 2016; 26(2): 185-91.
1259. Ranganathan P. An interesting case of Miller Fisher syndrome with ocular myasthenia. Journal of the Neurological Sciences 2019; 405(Supplement): 267.
1260. Raphael JC, Jars-Guincestre MC, Chevret S, Harboun M. Intravenous immune globulins in patients with Guillain-Barre syndrome and contraindications to plasma exchange: 3 Days versus 6 days. Journal of Neurology Neurosurgery and Psychiatry 2001; 71(2): 235-8.
1261. Rapin A, Supper C, Boyer FC, et al. Post-polio syndrome: Pathophysiological hypotheses, diagnosis criteria, medication therapeutics. Annals of Physical and Rehabilitation Medicine 2010; 53(1): 34-41.
1262. Raskova Kafkova L, Brokesova D, Raska M, Novak Z, Pospisilova D, Volejnikova J. Platelet Desialylation As a Predictive Marker in Childhood Immune Thrombocytopenia (ITP). Blood 2019; 134(Supplement 1): 221.
1263. Rawtaer I, Mahendran R, Feng L, et al. Mindful awareness practice for the prevention of dementia: A randomised controlled trial. Annals of the Academy of Medicine Singapore 2014; 43(10 SUPPL. 1): S66-S7.
1264. Raymond M, Iliffe S, Kharicha K, et al. Health risk appraisal for older people 5: self-efficacy in patient-doctor interactions. Primary health care research & development 2011; 12(4): 348-56.
1265. Read KA, Powell MD, Oestreich KJ. T follicular helper cell programming by cytokine-mediated events. Immunology 2016; 149(3): 253-61.
1266. Rectorc JL, Burns VE, Jarczok MN, et al. Personality as a predictor of cytomegalovirus infection. Psychosomatic Medicine 2013; 75(3): A-78.

1267. Reid VL, Gleeson M, Williams N, Clancy RL. Clinical investigation of athletes with persistent fatigue and/or recurrent infections. British Journal of Sports Medicine 2004; 38(1): 42-5.
1268. Reilly-Stitt C, Kitchen S, Jennings I, et al. Anti-PF4 testing for vaccine-induced immune thrombocytopenia and thrombosis and heparin induced thrombocytopenia: Results from a UK National External Quality Assessment Scheme exercise April 2021. J Thromb Haemost 2021; 19(9): 2263-7.
1269. Reinert T, Barrios CH. FATIGUE AND CACHEXIA: FROM BIOLOGY TO SOLUTIONS. Breast 2019; 48(Supplement 2): S26-S7.
1270. Reiss AB, Muhieddine D, De Leon AZ, Voloshyna I, Gomolin IH, Pinkhasov A. Tau and Alzheimer's Disease: Molecular Mechanisms and Treatment Approaches. SN Comprehensive Clinical Medicine 2021; 4(1): 7.
1271. Resnicow K, Teixeira PJ, Williams GC. Efficient Allocation of Public Health and Behavior Change Resources: The "Difficulty by Motivation" Matrix. American Journal of Public Health 2017; 107(1): 55-7.
1272. Rey JL, Rouffy D, Pinte H, Sy D. [Is too much money harmful for innovation?]. Med Sante Trop 2014; 24(1): 6-8.
1273. Rezaee S, Kahrizi S, Nabavi S, Hedayati M. VEGF and TNF-α Responses to Acute and Chronic Aerobic Exercise in the Patients withMultiple Sclerosis. Asian Journal of Sports Medicine 2020; 11(3): 1-6.
1274. Rezzonico-Jost T, Perruzza L, Rottoli E, et al. P2X7 receptor activity limits accumulation of T cells within tumors. Cancer Research 2020; 80(18): 3906-19.
1275. Rhee SY, Jeon H, Kim SW, Lee JS. The effect of an end-effector type of robot-assisted gait training on patients with Guillain-Barre syndrome: a crosssectional study [version 1; peer review: 1 approved]. F1000Research 2020; 9: 1-7.
1276. Riahi A, Zaouali J, Mabrouk E, Messelmani M, Mansour M, Mrissa R. Peripheral neuropathies in multiple myeloma: A clinical and electrophysiological study. European Journal of Neurology 2019; 26(Supplement 1): 744.
1277. Riani Costa LA, R FB, de Leandrini SMM, et al. The influence of a supervised group exercise intervention combined with active lifestyle recommendations on breast cancer survivors' health, physical functioning, and quality of life indices: study protocol for a randomized and controlled trial. Trials 2021; 22(1): 934.
1278. Ribeiro KC, Campelo RP, Rodrigues D, et al. Immunization with plasmids encoding M2 acetylcholine muscarinic receptor epitopes impairs cardiac function in mice and induces autophagy in the myocardium. Autoimmunity 2018; 51(5): 245-57.
1279. Richter J, Thibaud S. Anti-body building: The exercise of advancing immune based myeloma therapies. Blood Reviews 2021; 48: 100789.
1280. Ridker PM, Silvertown JD. Inflammation, C-reactive protein, and atherothrombosis. J Periodontol 2008; 79(8 Suppl): 1544-51.
1281. Riley M, Speck N, Eldred S, Hamid H, Snider J, Weinberg S. A medical student driven "vaccine blitz" at a schoolbased health center as an effective way to improve adolescent vaccination rates. Journal of Adolescent Health 2015; 56(2 SUPPL. 1): S105.
1282. Rinaldi F, Marzani B, Pinto D, Sorbellini E. Randomized controlled trial on a PRP-like cosmetic, biomimetic peptides based, for the treatment of alopecia areata. Journal of dermatological treatment 2019.
1283. Ring J, Brockow K, Behrendt H. History and classification of anaphylaxis. Novartis Foundation symposium 2004; 257.
1284. Rivas E, Crandall CG, Suman OE, Moustaid-Moussa N, Ben-Ezra V. Exercise heat acclimation causes post-exercise hypotension and favorable improvements in lipid and immune profiles: a crossover randomized controlled trial. Journal of thermal biology 2019; 84: 266-73.
1285. Rizzo C, Bella A, Alfonsi V, et al. Influenza vaccine effectiveness in Italy: Age, subtype-specific and vaccine type estimates 2014/15 season. Vaccine 2016; 34(27): 3102-8.

1286. Roberto Santos-Silva P, D'Andrea Greve JM, Pedrinelli A. DURING THE CORONAVIRUS (COVID-19) PANDEMIC, DOES WEARING A MASK IMPROVE OR WORSEN PHYSICAL PERFORMANCE? / ¿DURANTE LA PANDEMIA DE CORONAVIRUS (COVID 19) EL USO DE LA MÁSCARA MEJORA O EMPEORA EL DESEMPEÑO FÍSICO? Revista Brasileira de Medicina do Esporte 2020; 26(4): 281-4.
1287. Roberts JA, Kaack MB, Harrison RM, Klopp R, Ershler W. Bladder infection in the menopausal monkey. Journal of Urology 1999; 162(1): 254-7.
1288. Robinson SA, Mongiardo MA, Finer EB, Cruz Rivera PN, Goldstein RL, Moy ML. Effect of a Web-Based Education Platform on COPD Knowledge: A Retrospective Cohort Study. American Journal of Respiratory and Critical Care Medicine 2021; 203(9).
1289. Roche SE, Garner MG, Sanson RL, et al. Evaluating vaccination strategies to control foot-and-mouth disease: a model comparison study. Epidemiology & Infection 2015; 143(6): 1256-75.
1290. Rockabrand DM, Shaheen HI, Khalil SB, et al. Enterotoxigenic Escherichia coli colonization factor types collected from 1997 to 2001 in US military personnel during operation Bright Star in northern Egypt. Diagn Microbiol Infect Dis 2006; 55(1): 9-12.
1291. Rodrigues AF, Mendonca M, Correia AS, Alves L. Peripheral neuropathy-Report of a case of difficult diagnosis. European Journal of Internal Medicine 2013; 24(SUPPL. 1): e266.
1292. Rodrigues C, Ribeiro A, Martins S, Carvalho S, Pontes T, Antunes A. Spontaneus pneumomediastinum in a pediatric patient. European Journal of Pediatrics 2019; 178(11): 1796.
1293. Rodríguez Hernández C, Sanz Moreno L. [Immunity against SARS-CoV-2: walking to the vaccination]. Rev Esp Quimioter 2020; 33(6): 392-8.
1294. Rogers M, Franco K, Schofield E, et al. Efficacy of a survivorship-focused consultation versus a time-controlled rehabilitation consultation in patients with lymphoma: A cluster randomized controlled trial. Cancer 2018; 124(23): 4567-76.
1295. Romagnani A, Rottoli E, Mazza EMC, et al. P2X7 Receptor Activity Limits Accumulation of T Cells within Tumors. Cancer Res 2020; 80(18): 3906-19.
1296. Roman-Rodriguez M. Early treatment benefits in chronic obstructive pulmonary disease. Hot Topics in Respiratory Medicine 2010; (14): 19-26.
1297. Romutis S, Momi N, DelaCruz M, Farraye F, Chowdhury S, Roy HK. Exercise-induced myokines improve intestinal wound healing: Potential for novel therapeutics. Gastroenterology 2017; 152(5 Supplement 1): S765.
1298. Rongen GA, Marquet P, van Gerven JMA. The scientific basis of rational prescribing. A guide to precision clinical pharmacology based on the WHO 6-step method. European Journal of Clinical Pharmacology 2021; 77(5): 677-83.
1299. Ronsen O, Pedersen BK, Øritsland TR, Bahr R, Kjeldsen-Kragh J. Leukocyte counts and lymphocyte responsiveness associated with repeated bouts of strenuous endurance exercise. J Appl Physiol (1985) 2001; 91(1): 425-34.
1300. Rosenow CS, Dawit S, Farrugia LP, et al. Case Report: Opsoclonus-Myoclonus Syndrome Associated With Contactin-Associated Protein-Like 2 and Acetylcholine Receptor Autoantibodies in the Setting of Non-Small Cell Lung Carcinoma. Neurohospitalist 2022; 12(1): 100-4.
1301. Roser JF, Meyers-Brown G. Enhancing Fertility in Mares: Recombinant Equine Gonadotropins. Journal of Equine Veterinary Science 2019; 76: 6-13.
1302. Ross K, Kalula SZ. Immunosenescence - Inevitable or preventable? Current Allergy and Clinical Immunology 2008; 21(3): 126-30.
1303. Roukas C, Tomini F, Mihaylova B. UK cost-effectiveness value pyramid of asthma interventions. Thorax 2019; 74(Supplement 2): A116.
1304. Roy A, Nair S, Sen N, Soni N, Madhusudhan MS. In silico methods for design of biological therapeutics. Methods 2017; 131: 33-65.
1305. Roy CL, Carstensen M, LaSharr K, et al. WEST NILE VIRUS EXPOSURE AND INFECTION AMONG HUNTER-HARVESTED RUFFED GROUSE (BONASA UMBELLUS) COHORTS IN A STABLE POPULATION. J Wildl Dis 2022; 58(1): 30-9.

1306. Rozenbaum MH, Postma MJ, Meijboom MJ, et al. Cost-effectiveness of potential infant vaccination against respiratory syncytial virus infection in The Netherlands. Vaccine 2012; 30(31): 4691-700.
1307. Rubin BK. Aerosol antibiotics-not just for CF. Paediatric respiratory reviews Conference: 10th international congress on pediatric pulmonology France Conference start: 20110625 Conference end: 20110627 2011; 12: S37-S8.
1308. Rubin Z, Michaud DS, Riboli E, et al. Lifestyle, dietary factors, and antibody levels to oral bacteria in cancer-free participants of a European cohort study. Cancer Causes and Control 2013; 24(11): 1901-9.
1309. Rubinstein I, Reiss TF, Bigby BG, Stites DP, Boushey Jr HA. Effects of 0.60 PPM nitrogen dioxide on circulating and bronchoalveolar lavage lymphocyte phenotypes in healthy subjects. Environmental Research 1991; 55(1): 18-30.
1310. Ruppert L, Kendig T. A PT intervention for a patient with sensory ataxia in the acute care oncology setting. Rehabilitation Oncology 2012; 30(1): 24-5.
1311. Saad HB. The impact of cold-water-immersion on athletes' muscle function and aerobic capacity: Studies from thin to thick. Tunisie Medicale 2021; 99(3): 343-7.
1312. Sacco O, Capizzi A, Tosca M, Rossi GA. Biologicals in asthma treatment. Pediatric pulmonology 2017; 52: S45-S7.
1313. Sadabadi F, Zirak RG, Mohadese r, et al. Physical activity level (PAL) and risk factors of cardiovascular disease in the MASHAD study cohort. Diabetes and Metabolic Syndrome: Clinical Research and Reviews 2021; 15(6): 102316.
1314. Saeed M, Gohar A, Siuba M, Shahzadi A, Khan M. Miller fischer syndrome; a rare variant of guillian barre syndrome. American Journal of Respiratory and Critical Care Medicine 2021; 203(9).
1315. Safarian M, Jokar M, Tabesh H, et al. The effect of propolis supplementation on inflammatory factors and oxidative status in women with rheumatoid arthritis: Design and research protocol of a double-blind, randomized controlled. Contemporary Clinical Trials Communications 2021; 23: 100807.
1316. Sagai M, Bocci V. Mechanisms of Action Involved in Ozone Therapy: Is healing induced via a mild oxidative stress? Med Gas Res 2011; 1: 29.
1317. Sagiv Barfi I, Czerwinski DK, Levy R. In Situ Vaccination with IL-12Fc and TLR Agonist - a Crucial Role for B Cells in Generating Anti-Tumor T Cell Immunity. Blood 2021; 138(Supplement 1): 3514.
1318. Saha P, Mukherjee J, Bose DL, Ul Hadi S, Goyal K. Using participatory action research tools to understand gender dynamics in uptake of HIV prevention measures and participation in research: Insights through a road-block mapping exercise. Journal of the International AIDS Society 2021; 24(SUPPL 1): 94.
1319. Şahbaz Y, Yeldan İ. SEREBRAL PALSİ TANILI OLGULARDA KİNEZYOLOJİK BANTLAMA UYGULAMASI HAKKINDA LİTERATÜR ÖZETİ. / LİTERATURE REVİEW ON KİNESİO TAPİNG APPLİCATİON İN PATİENTS WİTH CEREBRAL PALSY. Journal of Exercise Therapy & Rehabilitation 2018; 5: S106-S.
1320. Sahraian MA, Maghzi AH, Etemadifar M, Minagar A. Dalfampridine: review of its efficacy in improving gait in patients with multiple sclerosis. J Cent Nerv Syst Dis 2011; 3: 87-93.
1321. Saito MM, Ejima K, Kinoshita R, Nishiura H. Assessing the effectiveness and cost-benefit of test-and-vaccinate policy for supplementary vaccination against rubella with limited doses. International Journal of Environmental Research and Public Health 2018; 15(4): 572.
1322. Saito Reis CA, Marjon KD, Karlen KL, Dodd RJ, Termini CM, Gillette JM. The tetraspanin CD82 regulates hematopoietic stem cell fitness. Molecular Biology of the Cell 2015; 26(25).
1323. Saleem S, Patel R, Gawarikar Y. A case of myasthenia gravis lambert eaton overlap syndrome (MLOS) in seronegative myasthenia gravis. Journal of Neurology, Neurosurgery and Psychiatry 2018; 89(6): e39.

1324. Sankowski R, Mader S, Valdés-Ferrer SI. Systemic inflammation and the brain: novel roles of genetic, molecular, and environmental cues as drivers of neurodegeneration. Front Cell Neurosci 2015; 9: 28.
1325. Santos ECMD, Santos AV, Picone CdM, Segurado AC, Florindo AA, Dias TG. Multicomponent physical activity program to prevent body changes and metabolic disturbances associated with antiretroviral therapy and improve quality of life of people living with HIV: a pragmatic trial. Clinics (Sao Paulo, Brazil) 2021; 76: e2457.
1326. Santos-Araújo C, Veiga PM, Santos MJ, et al. Time-dependent evolution of IgG antibody levels after first and second dose of mRNA-based SARS-CoV-2 vaccination in hemodialysis patients: a multicenter study. Nephrol Dial Transplant 2021.
1327. Sarin HV, Gudelj I, Honkanen J, et al. Molecular Pathways Mediating Immunosuppression in Response to Prolonged Intensive Physical Training, Low-Energy Availability, and Intensive Weight Loss. Front Immunol 2019; 10: 907.
1328. Sarrigiannis PG, Thorpe A, Jessop H, et al. Autologous haematopoietic stem cell transplantation for refractory stiff-person syndrome: the UK experience. Journal of Neurology 2021; 268(1): 265-75.
1329. Sato Y, Tanaka Y, Hino M, Seike M, Gemma A. A case of nivolumab-induced isolated adrenocorticotropic hormone (ACTH) deficiency. Respiratory Medicine Case Reports 2019; 26: 223-6.
1330. Sauter ER. Breast cancer prevention: Current approaches and future directions. Meme Sagligi Dergisi / Journal of Breast Health 2018; 14(2): 64-71.
1331. Savioli FP, Medeiros TM, Camara Jr SL, Peres Biruel E, Andreoli CV. DIAGNOSIS OF OVERTRAINING SYNDROME. / DIAGNÓSTICO DEL SÍNDROME DEL OVERTRAINING. Revista Brasileira de Medicina do Esporte 2018; 24(5): 391-4.
1332. Savitskaya YA, Duarte C, Tellez R, et al. The identification of circulating natural antibodies against endogenous mediators in the peripheral blood sera of patients with osteoarthritis of the knee: A new diagnostic frontier. Osteoarthritis and Cartilage 2010; 18(SUPPL. 2): S30-S1.
1333. Savoy L, Young C, Abbas T, Messenger G. Clinical and cost effectiveness of antioxidants and lifestyle modification as treatment for psoriasis in Veteran patients. Journal of Investigative Dermatology 2016; 136(5 SUPPL. 1): S35.
1334. Sawaki K, Takaoka I, Sakuraba K, Suzuki Y. Effects of distance running and subsequent intake of glutamine rich peptide on biomedical parameters of male Japanese athletes. Nutrition Research 2004; 24(1): 59-71.
1335. Sawynok J. Adenosine receptor targets for pain. Neuroscience 2016; 338: 1-18.
1336. Saxton JM, Daley A, Woodroofe N, et al. Study protocol to investigate the effect of a lifestyle intervention on body weight, psychological health status and risk factors associated with disease recurrence in women recovering from breast cancer treatment. BMC cancer 2006; 6.
1337. Saygin O, Zorba E, Karacabey K, Ozmerdivenli R, Ilhan F, Bulut V. Effect of chronic exercise on immunoglobin, complement and leukocyte types in volleyball players and athletes. Neuroendocrinology Letters 2006; 27(1-2): 271-6.
1338. Schaffner W, Rehm SJ, File Jr TM. Keeping our adult patients healthy and active: The role of vaccines across the lifespan. Physician and Sportsmedicine 2010; 38(4): 35-47.
1339. Schauer M, Filzwieser I, Geisler S, Gatterer H, Fuchs D, Burtscher M. Urinary neopterin in professional climbers. Pteridines 2017; 28(1): 54.
1340. Scheife RT, Hills JR, Munsat TL. Myasthenia gravis: signs, symptoms, diagnosis, immunology, and current therapy. Pharmacotherapy 1981; 1(1): 39-54.
1341. Scherer S, Jansen L, Boakye D, Hoffmeister M, Brenner H. Changes in health-related outcomes among colorectal cancer patients undergoing inpatient rehabilitation therapy: a systematic review of observational and interventional studies. Acta Oncologica 2021; 60(1): 124-34.
1342. Schibler KR, Liechty KW, White WL, Rothstein G, Christensen RD. Defective production of interleukin-6 by monocytes: A possible mechanism underlying several host defense deficiencies of neonates. Pediatric Research 1992; 31(1): 18-21.

1343. Schiopu E, Farshad S, Abdulaziz N, Anderson S, Impens A. Pain patterns in idiopathic inflammatory myopathy (IIM): Associations with disease activity measures (Muscle Enzymes, Manual Muscle Testing 8), patient-reported quality of life (HAQ) and pain scales (Widespread Pain Index (WPI), Symptom Severity Index (SSI) and Visual Analogue Scale). Arthritis and Rheumatology 2019; 71(Supplement 10): 5048-51.
1344. Schlenk EA, Bilt JV, Lo-Ciganic W-H, et al. Integration of a healthy aging program into the arthritis foundation exercise program: Six-month results. Arthritis and Rheumatism 2012; 64(SUPPL. 10): S1024-S5.
1345. Schmielau J. The need for rehabilitation in the era of new therapies. Oncology Research and Treatment 2018; 41(Supplement 4): 5-6.
1346. Schneider F, Horowitz A, Lesch KP, Dandekar T. Delaying memory decline: different options and emerging solutions. Transl Psychiatry 2020; 10(1): 13.
1347. Schneider RH, Walton KG, Salerno JW, Nidich SI. Cardiovascular disease prevention and health promotion with the transcendental meditation program and Maharishi Consciousness-Based Health Care. Ethnicity & disease 2006; 16(3): S4-15-S4-26.
1348. Scholler A, Ledniczky G, Kalmar A, et al. Increased cell-free DNA influences cancer growth in a mouse model. United European Gastroenterology Journal 2015; 3(5 SUPPL. 1): A8-A9.
1349. Schoser B, Jacob S, Vincent A, et al. Immune-mediated rippling muscle disease with myasthenia gravis: A report of seven patients with long-term follow-up in two. Neuromuscular Disorders 2009; 19(3): 223-8.
1350. Schuler PB. The effect of moderate physical activity on the production of antibody in response to bovine serum albumin in rats. Ann Arbor, Mich.;: University Microfilms International; 1995.
1351. Schuler PB, LeBlanc PA, Westerfield RC. The effect of moderate physical activity on the production of antibody in response to bovine serum albumin in rats. Medicine, Exercise, Nutrition & Health 1995; 4(6): 363-8.
1352. Schwall JA, Kosinski MA, Gnewikow DM, Shackelford DYK, Brown JM. The Administration Of Exercise Via Telehealth Promotes Physiological Benefits Amidst The Covid-19 Pandemic. Medicine & Science in Sports & Exercise 2021; 53: 452-.
1353. Schwarz PEH, Melzer N, Barteczek P, Reinhardt M, Pinter A. ERAPSO-Assessment of eating behavior, physical activity and prevalence of obesity in German psoriasis patients. Journal of the American Academy of Dermatology 2019; 81(4 Supplement 1): AB57.

1354. Sehatzadeh S. Influenza and pneumococcal vaccinations for patients with chronic obstructive pulmonary disease (COPD): an evidence-based review. Ont Health Technol Assess Ser 2012; 12(3): 1-64.
1355. Seib K, Omer SB, Barnett DJ, Weiss PS. Vaccine-related standard of care and willingness to respond to public health emergencies: A cross-sectional survey of California vaccine providers. Vaccine 2012; 31(1): 196-201.
1356. Seidu S, Khunti K, Yates T, Davies MJ, Sargeant J, Almaqhawi A. The importance of physical activity in management of type 2 diabetes and COVID-19. Therapeutic Advances in Endocrinology and Metabolism 2021; 12.
1357. Sekine C, Sugihara T, Miyasaka N, et al. Successful treatment of animal models of rheumatoid arthritis with small-molecule cyclin-dependent kinase inhibitors. Journal of Immunology 2008; 180(3): 1954-61.
1358. Selak S, Fritzler MJ. Altered neurological function in mice immunized with early endosome antigen 1. BMC Neuroscience 2004; 5: 2.
1359. Selfridge NJ. Meditation and Exercise vs Common Cold -- An ounce of Prevention and Pound of Cure? Integrative Medicine Alert 2012; 15(11): 129-31.
1360. Seligsohn U. Treatment of inherited platelet disorders. Haemophilia 2012; 18(SUPPL. 3): 156.

1361. Selvaratnam V, Rui Jeat F, Yee Yee Y, Sathar J. Emicizumab and surgery in severe haemophilia a with inhibitor: Our first experience. Research and Practice in Thrombosis and Haemostasis 2020; 4(SUPPL 1): 491-2.
1362. Semple S. Circulating immune complexes — reviewing the biological roles in human immune function and exercise. African Journal for Physical, Health Education, Recreation & Dance 2011; 17(4): 574-80.
1363. Senchina DS, Kohut ML. Immunological outcomes of exercise in older adults. Clinical interventions in aging 2007; 2(1): 3-16.
1364. Shah PK, Mukherjee N, Ji N, et al. Effects of yoga in men with prostate cancer on quality of life and immune response: a pilot randomized controlled trial. Prostate Cancer and Prostatic Diseases 2021.
1365. Shah R, Agarwal R. A rare case of proximal muscle weakness. Journal of General Internal Medicine 2014; 29(SUPPL. 1): S282.
1366. Shahbari NAE, Gesser-Edelsburg A, Mesch GS, Abed Elhadi Shahbari N. Case of Paradoxical Cultural Sensitivity: Mixed Method Study of Web-Based Health Informational Materials About the Human Papillomavirus Vaccine in Israel. Journal of Medical Internet Research 2019; 21(5): N.PAG-N.PAG.
1367. Shahrokh S, Qobadighadikolaei R, Abbasinazari M, et al. Efficacy and safety of melatonin as an adjunctive therapy on clinical, biochemical, and quality of life in patients with ulcerative colitis. Iranian journal of pharmaceutical research 2021; 20(2): 197-205.
1368. Shao T, Verma HK, Pande B, et al. Physical Activity and Nutritional Influence on Immune Function: An Important Strategy to Improve Immunity and Health Status. Frontiers in Physiology 2021; 12: 751374.
1369. Shao-Chiang Chang M, Wang J-S. Effects of cycling exercise on mitochondrial function and efficacy of peripheral blood mononuclear cells under hypoxic condition in sedentary. FASEB Journal 2017; 31(1 Supplement 1).
1370. Shapiro AD, Konkle BA, Croteau SE, et al. First-in-Human Phase 1/2 Clinical Trial of SIG-001, an Innovative Shielded Cell Therapy Platform, for Hemophilia A. Blood 2020; 136(Supplement 1): 8.
1371. Shaw E, Wong S, Kaczorowski J, et al. Systematic review of the literature on postpartum care: Methodology and literature search results. Birth 2004; 31(3): 196-202.
1372. Shearer WT, Ochs HD, Lee BN, et al. Immune responses in adult female volunteers during the bed-rest model of spaceflight: antibodies and cytokines. J Allergy Clin Immunol 2009; 123(4): 900-5.
1373. Shedlovsky T, Smadel JE. THE LS-ANTIGEN OF VACCINIA : II. ISOLATION OF A SINGLE SUBSTANCE CONTAINING BOTH L- AND S-ACTIVITY. J Exp Med 1942; 75(2): 165-78.
1374. Shek PN, Shephard RJ. Exercise in the assessment and treatment of patients with cancer. Critical Reviews in Physical and Rehabilitation Medicine 1998; 10(1): 37-56.
1375. Shemesh AA, Rasooly I, Horowitz P, et al. Health behaviors and their determinants in multiethnic, active Israeli seniors. Arch Gerontol Geriatr 2008; 47(1): 63-77.
1376. Shephard RJ. The Case for Increased Physical Activity in Chronic Inflammatory Bowel Disease: A Brief Review. International journal of sports medicine 2016; 37(7): 505-15.
1377. Sherer RD, Fath MJ, Da Silva BA, Nicolau A, Miller NL. The importance of potency and durability in HIV patient antiretroviral therapy preferences: a telephone survey. AIDS Patient Care & STDs 2005; 19(12): 794-802.
1378. Shinkai S, Konishi M, Shephard RJ. Aging, exercise, training, and the immune system. Exercise Immunology Review 1997; 3: 68-95.
1379. Shintani Y, Uchida K. Clinical case study of adult Streptococcus pneumoniae infection with evidence of bronchiolitis on CT. Journal of Tokyo Medical University 2015; 73(2): 130-6.

1380. Shipelin VA, Trusov NV, Apryatin SA, et al. Comprehensive assessment of the effectiveness of l-carnitine and transresveratrol in rats with diet-induced obesity. Nutrition 2021; 95: 111561.
1381. Shobugawa Y, Saito R, Sato I, et al. Clinical effectiveness of neuraminidase inhibitors--oseltamivir, zanamivir, laninamivir, and peramivir--for treatment of influenza A(H3N2) and A(H1N1)pdm09 infection: an observational study in the 2010-2011 influenza season in Japan. J Infect Chemother 2012; 18(6): 858-64.
1382. Shree T, Haebe S, Sathe A, et al. Single Cell Analysis of Serial Lymphoma Biopsies Reveals Dynamic Immune Modulation and Predictors of Response in Patients Undergoing in Situ Vaccination. Blood 2020; 136(Supplement 1): 36-7.
1383. Shree T, Haebe S, Sathe A, et al. Dynamic Immune Modulation Seen By Single Cell RNA-Sequencing of Serial Lymphoma Biopsies in Patients Undergoing in Situ Vaccination. Blood 2019; 134(Supplement 1): 1479.
1384. Shree T, Khodadoust MS, Czerwinski DK, et al. A Phase I/II Trial of Intratumoral CpG, Local Low-Dose Radiation, and Oral Ibrutinib in Patients with Low-Grade B-Cell Lymphoma. Blood 2019; 134(Supplement 1): 2825.
1385. Shree T, Shankar V, Rodriguez G, et al. Time Since Last Anti-CD20 Treatment Is a Major Determinant of Sars-Cov-2 Vaccine Response in a Large Cohort of Patients with B-Cell Lymphoma. Blood 2021; 138(Supplement 1): 2064.
1386. Shukla S, Averma A. Inclusion body myopathy in HIV: Clinical spectrum. Journal of Clinical Neuromuscular Disease 2021; 22(1 SUPPL): S3-S4.
1387. Shvidel L, Sigler E, Cohn M. Chronic Lymphocytic Leukemia: Recent progress and unresolved questions in clinical settings. International Journal of Cancer Research and Prevention 2013; 6(1): 89-113.
1388. Shwartzman G. STUDIES ON BACILLUS TYPHOSUS TOXIC SUBSTANCES : III. THE EFFECT OF SERA UPON THE INJURY PRODUCING FACTORS OF THE PHENOMENON OF LOCAL SKIN REACTIVITY. J Exp Med 1929; 50(4): 513-20.
1389. Sicherer SH, Warren CM, Dant C, Gupta RS, Nadeau KC. Food Allergy from Infancy Through Adulthood. J Allergy Clin Immunol Pract 2020; 8(6): 1854-64.
1390. Siddique Qurashi HM, Sendil S, Saleem T, Yarlagadda K, Shingala H. FIBROSING MEDIASTINITIS: CONSERVATIVE MANAGEMENT IN AN ASYMPTOMATIC PATIENT. Chest 2020; 158(4 Supplement): A1220.
1391. Siebert DJ, Lindschau PB, Burrell CJ. Lack of evidence for significant hepatitis B transmission in Australian Rules footballers. Medical Journal of Australia 1995; 162(6): 312-3.
1392. Silano V, Bolognesi C, Castle L, et al. A statement on the developmental immunotoxicity of bisphenol A (BPA): answer to the question from the Dutch Ministry of Health, Welfare and Sport. Efsa j 2016; 14(10): e04580.
1393. Silva Filho E, Xavier J, Cezarino L, Sales H, Albuquerque J. Comment on "The importance of physical exercise during the coronavirus (COVID-19) pandemic". Rev Assoc Med Bras (1992) 2020; 66(9): 1311-3.
1394. Silva VR, Belozo FL, Micheletti TO, et al. beta-hydroxy-beta-methylbutyrate free acid supplementation may improve recovery and muscle adaptations after resistance training: a systematic review. Nutrition Research 2017; 45: 1-9.
1395. Silva VR, Belozo FL, Micheletti TO, et al. β-hydroxy-β-methylbutyrate free acid supplementation may improve recovery and muscle adaptations after resistance training: a systematic review. Nutrition Research 2017; 45: 1-9.
1396. Silva-Filho E, Xavier J, Cezarino L, Sales H, Albuquerque J. Comment on "The importance of physical exercise during the coronavirus (COVID-19) pandemic". Revista da Associacao Medica Brasileira 2020; 66(9): 1311-3.

1397. Silveira SL, Richardson EV, Motl RW. Social cognitive theory as a guide for exercise engagement in persons with multiple sclerosis who use wheelchairs for mobility. Health education research 2020; 35(4): 270-82.
1398. Silvério de Godoy GS, Carlovich Nunes M, Queiroz Bernardino T, Furlan Viebig R. POTENCIAIS BENEFÍCIOS DO USO DE PROBIÓTICOS PARA ATLETAS. / Potential benefits of using probiotics for athletes. Revista Brasileira de Prescrição e Fisiologia do Exercício 2020; 14(94): 1024-32.
1399. Silvertown JD, Ridker PM. Inflammation, C-reactive protein, and atherothrombosis. Journal of Periodontology 2008; 79(8 SUPPL.): 1544-51.
1400. Simonini G, Matucci Cerinic M, Cimaz R, et al. Evidence for immune activation against oxidized lipoproteins in inactive phases of juvenile chronic arthritis. J Rheumatol 2001; 28(1): 198-203.
1401. Simonson SR. The effects of acute and chronic weight training by moderately conditioned and weight-trained individuals on selected immune parameters: University of Northern Colorado; 1998.
1402. Simpson IJ, Myles MJ, Smith GW. Immune complexes in normal subjects. Journal of Clinical and Laboratory Immunology 1983; 11(3): 119-22.
1403. Simpson RJ, Cosgrove C, Chee MM, et al. Senescent phenotypes and telomere lengths of peripheral blood T-cells mobilized by acute exercise in humans. Exercise Immunology Review 2010; 16: 40-55.
1404. Singh S, Ahirwar AK, Asia P, Gopal N, Kaim K, Ahirwar P. COVID-19 and neurology perspective. Horm Mol Biol Clin Investig 2021; 42(1): 69-75.
1405. Singh SK, Shaikh W. Anatomy, physiology, and biochemistry of happiness; How sustained happiness can be achieved? Indian Journal of Physiology and Pharmacology 2013; 57(5 SUPPL. 1): 31-2.
1406. Siqueira IR, Palazzo RP, Cechinel LR. Circulating extracellular vesicles delivering beneficial cargo as key players in exercise effects. Free Radic Biol Med 2021; 172: 273-85.
1407. Skalkou A, Petidis K, Pyrpasopoulou A, et al. Pyoderma gangrenosum: A paradoxical side effect in a rheumatoid arthritis patient treated with golimumab. Clinical and Experimental Rheumatology 2018; 36(Supplement 109): S82-S3.
1408. Smedley J, Coggon D, Heap D, Ross A. Management of sharps injuries and contamination incidents in health care workers: An audit in the Wessex and Oxford regions. Occupational Medicine 1995; 45(5): 273-5.
1409. Smith J, Petinos P, Hertzberg M, Koutts J, Favaloro EJ. Laboratory testing for von Willebrand's disease: An assessment of current diagnostic practice and efficacy by means of a multi-laboratory survey. Thrombosis and Haemostasis 1999; 82(4): 1276-82.
1410. Smith KA, Batatinha H, Hoffman EA, et al. Voluntary Wheel Running Slows Tumor Progression In A Murine Lymphoma Model. Medicine & Science in Sports & Exercise 2021; 53: 367-.
1411. Smith LL. Overtraining, excessive exercise, and altered immunity: Is this a T helper-1 versus T helper-2 lymphocyte response? Sports Medicine 2003; 33(5): 347-64.
1412. Smith ME, Haney E, McDonagh M, et al. Treatment of Myalgic Encephalomyelitis/Chronic Fatigue Syndrome: A Systematic Review for a National Institutes of Health Pathways to Prevention Workshop. Ann Intern Med 2015; 162(12): 841-50.
1413. Smith TP, Kennedy SL, Fleshner M. Influence of age and physical activity on the primary in vivo antibody and T cell-mediated responses in men. Journal of Applied Physiology 2004; 97(2): 491-8.
1414. So H, Mok CC, Yip RML. The Hong Kong Society of Rheumatology Consensus Recommendations for COVID-19 Vaccination in Adult Patients with Autoimmune Rheumatic Diseases. Journal of Clinical Rheumatology and Immunology 2021; 21(1): 7-14.
1415. Soares J, Kinghorn B, Deutsch G, Stevens AM. Pauciimmune and immune mediated pulmonary capillaritis in children. Arthritis and Rheumatology 2015; 67(SUPPL. 10).
1416. Soda N, Gonzaga ZJ, Pannu AS, et al. Electrochemical Detection of Global DNA Methylation Using Biologically Assembled Polymer Beads. Cancers (Basel) 2021; 13(15).

1417. Sojcher R. An integrative approach to the treatment of obesity and binge eating disorder: A review of 2 mind-body therapies. Topics in Clinical Nutrition 2011; 26(4): 346-52.
1418. Soliz LJ, VandenBerg JC. Newly diagnosed postural orthostatic tachycardia syndrome (POTS) in the acute rehab setting in a patient with recently diagnosed immune-mediated myopathy with perimysial pathology: A case report. PM and R 2017; 9(9 Supplement 1): S225.
1419. Somarriba G, Neri D, Schaefer N, Miller TL. The effect of aging, nutrition, and exercise during HIV infection. HIV AIDS (Auckl) 2010; 2: 191-201.
1420. Somerville VS, Braakhuis AJ, Hopkins WG. Effect of Flavonoids on Upper Respiratory Tract Infections and Immune Function: A Systematic Review and Meta-Analysis. Advances in nutrition (Bethesda, Md) 2016; 7(3): 488-97.
1421. Somkrua R, Chaiyakunapruk N, Hutubessy R, et al. Cost effectiveness of pediatric pneumococcal conjugate vaccines: A comparative assessment of decision-making tools. BMC Medicine 2011; 9: 53.
1422. Song J. Correlations of smoking status vs cancer mortality and other health statuses and demographics among 5919 cancer survivors. Supportive Care in Cancer 2017; 25(2 Supplement 1): S110.
1423. Sonnenfeld G. Immune responses in space flight. International Journal of Sports Medicine 1998; 19(Suppl 3): S195-s202.
1424. Sood L, Hix JK, Silver S. Crescentic glomerulonephritis complicating Castleman's disease. American Journal of Kidney Diseases 2013; 61(4): A91.
1425. Sorensen RU, Wall LA, Wisner EL, Gipson KS. Bronchiectasis in Primary Antibody Deficiencies: A Multidisciplinary Approach. Frontiers in Immunology 2020; 11: 522.
1426. Sorg C, Michels E, Malorny U, Neumann C. Migration inhibitory factors and macrophage differentiation. Springer Semin Immunopathol 1984; 7(4): 311-20.
1427. Souissi MA, Bellakhal S, Gharbi E, Abdelkefi I, Douggui MH. A Stiff Person Syndrome Misdiagnosed as a Psychiatric Illness: A Case Report. Curr Rheumatol Rev 2020; 16(4): 343-5.
1428. Sousa AC, Moraes LP, Da Silva TON, et al. Severe systemic lupus erythematosus: Clinical evolution and therapeutic response. Advances in Rheumatology 2018; 58(Supplement 1).
1429. Spankovich C, Le Prell CG. The role of diet in vulnerability to noise-induced cochlear injury and hearing loss. The Journal of the Acoustical Society of America 2019; 146(5): 4033.
1430. Spiritovic M, Hermankova B, Oreska S, et al. Effectiveness of specialized and intensive ADL training in patients with idiopathic inflammatory myopathies-Preliminary results of a one-year controlled study. Annals of the Rheumatic Diseases 2019; 78(Supplement 2): 1804.
1431. Srinithya F, Gadde S. A stiff woman. Journal of General Internal Medicine 2019; 34(2 Supplement): S459.
1432. Srinivasan M, Torres JE, McGeary D, Nagpal AS. Complementary and Alternative (CAM) Treatment Options for Women with Pelvic pain. Curr Phys Med Rehabil Rep 2020; 8(3): 240-8.
1433. Staats R, Balkow S, Sorichter S, et al. Change in perforin-positive peripheral blood lymphocyte (PBL) subpopulations following exercise. Clin Exp Immunol 2000; 120(3): 434-9.
1434. Stachowicz M, Lebiedzińska A. The role of vitamin D in health preservation and exertional capacity of athletes. Postepy Hig Med Dosw (Online) 2016; 70(0): 637-43.
1435. Staines DR. Is chronic fatigue syndrome an autoimmune disorder of endogenous neuropeptides, exogenous infection and molecular mimicry? Medical Hypotheses 2004; 62(5): 646-52.
1436. Standaert BA, Curran D, Postma MJ. Budget constraint and vaccine dosing: a mathematical modelling exercise. Cost Effectiveness & Resource Allocation 2014; 12(1): 1-19.
1437. Stavropoulou E. Neuropathic pain prevention: Current strategies. Regional Anesthesia and Pain Medicine 2018; 43(7 Supplement 1): e33-e5.
1438. Stec MJ, Thalacker-Mercer A, Mayhew DL, et al. Randomized, four-arm, dose-response clinical trial to optimize resistance exercise training for older adults with age-related muscle atrophy. Exp Gerontol 2017; 99: 98-109.

1439. Steel JL, Bress K, Popichak L, et al. A systematic review of randomized controlled trials testing the efficacy of psychosocial interventions for gastrointestinal cancers. Journal of Gastrointestinal Cancer 2014; 45(2): 181-9.
1440. Stenger T, Ledo A, Ziller C, et al. Timing of Vaccination after Training: Immune Response and Side Effects in Athletes. Medicine & Science in Sports & Exercise 2020; 52(7): 1603-9.
1441. Stepanchuk VA. The screening of immune donors in organized collectives. Likars'ka sprava / Ministerstvo okhorony zdorov'ia Ukrainy 1998; (1): 107-9.
1442. Stevenson EM, Huang S-H, Jones RB, et al. No evidence that ongoing HIV-Specific immune responses contribute to persistent inflammation and immune activation in persons on long-term ART. medRxiv 2021.
1443. Stock I. Molluscum contagiosum - A common but poorly understood "childhood disease" and sexually transmitted illness. Medizinische Monatsschrift fur Pharmazeuten 2013; 36(8): 282-90.
1444. Stoffey WJ. Diabetes mellitus: current treatments and future prospects... recertification series. Physician Assistant 1996; 20(1): 23-32.
1445. Stoner L, Hanson ED, Khosravi N, Farajivafa V. Exercise training, circulating cytokine levels and immune function in cancer survivors: A meta-analysis. Brain, Behavior, and Immunity 2019; 81: 92-104.
1446. Strober L. Laughing matters: The role of humor on psychological wellbeing, health and quality of life in multiple sclerosis (MS). Multiple Sclerosis Journal 2017; 23(3 Supplement 1): 420-1.
1447. Struchiner CJ, Halloran ME, Brunet RC, Ribeiro JM, Massad E. Malaria vaccines: lessons from field trials. Cad Saude Publica 1994; 10 Suppl 2: 310-26.
1448. Sudfeld CR, Mugusi F, Aboud S, Nagu TJ, Wang M, Fawzi WW. Efficacy of vitamin D3 supplementation in reducing incidence of pulmonary tuberculosis and mortality among HIV-infected Tanzanian adults initiating antiretroviral therapy: study protocol for a randomized controlled trial. Trials 2017; 18(1) (no pagination).
1449. Sukumar BS, Tripathy TB, Shashirekha HK, Shetty SK. Efficacy of ashwagandha (Withania somnifera [l.] dunal) in improving cardiorespiratory endurance (vo2 max test) in healthy subjects. International Journal of Research in Pharmaceutical Sciences 2021; 12(1): 911-8.
1450. Sun AC, Alvarez-Fontecilla E, Venkatesh AG, Aronoff-Spencer E, Hall DA. High-Density Redox Amplified Coulostatic Discharge-Based Biosensor Array. IEEE J Solid-State Circuits 2018; 53(7): 2054-64.
1451. Sun Q, Zhong Y, Wu F, et al. Immunotherapy using slow-cycling tumor cells prolonged overall survival of tumor-bearing mice. BMC Medicine 2012; 10(1): 172-.
1452. Sun YI, Pence BD, Wang SS, Woods JA. Effects of Exercise on Stress-induced Attenuation of Vaccination Responses in Mice. Medicine & Science in Sports & Exercise 2019; 51(8): 1635-41.
1453. Sureda A, Busquets-Cortes C, Capo X, et al. Nutritional intervention based on mediterranean diet reduces blood pressure and immune cells response after immune stimulation. Annals of Nutrition and Metabolism 2017; 71(1-2): 70-1.
1454. Sureda A, Tur JA, Pons A, Cordova A. Immune response to exercise in elite sportsmen during the competitive season. Journal of Physiology and Biochemistry 2010; 66(1): 1-6.
1455. Suzuki K. Chronic inflammation as an immunological abnormality and effectiveness of exercise. Biomolecules 2019; 9(6): 223.
1456. Suzuki K. Recent progress in applicability of exercise immunology and inflammation research to sports nutrition. Nutrients 2021; 13(12): 4299.
1457. Swain M. Effectiveness of breathing exercise as play way method on cardiopulmonary parameters among children (3-12 years) with LRTI (lower respiratory tract infections) at tertiary care hospital, Bhubaneswar. European Journal of Molecular and Clinical Medicine 2020; 7(11): 1114-20.
1458. Swinbourne R, Miller J, Smart D, Dulson DK, Gill N. The Effects of Sleep Extension on Sleep, Performance, Immunity and Physical Stress in Rugby Players. Sports (Basel) 2018; 6(2).

1459. Swindt C, Zaldivar F, Eliakim A, Casali P, Cooper DM. Reduced tetanus antibody titers in overweight children. Autoimmunity 2006; 39(2): 137-41.
1460. Tajima Y, Yaguchi H, Mito Y, Iwasaki S, Fukasawa Y. TBE Clinical Features: Clinico-pathological features of a Japanese case from central Hokkaido Island. Clinical Neurology 2018; 58(Supplement 1): S13.
1461. Takahashi H, Nagata M, Nagata T, Mori K. Association of organizational factors with knowledge of effectiveness indicators and participation in corporate health and productivity management programs. Journal of occupational health 2021; 63(1): e12205.

1462. Takatsuki K, Yanagihara T, Egashira A, et al. A Rare Case of Pembrolizumab-Induced Dermatomyositis in a Patient with Cancer of Unknown Primary Origin. Am J Case Rep 2021; 22: e930286.
1463. Takha E, Kazarian G, Valeeva A, et al. Causal risk and protective factors in rheumatoid arthritis: A genetic update. Journal of Translational Autoimmunity 2021; 4: 100119.
1464. Talaei M, Greenig M, Perdek N, et al. Determinants of pre-vaccination antibody responses to SARS-CoV-2: A population-based longitudinal study (COVIDENCE UK). medRxiv 2021.
1465. Talwar GP, Pal R, Singh O, et al. Safety of intrauterine administration of purified neem seed oil (Praneem Vilci) in women & effect of its co-administration with the heterospecies dimer birth control vaccine on antibody response to human chorionic gonadotropin. Indian J Med Res 1995; 102: 66-70.
1466. Tang H, Fang Z, Saborio GP, Xiu Q. Efficacy and Safety of OM-85 in Patients with Chronic Bronchitis and/or Chronic Obstructive Pulmonary Disease. Lung 2015; 193(4): 513-9.
1467. Tang SN, Jiang P, Kim S, Zhang J, Jiang C, Lü J. Interception Targets of Angelica Gigas Nakai Root Extract versus Pyranocoumarins in Prostate Early Lesions and Neuroendocrine Carcinomas in TRAMP Mice. Cancer Prev Res (Phila) 2021; 14(6): 635-48.
1468. Tania C, Emilie Z, Bernard B, et al. Feasibility, acceptability and effectiveness of integrated care for COPD patients: a mixed methods evaluation of a pilot community-based programme. Swiss Medical Weekly 2017; 147(49-50): w14567.
1469. Tankova T, Dakovska L, Kirilov G, Koev D. Insulin secretion and anti-GAD65 antibodies in subjects with impaired glucose tolerance. Experimental & Clinical Endocrinology & Diabetes 2001; 109(7): 355-60.
1470. Tanny A. Recapturing the immune response. Joe Weider's Muscle & Fitness 1993; 54(10): 48.
1471. Tedjaseputra A, Raj K, De Lavallade H, et al. Anti-phospholipase A2 receptor (Anti-PLA2R) antibody positive-nephrotic syndrome as a manifestation of multi-system, immune-mediated complications post allogeneic stem cell transplant: A case report. British Journal of Haematology 2016; 173(Supplement 1): 167.
1472. Teixeira AM, Rama L, Martins M, Cunha MR. KINETIC RESPONSE OF SALIVARY IGA TO SEVERAL EXERCISE PROTOCOLS PERFORMED BY WELL TRAINED SWIMMERS. Revista Portuguesa de Ciencias do Desporto 2006; 6(2 Suppl): 177-9.
1473. Teljeur C, Ryan M. Extrapolating ICERs at different discount rates. International journal of technology assessment in health care 2019; 35: 93-.
1474. Tenner-Racz K, Stellbrink HJ, van Lunzen J, et al. The unenlarged lymph nodes of HIV-1-infected, asymptomatic patients with high CD4 T cell counts are sites for virus replication and CD4 T cell proliferation. The impact of highly active antiretroviral therapy. J Exp Med 1998; 187(6): 949-59.
1475. Terlinden A, Renson B, Poirrier JE, Curran D, Ponder C. Carbon cost-effectiveness of cocooning immunization against pertussis in England and wales: An ecological perspective. Value in Health 2013; 16(7): A578.
1476. Tesfaye Yifru G, Yazie Derso B, Zewdie Shibeshi A. Assessment of Government Intervention Effectiveness in Scrubbing COVID-19 Pandemic and Its Pros and Cons on Educational Activities, the Case of Dire Dawa City. J Multidiscip Healthc 2021; 14: 2669-93.

1477. Thielecke F, Blannin A. Omega-3 Fatty Acids for Sport Performance—Are They Equally Beneficial for Athletes and Amateurs? A Narrative Review. Nutrients 2020; 12(12): 3712.
1478. Thomas M, Slowikowski K, Nasrallah M, et al. 318 SINGLE CELL DISSECTION OF THE TRANSCRIPTIONAL LANDSCAPE DEFINING CHECKPOINT INHIBITOR-ASSOCIATED COLITIS OPENS A WINDOW INTO IMMUNE DYSREGULATION IN THE HUMAN COLON. Gastroenterology 2020; 158(6 Supplement 1): S-57.
1479. Thomsen M, Vitetta L, Clarke S. Intestinal microbiome and chemotherapy-induced neutropenia. Asia-Pacific Journal of Clinical Oncology 2019; 15(SUPPL 9): 108.
1480. Thonier VL, Vrignaud C, Peyrard T, et al. A large deletion within the ART4 gene causing a Donull phenotype in a French patient/family of Northern African descent. Vox Sanguinis 2020; 115(SUPPL 1): 266.
1481. Tian D, Zhu X, Xue R, Zhao P, Yao Y. Case 259: Primary central nervous system lymphomatoid granulomatosis mimicking chronic lymphocytic inflammation with pontine perivascular enhancement responsive to steroids (CLIPPERS). Radiology 2018; 289(2): 572-7.
1482. Tiffreau V, Rannou F, Kopciuch F, et al. Postrehabilitation Functional Improvements in Patients With Inflammatory Myopathies: The Results of a Randomized Controlled Trial. Archives of Physical Medicine & Rehabilitation 2017; 98(2): 227-34.
1483. Tinazzi E, Confente F, Tacchella G, Patuzzo G, Lunardi C. Lung ultrasonography: Possible role as predictor of pulmonary involvement in patients affected by systemic sclerosis complicated by either interstitial lung disease or pulmonary arterial hypertension. Journal of Scleroderma and Related Disorders 2018; 3(Supplement 1): 75.
1484. Tipparaju VV, Xian X, Bridgeman D, et al. Reliable Breathing Tracking with Wearable Mask Device. IEEE Sens J 2020; 20(10): 5510-8.
1485. Tito PAL, Bernardino TCS, Bellozi PMQ, et al. Cannabidiol prevents lipopolysaccharide-induced sickness behavior and alters cytokine and neurotrophic factor levels in the brain. Pharmacol Rep 2021; 73(6): 1680-93.
1486. Tobin DJ. Characterization of hair follicle antigens targeted by the anti-hair follicle immune response. Journal of Investigative Dermatology Symposium Proceedings 2003; 8(2): 176-81.
1487. Toffoli EC, Sweegers MG, Bontkes HJ, et al. Effects of physical exercise on natural killer cell activity during (neo)adjuvant chemotherapy: A randomized pilot study. Physiol Rep 2021; 9(11): e14919.
1488. Toki M, Wong PF, Kluger H, et al. High-plex predictive marker discovery for melanoma immunotherapy treated patients using NanoString Digital Spatial Profiling. Journal for ImmunoTherapy of Cancer 2018; 6(Supplement 1).
1489. Tokuhara D. Role of the Gut Microbiota in Regulating Non-alcoholic Fatty Liver Disease in Children and Adolescents. Front Nutr 2021; 8: 700058.
1490. Tomar R, Hamdan M, Al-Qahtani MH. Effect of low to moderate intensity walking and cycling on glycaemic and metabolic control in type 1 diabetes mellitus adolescent males: A randomized controlled trial. Isokinetics & Exercise Science 2014; 22(3): 237-43.
1491. Tomás CC, Oliveira E, Sousa D, et al. Proceedings of the 3rd IPLeiria's International Health Congress : Leiria, Portugal. 6-7 May 2016. BMC Health Serv Res 2016; 16 Suppl 3(Suppl 3): 200.
1492. Tomasi TB, Trudeau FB, Czerwinski D, Erredge S. Immune parameters in athletes before and after strenuous exercise. J Clin Immunol 1982; 2(3): 173-8.
1493. Topping KD, Kelly DG. Investigation of binding characteristics of immobilized toll-like receptor 3 with poly(I:C) for potential biosensor application. Anal Biochem 2019; 564-565: 133-40.
1494. Torvinen S, Nieminen P, Paavonen J, Lehtinen M, Demarteau N, Hahl J. Cost effectiveness of prophylactic HPV 16/18 vaccination in Finland: Results from a modelling exercise. Journal of Medical Economics 2010; 13(2): 284-94.
1495. Tramontano M, De Angelis S, Galeoto G, et al. Physical Therapy Exercises for Sleep Disorders in a Rehabilitation Setting for Neurological Patients: A Systematic Review and Meta-Analysis. Brain Sci 2021; 11(9).

1496. Traylor CS, Johnson JD, Kimmel MC, Manuck TA. Effects of psychological stress on adverse pregnancy outcomes and nonpharmacologic approaches for reduction: an expert review. American Journal of Obstetrics and Gynecology MFM 2020; 2(4): 100229.
1497. Treanor C, Kyaw T, Donnelly M. An international review and meta-analysis of prehabilitation compared to usual care for cancer patients. Journal of cancer survivorship : research and practice 2018; 12(1): 64-73.
1498. Treurnicht FK, Seleka M, Kleynhans J, et al. Outbreak of influenza a in a boarding school in South Africa, 2016. Pan African Medical Journal 2019; 33: 42.
1499. Triplett-McBride NT, McBride JM, Bush JA, et al. Plasma proenkephalin peptide F and human B cell responses to exercise stress in fit and unfit women. Peptides 1998; 19(4): 731-8.
1500. Trochimiak T, Hübner-Woźniak E. EFFECT OF EXERCISE ON THE LEVEL OF IMMUNOGLOBULIN A IN SALIVA. Biology of Sport 2012; 29(4): 255-61.
1501. Tromp N, Baltussen R. Mapping of multiple criteria for priority setting of health interventions: an aid for decision makers. BMC Health Services Research 2012; 12(1): 454-.
1502. Trushina EN, Riger NA, Mustafina OK, et al. The efficiency of branched chain aminoacids (BCAA) in the nutrition of combat sport athletes. Voprosy pitaniia 2019; 88(4): 48-56.
1503. Trushina EN, Riger NA, Mustafina OK, et al. Immunomodulating effects of using L-carnitine and coenzyme Q10 in the nutrition of junior athletes. Voprosy pitaniia 2019; 88(2): 40-9.
1504. Trushina EN, Vybornov VD, Riger NA, et al. [The efficiency of branched chain aminoacids (BCAA) in the nutrition of combat sport athletes]. Vopr Pitan 2019; 88(4): 48-56.
1505. Tschumper RC, Walters DK, Osborne CA, et al. RNA-Seq Based Immunoglobulin Repertoire Analysis of Normal Plasma Cells Generated in an in Vitro B Cell Differentiation System. Blood 2019; 134(Supplement 1): 1051.
1506. Tsinti M, Dermentzoglou V, Tsitsami E, Ntokou A. Spondyloenchondrodysplasia in a female toddler presenting as systemic lupus erythematosus. Pediatric Rheumatology 2021; 19(SUPPL 1).
1507. Tulio P, Chiodo L, Kraig E, Curiel T, Wu Y, Kellogg D. Effect of mtor inhibition on immunological responses, muscle strength, and physical performance in octogenarians. Journal of the American Geriatrics Society 2014; 62(SUPPL. 1): S246.
1508. Turner JE. Is immunosenescence influenced by our lifetime "dose" of exercise? Biogerontology 2016; 17(3): 581-602.
1509. Uchida Y, Gherardini J, Bertolini M, et al. 396 Vdelta1+T-cells are stress-sentinels in human skin and are implicated in alopecia areata pathogenesis. Journal of Investigative Dermatology 2016; 136(9 Supplement 2): S228.
1510. Uhr JW, Marches R. Dormancy in a model of murine B cell lymphoma. Seminars in Cancer Biology 2001; 11(4): 277-83.
1511. Ulrich RG. Idiosyncratic toxicity: A convergence of risk factors. Annual Review of Medicine 2007; 58: 17-34.
1512. Unal M, Erdem S, Deniz G. The effects of chronic aerobic and anaerobic exercises n lymphocyte subgroups. Acta Physiol Hung 2005; 92(2): 163-71.
1513. Underwood JM, Townsend JS, Stewart SL, et al. Surveillance of Demographic Characteristics and Health Behaviors Among Adult Cancer Survivors -- Behavioral Risk Factor Surveillance System, United States, 2009. MMWR Surveillance Summaries 2012; 61(1): 1-23.
1514. Upham JW, Lundahl J, Liang H, Denburg JA, O'Byrne PM, Snider DP. Simplified quantitation of myeloid dendritic cells in peripheral blood using flow cytometry. Cytometry 2000; 40(1): 50-9.
1515. ur Rehman A, Hassali MAA, Abbas S, et al. Pharmacological and non-pharmacological management of COPD; limitations and future prospects: a review of current literature. Journal of Public Health (09431853) 2020; 28(4): 357-66.
1516. Urist MR, Hudak RT, Huo YK, Rasmussen JK. Osteoporosis: a bone morphogenetic protein auto-immune disorder. Progress in clinical and biological research 1985; 187: 77-96.

1517. Ushakov AV, Ivanchenko VS, Gagarina AA. Psychological stress in pathogenesis of essential hypertension. Current Hypertension Reviews 2016; 12(3): 203-14.
1518. Valizadeh R, Karampour S, Saiiari A, Sadeghi S. The effect of one bout submaximal endurance exercise on the innate and adaptive immune responses of hypertensive patients. J Sports Med Phys Fitness 2022; 62(2): 244-9.
1519. Valve P, Lehtinen-Jacks S, Eriksson T, et al. LINDA - a solution-focused low-intensity intervention aimed at improving health behaviors of young females: a cluster-randomized controlled trial. BMC public health 2013; 13: 1044.
1520. van Aken WG, Brand A, van der Poel CL. [Leukodepletion of blood products: a requirement for improvement of quality and safety]. Ned Tijdschr Geneeskd 2000; 144(22): 1033-6.
1521. Van Alfen N, Van Engelen BGM, Hughes RAC. Treatment for idiopathic and hereditary neuralgic amyotrophy (brachial neuritis). Cochrane Database of Systematic Reviews 2009; (3): CD006976.
1522. Van Der Noordaa J, Houweling H. Grounds for the inclusion of vaccination against cervical cancer within the National Immunisation Programme. Nederlands Tijdschrift voor Geneeskunde 2008; 152(42): 2267-9.
1523. Van Hemelrijck M, Karagiannis SN, Rohrmann S. Atopy and prostate cancer: Is there a link between circulating levels of IgE and PSA in humans? Cancer Immunology, Immunotherapy 2017; 66(12): 1557-62.
1524. van Lieshout J, Wensing M, Grol R, van Lieshout J, Wensing M, Grol R. Improvement of primary care for patients with chronic heart failure: a pilot study. BMC Health Services Research 2010; 10: 8-.
1525. van Noort SP, Gomes MGM, Codeco CT, Koppeschaar CE, van Ranst M, Paolotti D. Ten-year performance of Influenzanet: ILI time series, risks, vaccine effects, and care-seeking behaviour. Epidemics 2015; 13: 28-36.
1526. van Ree R, Poulsen LK, Wong GWk, Ballmer-Weber BK, Gao Z, Jia X. Food allergy:definitions, prevalence, diagnosis and therapy. Zhonghua yu fang yi xue za zhi [Chinese journal of preventive medicine] 2015; 49(1): 87-92.
1527. Van Schaik IN, Winer JB, De Haan R, Vermeulen M. Intravenous immunoglobulin for chronic inflammatory demyelinating polyradiculoneuropathy. Cochrane database of systematic reviews (Online) 2002; (2): CD001797.
1528. Vanage GR, Jaiswal YK, Lu YA, Tam JP, Wang LF, Koide SS. Immunization with synthetic peptide segments of a sperm protein impair fertility in rats. Res Commun Chem Pathol Pharmacol 1994; 84(1): 3-15.
1529. Vang KB, Dings RP. 3D Antibody Modeling: Changing Students' Perspective of Structure to Function. Molecular Biology of the Cell 2013; 24(24).
1530. Varadarajan I, Lee DW. Management of T-Cell Engaging Immunotherapy Complications. Cancer Journal 2019; 25(3): 223-30.
1531. Vargas L, Dalvi N, Karkare S. Asynchronous leg jerking with normal sensorium as a presentation of frontal lobe seizures. Epilepsy Currents 2015; 15(SUPPL. 1): 240.
1532. Vedenko A, Panara K, Goldstein G, Ramasamy R, Arora H. Tumor Microenvironment and Nitric Oxide: Concepts and Mechanisms. Adv Exp Med Biol 2020; 1277: 143-58.
1533. Vedhara K, Sunger K, Avery A, et al. Effects of non-pharmacological interventions as vaccine adjuvants in humans: a systematic review and network meta-analysis. Health psychology review 2021; 15(2): 245-71.
1534. Veerabattini N, Niven AS. Beyond young's syndrome: Diagnosis, management, and prognosis of primary ciliary dyskinesia. American Journal of Respiratory and Critical Care Medicine 2019; 199(9).
1535. Velica P, Gojkovic M, Wulliman D, et al. Cytotoxic t-cells mediate exercise-induced reductions in tumor growth. eLife 2020; 9: 1-25.

1536. Veljkovic V, Metlas R, Jevtovic D, et al. The role of passive immunization in hiv-positive patients : a case report. CHEST 2001; 120(2): 662-6.
1537. Vellas B. Preventive drug trials for Alzheimer's disease. Neurobiology of Aging 2014; 35(SUPPL. 1): S23-S4.
1538. Venkatesh A, Edirappuli SD, Zaman HP, Zaman R. The Effect of Exercise on Mental Health: A Focus on Inflammatory Mechanisms. Psychiatr Danub 2020; 32(Suppl 1): 105-13.
1539. Vera Perez E, Cervantes Trujano E, Andaluz Garza C, et al. The safety profile of human dialyzable leukocyte extract transferon used as an adjuvant in patients with autoimmune disease and its effect in rheumatoid arthritis. Journal of Clinical Rheumatology 2020; 20(3 SUPPL 1): S131.
1540. Verma V. Economic sustainability of immune-checkpoint inhibitors: the looming threat. Nature Reviews Clinical Oncology 2018; 15(12): 721-2.
1541. Verscaj C, Jeng M, Lo L. Neonatal polycythemia associated with nonimmune thrombocytopenia. Pediatric Blood and Cancer 2021; 68(SUPPL 3): S119-S20.
1542. Versteegen P, Berbers GAM, Smits G, et al. More than 10 years after introduction of an acellular pertussis vaccine in infancy: a cross-sectional serosurvey of pertussis in the Netherlands. Lancet Reg Health Eur 2021; 10: 100196.
1543. Vetrano M, Wegman A, Koes B, Mehta S, King CA. Serum IL-1RA levels increase from follicular to luteal phase of the ovarian cycle: A pilot study on human female immune responses. PLoS One 2020; 15(9): e0238520.
1544. Vetvicka V, Vetvickova J. Effects of glucan on immunosuppressive actions of mercury. Journal of Medicinal Food 2009; 12(5): 1098-104.
1545. Viitala PE, Newhouse IJ, LaVoie N, Gottardo C. The effects of antioxidant vitamin supplementation on resistance exercise induced lipid peroxidation in trained and untrained participants. Lipids Health Dis 2004; 3: 14.
1546. Vijaykumar T, DeAngelo DJ, Frede J, et al. Single-cell RNA-seq reveals developmental plasticity with coexisting oncogenic states and immune evasion programs in ETP-ALL. Blood 2021; 137(18): 2463-80.
1547. Vilas-Boas F, Feitosa GS, Soares MB, et al. Bone marrow cell transplantation in Chagas' disease heart failure: report of the first human experience. Arq Bras Cardiol 2011; 96(4): 325-31.
1548. Vilela MMdS. Human Inborn Errors of Immunity (HIEI): predominantly antibody deficiencies (PADs): if you suspect it, you can detect it. Jornal de Pediatria 2021; 97(Supplement 1): S67-S74.
1549. Villasana M, Ochoa G, Aguilar S. Modeling and optimization of combined cytostatic and cytotoxic cancer chemotherapy. Artif Intell Med 2010; 50(3): 163-73.
1550. Vinci MR, Camisa V, Santoro A, Zaffina S, Dalmasso G, Raponi M. Interventions and indicators of health promotion in a complex work reality. Journal of Preventive Medicine and Hygiene 2019; 60(3 Supplement 1): E26-E8.
1551. Visalakshi S, Mohan S. Evaluation of biotechnology-based healthcare products for prioritization in Indian context. Int J Technol Assess Health Care 2002; 18(3): 718-27.
1552. Vitlic A, Phillips AC, Gallagher S, Oliver C, Lord JM, Moss P. Anticytomegalovirus antibody titres are not associated with caregiving burden in younger caregivers. British journal of health psychology 2015; 20(1): 68-84.
1553. Vujicic AD, Gemovic B, Veljkovic V, Glisic S, Veljkovic N. Natural autoantibodies in healthy neonatals recognizing a peptide derived from the second conserved region of HIV-1 gp120. Vojnosanitetski Pregled 2014; 71(4): 352-61.
1554. Vyas GN. Human peripheral blood mononuclear cell substrate for propagating wild type HIV-1. Developments in biologicals 2001; 106.
1555. Wagner KH. Antioxidants in Sport Nutrition: All the Same Effectiveness? In: Lamprecht M, ed. Antioxidants in Sport Nutrition. Boca Raton (FL): CRC Press/Taylor & Francis
© 2015 by Taylor & Francis Group, LLC.; 2015.

1556. Wahab S, Ahmad MF, Hussain A, Usmani S, Shoaib A, Ahmad W. Effectiveness of Azithromycin as add-on Therapy in COVID-19 Management. Mini reviews in medicinal chemistry 2021.
1557. Wahlqvist ML. Enabling and disabling health systems through food systems. Annals of Nutrition and Metabolism 2017; 71(Supplement 2): 14-5.
1558. Wakeda T, Heike Y, Nishida S, et al. Novel cancer therapy and patient's quality of life: Camouflage makeup for unsightly skin reactions caused by cancer vaccine therapy. Supportive Care in Cancer 2011; 19(2 SUPPL. 1): S180.
1559. Wall LA, Wisner EL, Gipson KS, Sorensen RU. Bronchiectasis in Primary Antibody Deficiencies: A Multidisciplinary Approach. Front Immunol 2020; 11: 522.
1560. Wallace Z, Khosroshahi A, Carruthers M, et al. An international, multi-specialty validation study of the IgG4-related disease responder index. Arthritis and Rheumatology 2016; 68(Supplement 10): 1237-8.
1561. Wallach JC, Delpino MV, Scian R, Fossati CA, Baldi PC, Deodato B. Prepatellar bursitis due to Brucella abortus: Case report and analysis of the local immune response. Journal of Medical Microbiology 2010; 59(12): 1514-8.
1562. Walsh NP. Exercising in Environmental Extremes: A Greater Threat to Immune Function? Sports Medicine 2006; 36(11): 941-76.
1563. Walsmith J, Roubenoff R. Cachexia in rheumatoid arthritis. International Journal of Cardiology 2002; 85(1): 89-99.
1564. Wan Y-M, Wang Y-C, Xu J-Q. HIV/AIDS vaccine development: Are we walking out from the dark? Chinese Medical Journal 2010; 123(23): 3489-94.
1565. Wang C-W, Ho AHY, Ho RTH, et al. The effect of t'ai chi exercise on immunity and infections: A systematic review of controlled trials. Journal of Alternative and Complementary Medicine 2013; 19(5): 389-96.
1566. Wang K, Harnden A. Pertussis-induced cough. Pulmonary Pharmacology and Therapeutics 2011; 24(3): 304-7.
1567. Wang L, Zhong H, Zhang FX, Mei J, Li GQ, Xiao HB. Minimally invasive Nuss technique allows for repair of recurrent pectus excavatum following the Ravitch procedure: report of 12 cases. Surg Today 2011; 41(8): 1156-60.
1568. Wang L, Zhou Y, Yu W, Li P. Pre-existing cancer exacerbates cerebral ischemic stroke in mice via regulatory T cell redistribution. Stroke 2017; 48(Supplement 1).
1569. Wang N, Guo Y. Traditional Chinese Practice, A Promising Integrative Intervention for Chronic Non-Infectious Disease Management. Chinese Journal of Integrative Medicine 2018; 24(12): 886-90.
1570. Wang P, Zhang S, Lv H, et al. Clinical efficacy of gamma-globulin combined with dexamethasone and methylprednisolone, respectively, in the treatment of acute transverse myelitis and its effects on immune function and quality of life. Experimental and Therapeutic Medicine 2020; 20(5): 9234.
1571. Wang P, Zhang S, Lv H, et al. Clinical efficacy of γ-globulin combined with dexamethasone and methylprednisolone, respectively, in the treatment of acute transverse myelitis and its effects on immune function and quality of life. Exp Ther Med 2020; 20(5): 104.
1572. Wang RT, Liu XG, Hou Y, Hou M. A physician-patient survey for primary immune thrombocytopenia: Chinese subgroup analysis of I-WISh International Survey. Zhonghua xue ye xue za zhi = Zhonghua xueyexue zazhi 2021; 42(5): 369-75.
1573. Wanke K, Von Braun A, Haeberli L, et al. Cytomegalovirus and immune senescence: Immunogenicity of tick-borne encephalitis vaccine is impaired in CMV-infected elderly individuals. Clinical Microbiology and Infection 2012; 18(SUPPL. 3): 61.
1574. Waters-Banker C, Dupont-Versteegden EE, Kitzman PH, Butterfield TA. Investigating the Mechanisms of Massage Efficacy: The Role of Mechanical Immunomodulation. Journal of Athletic Training (Allen Press) 2014; 49(2): 266-73.

1575. Weaver JA, Kohut ML, Colbert LH, Ghaffar A, Mayer EP, Davis JM. Immune system activation and fatigue during treadmill running: Role of interferon. Medicine and Science in Sports and Exercise 1998; 30(6): 863-8.
1576. Webster J, Theodoratou E, Nair H, et al. An evaluation of emerging vaccines for childhood pneumococcal pneumonia. BMC Public Health 2011; 11(Suppl 3): S26-S.
1577. Wei KC, Huang MS, Chang TH. Dengue Virus Infects Primary Human Hair Follicle Dermal Papilla Cells. Front Cell Infect Microbiol 2018; 8: 268.
1578. Wei P, Wang K, Luo C, et al. Cordycepin confers long-term neuroprotection via inhibiting neutrophil infiltration and neuroinflammation after traumatic brain injury. Journal of Neuroinflammation 2021; 18(1): 1-17.
1579. Wei WE, Fook-Chong S, Chen WK, Chlebicki MP, Gan WH. The impact of healthcare worker influenza vaccination on nosocomial influenza in a tertiary hospital: an ecological study. BMC health services research 2020; 20(1): 636.
1580. Werner L, Paclik D, Berndt U, Sturm A. Linking TNFalpha inhibitors and Notch-1: Novel implications in inflammatory bowel diseases. Journal of Crohn's and Colitis 2011; 5(1): S27.
1581. Werner L, Sturm A, Reinhold D, Roggenbuck D. Identification of glycoprotein 2 as an immunomodulator of innate and adaptive immune responses. Journal of Crohn's and Colitis 2012; 6(SUPPL. 1): S1.
1582. Weyh C, Kruger K, Strasser B. Physical activity and diet shape the immune system during aging. Nutrients 2020; 12(3): 622.
1583. White CM, Robert-Lewis SF, Petty JL, Hadden RD, McCrone PR. Observer blind randomised controlled trial of a tailored home exercise programme versus usual care in people with stable inflammatory immune mediated neuropathy. BMC Neurology 2015; 15(1): 147.
1584. Whitehouse WG, Dinges DF, Orne EC, et al. Psychosocial and immune effects of self-hypnosis training for stress management throughout the first semester of medical school. Psychosomatic Medicine 1996; 58(3): 249-63.
1585. Wild CP. Preventable exposures associated with human cancer. Cancer Research 2013; 73(8 SUPPL. 1).
1586. Williams C. Guidelines for Training Individuals With Lupus. Strength & Conditioning Journal 2007; 29(2): 56-8.
1587. Williams MH. Dietary supplements and sports performance: minerals. J Int Soc Sports Nutr 2005; 2(1): 43-9.
1588. Williams NC, Killer SC, Svendsen IS, Jones AW. Immune nutrition and exercise: Narrative review and practical recommendations. European journal of sport science 2019; 19(1): 49-61.
1589. Williams T, Currie MJ, Dachs GU, et al. Effects of exercise and anti-PD-1 on the tumour microenvironment. Immunology Letters 2021; 239: 60-71.
1590. Willmott AGB, Hayes M, James CA, Dekerle J, Gibson OR, Maxwell NS. Once- and twice-daily heat acclimation confer similar heat adaptations, inflammatory responses and exercise tolerance improvements. Physiol Rep 2018; 6(24): e13936.
1591. Wilson J. Neuroimmunology of dancing eye syndrome in children. Developmental Medicine and Child Neurology 2006; 48(8): 693-6.
1592. Wilson N, Grout L, Summers J, et al. Should prioritising health interventions be informed by modelling studies? The case of cancer control in Aotearoa New Zealand. The New Zealand medical journal 2021; 134(1531): 101-13.
1593. Wiskemann J. Exercise in the setting of hematopoietic stem cell transplantation. European Reviews of Aging & Physical Activity 2013; 10(1): 15-8.
1594. Wiskemann J, Huber G. Physical exercise as adjuvant therapy for patients undergoing hematopoietic stem cell transplantation. Bone Marrow Transplantation 2008; 41(4): 321-9.
1595. Wiskemann J, Hummler S, Diepold C, et al. POSITIVE study: physical exercise program in non-operable lung cancer patients undergoing palliative treatment. BMC Cancer 2016; 16: 1-9.

1596. Wolbing F, Kaesler S, Kempf W, et al. Co-factor dependent anaphylaxis driven by innate immune signals is mediated by basophils. Experimental Dermatology 2011; 20(2): 181-2.
1597. Wolbing F, Kaesler S, Kempf W, et al. Innate immune signals act as co-factors modulating basophil and IgG1 dependent anaphylaxis. Allergy: European Journal of Allergy and Clinical Immunology 2013; 68(SUPPL. 97): 105.
1598. Wolbing F, Kaesler S, Kempf W, et al. Anaphylaxis triggered by innate immune signals as co-factors is mediated by basophils independent of IgE. Journal of Investigative Dermatology 2013; 133(SUPPL. 1): S5.
1599. Wolbing F, Kaesler S, Kempf W, et al. Innate immune signals trigger basophil dependent co-factor induced anaphylaxis independent of IgE. Experimental Dermatology 2013; 22(3): e3.
1600. Wolbing F, Kaesler S, Kempf W, et al. Innate immune signals trigger co-factor dependent anaphylaxis by modulating the IgG1 dependent activation of basophils. Allergo Journal 2013; 22(6): 394.
1601. Wolbing F, Kempf WE, Koberle M, et al. Specific TLR activation mediates innate immune signal triggered co-factor dependent anaphylaxis. Experimental Dermatology 2015; 24(3): E1.
1602. Wollen KA. Alzheimer's disease: The pros and cons of pharmaceutical, nutritional, botanical, and stimulatory therapies, with a discussion of treatment strategies from the perspective of patients and practitioners. Alternative Medicine Review 2010; 15(3): 223-44.
1603. Wolver S, Fadden P. An unusal case of satin-induced myopathy. Journal of General Internal Medicine 2015; 30(SUPPL. 2): S360.
1604. Wong ND, Eisenberg A, Cecere J, Patao C, Eisenberg H. Efficacy of a cardiovascular behavioral intervention program on progression of atherosclerosis. Cardiology (switzerland) 2016; 134: 234-.
1605. Wong SY, Wong CK, Chan FW, et al. Chronic psychosocial stress: does it modulate immunity to the influenza vaccine in Hong Kong Chinese elderly caregivers? Age (Dordr) 2013; 35(4): 1479-93.
1606. Wood M, Duryea T. Promoting early childhood literacy: What do pediatric residents know, believe, and practice? Pediatrics 2018; 141(1).
1607. Worth A, Sheikh A. Prevention of anaphylaxis in healthcare settings. Expert review of clinical immunology 2013; 9(9): 855-69.
1608. Wu C, Gao R, Zhang D, Han S, Zhang Y. PRWHMDA: Human Microbe-Disease Association Prediction by Random Walk on the Heterogeneous Network with PSO. International journal of biological sciences 2018; 14(8): 849-57.
1609. Wu GG, Kaplan C, Curtis B, Pearson H. Report on the 14th ISBT platelet immunology workshop. Vox Sanguinis 2010; 99(SUPPL. 1): 390.
1610. Wu H-Y, Tang C-F, He H. Orexin, orexin receptor and sports. Journal of Clinical Rehabilitative Tissue Engineering Research 2007; 11(39): 7966-9.
1611. Wullenweber M, Schneider U, Hagenah R. Myasthenia gravis associated with AIDS and neurosyphilis. Nervenarzt 1993; 64(4): 273-7.
1612. Wüllenweber M, Schneider U, Hagenah R. [Myasthenia gravis in AIDS and neurosyphilis]. Nervenarzt 1993; 64(4): 273-7.
1613. XaMeHoBa GS, kHa yH, BabaHha M, BoeHko GB, TkaeHko MB, daH BH. Evaluation of therapy efficiency in patients with combined course of copd and osteoarthritis. Wiadomosci lekarskie (Warsaw, Poland : 1960) 2016; 69(2): 214-8.
1614. Xie H, Yang X, Cao Y, Long X, Shang H, Jia Z. Role of lipoic acid in multiple sclerosis. CNS Neurosci Ther 2021.
1615. Xu K, Zhou B, Zheng X, et al. Protein panel for predicting the efficacy of neoadjuvant therapy for rectal cancer based on proteomics. Cancer Research 2020; 80(16 SUPPL).
1616. Xu Y, Yang B, Lin J, Ke J. Study of immunity target on the sports recipe of psychotherapy. Chinese Journal of Clinical Rehabilitation 2003; 7(4): 644-5.

1617. Xue-Jie Y, McBride JW, Walker DH. Genetic diversity of the 28-kilodalton outer membrane protein gene in human isolates of Ehrlichia chaffeensis. Journal of Clinical Microbiology 1999; 37(4): 1137-43.
1618. Yaacoub S, Khabsa J, El-Khoury R, et al. COVID-19 transmission during swimming-related activities: a rapid systematic review. BMC Infectious Diseases 2021; 21(1): 1112.
1619. Yada K, Nogami K. Spotlight on emicizumab in the management of hemophilia a: Patient selection and special considerations. Journal of Blood Medicine 2019; 10: 171-81.
1620. Yamaguchi S, Maruyama T, Miyashita K, et al. Severe osteomalacia caused by tubulointerstitial nephritis with fanconi syndrome accompanied with asymptomotic primary biliary cirrhosis. Endocrine Reviews 2014; 35(SUPPL. 3).
1621. Yang B, Yao H, Yang J, et al. In Situ Synthesis of Natural Antioxidase Mimics for Catalytic Anti-Inflammatory Treatments: Rheumatoid Arthritis as an Example. J Am Chem Soc 2022; 144(1): 314-30.
1622. Yang M. IL-6: A hack to body fat and exercise? Science Translational Medicine 2019; 11(476): eaaw5316.
1623. Yang S, Yu C, Chen P, et al. Protective immune barrier against hepatitis B is needed in individuals born before infant HBV vaccination program in China. Scientific reports 2015; 5: 18334.
1624. Yang WP, Green K, Pinz-Sweeney S, Briones AT, Burton DR, Barbas CF, 3rd. CDR walking mutagenesis for the affinity maturation of a potent human anti-HIV-1 antibody into the picomolar range. J Mol Biol 1995; 254(3): 392-403.
1625. Yang X, Li Z, Wang B, et al. Prognosis and antibody profiles in survivors of critical illness from COVID-19: a prospective multicentre cohort study. Br J Anaesth 2021.
1626. Yao H, Fu H, Yang B, et al. In Situ Synthesis of Natural Antioxidase Mimics for Catalytic Anti-Inflammatory Treatments: Rheumatoid Arthritis as an Example. Journal of the American Chemical Society 2021.
1627. Ye J, Tsang HWH, Cheung WM. The Neuroscience of Nonpharmacological Traditional Chinese Therapy (NTCT) for Major Depressive Disorder: A Systematic Review and Meta-Analysis. Evidence-based Complementary and Alternative Medicine 2019; 2019: 2183403.
1628. Yifru GT, Derso BY, Shibeshi AZ. Assessment of government intervention effectiveness in scrubbing covid-19 pandemic and its pros and cons on educational activities, the case of dire dawa city. Journal of Multidisciplinary Healthcare 2021; 14: 2669-93.
1629. Yokoi F, Yang G, Li J, DeAndrade MP, Zhou T, Li Y. Earlier onset of motor deficits in mice with double mutations in Dyt1 and Sgce. J Biochem 2010; 148(4): 459-66.
1630. Yoon DH, Han GY, Hwang SS, et al. The Effect of Fermented Porcine Placental Extract on Fatigue-Related Parameters in Healthy Adults: a Double-Blind, Randomized, Placebo-Controlled Trial. Nutrients 2020; 12(10).
1631. Yoon SS, Hwang KJ, Woo JT, Cho SS. Stiff person syndrome associated with Graves' disease and thymoma. European Journal of Neurology 2012; 19(SUPPL.1): 384.
1632. Yoshikawa T, Baba A, Akaishi M, et al. Immunoadsorption therapy for dilated cardiomyopathy using tryptophan column-A prospective, multicenter, randomized, within-patient and parallel-group comparative study to evaluate efficacy and safety. J Clin Apher 2016; 31(6): 535-44.
1633. Young LM, Motz VA, Markey ER, Young SC, Beaschler RE. Recommendations for Best Disinfectant Practices to Reduce the Spread of Infection via Wrestling Mats. Journal of athletic training 2017; 52(2): 82-8.
1634. Yu JY, Jung HY, Kim CH, Kim HS, Kim MO. Multiple cranial neuropathies without limb involvements: guillain-barre syndrome variant? Ann Rehabil Med 2013; 37(5): 740-4.
1635. Yu M, Chibalin AV, Krook A, Zierath JR, Blomstrand E. Marathon running increases ERK1/2 and p38 MAP kinase signalling to downstream targets in human skeletal muscle. Journal of Physiology 2001; 536(1): 273-82.
1636. Yu M, Stepto NK, Chibalin AV, et al. Metabolic and mitogenic signal transduction in human skeletal muscle after intense cycling exercise. J Physiol 2003; 546(Pt 2): 327-35.

1637. Yu P, Huang L, Zou J, et al. Immunization with recombinant Nogo-66 receptor (NgR) promotes axonal regeneration and recovery of function after spinal cord injury in rats. Neurobiol Dis 2008; 32(3): 535-42.
1638. Yu XJ, McBride JW, Walker DH. Genetic diversity of the 28-kilodalton outer membrane protein gene in human isolates of Ehrlichia chaffeensis. J Clin Microbiol 1999; 37(4): 1137-43.
1639. Yutzy B, Kruip C, Coulibaly C, et al. Early perturbation of peripheral blood lymphocyte subsets in BSE-infected non-human primates and evidence that an activated immune system contributes to a shortened incubation period. Prion; 5(SUPPL. 1): 50.
1640. Zalyalova ZA, Khasanova DM. Stiff person syndrome associated with autoimmune thyroiditis and sarcoidosis of intrathoracic lymph nodes: Case report. Parkinsonism and Related Disorders 2012; 18(SUPPL. 2): S75.
1641. Zanetti C, Cerutti A, Biffanti R, et al. Autoimmune-mediated congenital heart block: Long-term cardiovascular outcome in a tertiary centre experience. European Heart Journal, Supplement 2020; 22(SUPPL N): N50-N1.
1642. Zeiger RS, Druce HM, Rachelefsky GS. Prospects for ancillary treatment of sinusitis in the 1990s. Journal of Allergy and Clinical Immunology 1992; 90(3 II): 478-95.
1643. Zelber-Sagi S, Bord S, Dror-Lavi G, et al. Role of illness perception and self-efficacy in lifestyle modification among non-alcoholic fatty liver disease patients. World J Gastroenterol 2017; 23(10): 1881-90.
1644. Zeng LX. Clinical observation of the eight are scattered with Six Ingredient Rehmmania Pill for treating female urethral syndrome with yin deficiency and damp heat syndrome. Dissertation for master degree of beijing university of chinese medicine [beijing zhongyiyao daxue shuoshi xuewei lunwen] 2014: 1-30.
1645. Zenone T, Streichenberger N, Puget M. Camptocormia as a clinical manifestation of polymyositis/systemic sclerosis overlap myositis associated with anti-Ku. Rheumatol Int 2013; 33(9): 2411-5.
1646. Zhang D, Zhu A. [Pathogenesis and treatment of slow transit constipation]. Zhonghua Wei Chang Wai Ke Za Zhi 2016; 19(12): 1447-50.
1647. Zhang H. Buried cyclo(Arg-Gly-Tyr(Me)-Lys-Glu) microbubbles. Molecular Imaging and Contrast Agent Database (MICAD). Bethesda (MD): National Center for Biotechnology Information (US); 2004.
1648. Zhang J, Lyu T, Cao Y, Feng H. Role of TCF-1 in differentiation, exhaustion, and memory of CD8+ T cells: A review. FASEB Journal 2021; 35(5): e21549.
1649. Zhang X, Li S, Modis Y, et al. Functional assessment and structural basis of antibody binding to human papillomavirus capsid. Rev Med Virol 2016; 26(2): 115-28.
1650. Zhang Y-h, Liu J, Li X-f, Jia C-s. Advances of studies on acupuncture and moxibustion for exercise-induced fatigue. Zhongguo zhen jiu = Chinese acupuncture & moxibustion 2010; 30(3): 261-4.
1651. Zhao Q, Li S, Yu H, Xia N, Modis Y. Virus-like particle-based human vaccines: quality assessment based on structural and functional properties. Trends Biotechnol 2013; 31(11): 654-63.
1652. Zhou Y, Wang X, Zhao Y, et al. Elevated Thyroid Peroxidase Antibody Increases Risk of Post-partum Depression by Decreasing Prefrontal Cortex BDNF and 5-HT Levels in Mice. Front Cell Neurosci 2016; 10: 307.
1653. Zhu A, Zhang D. Pathogenesis and treatment of slow transit constipation. Zhonghua wei chang wai ke za zhi = Chinese journal of gastrointestinal surgery 2016; 19(12): 1447-50.
1654. Zhu S, Chen P, Chen Y, Li M, Chen C, Lu H. 3D-Printed Extracellular Matrix/Polyethylene Glycol Diacrylate Hydrogel Incorporating the Anti-inflammatory Phytomolecule Honokiol for Regeneration of Osteochondral Defects. American Journal of Sports Medicine 2020; 48(11): 2808-18.
1655. Zhu W, Feng J, Li C, et al. COVID-19 Risk Assessment for the Tokyo Olympic Games. Front Public Health 2021; 9: 730611.

1656. Zimmermann P, Curtis N. Factors That Influence the Immune Response to Vaccination. Clin Microbiol Rev 2019; 32(2).
1657. Zinn JR, Wright S, Howard E, et al. LPS-induced TLR4 activation during prolonged running and cycling events in hot and humid environments. FASEB Journal 2017; 31(1 Supplement 1).
1658. Zinn Z, Pyles M, Teng J, Khuu P. Cannabidiol (CBD) oil use in epidermolysis bullosa. Pediatric Dermatology 2017; 34(Supplement 1): S17.
1659. Zinzani PL, Samaniego F, Fowler NH, et al. Umbralisib, the Once Daily Dual Inhibitor of PI3Kdelta and Casein Kinase-1epsilon Demonstrates Clinical Activity in Patients with Relapsed or Refractory Indolent Non-Hodgkin Lymphoma: Results from the Phase 2 Global Unity-NHL Trial. Blood 2020; 136(Supplement 1): 34-5.
1660. Zogas N, Tsolaki E, Scouras Z, et al. The ex-vivo tolerance induction to TLR7 in donor lymphocytes prevents AGvHD in a mismatched transplantation mouse model. Blood 2013; 122(21).
1661. Zu Y, Li D, Lei X, Zhang H. Effects of the Chinese herbal formula San-Huang Gu-Ben Zhi-Ke treatment on stable chronic obstructive pulmonary disease: study protocol of a randomized, double-blind, placebo-controlled trial. Trials 2019; 20(1): 647.
1662. Zuercher E, Casillas A, Hagon-Traub I, Bordet J, Burnand B, Peytremann-Bridevaux I. Baseline data of a population-based cohort of patients with diabetes in Switzerland (CoDiab-VD). Swiss Med Wkly 2014; 144: w13951.
1663. Zuercher E, Diatta ID, Burnand B, Peytremann-Bridevaux I. Health literacy and quality of care of patients with diabetes: A cross-sectional analysis. Primary Care Diabetes 2017; 11(3): 233-40.
1664. ZuWallack R. The nonpharmacologic treatment of chronic obstructive pulmonary disease: Advances in our understanding of pulmonary rehabilitation. Proceedings of the American Thoracic Society 2007; 4(7): 549-53.
1665. Хайменова Г, Шилкина Л, Бабанина М, Волченко Г, Ткаченко М, Ждан В. [Evaluation of therapy efficiency in patients with combined course of copd and osteoarthritis]. Wiad Lek 2016; 69(2 Pt 2): 214-8.
1666. 中川 ひ, 田中 喜, 笹井 浩, 西澤 祐, 伊藤 雅. 肥満を有する大腸がん患者の周術期における減量の必要性. / Need for peri-operative weight loss among obese colorectal cancer patients. Japanese Journal of Physical Fitness & Sports Medicine 2018; 67(2): 147-55.
1667. Aarrestad Provan S, Kristianslund E, Berg IJ, Solveig Dagfinrud H, Halvorsen Sveaas S. High intensity exercise has comparable 3-month effectiveness to tnf-inhibitors on disease activity in patients with axial spondyloarthritis. post-hoc analyses of data from (the ESPA trial). Annals of the rheumatic diseases 2020; 79(SUPPL 1): 417-8.
1668. Aksenova AM, Teslenko OI, Boganskaia OA. Changes in the immune status of peptic ulcer patients after combined treatment including deep massage. Voprosy kurortologii, fizioterapii, i lechebnoi fizicheskoi kultury 1999; (2): 19-20.
1669. Alwihed RA, Taherh HL, Baiji MO, Yasser IK. Physiochemical changes in physical activity in the immune system to prevent corona virus in football players. Indian Journal of Forensic Medicine and Toxicology 2021; 15(3): 1188-93.
1670. Andrade S, Ribeiro A, Teixeira L, et al. Development of an anti-ghrelin vaccine for obesity treatment. Endocrine Reviews 2011; 32(3 Meeting Abstracts).
1671. Apostolopoulos V, Pudkasam S, Chinlumprasert N, et al. The PAPHIO study protocol: a randomised controlled trial with a 2 x 2 crossover design of physical activity adherence, psychological health and immunological outcomes in breast cancer survivors. BMC public health 2020; 20(1): 696.
1672. Arroyo-Morales M, Olea N, Ruiz C, et al. Massage after exercise--responses of immunologic and endocrine markers: a randomized single-blind placebo-controlled study. Journal of strength and conditioning research / National Strength & Conditioning Association 2009; 23(2): 638-44.
1673. Ashkenazi S, Vertruyen A, Arístegui J, et al. Superior relative efficacy of live attenuated influenza vaccine compared with inactivated influenza vaccine in young children with recurrent respiratory tract infections. Pediatric infectious disease journal 2006; 25(10): 870-9.

1674. Avloniti AA, Douda HT, Tokmakidis SP, Kortsaris AH, Papadopoulou EG, Spanoudakis EG. Acute Effects of Soccer Training on White Blood Cell Count in Elite Female Players. International Journal of Sports Physiology & Performance 2007; 2(3): 239-49.
1675. Baker FL, Smith KA, Zúñiga TM, et al. Acute exercise increases immune responses to SARS CoV-2 in a previously infected man. Brain Behav Immun Health 2021; 18: 100343.
1676. Bauer JM, De Castro A, Bosco N, et al. Influenza vaccine response in community-dwelling German prefrail and frail individuals. Immun Ageing 2017; 14: 17.
1677. Bergendiová K, Tibenská E. Imuno-modulačný efekt beta-(1,3/1,6)-D-glukanu u vrcholových športovcov. / Effects of physical activity and sport on the immune system and possibilities of their modulation. Medicina Sportiva Bohemica et Slovaca 2009; 18(4): 163-74.
1678. Bessa A, Lopez JC, Di Masi F, Ferry F, Costa E Silva G, Martins Dantas EH. Lymphocyte CD4+ cell count, strength improvements, heart rate and body composition of HIV-positive patients during a 3-month strength training program. The Journal of sports medicine and physical fitness 2017; 57(7-8): 1051-6.
1679. Blazek AD, Anderson PJ, Brichler JG, et al. Effects of a Simulated Altitude Device on Endurance Performance and Mucosal Immunity. Journal of Exercise Physiology Online 2014; 17(6): 45-57.
1680. Bloming D, Lechtermann A, Volker K, Lerch MM, Mooren FC. Lymphocyte apoptosis after exhaustive and moderate exercise. Journal of Applied Physiology 2002; 93(1): 147-53.
1681. Boussetta N, Abedelmalek S, Aloui K, Souissi N. The effect of strength training by electrostimulation at a specific time of day on immune response and anaerobic performances during short-term maximal exercise. Biological rhythm research 2017; 48(1): 157-74.
1682. Breitbart P, Meister S, Meyer T, Gärtner BC. Incidence and Prevalence of Borrelia burgdorferi Antibodies in Male Professional Football Players. Clinical Journal of Sport Medicine 2021; 31(4): e200-e6.
1683. Campbell PT, Wener MH, Sorensen B, et al. Effect of exercise on in vitro immune function: A 12-month randomized, controlled trial among postmenopausal women. Journal of Applied Physiology 2008; 104(6): 1648-55.
1684. Cao Dinh H, Bautmans I, Beyer I, et al. Six weeks of strength endurance training decreases circulating senescence-prone T-lymphocytes in cytomegalovirus seropositive but not seronegative older women. Immun Ageing 2019; 16: 17.
1685. Clemente-Suárez VJ, Beltrán-Velasco AI, Ramos-Campo DJ, et al. Physical activity and COVID-19. The basis for an efficient intervention in times of COVID-19 pandemic. Physiol Behav 2022; 244: 113667.
1686. Colburn A, Wright S, Lopez V, et al. Aerobic exercise and environmental heat stress as adjuvants to seasonal influenza vaccine. FASEB Journal 2018; 32(1 Supplement 1).
1687. Córdova A, Sureda A, Tur JA, Pons A. Immune response to exercise in elite sportsmen during the competitive season. J Physiol Biochem 2010; 66(1): 1-6.
1688. Curtis N, Zimmermann P. Factors that influence the immune response to vaccination. Clinical Microbiology Reviews 2019; 32(2): e00084-18.
1689. Edwards KM, Pascoe AR, Fiatarone-Singh MA, Singh NA, Kok J, Booy R. A randomised controlled trial of resistance exercise prior to administration of influenza vaccination in older adults. Brain, behavior, and immunity 2015; 49: e24-e5.
1690. Edwards KM, Pascoe AR, Maria AFS, et al. Exercise as an adjuvant for influenza vaccination in older adults. Psychosomatic Medicine 2013; 75(3): A-81.
1691. Eriksson MD, McKinley PS, McIntyre KM, Choo T-HJ, Lee S, Sloan RP. Exercise and heart rate variability on the antibody response to influenza vaccination. Psychosomatic Medicine 2015; 77(3): A22-A3.
1692. Fernandez-Lazaro D, Gonzalez-Bernal JJ, Sanchez-Serrano N, Navascues LJ, Del Rio AA, Mielgo-Ayuso J. Physical exercise as a multimodal tool for COVID-19: Could it be used as a

preventive strategy? International Journal of Environmental Research and Public Health 2020; 17(22): 1-13.
1693. Fleshner M. Exercise and neuroendocrine regulation of antibody production: Protective effect of physical activity on stress-induced suppression of the specific antibody response. International Journal of Sports Medicine, Supplement 2000; 21(1): s14-s9.
1694. Gärtner B, Meyer T. Vaccination in Elite Athletes. Sports Medicine 2014; 44(10): 1361-76.
1695. Gleeson M, Bishop NC, Struszczak L. Effects of Lactobacillus casei Shirota ingestion on common cold infection and herpes virus antibodies in endurance athletes: a placebo-controlled, randomized trial. European journal of applied physiology 2016; 116(8): 1555-63.
1696. Goldbaum E, Edwards K. Can Sufficient Physical Activity Improve Vaccination Experience? Medicine & Science in Sports & Exercise 2021; 53: 366-.
1697. Gomes-Santos IL. Exercise training boosts CTL infiltration and sensitizes tumors to immune checkpoint blockade. Cancer Research 2021; 81(13 SUPPL).
1698. Gomes-Santos IL, Amoozgar Z, Kumar AS, et al. Exercise Training Improves Tumor Control by Increasing CD8(+) T-cell Infiltration via CXCR3 Signaling and Sensitizes Breast Cancer to Immune Checkpoint Blockade. Cancer Immunol Res 2021; 9(7): 765-78.
1699. Grande AJ, Nunan D, Reid H, Thomas EE, Foster C. Exercise prior to influenza vaccination for limiting influenza incidence and its related complications in adults. Cochrane Database of Systematic Reviews 2015; 2015(9): CD011857.
1700. Grande AJ, Reid H, Thomas EE, Nunan D, Foster C. Exercise prior to influenza vaccination for limiting influenza incidence and its related complications in adults. Cochrane Database of Systematic Reviews 2016; 2016(8): CD011857.
1701. Grant RW, Mariani RA, Vieira VJ, et al. Cardiovascular exercise intervention improves the primary antibody response to keyhole limpet hemocyanin (KLH) in previously sedentary older adults. Brain Behav Immun 2008; 22(6): 923-32.
1702. Hara Y, Hagihara A, Ikematu H, Nobutomo K. Efficacy of influenza vaccine among elderly patients by physical activity status. Environmental Health & Preventive Medicine 2002; 7(5): 183-8.
1703. Howe WB. Preventing infectious disease in sports. Physician & Sportsmedicine 2003; 31(2): 23-9.
1704. Krzywaiiński J, Kuchar E, Pokrywka A, et al. Safety and Impact on Training of the Influenza Vaccines in Elite Athletes Participating in the Rio 2016 Olympics. Clinical Journal of Sport Medicine 2021; 31(5): 423-9.
1705. Krzywański J, Nitsch-Osuch A, Mikulski T, et al. Antibody Response to Trivalent Influenza Vaccine in the Northern and the Southern Hemisphere in Elite Athletes. Adv Exp Med Biol 2018; 1108: 49-54.
1706. Mariani RA, Vieira VJ, Keylock KT, et al. Cardiovascular exercise intervention improves the primary antibody response to keyhole limpet hemocyanin (KLH) in previously sedentary older adults. Brain, Behavior, and Immunity 2008; 22(6): 923-32.
1707. Milush JM, Gurfein BT, York VA, et al. Effect of a mindfulness-enhanced weight loss program on seasonal influenza vaccine-mediated immune responses. Psychosomatic medicine 2015; 77(3): A21.
1708. Ramírez-Vélez R, García-Hermoso A, Correa-Rodríguez M, et al. Effects of Different Doses of Exercise on Inflammation Markers Among Adolescents with Overweight/Obese: HEPAFIT Study. J Clin Endocrinol Metab 2022.
1709. Reavis RD, Ebbs JB, Onunkwo AK, Sage LM. A self-affirmation exercise does not improve intentions to vaccinate among parents with negative vaccine attitudes (and may decrease intentions to vaccinate). PLoS ONE 2017; 12(7): e0181368.
1710. Schmader KE, Levin MJ, Grupping K, et al. The Impact of Reactogenicity After the First Dose of Recombinant Zoster Vaccine on the Physical Functioning and Quality of Life of Older Adults:

An Open-Label, Phase III Trial. Journals of Gerontology Series A: Biological Sciences & Medical Sciences 2019; 74(8): 1217-24.
1711. Schuler P, Leblanc P, Soloman J, Westerfield R. The effect of physical activity of aging subjects on the production of specific antibody in response influenza vaccine (Les effets d'une activite physique de sujets ages sur la production d'anticorps specifiques en reponse au vaccin contre la grippe). Research Quarterly for Exercise & Sport 1995; 66(suppl.): A27-A.
1712. Schuler PB, Abadie BR, Collins RK. The effect of physical activity on the production of specific antibody in response to influenza vaccine in college students. Sports Medicine, Training and Rehabilitation 1997; 8(1): 85.
1713. Smith KA, Zuniga TM, Batatinha H, et al. Acute exercise increases immune responses to SARS CoV-2 in a previously infected man. Brain, Behavior, and Immunity - Health 2021; 18: 100343.
1714. Veljkovic M, Veljkovic V, Glisic S, et al. Aerobic exercise training as a potential source of natural antibodies protective against human immunodeficiency virus-1. Scandinavian Journal of Medicine and Science in Sports 2010; 20(3): 469-74.
1715. Wang J, Song H, Tang X, et al. Effect of exercise training intensity on murine T-regulatory cells and vaccination response. Scandinavian Journal of Medicine & Science in Sports 2012; 22(5): 643-52.
1716. White P, Fulcher K, Bertolli J, Pangi C, Frerichs R, Halloran ME. A randomised controlled trial of graded exercise therapy in patients with a chronic fatigue: a case-control study of the effectiveness of BCG vaccine for preventing leprosy in Yangon, Myanmar. Int-j-epidemiol 1997; 26: 888-96.
1717. Woolpert T, Phillips CJ, Sevick C, Crum-Cianflone NF, Blair PJ, Faix D. Health-related behaviors and effectiveness of trivalent inactivated versus live attenuated influenza vaccine in preventing influenza-like illness among young adults. PLoS One 2014; 9(7): e102154.
1718. Yu Y, Lau MMC, Jiang H, Lau JTF. Prevalence and factors of the performed or scheduled covid-19 vaccination in a chinese adult general population in hong kong. Vaccines 2021; 9(8): 847.
1719. Yucheng Q. The research progress on the influence of exercise on vaccine immune response. Journal of Physical Education / Tiyu Xuekan 2022; 29(1): 138-44.

References used in the supplement

1. Bohn-Goldbaum, E.; Pascoe, A.R.; Singh, M.; Singh, N.; Kok, J.; Dwyer, D.E.; Mathieson, E.; Booy, R.; Edwards, C. Acute exercise decreases vaccine reactions following influenza vaccination among older adults. *Brain, behavior, and immunity - health* **2020**, *1*, doi:10.1016/j.bbih.2019.100009.
2. Woods, J.A.; Keylock, K.T.; Lowder, T.; Vieira, V.J.; Zelkovich, W.; Dumich, S.; Colantuano, K.; Lyons, K.; Leifheit, K.; Cook, M.; et al. Cardiovascular exercise training extends influenza vaccine seroprotection in sedentary older adults: the immune function intervention trial. *J Am Geriatr Soc* **2009**, *57*, 2183-2191, doi:10.1111/j.1532-5415.2009.02563.x.
3. Elzayat, M.T.; Markofski, M.M.; Simpson, R.J.; Laughlin, M.; LaVoy, E.C. No Effect of Acute Eccentric Resistance Exercise on Immune Responses to Influenza Vaccination in Older Adults: A Randomized Control Trial. *Front Physiol* **2021**, *12*, 713183, doi:10.3389/fphys.2021.713183.
4. Campbell, J.P.; Edwards, K.M.; Ring, C.; Drayson, M.T.; Bosch, J.A.; Inskip, A.; Long, J.E.; Pulsford, D.; Burns, V.E. The effects of vaccine timing on the efficacy of an acute eccentric exercise intervention on the immune response to an influenza vaccine in young adults. *Brain Behav Immun* **2010**, *24*, 236-242, doi:10.1016/j.bbi.2009.10.001.
5. Edwards, K.M.; Burns, V.E.; Allen, L.M.; McPhee, J.S.; Bosch, J.A.; Carroll, D.; Drayson, M.; Ring, C. Eccentric exercise as an adjuvant to influenza vaccination in humans. *Brain Behav Immun* **2007**, *21*, 209-217, doi:10.1016/j.bbi.2006.04.158.
6. Edwards, K.M.; Campbell, J.P.; Ring, C.; Drayson, M.T.; Bosch, J.A.; Downes, C.; Long, J.E.; Lumb, J.A.; Merry, A.; Paine, N.J.; et al. Exercise intensity does not influence the efficacy of eccentric exercise as a behavioural adjuvant to vaccination. *Brain Behav Immun* **2010**, *24*, 623-630, doi:10.1016/j.bbi.2010.01.009.
7. Kohut, M.L.; Arntson, B.A.; Lee, W.; Rozeboom, K.; Yoon, K.J.; Cunnick, J.E.; McElhaney, J. Moderate exercise improves antibody response to influenza immunization in older adults. *Vaccine* **2004**, *22*, 2298-2306, doi:10.1016/j.vaccine.2003.11.023.
8. Kohut, M.L.; Lee, W.; Martin, A.; Arnston, B.; Russell, D.W.; Ekkekakis, P.; Yoon, K.J.; Bishop, A.; Cunnick, J.E. The exercise-induced enhancement of influenza immunity is mediated in part by improvements in psychosocial factors in older adults. *Brain Behav Immun* **2005**, *19*, 357-366, doi:10.1016/j.bbi.2004.12.002.
9. Bohn-Goldbaum, E.; Lee, V.Y.; Skinner, S.R.; Frazer, I.H.; Khan, B.A.; Booy, R.; Edwards, K.M. Acute exercise does not improve immune response to HPV vaccination series in adolescents. *Papillomavirus Res* **2019**, *8*, 100178, doi:10.1016/j.pvr.2019.100178.
10. Edwards, K.M.; Burns, V.E.; Adkins, A.E.; Carroll, D.; Drayson, M.; Ring, C. Meningococcal A vaccination response is enhanced by acute stress in men. *Psychosom Med* **2008**, *70*, 147-151, doi:10.1097/PSY.0b013e318164232e.
11. Edwards, K.M.; Burns, V.E.; Reynolds, T.; Carroll, D.; Drayson, M.; Ring, C. Acute stress exposure prior to influenza vaccination enhances antibody response in women. *Brain Behav Immun* **2006**, *20*, 159-168, doi:10.1016/j.bbi.2005.07.001.
12. Edwards, K.M.; Pung, M.A.; Tomfohr, L.M.; Ziegler, M.G.; Campbell, J.P.; Drayson, M.T.; Mills, P.J. Acute exercise enhancement of pneumococcal vaccination response: a randomised controlled trial of weaker and stronger immune response. *Vaccine* **2012**, *30*, 6389-6395, doi:10.1016/j.vaccine.2012.08.022.
13. Hayney, M.S.; Coe, C.L.; Muller, D.; Obasi, C.N.; Backonja, U.; Ewers, T.; Barrett, B. Age and psychological influences on immune responses to trivalent inactivated influenza vaccine in the meditation or exercise for preventing acute respiratory infection (MEPARI) trial. *Hum Vaccin Immunother* **2014**, *10*, 83-91, doi:10.4161/hv.26661.

14. Long, J.E.; Ring, C.; Bosch, J.A.; Eves, F.; Drayson, M.T.; Calver, R.; Say, V.; Allen, D.; Burns, V.E. A life-style physical activity intervention and the antibody response to pneumococcal vaccination in women. *Psychosom Med* **2013**, *75*, 774-782, doi:10.1097/PSY.0b013e3182a0b664.
15. Long, J.E.; Ring, C.; Drayson, M.; Bosch, J.; Campbell, J.P.; Bhabra, J.; Browne, D.; Dawson, J.; Harding, S.; Lau, J.; et al. Vaccination response following aerobic exercise: can a brisk walk enhance antibody response to pneumococcal and influenza vaccinations? *Brain Behav Immun* **2012**, *26*, 680-687, doi:10.1016/j.bbi.2012.02.004.
16. Ranadive, S.M.; Cook, M.; Kappus, R.M.; Yan, H.; Lane, A.D.; Woods, J.A.; Wilund, K.R.; Iwamoto, G.; Vanar, V.; Tandon, R.; et al. Effect of acute aerobic exercise on vaccine efficacy in older adults. *Med Sci Sports Exerc* **2014**, *46*, 455-461, doi:10.1249/MSS.0b013e3182a75ff2.
17. Monteiro, F.R.; Roseira, T.; Amaral, J.B.; Paixão, V.; Almeida, E.B.; Foster, R.; Sperandio, A.; Rossi, M.; Amirato, G.R.; Apostólico, J.S.; et al. Combined Exercise Training and l-Glutamine Supplementation Enhances Both Humoral and Cellular Immune Responses after Influenza Virus Vaccination in Elderly Subjects. *Vaccines (Basel)* **2020**, *8*, 685, doi:10.3390/vaccines8040685.
18. Whitham, M.; Blannin, A.K. The effect of exercise training on the kinetics of the antibody response to influenza vaccination. *J Sports Sci* **2003**, *21*, 991-1000, doi:10.1080/0264041031000140464.
19. Bachi, A.L.; Suguri, V.M.; Ramos, L.R.; Mariano, M.; Vaisberg, M.; Lopes, J.D. Increased production of autoantibodies and specific antibodies in response to influenza virus vaccination in physically active older individuals. *Results Immunol* **2013**, *3*, 10-16, doi:10.1016/j.rinim.2013.01.001.
20. Bruunsgaard, H.; Hartkopp, A.; Mohr, T.; Konradsen, H.; Heron, I.; Mordhorst, C.H.; Pedersen, B.K. In vivo cell-mediated immunity and vaccination response following prolonged, intense exercise. / Immunite a mediation cellulaire, in-vivo et reponse a la vaccination suivant un exercice intense prolonge. *Medicine & Science in Sports & Exercise* **1997**, *29*, 1176-1181.
21. Yang, Y.; Verkuilen, J.; Rosengren, K.S.; Mariani, R.A.; Reed, M.; Grubisich, S.A.; Woods, J.A. Effects of a Taiji and Qigong intervention on the antibody response to influenza vaccine in older adults. *Am J Chin Med* **2007**, *35*, 597-607, doi:10.1142/s0192415x07005090.
22. Felismino, E.S.; Santos, J.M.B.; Rossi, M.; Santos, C.A.F.; Durigon, E.L.; Oliveira, D.B.L.; Thomazelli, L.M.; Monteiro, F.R.; Sperandio, A.; Apostólico, J.S.; et al. Better Response to Influenza Virus Vaccination in Physically Trained Older Adults Is Associated With Reductions of Cytomegalovirus-Specific Immunoglobulins as Well as Improvements in the Inflammatory and CD8(+) T-Cell Profiles. *Front Immunol* **2021**, *12*, 713763, doi:10.3389/fimmu.2021.713763.
23. de Araújo, A.L.; Silva, L.C.; Fernandes, J.R.; Matias Mde, S.; Boas, L.S.; Machado, C.M.; Garcez-Leme, L.E.; Benard, G. Elderly men with moderate and intense training lifestyle present sustained higher antibody responses to influenza vaccine. *Age (Dordr)* **2015**, *37*, 105, doi:10.1007/s11357-015-9843-4.
24. Gleeson, M.; Pyne, D.B.; McDonald, W.A.; Clancy, R.L.; Cripps, A.W.; Horn, P.L.; Fricker, P.A. Pneumococcal antibody responses in elite swimmers. *Clin Exp Immunol* **1996**, *105*, 238-244, doi:10.1046/j.1365-2249.1996.d01-752.x.
25. Keshtkar-Jahromi, M.; Vakili, H.; Rahnavardi, M.; Gholamin, S.; Razavi, S.M.; Eskandari, A.; Sadeghi, R.; Vatan-Pour, H.; Keshtkar-Jahromi, M.; Haghighat, B.; et al. Antibody response to influenza immunization in coronary artery disease patients: a controlled trial. *Vaccine* **2010**, *28*, 110-113, doi:10.1016/j.vaccine.2009.09.108.
26. Keylock, K.T.; Lowder, T.; Leitbeit, K.A.; Cook, M.; Mariani, R.A.; Ross, K.; Kim, K.; Chapman-novakofski, K.; McAuley, E.; Woods, J.A. Higher antibody, but not cell-mediated, responses to vaccination in high physically fit elderly. *Journal of Applied Physiology* **2007**, *102*, 1090-1098.
27. Kohut, M.L.; Cooper, M.M.; Nickolaus, M.S.; Russell, D.R.; Cunnick, J.E. Exercise and psychosocial factors modulate immunity to influenza vaccine in elderly individuals. *J Gerontol A Biol Sci Med Sci* **2002**, *57*, M557-562, doi:10.1093/gerona/57.9.m557.

28. Ledo, A.; Schub, D.; Ziller, C.; Enders, M.; Stenger, T.; Gärtner, B.C.; Schmidt, T.; Meyer, T.; Sester, M. Elite athletes on regular training show more pronounced induction of vaccine-specific T-cells and antibodies after tetravalent influenza vaccination than controls. *Brain Behav Immun* **2020**, *83*, 135-145, doi:10.1016/j.bbi.2019.09.024.
29. Schuler, P.B.; Lloyd, L.K.; Leblanc, P.A.; Clapp, T.A.; Abadie, B.R.; Collins, R.K. The effect of physical activity and fitness on specific antibody production in college students. / Effet de l ' activite physique et de la condition physique sur la production d ' anticorps chez des lyceens, suite a une vaccination. *Journal of Sports Medicine & Physical Fitness* **1999**, *39*, 233-239.
30. Kenzaka, T.; Goda, K.; Kamada, M.; Okayama, M.; Yahata, S.; Kumabe, A. Effects of vaccination day routine activities on influenza vaccine efficacy and vaccination-induced adverse reaction incidence: a cohort study. *Vaccines (Basel)* **2021**, *9*, 753, doi:http://dx.doi.org/10.3390/vaccines9070753.
31. Mitsunaga, T.; Ohtaki, Y.; Seki, Y.; Yoshioka, M.; Mori, H.; Suzuka, M.; Mashiko, S.; Takeda, S.; Mashiko, K. The evaluation of factors affecting antibody response after administration of the BNT162b2 vaccine: a prospective study in Japan. *PeerJ* **2021**, *9*, e12316, doi:10.7717/peerj.12316.
32. Turner, S.E.G.; Hull, J.H.; Jackson, A.; Loosemore, M.; Ranson, C.; Kelleher, P.; Shah, A. Screening Identifies Suboptimal Vaccination Protection in Illness-Susceptible Elite Athletes. *Clinical journal of sport medicine : official journal of the Canadian Academy of Sport Medicine* **2021**, *31*, e470-e472, doi:https://dx.doi.org/10.1097/JSM.0000000000000969.
33. Ayling, K.; Fairclough, L.; Tighe, P.; Todd, I.; Halliday, V.; Garibaldi, J.; Royal, S.; Hamed, A.; Buchanan, H.; Vedhara, K. Positive mood on the day of influenza vaccination predicts vaccine effectiveness: A prospective observational cohort study. *Brain Behav Immun* **2018**, *67*, 314-323, doi:10.1016/j.bbi.2017.09.008.
34. Schuler, P.B.; Leblanc, P.A.; Marzilli, T.S. Effect of physical activity on the production of specific antibody in response to the 1998-99 influenza virus vaccine in older adults. / Effet de l ' activite physique sur la production d ' anticorps specifiques en reaction au vaccin contre la grippe de 1998-1999 chez les personnes agees. *Journal of Sports Medicine & Physical Fitness* **2003**, *43*, 404-408.
35. Segerstrom, S.C.; Hardy, J.K.; Evans, D.R.; Greenberg, R.N. Vulnerability, distress, and immune response to vaccination in older adults. *Brain Behav Immun* **2012**, *26*, 747-753, doi:10.1016/j.bbi.2011.10.009.
36. Stewart, A.; Vanderkooi, O.G.; Reimer, R.A.; Doyle-Baker, P.K. Immune response in highly active young men to the 2014/2015 seasonal influenza vaccine. *Appl Physiol Nutr Metab* **2018**, *43*, 769-774, doi:10.1139/apnm-2017-0683.
37. Wong, G.C.L.; Narang, V.; Lu, Y.; Camous, X.; Nyunt, M.S.Z.; Carre, C.; Tan, C.; Xian, C.H.; Chong, J.; Chua, M.; et al. Hallmarks of improved immunological responses in the vaccination of more physically active elderly females. *Exerc Immunol Rev* **2019**, *25*, 20-33.
38. Gualano, B.; Lemes, I.R.; Silva, R.P.; Pinto, A.J.; Mazzolani, B.C.; Smaira, F.I.; Sieczkowska, S.M.; Aikawa, N.E.; Pasoto, S.G.; Medeiros-Ribeiro, A.C.; et al. Association between physical activity and immunogenicity of an inactivated virus vaccine against SARS-CoV-2 in patients with autoimmune rheumatic diseases. *Brain Behav Immun* **2021**, *101*, 49-56, doi:10.1016/j.bbi.2021.12.016.
